# Identification of ASB7 as ER stress responsive gene through a genome wide *in silico* screening for genes with ERSE

**DOI:** 10.1371/journal.pone.0194310

**Published:** 2018-04-09

**Authors:** Vivek Vishnu Anasa, Madhumathi Manickam, Priti Talwar, Palaniyandi Ravanan

**Affiliations:** Apoptosis and Cell Survival Research Lab, Department of Biosciences, School of Biosciences and Technology, VIT University, Vellore, Tamil Nadu, India; Duke University School of Medicine, UNITED STATES

## Abstract

The endoplasmic reticulum (ER) not only performs its basic function of regulating calcium homeostasis, lipid biosynthesis, folding, modifying and transporting proteins but also plays a decisive role in regulating multiple cellular processes ranging from cell growth and differentiation to apoptosis and autophagy. Disturbances in ER homeostasis initiate the unfolded protein response (UPR) implicated in the pathogenesis of many human diseases. Drugging the UPR components for therapeutic interventions has received considerable attention. The purpose of this study is to identify genes that are previously unsuspected to be regulated under ER stress. Because ER stress-inducible gene expression is majorly regulated under ERSE elements, we screened human genome by adopting an *in silico* approach using ERSE elements (I, II, III) as probes and identified 337 candidate genes. Having knowledge of the importance of E3 ubiquitin ligase in the ERAD machinery; we validated our preliminary search by focusing on one of the hits i.e. ASB7 gene that encodes E3 ubiquitin ligase. In HeLa cells, we found that pharmacological induction of ER stress led to an increase in the expression of ASB7 with simultaneous activation of UPR pathways. Although knockdown of ASB7 expression leads to significant reduction in GRP78 and CHOP mRNA levels, it did not protect cells from ER stress-induced cell death. Also, an up-regulation in the expression of pro-inflammatory genes like TNF-α and IL-1β in ASB7 knockdown cells was observed under ER stress. Collectively, our findings suggest that ASB7 is regulated under ER stress and this study also identifies several other genes that could apparently be regulated under ER stress.

## Introduction

ER is an essential organelle involved in various cellular processes including protein folding, sorting and transportation [1, 2]. Proteins enter the ER as unfolded polypeptides, from which they change into their correct conformation; then these secreted and transmembrane proteins are transported to the desired destination [3]. Cellular disturbances, inefficient clearance of misfolded proteins or change in the Ca^2+^ homeostasis leads to accumulation of unfolded proteins in the ER. The ER responds by increasing its protein folding capacity through specialized signaling pathways that are collectively known as the UPR which restores the ER protein homeostasis and further regulates cell survival [4, 5]. UPR increases transcription of genes encoding enzymes and chaperones involved in protein folding, secretion and degradation of misfolded proteins, and thereby constituting a coordinated regulatory mechanism that restores protein-folding in the ER and re-establishes normal cellular function [6, 7].

The UPR pathway is a highly conserved mechanism between yeast and human. UPR is a linear signaling pathway in budding yeast controlling the expression of numerous genes in response to ER stress [7]. Meanwhile, in mammalian cells, the UPR has diversified and comprises at least three parallel signaling sensors in the membrane of ER that respond to increased levels of unfolded proteins: IRE-1α (inositol-requiring kinase-1), ATF6 (activating transcription factor 6) and PERK (RNA-dependent protein kinase-like ER kinase) [8, 9]. During unstressed conditions, the ER chaperone, GRP78 binds to the luminal domains of these key regulators keeping them inactive. Upon ER stress, GRP78 dissociates from these sensors resulting in their activation [10]. IRE-1α a type I ER transmembrane kinase undergoes auto phosphorylation, which activates its intrinsic RNase activity and leads to splicing of XBP1 mRNA to produce the active transcription factor sXBP1. ATF6 is a type II ER transmembrane transcription factor which is proteolytically cleaved upon trafficking to the Golgi apparatus to generate the soluble active product, which initiates a transcriptional program to relieve ER stress. Activated PERK a type I ER transmembrane kinase phosphorylates the eukaryotic initiation factor 2 (eIF2α) on the alpha subunit, resulting in an overall attenuation of mRNA translation. Although global protein production is reduced following UPR, the translation of certain mRNAs, including the transcription factor ATF4, is increased following PERK activation. Transcription factor C/EBP homologous protein (CHOP) can activate components of the cell death and promote apoptosis downstream of the UPR [11]. CHOP expression is low in non-stressed conditions but increases in response to ER stress, hypoxia and amino acid starvation in cells [12–14]. Although most of these molecular events are clearly established, the mechanism leading to the transcriptional regulation of specific genes under ER stress remains poorly understood.

ERSE (ER stress-response element) is short sequences of DNA located within a gene promoter region that contains binding sites for specific transcription factors which regulate ER stress [15]. However, many ER stress-responsive genes possess proximal promoter regions without an ERSE sequence, such as *FKBP13* [16], *asparagine synthetase* [17], *ATF3* [18], and *RTP/NDRG* [19]. ERSE possess a consensus sequence of tripartite structure CCAAT-N_9_-CCACG, (N-Nucleotides) which is necessary for the induction of the major ER chaperones (GRP and Calreticulin) [16]. The general transcription factor, NF-Y/CBF binds to the CCAAT motif of ERSE [20–22]. While under conditions of ER stress, ATF6 binds to the CCACG motif resulting in the transcriptional induction of ER chaperones. ERSE-II (ATTGG-N-CCACG) was first found in the promoter region of *Herp* gene which is involved in ER stress-associated protein degradation, and it is a direct target of lumen [23]. ERSE-II was also dependent on p50ATF6, in a manner similar to that of ERSE, despite the disparate structure. Finally, the third novel ERSE like element (CCAAT-N_26_-CCACG) was identified by luciferase reporter assays in the human PRNP promoter regulated in ER stress by XBP1, but not via ATF6 [24]. Both IRE-1/XBP-1 and ATF6 mediate transcriptional regulation of molecular chaperones that support folding in the ER and protein degradation mechanisms [25], metabolism [26] and apoptosis [27].

In eukaryotes, ASB (Ankyrin SOCS box) gene family is the largest family of SOCS box proteins, having 18 members (ASB1–18). Most of the ASB proteins have been defined as E3 ubiquitin ligases as they contain a SOCS box domain for substrate recognition forming a subset of Elongin-Cullin-SOCS (ECS) complex [28]. Despite having 18 members in humans, only a few ASB family proteins are examined for their E3 ligase activity and few subsets of substrates identified and explored to date. All members of ASB proteins have two functional domains: an ankyrin repeat region at its amino-terminal which is involved in specific protein-protein interactions, and a SOCS box domain at its carboxyl-terminal, which function as an adaptor providing a link for the degradation of proteins which are targeted by the ankyrin repeat region. Recent studies indicate the roles of ASBs in regulating both normal and pathological conditions in various systems in different vertebrates. Some of them have been reported in various cellular functions such as ubiquitination of different proteins via SOCS box domain, as degradation of TNFR *(ASB3*) [29], creatine kinase B (*ASB9)* [30, 31] but also in regulatory like functions as the inhibition of mitochondrial function by (*ASB9*) [30], spermatogenesis *(ASB9*) [32], alteration of myoblast differentiation (*ASB15*) [33] and stimulation of angiogenesis (*ASB5*) [34]. However, it remains to be determined whether any of the mammalian ASB genes are involved in UPR signaling. The majority of ASB family members remain poorly characterized, lacking in most cases *in vitro* and *in vivo* verification of their biological functions. Hence, a better understanding of ASB interaction partners in ER stress can potentially shed light on the inexplicable physiological actions of this family and unveil elusive aspects of the ubiquitination machinery as a whole.

Several approaches have been used to identify genes induced by ER stress including microarray technique, analysis of gene expression, networking, and representational difference analysis [35–37]. However, identification of ER stress-inducible targets by gene expression profiling is highly dependent on the expression level of the target gene, potentially overlooking genes with low expression in microarray techniques. In this case, *in silico* approach is an effective tool for identification and determination of novel ER stress target genes. In the present study, the aim was to identify ERSE target genes in the whole human genome using Python programming with ERSE (I, II, III) elements as probes. In our study we identified 337 candidate genes; among them, we examined the role of ASB7 gene in ER stress. We show that ER stress increases the expression of ASB7 *in vitro* conditions. However, ASB7 knockdown had reduced GRP78 and CHOP levels but had no effect on ER stress-mediated cell death in HeLa cells. Additionally, we also provide evidence that the induction of ASB7 expression seems to be involved in the PERK/ATF4/CHOP pathway during ER stress. Our results indicate that this *in silico* approach is an effective tool for identification of ERSE sequence containing genes, and help determine new targets in UPR signaling and identifies ASB7 as an ER stress-responsive gene.

## Materials and methods

### Bioinformatic analysis

#### Dataset extraction and prediction of ERSE element containing genes

To find genes in the human genome containing ERSE I-CCAAT-N_9_-CCACG, ERSE II-ATTGG-N-CCACG and ERSE III-CCAAT-N_26_-CCACG (N-nucleotides), a program was written in Python. It is a text-based programming language where the programmer type text based instructions using the language syntax to determine the function of the program and get the required results [38]. The sequences of the complete human genome were downloaded from the National Center for Biotechnology Information (NCBI) Human Assembly GRCh37.p13 (June 28, 2013) [39]. The Genome Reference Consortium (GRC) was formed in 2008 to maintain the reference assembly for the human genome. Further, the graphical sequences viewer (GSV) is used here to determine the distance between the ERSE element and their closest genes. After finding the elements by python programming, each ERSE element was subjected to the GSV in find box to pick the corresponding closest gene by using the zoom out and zoom in control (S1 Fig). By using GSV we found the gene, gene ID, gene name, ERSE location in the chromosome, gene location and distance between ERSE element and gene. Additionally, we further calculated the distance between each ERSE element and the corresponding closest gene and retrieved only the ERSE element located within 10 kb upstream and 20 kb region downstream of the gene from TSS (transcription start site).

#### *In silico* promoter analysis of ASB7 and protein-protein interactions of ASB gene family

The promoter of ASB7 was scanned further for binding of transcription factors 5 kb upstream of TSS using consensus sequence for transcription factors by using ‘MEME analysis’ tool (http://meme suite.org/tools/fime) (p-value <0.0001). Additionally, ERSE-II element in ASB7 gene sequence was checked for the evolutionary conservation in mouse, rat and zebrafish genome. Besides, this ASB7 gene and its family members were checked for protein-protein interaction network between the potential UPR components by utilizing the available physical interactions in protein databases (APID interactome, Innate DB-all, Intact, MINT) to delineate the critical regulators and the network of interactions controlling the expression of ASB genes using Cytoscape [40].

### Reagents and cell culture

Human cervical carcinoma (HeLa) cells were obtained from the NCCS, India. Cells were cultured in Dulbecco’s modified Eagle’s medium containing (DMEM, HiMedia) 10% (v/v) fetal bovine serum (FBS, HiMedia), 3.7 g/L sodium bicarbonate (HiMedia), 1X glutamax (GIBCO) antibiotic and antimycotic (HiMedia) (1X).

Tunicamycin and DTT were purchased from Sigma-Aldrich. In order to block PERK pathway GSK2606414 (Medchem express) was used to pretreat cells for 1 h before cells treated with tunicamycin.

### Cell viability assay (MTT assay)

Cell viability was measured using the MTT assay [3-(4, 5-dimethylthiazol-2-yl)-2, 5- diphenyltetrazolium bromide] [41]. It is a colorimetric assay which detects the reduction of MTT dependent on NAD (P) H-dependent oxidoreductase enzymes in cells. Cells were seeded in a 96-well plate at 1×10^4^ cells/well. Treated cells were incubated with 20 μl MTT (5 mg/ml) in sterilized PBS for 3 h at 37°C in a humidified 5% CO_2_ atmosphere. Then, the MTT solution was removed and the formazan crystals were dissolved in 200 μL / well DMSO and absorbance was read at 570 nm using a microplate reader (Bio-Rad) and 100% viability was defined as the absorbance of the control. The experiment was triplicate-performed independently. Cell survival rates were calculated according to the following equation: survival rate = [experimental absorbance value / control absorbance value] × 100%.

### Gene knockdown by RNAi transfection

Human Hela cells were reverse transfected with 0.5 μM siRNAs using Lipofectamine RNAiMax (Invitrogen) in OptiMEM medium (Invitrogen), according to the manufacturer's protocol. At 48 h after transfection, the transfection medium was replaced with the fresh culture media containing chemical inducers tunicamycin and DTT for 3, 6 and 12 h for mRNA, 6 h for protein expression and 24 h for cell viability. Human siRNAs used were purchased from Thermo scientific (Ambion, USA): *ASB7* siRNA 1# Sense strand: CGGAUGUUAUAUAAUUACGTT Antisense strand: CGUAAUUAUAUAACAUCCGTG, *ASB7* siRNA 2# Sense strand: CGAACACACGGAACUAUGATT, Antisense strand: UCAUAGUUCCGUGUGUUCGTG and control scramble siRNA.

### Quantitative RT-PCR analysis

Total RNA was extracted using RNAiso Plus reagent (Takara) from cultured cells. Subsequently, 2 μg RNA per sample was reverse transcribed using oligo (dT) and M-MLV reverse transcriptase (RT) (Takara) according to the manufacturer’s instructions. For quantification 2 μL of the resulting RT product (1:10 dilution) was used for q-PCR using 5 μM of specific primers from Sigma in conjunction with SYBR Premix Ex Taq (Takara, Siga, Japan) and AB step one instrument (Applied Biosystems). The PCR reaction was carried out for 40 thermal cycles. Expression of the target genes was analyzed by the comparative 2^−ΔΔ^*C*^t^ method and normalized using 18S rRNA levels. The primer sequence used for qRT‐PCR analysis of transcripts was as follows (Table 1):

**Table 1:** Primer sequences of genes.

S.No	Gene	Forward	Reverse
1	18s	GCCGCTAGAGGTGAAATTCT	CATTCTTGGCAAATGCTTTC
2	ASB7	TGGAGTTCAAGGCTGAGGTT	GTGTTCGTGTCTGCTCGGTA
3	GRP78	CAACCAACTGTTACAATCAAGGC	CAAAGGTGACTTCAATCTGTGG
4	CHOP	CAGAGCTGGAACCTGAGGAG	TGGATCAGTCTGGAAAAGCA
5	PERK	CAGTGGGATTTGGATGTGG	GGAATGATCATCTTATTCCCAAA
6	IRE-1	CCATCGAGCTGTGTGCAG	TGTTGAGGGAGTGGAGGTG
7	ATF6	TTGGCATTTATAATACTGAACTATA	TTTGATTTGCAGGGCTCAC
8	ATF4	GTTCTCCAGCGACAAGGCTA	ATCCTGCTTGCTGTTGTTGG,
9	sXBP-1	CCGCAGCAGGTGCAGG	GAGTCAATACCGCCAGAATCCA
10	ASB4	ATTTCCTTGAGGCGCTAAAGTC	GCTAGGCAACCAGTAACCTTGT
11	JUN	CCAAAGGATAGTGCGATGTTT	CTGTCCCTCTCCACTGCAAC,
12	TNF-α	AACATCCAACCTTCCCAAACG	GACCCTAAGCCCCCAATTCTC
13	IL-1β	AATCTGGTACCTGTCCTGCGTGTT	TGGGTAATTTTTGGGATCTACACT
14	XBP-1	CGCAGCACCTCAGACTACG	ATGTTCTGGAGGGGTGACAA
15	TRB3	ATGCGAGCCACCCCTCTAGC	CTAGCCATACAGAACCACTTC
16	DR5	GGGAGCCGCTCATGAGGAAGTTGG	GGCAAGTCTCTCTCCCAGCGTCTC

### Western blotting

Media was aspirated and cells were washed with DPBS and lysates were obtained using RIPA buffer (20 mM Tris pH-7.5, 150 mM NaCl, 1 mM Na_2_EDTA, 1 mM EGTA, 1% NP-40, 1% Sodium deoxycholate) supplemented with 1 mM PMSF, Protease inhibitor cocktail (1X, Sigma Aldrich) and Phosphatase inhibitor cocktail (1X, Phos STOP, Roche). Cell lysates were passed several times through an insulin syringe and incubated in ice for 30 min. The cellular extracts were centrifuged for 30 min at 13000 rpm at 4°C and the supernatant was collected. Total protein was estimated by Lowry method [42]. Total cellular extracts containing 50 μg protein, were separated by electrophoresis on 10–12% (w/v) SDS-polyacrylamide gel (SDS-PAGE) after denaturation at 95°C for 5 min in sample buffer (pH-8 in mM): 100 Tris, 100 DTT, 4% (v/v) SDS, 0.2% (w/v) bromophenol blue and 20% (v/v) glycerol. Proteins were then transferred to PVDF membranes (Trans W Pall Corporation), which were further blocked for 1 h at RT with 5% skim milk in Tris buffered saline-Tween20 (TBST) for 1 h. The Blots were blocked with specific rabbit anti human antibodies: ASB7 antibody (dilution, 1:1000, Novus), GRP78 Antibody (dilution, 1:1000, Abclonal); CHOP antibody (dilution, 1:1000, Abclonal), ATF4 (dilution, 1:1000, Elabsciences), eIF2α and phospho-eIF2α (dilution, 1:1000, Cell Signaling Technology), p38 and phospho-p38 (dilution, 1:1000, Cell Signaling Technology), IRE-1α and phospho-IRE-1α (dilution,1:1000, Cell Signaling Technology) for 3 h at room temperature. Membrane was then incubated for 1 h with anti-rabbit HRP conjugated secondary antibody (CST). Immunocomplexes were visualized using enhanced chemiluminescence (ECL) chemistry and developed on film (Fuji) [43]. Equal loading and transfer was validated by probing the membranes with β-actin antibody (dilution, 1:1000, Cell Signaling Technology).

### Statistical analysis

Statistical tests were performed using Graph Pad Prism5 software. All other experiments have been done twice and performed in duplicates except for MTT cell viability assay that was performed twice in triplicates. One-way ANOVA followed by bonferroni post test was applied to compare more than two groups. Two-way ANOVA followed by bonferroni post test was applied to compare multiple groups (two parameters). Results are represented as mean ± SEM and p≤0.05 is considered as significant.

## Results

### Genome-wide identification of ERSE elements

**Fig 1:**
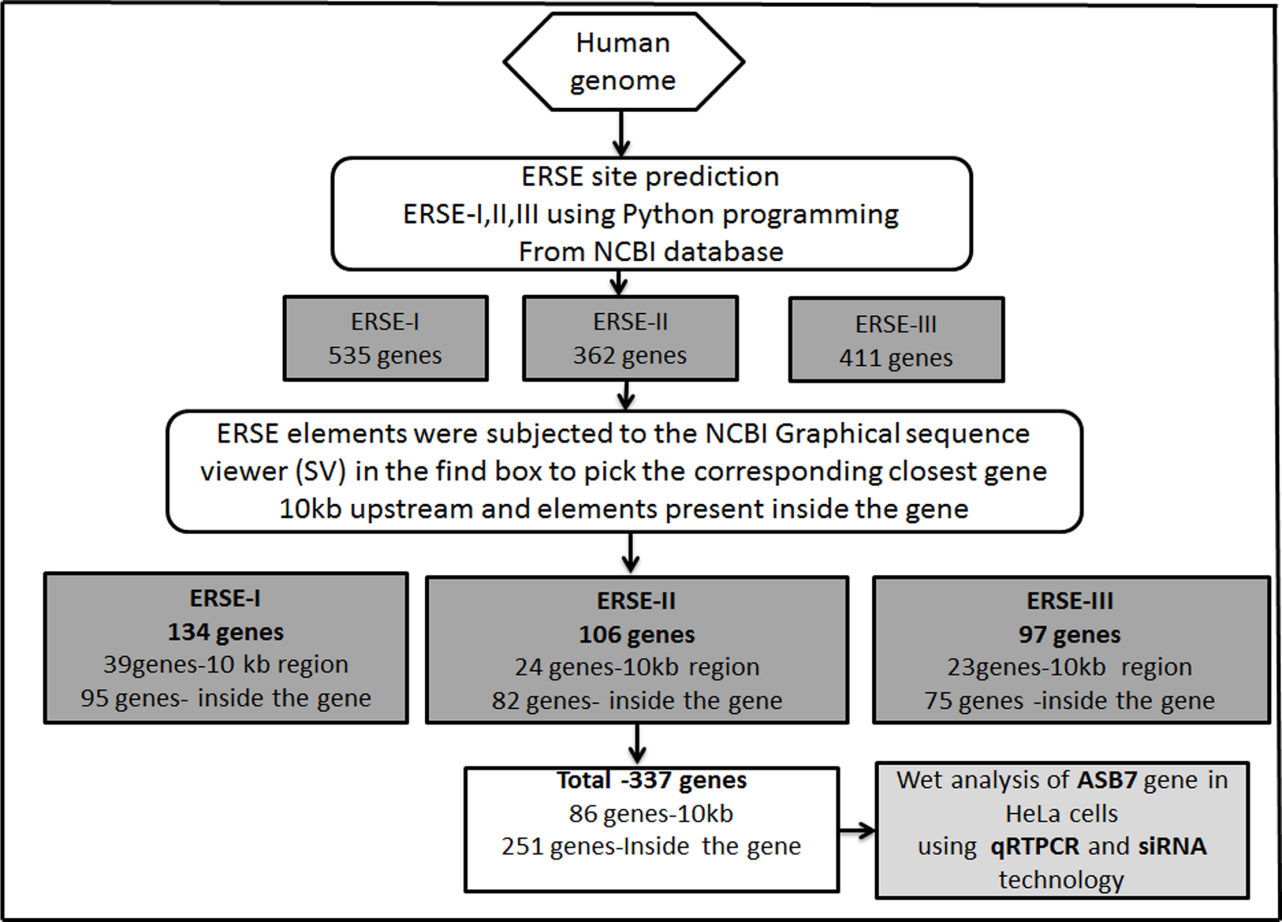
Schematic representation of strategy for identifying putative ERSE binding target genes in the human genome. Putative ERSE binding target genes in the human genome were identified using an *in silico* approach. The ERSE elements were identified from the Human Assembly GRCh37.p13 using the algorithm which was implemented in a computer program written in the python programming language.

Most of the mammalian UPR-target genes contain a cis-acting ERSE element in their promoter regions. The transcription factors bind to ERSE element and transactivate UPR target genes. As a starting point to identify genes unknown to be regulated under ER stress, we used a computational approach to search for ERSE motifs within human GRC (GRCh37.p13) (Fig 1). Accordingly, Python program was written to scan the human genome using ERSE elements: ERSE-I (CCAAT-N_9_-CCACG), ERSE-II (ATTGG-N-CCACG) and ERSE-III (CCAAT-N_26_-CCACG) as probes and found 535 ERSE-I, 362 ERSE-II and 411 ERSE-III elements with their locations summarized in S1 Table. The result of the program was considered legitimate as also got known ER stress target genes like *GRP94*, *Herp* and *PRNP* in our list of ERSE-I, II and III containing genes. The orientation of the elements within promoters of the *GRP94*, *Herp* and *PRNP* are shown in S2, S3 and S4 Figs.

Further, using GSV we performed a systematic search to determine the gene, gene ID, ERSE location on the chromosome, gene location and distance between ERSE elements and the gene. Genes, having ERSE element beyond 20 kb regions to their upstream ends and elements which have none genes (marked it as zero in the S2 Table) in their vicinity up to 100 kb upstream region are also listed (S2 Table). Although genes containing ERSE elements within promoter region are certainly ER stress responsive, we hypothesized that ERSE elements present up to 10 kb region upstream of the gene and the elements present inside the gene may probably regulate the gene expression under ER stress. Applying these criteria, the list of genes narrowed down to 134 genes containing ERSE-I (39 genes having ERSE element within 10 kb region of TSS and for 95 genes the elements were present within the gene); 106 genes containing ERSE-II (24 genes having ERSE element within 10 kb region of TSS and for 82 genes the elements were present within the gene) and 97 genes containing ERSE-III (23 genes having ERSE element within 10 kb region of TSS and for 74 genes the elements were present within the gene) S3 Table. Thus in total 86 genes had ERSE element within 10 kb region of TSS and 251 genes the elements were present within the gene (highlighted in gray) (Table 2). These preliminary *in silico* results suggest that ER stress may regulate a wide variety of genes unknown to be involved in the ER stress response.

**Table 2:** ER stress target genes having ERSE elements less than -10 kb upstream and present inside the gene less than +20 kb downstream from TSS. Genes having ERSE elements present inside the gene were marked in gray.

SNO	Gene	SNO	Gene	SNO	Gene	SNO	Gene
ERSE-I							
1	B3GALT6	35	PCDHGA1	69	DDX50	103	GSE1
2	TPRG1L	36	PCDHG@	70	C10orf11	104	ADORA2B
3	DARS2	37	PCDHGA2	71	CCDC147	105	NMT1
4	NOS1AP	38	PCDHGB1	72	INPP5A	106	CCDC40
5	DNM3	39	PCDHGA3	73	ART1	107	DTNA
6	TDRD5	40	PCDHGA4	74	TRIM22	108	KATNAL2
7	IGFN1	41	PCDHGB2	75	TSPAN18	109	KATNAL2
8	DISP1	42	CANX	76	ATG13	110	KATNAL2
9	SLC35F3	43	BTNL9	77	PPP6R3	111	CTIF
10	LPIN1	44	PPP1R3G	78	PPFIA1	112	CELF5 (Partial stop)
11	NCOA1	45	PGBD1	79	FAT3	113	KDM4B
12	KCNK3	46	DNAH8	80	FOXR1	114	CYP4F12
13	SLC9A2	47	GPR111	81	POU2F3	115	UBA2
14	STEAP3	48	FAM135A	82	CACNA1C	116	CEACAM3
15	MYO7B	49	ESR1	83	COPS7A	117	ZNF580
16	MZT2B	50	SDK1	84	BICD1	118	NLRP4
17	BOK	51	RPS14P10 (pseudo)	85	GLIPR1L1	119	ZNF17
18	RPL23AP39	52	CDK14	86	HSP90B1	120	PLCB4
19	CNTN4	53	FOXP2	87	MIR3652	121	SLC5A3
20	GRM7	54	MET	88	FICD	122	MRPS6
21	ENPP7P4 (pseudo)	55	KCND2	89	SMARCE1P5 (Pseudo)	123	DGCR5 (Partial stop)
22	LOC100128827 (pseudo)	56	CNTNAP2	90	TEX29	124	COMT
23	FRG2C	57	CHPF2	91	TFDP1	125	BCR
24	EIF2A	58	HSPD1P3 Pseudo	92	MIA2	126	IGL
25	CLRN1-AS1 (pseudo)	59	CSPP1	93	LIN52	127	FUNDC2P4 (Pseudo)
26	KCNMB2	60	SLC30A8	94	GPATCH2L	128	GLRA2
27	SORCS2	61	LRRC14	95	GABRG3	129	KDM6A
28	SPATA18	62	PIP5K1B	96	CHRNA7 (Partial stop)	130	ARAF
29	LPHN3	63	PCSK5	97	TCF12	131	TIMP1
30	MOB1B	64	TLE4	98	FOXB1	132	EDA
31	TRIM2	65	NTRK2	99	C15orf26	133	PAK3
32	ZSWIM6	66	SUSD3	100	SV2B	134	STAG2
33	CKMT2	67	C10orf68	101	CCNF		
34	CTNNA1	68	WDFY4	102	CDIPT-AS1 (Partial stop)		
**ERSE-II**							
1	PAPPA2	28	ARIH2	55	ENDOD1	82	WIPF2
2	ATP1A2	29	BANK1	56	RBM4	83	DNAH9
3	CAMTA1 (Partial stop)	30	TBC1D1	57	SLC22A18	84	DHX40
4	RABGAP1L	31	C4orf22	58	UBASH3B	85	ZNF473
5	SGIP1	32	TENM2	59	TRIM22	86	RUVBL2
6	HTR6	33	ZNF454	60	VPS11	87	QPCTL
7	FHAD1	34	RASGRF2	61	VPS11	88	PIN1 (Partial stop)
8	KIF26B	35	SCGB3A2	62	HS6ST3	89	NFIC
9	PAPPA2	36	RNF146 (Partial stop)	63	COL4A2	90	RPS19
10	PDE4B	37	C6orf201	64	CCNK	91	CC2D1A
11	ST3GAL3 (Partial stop)	38	LY86	65	NRXN3	92	ZNF175
12	FHAD1	39	NRF1	66	FAM177A1	93	ARHGEF1
13	SRGAP2 (Partial stop)	40	HECW1	67	AHSA1	94	FAM83D
14	ANKRD36	41	POU6F2	68	CALM1	95	CDS2
15	ITGA4	42	LRGUK	69	NRXN3	96	CDH4
16	EPC2	43	GTF2I	70	FBXO34	97	CDH4
17	FAM117B	44	EN2	71	SNAP23	98	DNAJC5
18	ZNF804A	45	RDH10	72	IGF1R	99	CTNNBL1
19	GPD2	46	COL27A1	73	ASB7	100	GRAP2
20	LIMS1	47	KANK1	74	SV2B	101	CLCN4
21	DIS3L2 (Partial stop)	48	PCSK5	75	BANP	102	PCDH11X
22	PTCD3	49	RUSC2	76	WFDC1	103	SMS
23	ANTXR1	50	PALM2	77	HERPUD1	104	ZNF275
24	CCRL2	51	SLC18A2	78	RBFOX1	105	MAGEB10
25	EEFSEC	52	DYDC2 (Partial stop)	79	PLCG2	106	SURF6P1 (Pseudo)
26	LPP	53	ZNF365	80	SCNN1B		
27	HPS3	54	FDX1	81	(SEPT9)		
**ERSE-III**							
1	DFFB	26	PCDHB16	51	ANO6	76	FOXK2
2	KAZN	27	PCDHB9	52	TUBA1C	77	NDC80
3	PADI1	28	PPARD	53	NUP107	78	SOGA2
4	SZT2	29	EYA4	54	NAV3	79	MALT1
5	VPS25P1 (Pseudo)	30	ZNF713	55	SYT1	80	GADD45B
6	TUFT1	31	DPP6 (Partial start)	56	TCP11L2	81	MAST1
7	DNM3	32	DLGAP2	57	BTBD11	82	PLEKHG2
8	CENPF	33	TGS1	58	UBE3B	83	LIPE-AS1 (Partial stop)
9	LYPLAL1	34	VPS13B	59	MVK	84	ZNF221
10	LCLAT1	35	DMRTA1	60	TPCN1	85	SULT2B1
11	EIF5B	36	DAPK1	61	SRRM4	86	PRNP
12	GPR45	37	ZNF618	62	AKAP11	87	GZF1
13	RHBDD1	38	DEC1	63	CPB2-AS1	88	CTNNBL1
14	CRTAP	39	DBH	64	WDFY2	89	PHACTR3
15	RNF13	40	GAD2	65	RAD51B	90	CDH4
16	POLR2H	41	INPP5A	66	NRXN3	91	PCBP3
17	LPP	42	ATHL1	67	CTDSPL2	92	IGL
18	SORCS2	43	SMPD1	68	SRRM2	93	INPP5J
19	ADAD1	44	GTF2H1	69	RBFOX1	94	TBC1D22A
20	MGST2	45	GLYATL1	70	EEF2K	95	TBC1D22A
21	PTGER4	46	MS4A15 (Partial stop)	71	ITGAX	96	FRMPD4
22	ZNF474	47	FLRT1	72	LLGL1	97	ATG4A
23	LOC100505841	48	C11orf53	73	STXBP4		
24	PCDHB@	49	UBASH3B	74	PITPNC1		
25	PCDHB8	50	SSPN	75	RNF157-AS1		

### Promoter analysis of ASB7 and interaction with ER stress proteins

**Fig 2:**
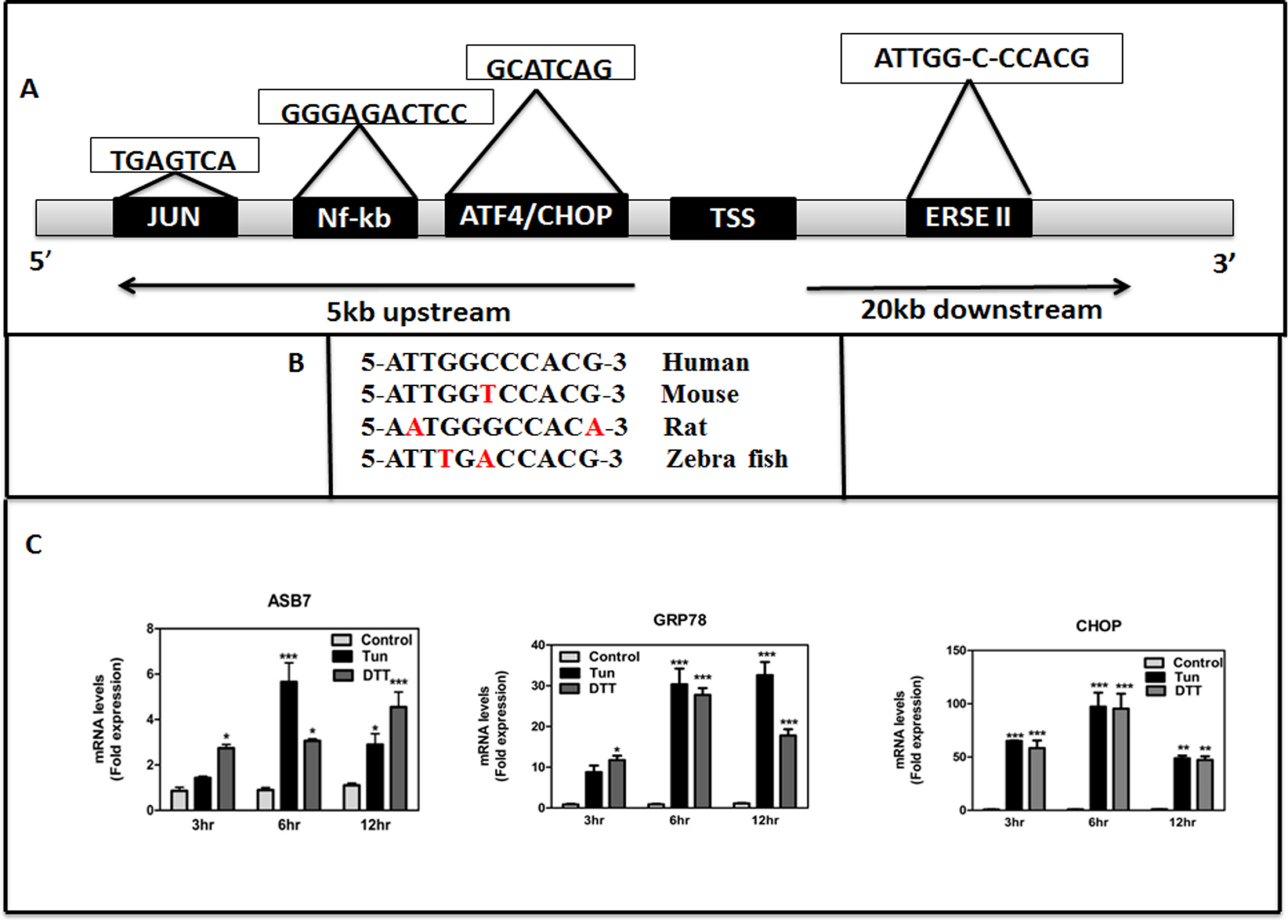
*In silico* promoter analysis and expression of ASB7 in ER stress. **(A)** Schematic representation of ASB7 gene promoter upstream (5000 bp) and downstream (20000 bp) of TSS with binding regions of ERSE-II element and transcription factor binding sequences of ATF4/CHOP, AP-1 and Nf- κB. **(B)** The mouse, rat and zebrafish sequence was selected as the reference species for evolutionary conservation. Numbers of mismatched nucleotides in the consensus sequence are marked in red; those conserved are marked in black. **(C)** HeLa cells were cultured with DMSO control (untreated cells) and concentrations of DTT (2 mM) and Tun (20 μg/ml). At 3,6 and 12 h after treatment, cells were lysed and levels of ASB7, GRP78 and CHOP mRNAs were measured by qRT-PCR and normalized relative to housekeeping gene, 18S rRNA (mean ± SEM; n = 2). “*” denotes significance with respect to control cells (DMSO treated). Error bars represent mean ± SEM. *** p≤0.001; **:p≤0.01; *: p≤0.05 calculated using two-way ANOVA with Bonferroni correction.

Among the ERSE containing genes identified by python programming, we were particularly interested in knowing the function of ASB7 that encodes an E3 ligase. Upregulation of E3 ubiquitin ligases is a vital mechanism through which ER stress improves degradation of misfolded ER proteins [44, 45]. Previously, ASB7 has been reported to play a crucial role in regulating spindle dynamics and genome integrity by controlling the expression of DDA3 [46]. However, the regulation of ASB7 gene in ER stress is not yet explored. Here our computational search revealed that ASB7 ERSE-II motif was in the intronic region. Looking for conservation across species is one of the best strategies for finding functional sequences in comparative genomics. Hence, we further examined whether the ERSE-II sequence is evolutionarily conserved by comparing it in the mouse, rat and zebrafish genome. As shown in Fig 1B, ERSE-II motif of mouse, rat and zebrafish ASB7 (located within the gene) were mostly homologous to that of the human ERSE motif with only 1 in mouse and 2 mismatches in rat and zebrafish, supporting the notion that this sequence is well conserved and may be functionally relevant. Additionally, we scanned the promoter region of human ASB7 for binding of transcription factors using FIMO (MEME suite) through their consensus sequences and located putative ATF/CREB binding sequence at TGAGTCA-3530 bp, NF-κB binding domain at GGGAGACTCC-2422 bp and ATF4/CHOP domain at GCATCAG-1468 bp from the TSS (Fig 2B).{Consensus sequence for ATF/CREB- TGA(C/G)TCA [47], ATF/CHOP-(GCATCAT/G) [48], NF-κB binding domain (GGGRNNYYCC)—R = purine; Y = pyrimidine [49]}.

The function and activity of a protein are generally modulated by other proteins with which it interacts. Hence next, we wanted to investigate protein-protein interactions of ASB7 and its family members to determine whether they can interact with any of the ER stress proteins (eg. CHOP, GRP78 etc) using cytoscape open source software platform. Interestingly, from the interacting protein-protein databases we found only ASB7 in the whole ASB family to have a direct protein-protein interaction with ATF4 an ER stress transcription factor and JUN proto-oncogene (AP-1 transcription factor subunit) S4 Table. ATF4 and JUN belong to a family of DNA-binding proteins that includes the AP-1 family of transcription factors, cAMP-response element binding proteins (CREBs) and CREB-like proteins [50]. Altogether, aforementioned computational results suggest that ERSE-II present within ASB7 seem to be evolutionarily conserved which might be under the regulation of CHOP and NF-κB and ASB7 potentially interacts with transcription factors like JUN and ATF4.

### ER stress increases the expression of ASB7 at the transcriptional level

To further confirm whether ERSE-II containing ASB7 gene is expressed during ER stress, we experimentally investigated its expression using chemical ER stress inducers like dithiothreitol (DTT; reduces the disulfide bridges of proteins) and tunicamycin (Tun; N-glycosylation inhibitor) in HeLa cells, a well-characterized cell line where the three major UPR pathways are active and responsive to ER stress. The expression of ASB7 was significantly elevated by both Tun (5 fold) and DTT (4 fold). The increase in ASB7 mRNA levels following stimulation with ER stress agents was evident within 6 h for Tun and 12 h for DTT after treatment (Fig 2C). To ascertain whether the treatments resulted in ER stress we checked the expression of CHOP and GRP78 mRNA (Fig 2C). These data strongly suggest that ASB7 gene expression is up regulated by ER stress in HeLa cells.

### Effect of ASB7 knockdown on the expression of UPR markers and ER stress pathways

The experiments described above established that ASB7 mRNA is regulated in response to ER stress. Based on these findings, first, we examined the effect of ASB7 knockdown on upstream (GRP78) and downstream UPR signaling component (CHOP). We compared the signaling events in which ASB7 was knocked down by transfecting pooled ASB7 siRNAs to a nonspecific scramble siRNA (control) in HeLa cells. Compared to control siRNA, ASB7 knockdown reduced the expression of CHOP and GRP78 mRNA in HeLa cells treated with DTT (2 mM) and Tun (20 μg/ml). Additionally, CHOP and GRP78 protein levels were measured by western blot and GRP78 levels were dramatically reduced in cells treated with Tun (20 μg/ml) compared to CHOP which showed no significant changes.

**Fig 3:**
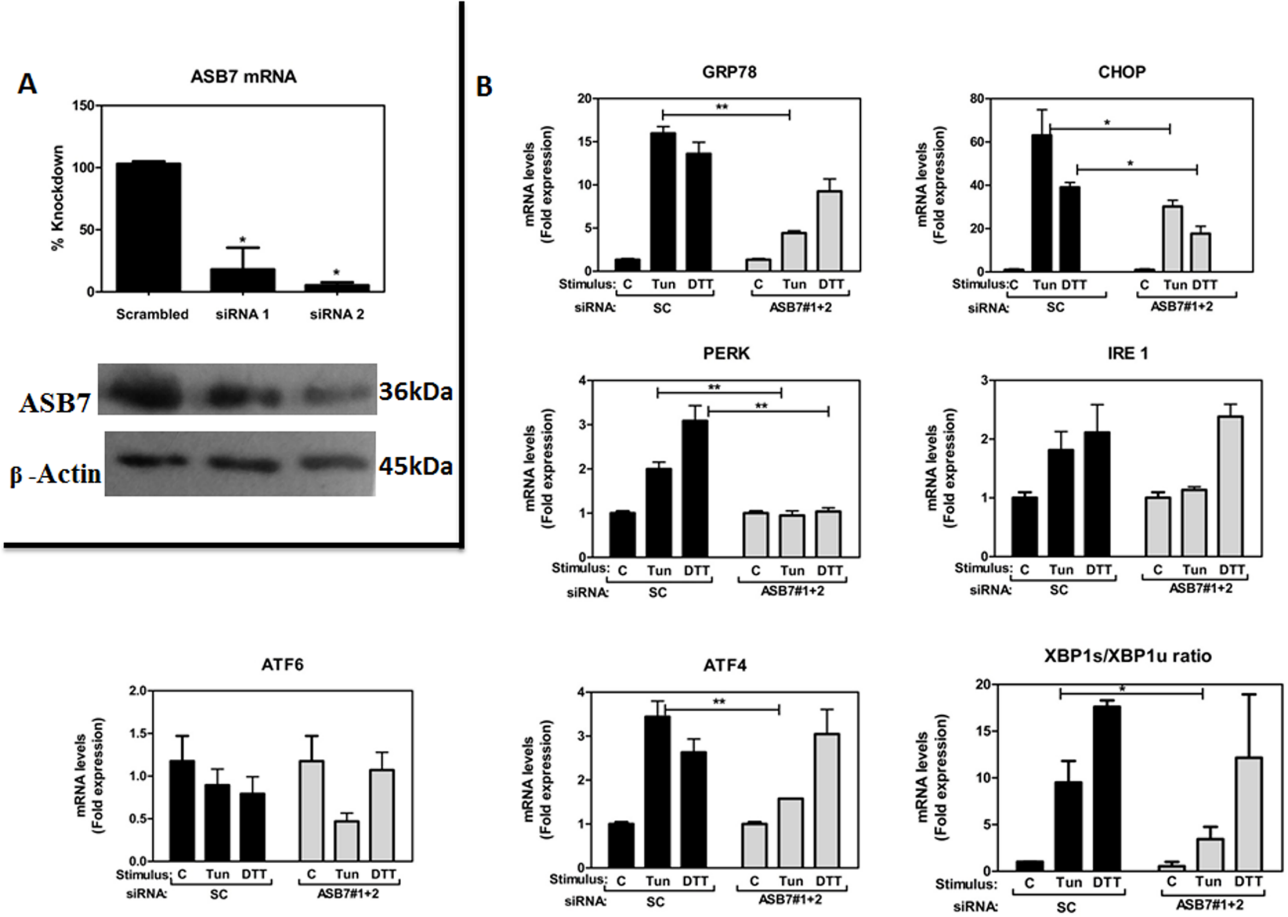
Effect of ASB7 knockdown on UPR markers and pathways under ER stress. **(A)** HeLa cells were transiently transfected with 2 independent ASB7 siRNA and a scrambled control (SC). After 2 days, levels of endogenous ASB7 mRNA and protein levels were measured by qRT‐PCR and immunoblotting and normalized relative to the housekeeping gene, 18S rRNA and β-actin (mean ± SEM, n = 2). **(B)** HeLa cells were transiently transfected pooling 2 independent ASB7 siRNAs (gray bars) and compared with scrambled siRNA as control (SC) (black bars). After 2 days, cells were cultured with DTT (2 mM) and Tun (20 μg/ml) for 3 h then total RNA was extracted. Relative levels of mRNAs encoding CHOP, GRP78, PERK, IRE-1α, ATF6 and spliced XBP-1 ratio were compared by qRT-PCR and displayed as ratios relative to a housekeeping gene (18S rRNA). “*” denotes significance with respect to scramble control. Error bars represent mean ± SEM. *** p≤0.001; **: p≤0.01; *: p≤0.05 calculated using one-way ANOVA.

**Fig 4:**
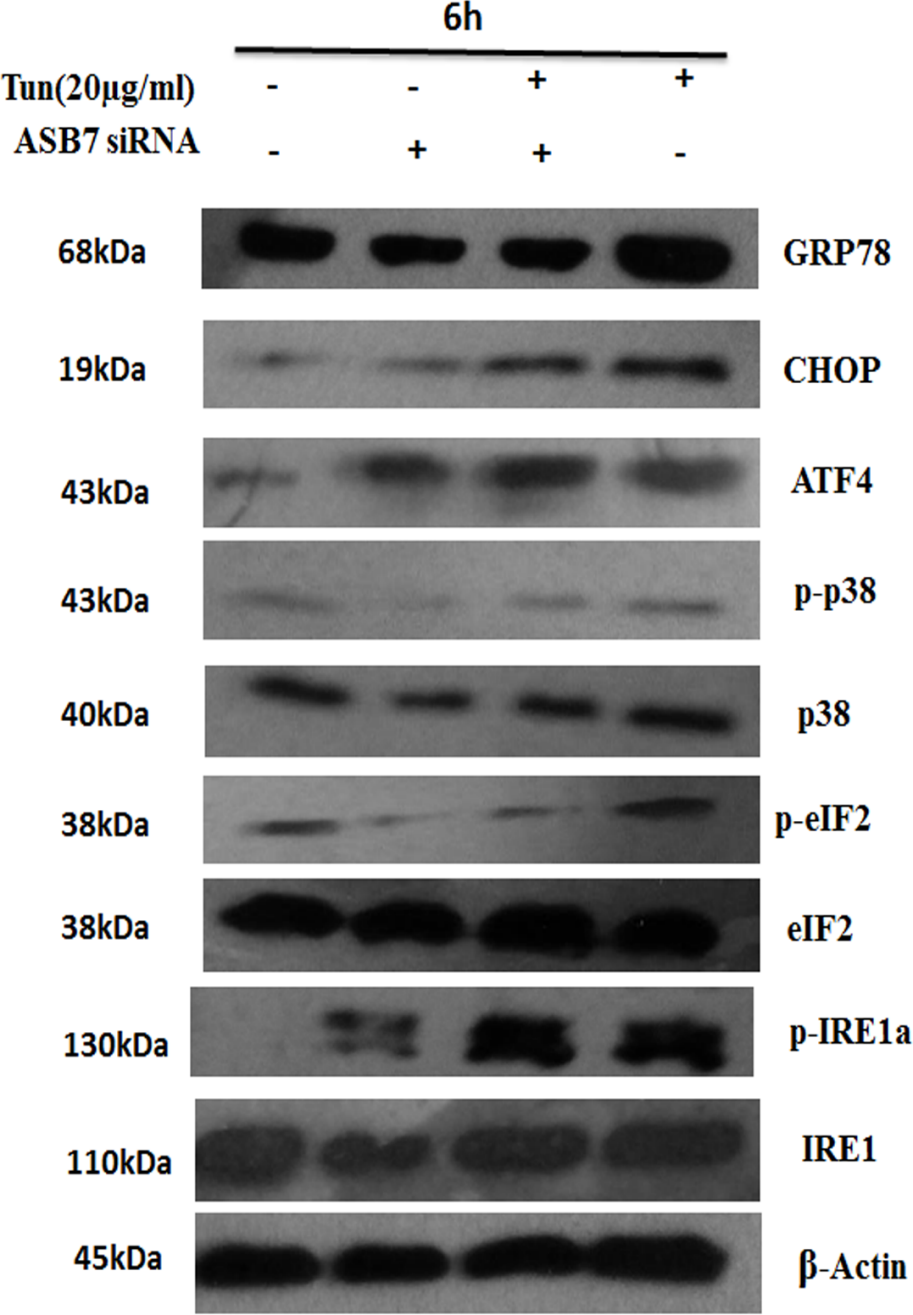
Effect of ASB7 knockdown on protein levels of UPR markers under ER stress. HeLa cells were transiently transfected pooling 2 independent ASB7 siRNAs, only ASB7 siRNA and compared with scrambled control. After 2 days, cells were cultured with Tun (20 μg/ml) for 6 h then total protein was extracted. Cell lysates were prepared, normalized for total protein and analyzed for immunoblotting for indicated proteins GRP78, CHOP, ATF4, IRE-1α, p-IRE-1α, p-eIF2α, eIF2α, p-p38 and p38 and normalized relative to the housekeeping gene β-Actin. Data representative of 2 experiments.

Further to investigate the impact of ASB7 knockdown on ER stress pathway, we studied the expression level of PERK, IRE-1α or ATF6, in HeLa cells upon ER stress induction (Fig 3B). These three ER stress response branches control the expression of specific transcription factors and signaling events that modulate a variety of downstream ER stress responses, which orchestrate adaptation to ER stress. Interestingly; we found reduced PERK mRNA levels compared to IRE-1α and ATF6 mRNA levels which were not much altered with respective scramble control. Indeed, ASB7 knockdown led to a decrease in Tun induced phosphorylated eIF2α levels; at 6 h post induction (Fig 4). ATF4 which gets translated under stress conditions via PERK pathway was found to show a significant reduction in their mRNA levels but was found to be expressed more at protein levels even in only ASB7 siRNA control condition also indicating its interaction with ASB7. ATF4 is one of the several key UPR transcriptional activators which have interaction with ASB7, suggesting it may play an important role in ASB7 regulation. Next, we checked IRE-1α phosphorylation protein levels which were not much altered on ASB7 knockdown. Further, the efficiency of XBP-1 splicing in these cells was also examined. The spliced XBP-1 ratio mRNA also showed decreased levels in ASB7 knockdown cells. P38 MAPK is a key kinase activated by many cellular stress conditions including ER stress. ASB7 knockdown cells significantly displayed reduced p-p38MAPK levels (Fig 4). Taken together these findings suggest that the phosphorylation of eIF2α and p38, ATF4 and GRP78 expression is mostly influenced by the ASB7 knockdown in ER stress. The reduction in the mRNA levels of PERK, ATF4 and sXBP-1 ratio and phosphorylation of eIF2α protein level may be due to a partial shutdown of the PERK-ATF4-CHOP pathway. Overall, these results establish ASB7 might act as a mediator of ER stress signaling *in vitro* via PERK/ATF4/CHOP pathway and suggest a possible role for ASB7 downstream of PERK signaling pathway. qRT-PCR and western blot analysis confirmed that ASB7 protein and mRNA expression level was decreased by ASB7 siRNAs compared to control siRNA (Fig 3A).

### ASB7 plays an important role in the PERK/ATF4/CHOP pathway in ER stress

**Fig 5:**
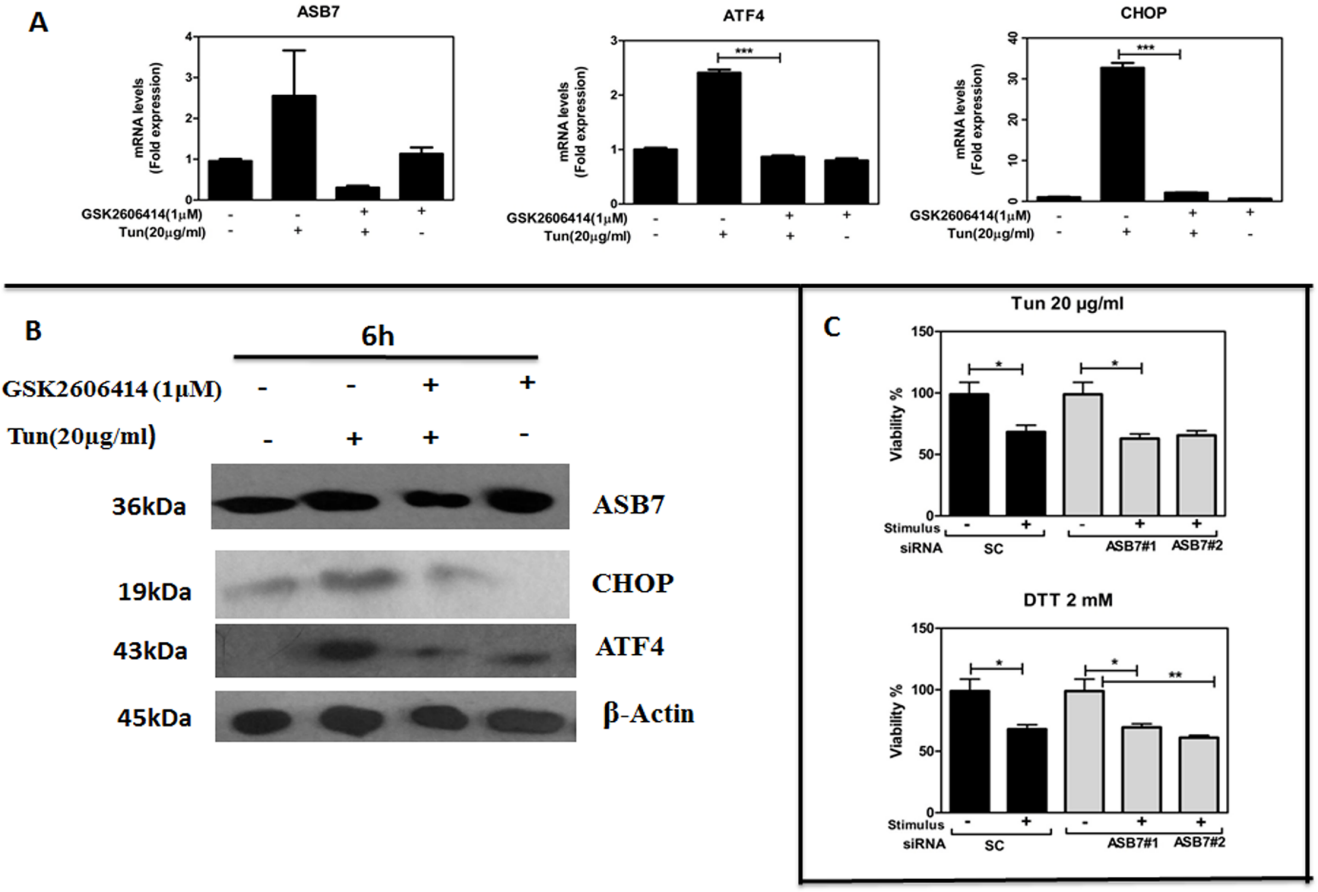
ASB7 may act downstream of PERK pathway and knockdown of ASB7 did not influence ER stress-induced cell death in HeLa cells. (A) and (B) HeLa cells were treated with Tun (20 μg/ml, 6 h) in the absence or presence of PERK kinase inhibitor GSK2606414 (1 μM) with DMSO as the control. Total RNA and cell lysates were prepared, normalized for total protein and mRNA expression and immunoblotting of indicated proteins ASB7, CHOP and ATF4 for 6 h in HeLa cells and displayed as rations of the housekeeping gene (18s rRNA and β-actin). Data representative of 2 experiments. (C) HeLa cells were transfected with two different siRNAs targeting ASB7 and with scrambled (SC) siRNA control. After 2 days of transfection, cells were cultured with DTT (2 mM) and Tun (20 μg/ml) for 24 h. Cell viability was estimated by measuring MTT assay, expressing data as % of control untreated DMSO cells (mean ± SEM, n = 2). “ * ” denotes significance with respect to scramble and DMSO control. Error bars represent mean ± SEM. *** p≤0.001; **: p≤0.01; *: p≤0.05 calculated using one-way ANOVA.

Since PERK mRNA expression and eIf2α phosphorylation was decreased on ASB7 knockdown compared to IRE-1α and ATF6 mRNA levels, we tried to investigate whether PERK pathway had its roles in the ASB7 induction. HeLa cells were treated with the ATP-competitive PERK kinase inhibitor GSK2606414 (1 μM), followed by treatment with Tun for 6 h. PERK inhibition reduced ATF4 and CHOP mRNA and protein levels, confirming PERK inhibition, and slightly attenuated ASB7 mRNA and protein expression, indicating PERK pathway can play an essential for ER stress-induced ASB7 activation (Fig 5A and 5B). Moreover, we also measured the mRNA expression levels of ATF4/CHOP signaling targeted genes, such as TRB3 and DR5 in ASB7 knockdown cells which displayed a slight but non-significant decrease in the expression of TRB3 and DR5 suggesting defective PERK/ATF4/CHOP signaling (S5 Fig). Taken together these findings suggest that, the downstream targets of PERK/ATF4/CHOP pathway are influenced by the ASB7 knockdown in ER stress. Overall, these results establish ASB7 might act as a mediator of ER stress signaling *in vitro* via PERK/ATF4/CHOP pathway and suggest a possible role for ASB7 downstream of PERK signaling pathway.

### ASB7 knockdown does not influence ER stress-induced cell death in HeLa cells

As ASB7 was up regulated in ER stress and ASB7 knockdown reduced UPR markers we investigated the ability of ASB7 to protect cells from ER stress-induced cell death in HeLa cells, we transfected HeLa cells with two different ASB7 siRNAs and a non-specific scramble siRNA (control) exposed to the ER stressors Tun (20 μg/ml) and DTT (2 mM) 48 h post transfection. MTT assay was performed after 24 h to confirm the effect of ASB7 knockdown on cell viability. We found ablation of ASB7 confers no protection in ER stress-induced cell death in HeLa cells (Fig 5C). These results suggested that ASB7 knockdown cells retain their sensitivity to ER stress-induced cytotoxicity despite the up-regulation of ASB7 in response to ER stress.

### ASB7 knockdown alters pro-inflammatory markers in ER Stress

**Fig 6:**
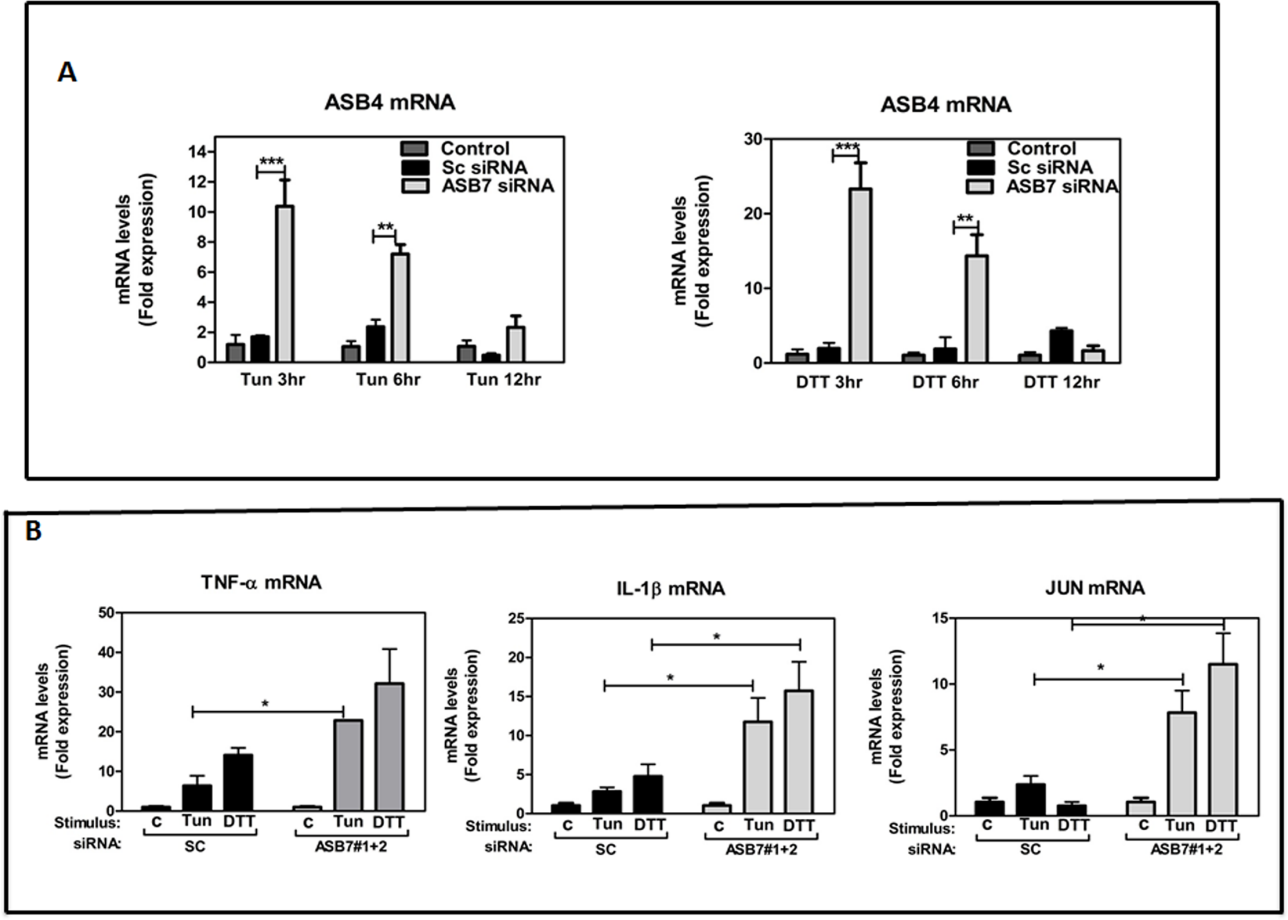
Effect of ASB*7* knockdown on pro-inflammatory markers in ER stress. **(A)** HeLa cells were transiently transfected pooling 2 independent ASB7 siRNA (gray bars) and compared with scrambled siRNA control (black bars) (SC). Cells were cultured with Tun (20 μg/ml) and DTT (2 mM) and total RNA was extracted. Levels of endogenous ASB4 mRNA levels were measured by qRT‐PCR for different time points 3, 6, 12 h and normalized relative to housekeeping gene, 18S rRNA (mean ± SEM, n = 2). “_*_” denotes significance with respect to scramble control. Error bars represent mean ± SEM. ***: p≤0.001; **: p≤0.01; *: p≤0.05 calculated using two-way ANOVA with Bonferroni correction. (**B**) HeLa cells were transiently transfected pooling 2 independent ASB7 siRNA (gray bars) and compared with scrambled siRNA control (black bars) (SC). After 2 days, total RNA was extracted for 6 h. Relative levels of mRNAs encoding TNF-α (left), IL-1β (center), and JUN (right) were compared by qRT-PCR and displayed as ratios relative to a housekeeping gene (18S rRNA). Data are mean ± SEM, n = 2. “_*_” denotes significance with respect to scramble control. Error bars represent mean ± SEM. *** p≤0.001; **: p≤0.01; *: p≤0.05 calculated using one-way ANOVA with Bonferroni correction.

Recent research reveals connections between UPR and inflammation at multiple levels [51]. Finally, we wanted to investigate the effect of ASB7 knockdown on the regulation of ER stress-induced inflammation and the expression of proinflammatory markers. At first, we found that knockdown of ASB7 showed significant upregulation of ASB4 mRNA with respective scramble control (Fig 6A). Recent studies, identified dimers of ASB7 and ASB4, but no evidence of target-induced dimerization are reported, as the putative interactions identified do not contain overlapping proteins [52]. Previously, ASB4 has also been reported to act downstream of NF-κB in the TNF-α signaling cascade which can aid in the potential regulation of TNF-α’s numerous functions linked with inflammation, angiogenesis, and apoptosis [53]. We were particularly interested in understanding whether ASB4 upregulation influences the pro-inflammatory cytokines expression such as TNF-α, IL-1β, and IL-6 in ER stress. As shown in Fig 6B; TNF-αand IL-1β mRNA levels were strongly upregulated in the ASB7 knockdown cells, compared to the scramble control. Additionally, we checked JUN proto-oncogene AP-1 transcription factor subunit which was also found to be upregulated in ASB7 knockdown cells. Current studies indicate that there may be interactions between the AP-1 activating pathways and NF-κB pathways, in which TNF receptor associated factor (TRAF-2) (activated by TNF-α) and TRAF-6 (activated by IL-1β) may both activate NF-κB inducing kinase (NIK) and subsequently IκB kinases (IKK) [54]. Collectively, above result suggests that knockdown of ASB7 and upregulation of ASB4 in HeLa cells can be associated with alterations of immune/inflammatory players, such as pro-inflammatory cytokines, and JUN under ER stress conditions showing its signaling link between AP-1 and NF-κB signaling pathway.

## Discussion

ER stress is implicated in numerous diseases [55–57]. In spite of our current understanding of ER stress and related pathways mechanisms underlying cell decision to undergo adaptation versus death programs has been poorly understood. In this study, we performed a genome-wide search using computational programming approach to predict ER stress target genes regulated under ERSEs (I, II, II). Our results show that putative ERSE are present, on average, on every chromosome in the human genome, although it is likely that most of the genes found may or not be regulated under ER stress, at least by transactivation. In this regard, we screened genes having ERSE in the 10 kb region upstream of the promoter and inside the gene, out of which we found 337 candidate genes might be ER stress targeted genes. Most of these genes had not been identified previously as ERSE containing or ER stress-responsive genes. Nevertheless, the data obtained in the present study should be useful not only for identifying ER stress target genes but also for identifying transcriptional factors that act cooperatively and regulate their expression. Among the found candidate genes, we were interested in ASB7 gene, which belongs to ASB family, composed of 18 members. At present, knowledge on the function of the ASB members as a substrate-recognition component of the ECS complex is limited. Most of these proteins have interaction with Cul5 and Rbx2 which act as components of ubiquitin ligase complexes [58]. Ubiquitin ligases can regulate factors that improve the magnitude and duration of the UPR and thus impact overall cellular stress outcome.

In our findings, we found ERSE-II located within ASB7 in the intronic region. Previous studies have shown intron sequences to be involved in transcriptional regulation of many other genes. First known intronic enhancement of gene expression was recognized in Human alpha-1 collagen gene [59] and was further found in other genes including the *CD21* [60], *ALAS2* [61], *delta1-crystallin* [62], *CRP1* [63] and *Opalin* gene. Mostly, intronic control of gene transcription usually involves the binding of transcription factors to the DNA sequence [64, 65]. Additionally, the sequence surrounding the ERSE in the ASB7 seems to be conserved across species. Other species have limited number of ASB proteins; in spite of this, they show very high conservation with human ASBs. Moreover, we identified several putative cis-acting elements for ATF4/CHOP, NF-κB, and AP-1, within 5 kb upstream of ASB7. Moreover, all UPR branches can be modulated at the level of ER sensors and transcription factors, through the binding of co-factors and post-translational modifications. It is thus possible that this consensus sequences close to promoter may play a role in recruiting this transcriptional co-activators to activate ASB7 gene expression.

Gene expression in response to numerous stimuli is regulated by conserved cis-elements found in the promoter region of the gene. Most UPR-target genes, such as *GRP78*, *GRP94*, and *calreticulin*, are fully activated by multiple copies of ERSE (CCAAT-N9-CCACG/A). We found that expression of the ASB7 gene is directly up-regulated by ER stress, which confirmed an association between ERSE and ASB7 and helps to elucidate one aspect of the biological significance of elevated ASB7 expression. During ER stress GRP78 dissociate from PERK, ATF6 and IRE-1α, and leads to downstream UPR signaling [10]. The regulatory interplay between ER stress and other pathway operates at multiple levels, suggesting that fine-tuning the balance is of importance to cellular physiology and metabolism [66]. In this study, we suggest ASB7 might collaborate with signaling enzymes or transcription factors such as ATF4 and CHOP that can regulate the PERK pathway. With regards to ER stress, ASB7 potentially could operate at several levels to impact PERK signaling. Two aspects of our findings in this report suggest that ASB7 may play a major role in PERK signaling. First, we found ASB7 has interaction with ATF4 and regulates ATF4 protein and PERK mRNA levels, GRP78 and CHOP levels, eIF2α phosphorylation and decreases induction of PERK/ATF4/CHOP target genes like TRB3 and DR5 mRNA under ER stress. Second, we found inhibition of PERK pathway using PERK inhibitor slightly reduced the levels of ASB7 protein levels in Tun treated cells. However, it could not be elucidated whether ASB7 promoted ATF4 expression directly or indirectly in this study. Comprehending detailed mechanism of ATF4 regulation by ASB7 will help us elucidate its role in ER stress. However, knockdown of ASB7 in HeLa cells did not influence cell survival in ER stress-induced cell death. Although these observations could suggest ASB7 is not required for ER stress induced cell death in HeLa cells as CHOP protein levels were not much regulated in ASB7 knockdown. All three UPR signaling events finally converge into the activation of CHOP a pro-apoptotic transcription factor that induces apoptosis by modulating the expression of several genes. The other reason may be only the inhibition of ASB7 gene alone would likely not be sufficient to protect cells during ER stress in HeLa cells.

Experimental evidence has demonstrated that synthesis of many pro-inflammatory cytokines takes place during ER stress-induced UPR signaling. It has been shown that activation of UPR itself induces inflammation through c-Jun N-terminal kinase (JNK)-activator protein-1 (AP-1) [67] and inhibitor of κB (IκB) kinase (IKK)-nuclear factor-κ B (NF-κB) pathways [68]. ASB7 can play a pro-inflammatory role comes from our following observation: first, ASB7 knockdown upregulated pro-inflammatory genes as TNF-α, IL-1β and protoncogene JUN. Studies have revealed AP-1 as a transcription factor can activate an inflammatory gene program in ER stress [69]. Also the co-activation of NF-κB and AP-1 can synergistically and effectively enhance the transcription of genes in response to a variety of stimuli [70]. Second, we also found an NF-κB binding site in the promoter region of ASB7. Binding of NF-κB to the promoter region of ASB7 can affect transcriptional regulation of several cellular and viral genes expressed in ER stress. These observations could suggest a potential role for ASB7 in inflammatory processes; although no known association of ASB7 in inflammatory response has been reported earlier. These data propose a link between ER stress and inflammation. The mechanism behind the action of ASB7 and ASB4 under ER stress on pro-inflammation is still unclear. Dimers of ASB4/ASB7 has been described, but no evidence of target-induced dimerization, as the putative interactions identified do not contain overlapping proteins, suggesting that if they bind to the same targets they will do it with different affinities. However, if similar regulation of ASB7 is demonstrated in other cell lines, it could suggest that ASB7 is potentially instrumental in ER stress-induced inflammation and UPR signaling.

In summary, we have identified 337 ERSE containing genes by *in silico* computational approach using python programming. Our results indicate that *in silico* approach is a commanding method for identification of ERSE containing genes and could serve to facilitate analysis of the function of them in UPR signaling. In particular, we have shed light on the regulation of ASB7 *in vitro* and highlight the complexity of the regulatory pathways involved in modulating the effects of this protein in ER stress. The involvement of ASB7 in ER stress signaling is particularly exciting as the data suggests a potential role for ASB7 in UPR and inflammatory responses which are involved in numerous disease conditions. ASB7 being an E3 ligase, it is also critical to determine the experimental substrate, cellular localization of ASB7 under ER stress. Further studies are required to determine the tissue specific functions of ASB7 in different cell lines and detailed mechanisms of how ASB7 contributes to ER stress. Our study identifies genes previously unsuspected to be involved in ER stress; however their experimental validation is demanded. Although ASB7 did not regulate ER stress induced cell death, it decreased UPR signaling events indicating its involvement in other cellular signaling pathways. We believe that our finding contributes to the existing knowledge on ER stress target genes and may open new therapeutics perspectives by providing a suitable starting point for further study of UPR target genes.

## Supporting information

S1 Table ERSE hits found by python programming in the human genome with their location on the chromosome. (DOCX)

S2 Table Detailed information about the gene, gene name, ERSE element location, gene location and distance between the ERSE element and gene. (DOCX)

S3 Table The extracted corresponding closest gene present within 10 kb region and also extracted the gene which is having ERSE element inside the gene. (DOC)

S4 Table Gene/gene and protein-protein interactions of ASB gene family using Cytoscape. (DOCX)

S1 Fig Schematic representation of SV viewer. (TIF)

S2 Fig Schematic representation of location of ERSE elements of GRP94. (TIF)

S3 Fig Schematic representation of location of ERSE elements of HERP. (TIF)

S4 Fig Schematic representation of location of ERSE elements of PRNP gene. (TIF)

S5 Fig ASB7 knockdown affects ATF4/CHOP downstream genes TRB3 and DR5. (TIF)

## Abbreviations

ER: Endoplasmic reticulum
ERAD: Endoplasmic reticulum associated protein degradation
ERSE: Endoplasmic reticulum stress element
UPR: Unfolded protein response
ASB7: Ankyrin repeat SOCS box protein 7
TNF-α: Tumor necrosis factor alpha
TNFR: Tumor necrosis factor receptor
IL-1β: Interleukin 1 beta
CHOP: C/EBP Homologous Protein
GRP78: Glucose-regulated protein 78
XBP1: X-Box binding protein 1
ATF4: Activating transcription factor 4
FKBP13: FK506 Binding Protein 1A
ATF3: Activating transcription factor 3
RTP/NDRG: Receptor transporter protein/ N-Myc Downstream Regulated
HERP: Homocysteine-responsive endoplasmic reticulum-resident ubiquitin-like domain member protein
PRNP: Prion protein
SOCS: Suppressor of cytokine signaling
DDA3: Differential Display and Activated By P53
TRB3: Tribbles Pseudo kinase 3
DR5: Death receptor 5
Cul5: Cullin 5 protein
Rbx2: Ring box protein 2
CD21: Complement C3d Receptor 2
ALAS2: Aminolevulinate, Delta Synthase 2
CRP1: Cruciform DNA-Recognizing Protein
AP-1: Activator protein 1
TSS: Transcription start site
TRAF2: TNF receptor associated factor 2
NF-κB: Nuclear factor kappa-light-chain-enhancer of activated B cells
PMSF: Phenyl methane sulfonyl fluoride or phenylmethylsulfonyl fluoride
PVDF: Polyvinylidene difluoride
