# Supplementary material for: Identification of ASB7 as ER stress responsive gene through a genome wide in silico screening for genes with ERSE
Source: PLoS One. 2018 Apr 9;13(4):e0194310. doi: 10.1371/journal.pone.0194310 (PMC5890977; doi:10.1371/journal.pone.0194310)
Supplement: S1 Table — (DOCX) [file pone.0194310.s001.docx]

**Python program for identification of ERSE-I,II,III hits in the genome:**

#!/usr/bin/env python

import re

bible = open(&quot;sequence.fasta&quot;, &quot;r&quot;)

str = &quot;&quot;;

for line in bible:

str += line.rstrip()

pat = re.compile(r&#39;(CCAAT.........CCACG)&#39;)

res = [(m.group(), m.start()) for m in pat.finditer(str)]

count = 0

for temp in res:

print temp[0], &quot; found at &quot; , (temp[1]/72)+1 , &quot; line&quot;

count = count + 1

print &quot;Total Matches &quot; ,count

**S1 Table :**

535 ERSE-I (CCAAT-N_9_-CCACG) elements, 362 ERSE-II (ATTGG-N-CCACG) and 411 ERSE-III (CCAAT-N_26_-CCACG) hits were found by python programming in the human genome with their location on the chromosome.

| **ERSE-I** | | | |
| --- | --- | --- | --- |
| **SNO** | **Chromosome 1** | SNO |  |
| 1 | CCAATCCCCGGGCACCACG found at 16172 line | 272 | CCAATGATCCAGCACCACG found at 1663853 line |
| 2 | CCAATTGTAATCCCCCACG found at 49113 line | 273 | CCAATGGAGGATGTCCACG found at 1867593 line |
| 3 | CCAATTGTAATCCCCCACG found at 396193 line | 274 | CCAATTTAATCATGCCACG found at 1929372 line |
| 4 | CCAATATGGTGAAACCACG found at 56437 line |  | **chromosome 10** |
| 5 | CCAATATGGTGAAACCACG found at 356291 line | 275 | CCAATGTTTAGCTCCCACG found at 124154 line |
| 6 | CCAATATGGTGAAACCACG found at 2414064 line | 276 | CCAATACCAAATGCCCACG found at 138077 line |
| 7 | CCAATTTGCCTCATCCACG found at 64905 line | 277 | CCAATGCCTCGAGCCCACG found at 178790 line |
| 8 | CCAATATGGGGAAACCACG found at 72760 line | 278 | CCAATGATCACTTACCACG found at 458558 line |
| 9 | CCAATCGTGGTGGTCCACG found at 180521 line | 279 | CCAATTTTGCCTGCCCACG found at 693144 line |
| 10 | CCAATCGTGGTGGTCCACG found at 182054 line | 280 | CCAATATATAACCCCCACG found at 710207 line |
| 11 | CCAATATGCTTGTCCCACG found at 232552 line | 281 | CCAATATATAACCCCCACG found at 713286 line |
| 12 | CCAATCATGATGGCCCACG found at 265831 line | 282 | CCAATCATTCTGACCCACG found at 723776 line |
| 13 | CCAATATGAACCATCCACG found at 703891 line | 283 | CCAATTACAAAGAGCCACG found at 771279 line |
| 14 | CCAATGAAGTTTGTCCACG found at 937151 line | 284 | CCAATATGGTGAAACCACG found at 969744 line |
| 15 | CCAATCCCTTATTTCCACG found at 986878 line | 285 | CCAATATGGTGAAACCACG found at 1088118 line |
| 16 | CCAATCCCTTATTTCCACG found at 1134892 line | 286 | CCAATTCCCAAACTCCACG found at 981388 line |
| 17 | CCAATTTCAAACTTCCACG found at 1033004 line | 287 | CCAATCCGTGGGCCCCACG found at 1007760 line |
| 18 | CCAATCACCTTCTACCACG found at 1079206 line | 288 | CCAATTCTACTTAACCACG found at 1082994 line |
| 19 | CCAATTCATACTGGCCACG found at 1279476 line | 289 | CCAATCGCTGTCCCCCACG found at 1248054 line |
| 20 | CCAATTCTTTCGTTCCACG found at 1336222 line | 290 | CCAATCCCTTATTTCCACG found at 1278556 line |
| 21 | CCAATTGAAGTGGTCCACG found at 1536971 line | 291 | CCAATCCTGGTGCCCCACG found at 1348334 line |
| 22 | CCAATGCAGGCAGACCACG found at 1677117 line | 292 | CCAATCACTCCTCACCACG found at 1390682 line |
| 23 | CCAATACCCCAGAGCCACG found at 2026934 line | 293 | CCAATTTATACTACCCACG found at 1473624 line |
| 24 | CCAATACCCCAGAGCCACG found at 2048347 line | 294 | CCAATGGAGTCTGGCCACG found at 1818294 line |
| 25 | CCAATGAAATAAAGCCACG found at 2252455 line | 295 | CCAATCTGTGAGCCCCACG found at 1837747 line |
| 26 | CCAATCAGGCTCTGCCACG found at 2299551 line | 296 | CCAATGGTCTCCGGCCACG found at 1851396 line |
| 27 | CCAATTCTTTCTGGCCACG found at 2392948 line | 297 | CCAATCCCTAAACCCCACG found at 1867829 line |
| 28 | CCAATATCCTAAAGCCACG found at 2438948 line | 298 | CCAATTCTCACCGGCCACG found at 1872818 line |
| 29 | CCAATCTCCTAAAACCACG found at 2456123 line | 299 | CCAATGAGCTGCCCCCACG found at 1876117 line |
| 30 | CCAATTAGAGACATCCACG found at 2494728 line |  | **chromosome 11** |
| 31 | CCAATATGCTGAAACCACG found at 2699696 line | 300 | CCAATACTGACATTCCACG found at 24879 line |
| 32 | CCAATGCACACCACCCACG found at 2793800 line | 301 | CCAATGCCTGGGGTCCACG found at 44061 line |
| 33 | CCAATCACCTCCCACCACG found at 3098804 line | 302 | CCAATCCCTTATTTCCACG found at 50977 line |
| 34 | CCAATGTGAAAAGGCCACG found at 3114385 line | 303 | CCAATGGCATTTCTCCACG found at 79256 line |
| 35 | CCAATGGCCAGGCACCACG found at 3140051 line | 304 | CCAATCACCTCAGACCACG found at 182798 line |
| 36 | CCAATCTCCTGGTGCCACG found at 3150193 line | 305 | CCAATAGAAGAGTGCCACG found at 314193 line |
| 37 | CCAATACTCAGTTCCCACG found at 3201884 line | 306 | CCAATTTGGGTGTTCCACG found at 468268 line |
| 38 | CCAATGCACACTGGCCACG found at 3255058 line | 307 | CCAATATTTTATATCCACG found at 617724 line |
| 39 | CCAATGAGGCGGGACCACG found at 3283967 line | 308 | CCAATCAGGGACCTCCACG found at 622844 line |
| 40 | CCAATTAACCAAAGCCACG found at 3305944 line | 309 | CCAATGTCCGGTGACCACG found at 647748 line |
| 41 | CCAATCAGCATTCTCCACG found at 3309770 line | 310 | CCAATCATCTTGTACCACG found at 813428 line |
| 42 | CCAATAGGTAGGGACCACG found at 3371703 line | 311 | CCAATATCCCTGGTCCACG found at 892433 line |
| 43 | CCAATCATGTCTCACCACG found at 3374753 line | 312 | CCAATGGGCCCGTGCCACG found at 897554 line |
| 44 | CCAATAAAAAAGTACCACG found at 3431003 line | 313 | CCAATGGAAACAACCCACG found at 949555 line |
|  | **Chromosome 2** | 314 | CCAATGCGTTTATCCCACG found at 951744 line |
| 45 | CCAATACAAACATCCCACG found at 23996 line | 315 | CCAATCGCCTCCCACCACG found at 960553 line |
| 46 | CCAATGCTGTCCGCCCACG found at 31324 line | 316 | CCAATTCTTTATCTCCACG found at 973962 line |
| 47 | CCAATCCCTAGGACCCACG found at 34017 line | 317 | CCAATCCACCTCTGCCACG found at 1007084 line |
| 48 | CCAATTTCTTCCTTCCACG found at 39280 line | 318 | CCAATTCACTGAGACCACG found at 1079940 line |
| 49 | CCAATATGGTAAAACCACG found at 73878 line | 319 | CCAATAAGTTTTCTCCACG found at 1282269 line |
| 50 | CCAATAGACTTGTTCCACG found at 90898 line | 320 | CCAATTCTAGGTTCCCACG found at 1306724 line |
| 51 | CCAATCACCTCCCACCACG found at 119777 line | 321 | CCAATACAGTGGCCCCACG found at 1646335 line |
| 52 | CCAATTTGGACAAGCCACG found at 165492 line | 322 | CCAATATGGTGAAACCACG found at 1650679 line |
| 53 | CCAATCCCTTATTTCCACG found at 177330 line | 323 | CCAATAAATGCAAACCACG found at 1668738 line |
| 54 | CCAATCCCTTATTTCCACG found at 938333 line | 324 | CCAATGTCCTTGAGCCACG found at 1732948 line |
| 55 | CCAATCCCTTATTTCCACG found at 1073842 line | 325 | CCAATCTCCCTCTCCCACG found at 1760607 line |
| 56 | CCAATCCCTTATTTCCACG found at 3127259 line | 326 | CCAATAAAGCTCTTCCACG found at 1840871 line |
| 57 | CCAATTAAAGGACACCACG found at 244929 line |  | **chromosome 12** |
| 58 | CCAATGTCATGCTTCCACG found at 248544 line | 327 | CCAATGAGAAGGGGCCACG found at 36465 line |
| 59 | CCAATGTGGTGAAACCACG found at 344343 line | 328 | CCAATGGATGATCCCCACG found at 85503 line |
| 60 | CCAATAAATGCTATCCACG found at 374363 line | 329 | CCAATATGGTAAAACCACG found at 94836 line |
| 61 | CCAATGGGAGACAACCACG found at 491805 line | 330 | CCAATGTCAACTTACCACG found at 160097 line |
| 62 | CCAATTGTACATGACCACG found at 746785 line | 331 | CCAATCTGAGACACCCACG found at 176588 line |
| 63 | CCAATCGCTTATTTCCACG found at 895568 line | 332 | CCAATAAACAAAAACCACG found at 176677 line |
| 64 | CCAATCTCTCCTGCCCACG found at 912704 line | 333 | CCAATTTTCATTTTCCACG found at 246464 line |
| 65 | CCAATAATGTGATACCACG found at 989408 line | 334 | CCAATTCTTCTTTTCCACG found at 375894 line |
| 66 | CCAATCAGGGTTGCCCACG found at 1434480 line | 335 | CCAATGGGCTCTCACCACG found at 448324 line |
| 67 | CCAATTTTCACACACCACG found at 1645507 line | 336 | CCAATGTGTAACCTCCACG found at 547672 line |
| 68 | CCAATGCTCACAGCCCACG found at 1664456 line | 337 | CCAATTGTCTCTTACCACG found at 931256 line |
| 69 | CCAATCACACTGGGCCACG found at 1666378 line | 338 | CCAATGACCACCCCCCACG found at 976129 line |
| 70 | CCAATGCAGAAAAGCCACG found at 1710564 line | 339 | CCAATCCCTTATTTCCACG found at 1015254 line |
| 71 | CCAATTCTCTCCATCCACG found at 1782842 line | 340 | CCAATCCCTTATTTCCACG found at 1212967 line |
| 72 | CCAATCATCACCCACCACG found at 1818698 line | 341 | CCAATCCCTTATTTCCACG found at 1335333 line |
| 73 | CCAATCCAAGGAAGCCACG found at 1938262 line | 342 | CCAATGAGTCATTTCCACG found at 1052231 line |
| 74 | CCAATGATGTGCACCCACG found at 2218461 line | 343 | CCAATCTCCCAATTCCACG found at 1388961 line |
| 75 | CCAATACCCGGCAGCCACG found at 2346017 line | 344 | CCAATCGGAAGGAGCCACG found at 1448946 line |
| 76 | CCAATATGGTGAAACCACG found at 2425248 line | 345 | CCAATGGGAGTGGCCCACG found at 1512625 line |
| 77 | CCAATCTCCAACTCCCACG found at 2528141 line | 346 | CCAATTAGCAGGTACCACG found at 1513764 line |
| 78 | CCAATTGAGGAGTGCCACG found at 2665202 line | 347 | CCAATGAGATGAAGCCACG found at 1623563 line |
| 79 | CCAATCATTGTGAACCACG found at 2950234 line |  | **chromosome 13** |
| 80 | CCAATTCACTGAGACCACG found at 3109031 line | 348 | CCAATGGCCAGGCACCACG found at 374763 line |
| 81 | CCAATCATCTTCCACCACG found at 3294212 line | 349 | CCAATACGGCAAAACCACG found at 422389 line |
| 82 | CCAATTCACGAAACCCACG found at 3295000 line | 350 | CCAATATGGTGAAACCACG found at 627234 line |
| 83 | CCAATGCACTTTAGCCACG found at 3367898 line | 351 | CCAATCAGATGCACCCACG found at 633513 line |
|  | **chromosome 3** | 352 | CCAATGTGGGACTTCCACG found at 645884 line |
| 84 | CCAATTTCTGAATCCCACG found at 24609 line | 353 | CCAATCCAAGGAATCCACG found at 777931 line |
| 85 | CCAATTTCTTTCTTCCACG found at 36316 line | 354 | CCAATGTGTACTTTCCACG found at 921489 line |
| 86 | CCAATGGTTTGACACCACG found at 107178 line | 355 | CCAATTCTGAATACCCACG found at 938654 line |
| 87 | CCAATTTCCCCACTCCACG found at 110205 line | 356 | CCAATGATTAGCTCCCACG found at 969487 line |
| 88 | CCAATCCCTTATTTCCACG found at 114165 line | 357 | CCAATCACCTCCCACCACG found at 1051809 line |
| 89 | CCAATCCCTTATTTCCACG found at 563940 line | 358 | CCAATCCCTTATTTCCACG found at 1121652 line |
| 90 | CCAATCCCTTATTTCCACG found at 1744218 line | 359 | CCAATCCCTTATTTCCACG found at 1208495 line |
| 91 | CCAATCCCTTATTTCCACG found at 2636994 line | 360 | CCAATAATCTCATCCCACG found at 1216988 line |
| 92 | CCAATGGCTGGCAACCACG found at 295311 line | 361 | CCAATATGATGACACCACG found at 1383707 line |
| 93 | CCAATGCCCAGAAACCACG found at 311293 line | 362 | CCAATATGATGAAACCACG found at 1487944 line |
| 94 | CCAATCGCCTCCCACCACG found at 317528 line | 363 | CCAATCACCCCCCACCACG found at 1555132 line |
| 95 | CCAATATGGTGAAACCACG found at 461775 line | 364 | CCAATATCTGTTCTCCACG found at 1586591 line |
| 96 | CCAATGATCTTAACCCACG found at 777377 line | 365 | CCAATGCGATGCTCCCACG found at 1593984 line |
| 97 | CCAATTGCATGTTTCCACG found at 793542 line |  | **chromosome 14** |
| 98 | CCAATCTAGTTTCACCACG found at 964737 line | 366 | CCAATCCTCATGGTCCACG found at 343463 line |
| 99 | CCAATCAAAAATTACCACG found at 977466 line | 367 | CCAATCCAAAGAATCCACG found at 518793 line |
| 100 | CCAATCCACCGAATCCACG found at 1010162 line | 368 | CCAATCCCTTATTTCCACG found at 536959 line |
| 101 | CCAATAAGAGTAAGCCACG found at 1051577 line | 369 | CCAATCCCTTATTTCCACG found at 536960 line |
| 102 | CCAATAAGAGTAAGCCACG found at 2747737 line | 370 | CCAATCCCTTATTTCCACG found at 536960 line |
| 103 | CCAATTCGCTGTCTCCACG found at 2087009 line | 371 | CCAATCCCTTATTTCCACG found at 583144 line |
| 104 | CCAATGAAGAATACCCACG found at 2093786 line | 372 | CCAATACGGTGAAACCACG found at 551569 line |
| 105 | CCAATATGATGAAACCACG found at 2456361 line | 373 | CCAATGTGGTGAAACCACG found at 776446 line |
| 106 | CCAATCACTCCTCACCACG found at 2477008 line | 374 | CCAATTTTTAATTCCCACG found at 869588 line |
| 107 | CCAATGCCTAGTTTCCACG found at 2533039 line | 375 | CCAATCATGCCTTCCCACG found at 874500 line |
| 108 | CCAATAGATCATCCCCACG found at 2602376 line | 376 | CCAATATGGTGAAACCACG found at 892788 line |
| 109 | CCAATAAAAAATGACCACG found at 2689927 line | 377 | CCAATTCCAAGCACCCACG found at 960845 line |
| 110 | CCAATTATTTAAGCCCACG found at 2690767 line | 378 | CCAATAGGTCTGTCCCACG found at 963276 line |
|  | **chromosome 4** | 379 | CCAATATAACGAAACCACG found at 1035627 line |
| 111 | CCAATTAGCGCACACCACG found at 16122 line | 380 | CCAATAATTTTTCTCCACG found at 1064360 line |
| 112 | CCAATCTCAACATGCCACG found at 104858 line | 381 | CCAATCAAACTCACCCACG found at 1241316 line |
| 113 | CCAATGTCAGCTGGCCACG found at 112300 line | 382 | CCAATCGCTTATTTCCACG found at 1381287 line |
| 114 | CCAATTCAAGCCCTCCACG found at 155852 line | 383 | CCAATACGGTAAAACCACG found at 1389584 line |
| 115 | CCAATGGTTGGACCCCACG found at 240576 line | 384 | CCAATCAGAGATTCCCACG found at 1489144 line |
| 116 | CCAATCACCTCCCACCACG found at 240991 line |  | **chromosome 15** |
| 117 | CCAATCACCTCCCACCACG found at 1181928 line | 385 | CCAATTCAGAGTTTCCACG found at 281787 line |
| 118 | CCAATTGCAGTTTCCCACG found at 335454 line | 386 | CCAATTCAGAGTTTCCACG found at 295776 line |
| 119 | CCAATGTCTTAAAACCACG found at 482543 line | 387 | CCAATTTGAAAAGGCCACG found at 371294 line |
| 120 | CCAATCGCCTTCCACCACG found at 486871 line | 388 | CCAATGCTAGCTGCCCACG found at 380923 line |
| 121 | CCAATTTTCTGGGTCCACG found at 508997 line | 389 | CCAATTCAGTGTCTCCACG found at 409296 line |
| 122 | CCAATATGGTGAAACCACG found at 590412 line | 390 | CCAATGATTCTAGTCCACG found at 450159 line |
| 123 | CCAATGTGGGCAAACCACG found at 664746 line | 391 | CCAATGCGGGGCTTCCACG found at 459593 line |
| 124 | CCAATGACCAGGCCCCACG found at 735027 line | 392 | CCAATCCTGAGTGTCCACG found at 551195 line |
| 125 | CCAATCCCCCCACCCCACG found at 773111 line | 393 | CCAATATGGTGAAACCACG found at 617537 line |
| 126 | CCAATTTATTGAAGCCACG found at 865498 line | 394 | CCAATATGGTGAAACCACG found at 1224034 line |
| 127 | CCAATCACCTCTCACCACG found at 886556 line | 395 | CCAATTATAGAGTCCCACG found at 763023 line |
| 128 | CCAATTACCTCCCACCACG found at 997210 line | 396 | CCAATGTTAACCTCCCACG found at 796512 line |
| 129 | CCAATGAAATCAGGCCACG found at 1052710 line | 397 | CCAATATATCTTCCCCACG found at 806003 line |
| 130 | CCAATCAGCCCCTGCCACG found at 1380254 line | 398 | CCAATTCCAGCCGCCCACG found at 837420 line |
| 131 | CCAATTCTTCATCTCCACG found at 1400974 line | 399 | CCAATTTCTGACTTCCACG found at 860147 line |
| 132 | CCAATATTTTAAGTCCACG found at 1505191 line | 400 | CCAATCACAGCTATCCACG found at 1130692 line |
| 133 | CCAATTTCCTCTTTCCACG found at 1856812 line | 401 | CCAATGCACCTGGCCCACG found at 1274280 line |
| 134 | CCAATGCTGAGTTGCCACG found at 2142291 line | 402 | CCAATGGTGAATTGCCACG found at 1323017 line |
|  | **chromosome 5** | 403 | CCAATTGCTGAATCCCACG found at 1338659 line |
| 135 | CCAATCCCTTATTTCCACG found at 100901 line |  | **chromosome 16** |
| 136 | CCAATCCCTTATTTCCACG found at 228074 line | 404 | CCAATGTGTTGAAACCACG found at 34568 line |
| 137 | CCAATCCCTTATTTCCACG found at 257440 line | 405 | CCAATATCTGGCACCCACG found at 55128 line |
| 138 | CCAATCCCTTATTTCCACG found at 1050754 line | 406 | CCAATCACAAAGGGCCACG found at 159190 line |
| 139 | CCAATCCCTTATTTCCACG found at 1128476 line | 407 | CCAATGCCAATTTGCCACG found at 185231 line |
| 140 | CCAATCACCAATCACCACG found at 153979 line | 408 | CCAATTTGACTGGGCCACG found at 248730 line |
| 141 | CCAATTACACTTCTCCACG found at 207224 line | 409 | CCAATCTATCCACTCCACG found at 263369 line |
| 142 | CCAATCTCCTGTCTCCACG found at 229484 line | 410 | CCAATAAACAGTAGCCACG found at 316806 line |
| 143 | CCAATCCCCGCACCCCACG found at 283517 line | 411 | CCAATCCTCCTCCTCCACG found at 414887 line |
| 144 | CCAATGTGGCAAAACCACG found at 761665 line | 412 | CCAATCACATCTTCCCACG found at 447153 line |
| 145 | CCAATTTCTACTTCCCACG found at 842995 line | 413 | CCAATCACATCTTCCCACG found at 460222 line |
| 146 | CCAATCCTCCACTCCCACG found at 948416 line | 414 | CCAATTTTATATACCCACG found at 729466 line |
| 147 | CCAATAAACACTCTCCACG found at 1026652 line | 415 | CCAATCCCTTAATTCCACG found at 834915 line |
| 148 | CCAATTGGTCATGTCCACG found at 1118452 line | 416 | CCAATCAGACATGGCCACG found at 860545 line |
| 149 | CCAATCCCCTTGATCCACG found at 1553702 line | 417 | CCAATTACTGTGACCCACG found at 862891 line |
| 150 | CCAATATGGTGAAACCACG found at 1588918 line | 418 | CCAATTAGAACACCCCACG found at 919250 line |
| 151 | CCAATCACCTCTCCCCACG found at 1808354 line | 419 | CCAATTCAATCCACCCACG found at 921844 line |
| 152 | CCAATGCCCCTTCCCCACG found at 1920323 line | 420 | CCAATTTCCTTCTTCCACG found at 1111264 line |
| 153 | CCAATCTCAAATTTCCACG found at 1924081 line | 421 | CCAATTAAATGACCCCACG found at 1183374 line |
| 154 | CCAATGAATGTTTTCCACG found at 1954587 line | 422 | CCAATCCCCACCCTCCACG found at 1230760 line |
| 155 | CCAATCACCTCCCACCACG found at 1959773 line | 423 | CCAATGGGCAGAGCCCACG found at 1233365 line |
| 156 | CCAATAGATAAGTGCCACG found at 2054828 line | 424 | CCAATAACCAACAGCCACG found at 1233874 line |
| 157 | CCAATGAATGGTAGCCACG found at 2193684 line | 425 | CCAATCCCTGGCTTCCACG found at 1237892 line |
| 158 | CCAATCCAATACTTCCACG found at 2272392 line |  | chromosome 17 |
| 159 | CCAATCTTCACACTCCACG found at 2487860 line | 426 | CCAATATGGTGAAACCACG found at 139353 line |
| 160 | CCAATCACCTCCCTCCACG found at 2506444 line | 427 | CCAATCCCTTATTTCCACG found at 164950 line |
|  | **chromosome 6** | 428 | CCAATTCTCACTCACCACG found at 220197 line |
| 161 | CCAATCCCTAGGTACCACG found at 70620 line | 429 | CCAATGTCACACAACCACG found at 263108 line |
| 162 | CCAATGAATAAGCTCCACG found at 165490 line | 430 | CCAATACATTTATACCACG found at 274196 line |
| 163 | CCAATACATCTCTACCACG found at 253666 line | 431 | CCAATTTATCAGCTCCACG found at 579507 line |
| 164 | CCAATGAGGAGCTACCACG found at 265200 line | 432 | CCAATGAGGCCTTCCCACG found at 599760 line |
| 165 | CCAATATGGTGAAACCACG found at 281534 line | 433 | CCAATGTGTTGAAACCACG found at 660225 line |
| 166 | CCAATCACCTCCCCCCACG found at 304588 line | 434 | CCAATAAACATCTCCCACG found at 703421 line |
| 167 | CCAATTTTCTATACCCACG found at 392311 line | 435 | CCAATATAGTGAAACCACG found at 827676 line |
| 168 | CCAATTATGTGCCACCACG found at 538766 line | 436 | CCAATTTATTTGACCCACG found at 929889 line |
| 169 | CCAATCAAACTGAACCACG found at 551433 line | 437 | CCAATCTTCTTTGACCACG found at 976531 line |
| 170 | CCAATCCATAACCACCACG found at 584679 line | 438 | CCAATCAGGCAGATCCACG found at 983669 line |
| 171 | CCAATACTGAATGACCACG found at 661393 line | 439 | CCAATATCCATGTGCCACG found at 1073851 line |
| 172 | CCAATATCTGGTGCCCACG found at 815644 line | 440 | CCAATGGCCCGACCCCACG found at 1080051 line |
| 173 | CCAATTACTTCAAACCACG found at 878238 line | 441 | CCAATCCAGAAAGACCACG found at 1083418 line |
| 174 | CCAATCATCCTCTCCCACG found at 978123 line | 442 | CCAATCCCCATCCCCCACG found at 1108027 line |
| 175 | CCAATATGGTGAGACCACG found at 987806 line |  | **chromosome 18** |
| 176 | CCAATAAACCTTGGCCACG found at 1111464 line | 443 | CCAATGGTAAGTGGCCACG found at 83448 line |
| 177 | CCAATGCTTAGGCTCCACG found at 1139481 line | 444 | CCAATATGGTGAAACCACG found at 165437 line |
| 178 | CCAATCCCTTATTTCCACG found at 1364286 line | 445 | CCAATATGGTGAAACCACG found at 603520 line |
| 179 | CCAATCCCTTATTTCCACG found at 1828412 line | 446 | CCAATATGGTGAAACCACG found at 654474 line |
| 180 | CCAATCCCTTATTTCCACG found at 1836444 line | 447 | CCAATCTGCTATGGCCACG found at 312531 line |
| 181 | CCAATCCCTTATTTCCACG found at 1976916 line | 448 | CCAATTATTCTTAACCACG found at 320617 line |
| 182 | CCAATAACTTGTGACCACG found at 1944822 line | 449 | CCAATGTTTCTGGACCACG found at 446677 line |
| 183 | CCAATCCCCAAAACCCACG found at 2059232 line | 450 | CCAATTATAAAGTCCCACG found at 568931 line |
| 184 | CCAATCACCTCCCTCCACG found at 2068849 line | 451 | CCAATCGGGGCGGTCCACG found at 618679 line |
| 185 | CCAATAAAAATTCACCACG found at 2115340 line | 452 | CCAATCGGGGCGGTCCACG found at 618762 line |
| 186 | CCAATCTGTTTACTCCACG found at 2247769 line | 453 | CCAATCGGGGCGGTCCACG found at 618844 line |
| 187 | CCAATTTAAATTAACCACG found at 2358073 line | 454 | CCAATTTGTTGGTCCCACG found at 643118 line |
|  | **chromosome 7** | 455 | CCAATATTCTTTGCCCACG found at 679296 line |
| 188 | CCAATCTGGCAGGACCACG found at 59193 line | 456 | CCAATAAAAATATCCCACG found at 829388 line |
| 189 | CCAATAAGACCCCACCACG found at 79680 line | 457 | CCAATTACTTTCCACCACG found at 974308 line |
| 190 | CCAATTAGAAGGTTCCACG found at 87188 line |  | **chromosome 19** |
| 191 | CCAATCCCTTATTTCCACG found at 339405 line | 458 | CCAATCCCGGCTTCCCACG found at 4774 line |
| 192 | CCAATCCCTTATTTCCACG found at 1743766 line | 459 | CCAATGCCAAGACCCCACG found at 29224 line |
| 193 | CCAATGCATAGTTACCACG found at 402250 line | 460 | CCAATCCGCGACACCCACG found at 45782 line |
| 194 | CCAATATGATGAAACCACG found at 425010 line | 461 | CCAATTCCACAGCTCCACG found at 61743 line |
| 195 | CCAATCACCTCCCACCACG found at 451122 line | 462 | CCAATAAACCGGCACCACG found at 71015 line |
| 196 | CCAATCACCTCCCACCACG found at 1589802 line | 463 | CCAATATGGTGAAACCACG found at 219070 line |
| 197 | CCAATCACCTCCCACCACG found at 1824308 line | 464 | CCAATGTTTAGCTCCCACG found at 394547 line |
| 198 | CCAATCACCTCCCACCACG found at 1914078 line | 465 | CCAATTCTCTTCTCCCACG found at 433244 line |
| 199 | CCAATGGTGAGGTTCCACG found at 631194 line | 466 | CCAATACGGTGAAACCACG found at 460325 line |
| 200 | CCAATCAGAAAAGACCACG found at 781208 line | 467 | CCAATGTAGTGAAACCACG found at 485093 line |
| 201 | CCAATATGGTGAAGCCACG found at 875782 line | 468 | CCAATGTGGTGAAACCACG found at 518418 line |
| 202 | CCAATGGGGAAAGGCCACG found at 1050335 line | 469 | CCAATTCACTGAGACCACG found at 587577 line |
| 203 | CCAATTCCCTGAGACCACG found at 1053029 line | 470 | CCAATCCATAATGACCACG found at 653179 line |
| 204 | CCAATTACATTTTTCCACG found at 1085892 line | 471 | CCAATGTTGCTTCTCCACG found at 723875 line |
| 205 | CCAATAGGTAAAGTCCACG found at 1239504 line | 472 | CCAATCAGGACGCTCCACG found at 729605 line |
| 206 | CCAATGTAAAATTACCACG found at 1253303 line | 473 | CCAATCATAATCCCCCACG found at 779660 line |
| 207 | CCAATTGGTGAATTCCACG found at 1318513 line | 474 | CCAATCAAATGGCACCACG found at 782611 line |
| 208 | CCAATATGGTAAAACCACG found at 1331402 line | 475 | CCAATGAAGCCCCTCCACG found at 804478 line |
| 209 | CCAATCACATTGGACCACG found at 1334672 line |  | **chromosome 20** |
| 210 | CCAATTCTGCCGCACCACG found at 1356143 line | 476 | CCAATGCGCAGGTGCCACG found at 125674 line |
| 211 | CCAATTTTTTGTGGCCACG found at 1587188 line | 477 | CCAATTGATAGGGACCACG found at 162257 line |
| 212 | CCAATCACCTCCAACCACG found at 1617011 line | 478 | CCAATAAGGTGAAACCACG found at 162748 line |
| 213 | CCAATGGGTAATACCCACG found at 1671072 line | 479 | CCAATATGGTGAAACCACG found at 495232 line |
| 214 | CCAATCTAGTCCCTCCACG found at 1742336 line | 480 | CCAATATGGTGAAACCACG found at 683816 line |
| 215 | CCAATGTTAGTTACCCACG found at 1749231 line | 481 | CCAATCTGCTTTAGCCACG found at 503214 line |
| 216 | CCAATATGGAGAAACCACG found at 1840609 line | 482 | CCAATCCCTTATTTCCACG found at 616517 line |
| 217 | CCAATTCTTCCTTTCCACG found at 1886662 line | 483 | CCAATCTACTGCATCCACG found at 651810 line |
| 218 | CCAATATATAAGGACCACG found at 2045782 line | 484 | CCAATAGTGCAGGGCCACG found at 691900 line |
| 219 | CCAATGTCCTGGAGCCACG found at 2096321 line | 485 | CCAATGGGCTGAAACCACG found at 754313 line |
| 220 | CCAATGGACCAGGCCCACG found at 2126419 line |  | **chromosome 21** |
| 221 | CCAATTTAAGGGGACCACG found at 2188335 line | 486 | CCAATTCAAATATGCCACG found at 363580 line |
|  | **chromosome 8** | 487 | CCAATCAGCCTTGTCCACG found at 408656 line |
| 222 | CCAATGCACAGATGCCACG found at 39548 line | 488 | CCAATGGGTCCCTCCCACG found at 452798 line |
| 223 | CCAATTATCATTCCCCACG found at 59165 line | 489 | CCAATATTGCTTACCCACG found at 492620 line |
| 224 | CCAATTTATTCTCTCCACG found at 82364 line | 490 | CCAATCCCCACCCCCCACG found at 596040 line |
| 225 | CCAATCCCTTATTTCCACG found at 100847 line |  | **chromosome 22** |
| 226 | CCAATCCCTTATTTCCACG found at 970644 line | 491 | CCAATCATATTTCACCACG found at 262772 line |
| 227 | CCAATATGGTGAAACCACG found at 115240 line | 492 | CCAATGGAGGCAACCCACG found at 263369 line |
| 228 | CCAATATGGTGAAACCACG found at 284478 line | 493 | CCAATCTGGGTTTGCCACG found at 276937 line |
| 229 | CCAATATGGTGAAACCACG found at 301966 line | 494 | CCAATCAGATTCTCCCACG found at 298211 line |
| 230 | CCAATCATACTTTTCCACG found at 122311 line | 495 | CCAATCAGATTCTCCCACG found at 319108 line |
| 231 | CCAATGCCTGAGCCCCACG found at 159462 line | 496 | CCAATCAGATTCTCCCACG found at 328539 line |
| 232 | CCAATCACCTCCCACCACG found at 375368 line | 497 | CCAATCAGATTCTCCCACG found at 347832 line |
| 233 | CCAATCACCTCCCACCACG found at 1999571 line | 498 | CCAATGTGGGTGAGCCACG found at 391006 line |
| 234 | CCAATCCTCCACTCCCACG found at 426204 line | 499 | CCAATCGCTTATTTCCACG found at 456943 line |
| 235 | CCAATCTGCCTGAGCCACG found at 565581 line | 500 | CCAATATGGTGAAACCACG found at 470829 line |
| 236 | CCAATTAGTAAAGCCCACG found at 729998 line | 501 | CCAATAGCAAGAGTCCACG found at 510603 line |
| 237 | CCAATGCTACCCCTCCACG found at 868360 line | 502 | CCAATGGGGAGGCCCCACG found at 549238 line |
| 238 | CCAATACCCTCTTACCACG found at 944187 line | 503 | CCAATGCCCTCAGCCCACG found at 646056 line |
| 239 | CCAATCTTCATAATCCACG found at 976484 line | 504 | CCAATGACGACAGTCCACG found at 691186 line |
| 240 | CCAATTCTTCATCTCCACG found at 986337 line |  | **chromosome x** |
| 241 | CCAATAAAAATAAACCACG found at 1015617 line | 505 | CCAATCAGTGATCTCCACG found at 110514 line |
| 242 | CCAATAAGTCCAGGCCACG found at 1333063 line | 506 | CCAATTTCGTTTTCCCACG found at 202048 line |
| 243 | CCAATGCACTTATGCCACG found at 1404068 line | 507 | CCAATAACCAGCACCCACG found at 238809 line |
| 244 | CCAATGGGCCCCTTCCACG found at 1522562 line | 508 | CCAATTCCAAGCTTCCACG found at 247717 line |
| 245 | CCAATTTTAATTTGCCACG found at 1565416 line | 509 | CCAATAACAGTACTCCACG found at 486447 line |
| 246 | CCAATAGACAGCCTCCACG found at 1611773 line | 510 | CCAATCAGTATAAACCACG found at 560173 line |
| 247 | CCAATTCATTCAGTCCACG found at 1641471 line | 511 | CCAATTTCCTTATCCCACG found at 585208 line |
| 248 | CCAATCCCAGCTCCCCACG found at 1972441 line | 512 | CCAATATGGTGAAACCACG found at 621380 line |
| 249 | CCAATGGCTGAAGACCACG found at 1989452 line | 513 | CCAATATGGTGAAACCACG found at 2008143 line |
| 250 | CCAATCTCCGGCGCCCACG found at 2024277 line | 514 | CCAATGTGGCAAAGCCACG found at 647757 line |
|  | **chromosome 9** | 515 | CCAATCTCAGCCCTCCACG found at 658764 line |
| 251 | CCAATATGTCATGGCCACG found at 276119 line | 516 | CCAATTTGGATTGACCACG found at 959770 line |
| 252 | CCAATAGATTGAATCCACG found at 295882 line | 517 | CCAATCCCTTATTTCCACG found at 1278899 line |
| 253 | CCAATAAAATGTAGCCACG found at 381818 line | 518 | CCAATCCCTTATTTCCACG found at 1685514 line |
| 254 | CCAATATTCTCACCCCACG found at 530249 line | 519 | CCAATCACCTCCCACCACG found at 1287748 line |
| 255 | CCAATTAGCTGCTGCCACG found at 642955 line | 520 | CCAATCACCTCCCACCACG found at 1761277 line |
| 256 | CCAATTAGCTGCTGCCACG found at 940301 line | 521 | CCAATATTTAGCTCCCACG found at 1320515 line |
| 257 | CCAATTTAATAACCCCACG found at 990769 line | 522 | CCAATTTATAGTAACCACG found at 1531667 line |
| 258 | CCAATATGATGAAACCACG found at 1077585 line | 523 | CCAATTAATCATCTCCACG found at 1537373 line |
| 259 | CCAATGGGTCCCTCCCACG found at 1092606 line | 524 | CCAATGTCCAGGTACCACG found at 1610089 line |
| 260 | CCAATATTTTACTGCCACG found at 1100758 line | 525 | CCAATTGTGCCAGTCCACG found at 1662050 line |
| 261 | CCAATACCCCTACTCCACG found at 1143378 line | 526 | CCAATCTCTGCCTCCCACG found at 1711030 line |
| 262 | CCAATCCCTTATTTCCACG found at 1161460 line | 527 | CCAATCATTCTGGTCCACG found at 1729396 line |
| 263 | CCAATCCCTTATTTCCACG found at 1334875 line | 528 | CCAATTAAAACTCCCCACG found at 1866086 line |
| 264 | CCAATGGCACCATGCCACG found at 1214195 line | 529 | CCAATGGTGCACTGCCACG found at 1888262 line |
| 265 | CCAATGCTACTCAGCCACG found at 1296364 line | 530 | CCAATCATACTTCTCCACG found at 2004183 line |
| 266 | CCAATGAGCCCTTCCCACG found at 1318667 line |  | **chromosome Y** |
| 267 | CCAATCTAGTCCTTCCACG found at 1331026 line | 531 | CCAATCGCTTATTTCCACG found at 80700 line |
| 268 | CCAATCCATAGTGCCCACG found at 1359515 line | 532 | CCAATGTGAATACTCCACG found at 117300 line |
| 269 | CCAATCTCCAGTACCCACG found at 1404294 line | 533 | CCAATGGGACCTGACCACG found at 133732 line |
| 270 | CCAATCTAATGTTTCCACG found at 1521533 line | 534 | CCAATGTATGATCTCCACG found at 254420 line |
| 271 | CCAATGACTGAGCTCCACG found at 1547293 line | 535 | CCAATACAGGAGCACCACG found at 373953 line |
| **ERSE-II** | | | |
| **SNO** | **chromosome 1** | 185 | ATTGGGCCACG found at 133927 line |
| 1 | ATTGGACCACG found at 80952 line | 186 | ATTGGGCCACG found at 492916 line |
| 2 | ATTGGACCACG found at 107845 line | 187 | ATTGGGCCACG found at 600061 line |
| 3 | ATTGGACCACG found at 1598543 line | 188 | ATTGGGCCACG found at 630480 line |
| 4 | ATTGGACCACG found at 2223339 line | 189 | ATTGGGCCACG found at 1091364 line |
| 5 | ATTGGACCACG found at 2454224 line | 190 | ATTGGGCCACG found at 1143771 line |
| 6 | ATTGGCCCACG found at 158362 line | 191 | ATTGGGCCACG found at 1634115 line |
| 7 | ATTGGCCCACG found at 217378 line | 192 | ATTGGGCCACG found at 1890952 line |
| 8 | ATTGGCCCACG found at 277603 line | 193 | ATTGGTCCACG found at 227638 line |
| 9 | ATTGGCCCACG found at 931449 line | 194 | ATTGGTCCACG found at 1220089 line |
| 10 | ATTGGCCCACG found at 1181268 line | 195 | ATTGGTCCACG found at 1809548 line |
| 11 | ATTGGCCCACG found at 2426961 line | 196 | ATTGGACCACG found at 1561741 line |
| 12 | ATTGGCCCACG found at 3184467 line | 197 | ATTGGACCACG found at 1674670 line |
| 13 | ATTGGGCCACG found at 185367 line | 198 | ATTGGACCACG found at 1844043 line |
| 14 | ATTGGGCCACG found at 218091 line |  | **chromosome 10** |
| 15 | ATTGGGCCACG found at 615624 line | 199 | ATTGGCCCACG found at 26454 line |
| 16 | ATTGGGCCACG found at 881019 line | 200 | ATTGGCCCACG found at 1140238 line |
| 17 | ATTGGGCCACG found at 921313 line | 201 | ATTGGCCCACG found at 1653140 line |
| 18 | ATTGGGCCACG found at 999485 line | 202 | ATTGGCCCACG found at 1775820 line |
| 19 | ATTGGGCCACG found at 1546086 line | 203 | ATTGGACCACG found at 98805 line |
| 20 | ATTGGGCCACG found at 2380410 line | 204 | ATTGGACCACG found at 148023 line |
| 21 | ATTGGGCCACG found at 2452550 line | 205 | ATTGGACCACG found at 893072 line |
| 22 | ATTGGGCCACG found at 2524822 line | 206 | ATTGGACCACG found at 1852157 line |
| 23 | ATTGGGCCACG found at 2687029 line | 207 | ATTGGTCCACG found at 775336 line |
| 24 | ATTGGGCCACG found at 3413412 line | 208 | ATTGGTCCACG found at 924994 line |
| 25 | ATTGGTCCACG found at 444164 line | 209 | ATTGGGCCACG found at 1093404 line |
| 26 | ATTGGTCCACG found at 1232536 line |  | **chromosome 11** |
| 27 | ATTGGTCCACG found at 1428703 line | 210 | ATTGGGCCACG found at 40905 line |
| 28 | ATTGGTCCACG found at 1485311 line | 211 | ATTGGGCCACG found at 41774 line |
| 29 | ATTGGTCCACG found at 1672503 line | 212 | ATTGGGCCACG found at 338009 line |
| 30 | ATTGGTCCACG found at 2297405 line | 213 | ATTGGGCCACG found at 826405 line |
| 31 | ATTGGTCCACG found at 2555339 line | 214 | ATTGGGCCACG found at 922590 line |
| 32 | ATTGGTCCACG found at 2869734 line | 215 | ATTGGGCCACG found at 1317471 line |
| 33 | ATTGGTCCACG found at 3189285 line | 216 | ATTGGGCCACG found at 1477268 line |
|  | **chromosome 2** | 217 | ATTGGGCCACG found at 1516949 line |
| 34 | ATTGGTCCACG found at 4803 line | 218 | ATTGGGCCACG found at 1531818 line |
| 35 | ATTGGTCCACG found at 219909 line | 219 | ATTGGGCCACG found at 1815194 line |
| 36 | ATTGGTCCACG found at 698545 line | 220 | ATTGGGCCACG found at 1844348 line |
| 37 | ATTGGTCCACG found at 897344 line | 221 | ATTGGCCCACG found at 79292 line |
| 38 | ATTGGTCCACG found at 1359995 line | 222 | ATTGGCCCACG found at 958520 line |
| 39 | ATTGGTCCACG found at 1806619 line | 223 | ATTGGCCCACG found at 1701994 line |
| 40 | ATTGGTCCACG found at 2229505 line | 224 | ATTGGACCACG found at 375281 line |
| 41 | ATTGGTCCACG found at 3305182 line | 225 | ATTGGACCACG found at 774178 line |
| 42 | ATTGGACCACG found at 71703 line | 226 | ATTGGACCACG found at 895706 line |
| 43 | ATTGGACCACG found at 422153 line | 227 | ATTGGACCACG found at 1277187 line |
| 44 | ATTGGACCACG found at 749491 line | 228 | ATTGGACCACG found at 1651779 line |
| 45 | ATTGGACCACG found at 1102365 line | 229 | ATTGGACCACG found at 1651781 line |
| 46 | ATTGGACCACG found at 2075773 line |  | **chromosome 12** |
| 47 | ATTGGACCACG found at 2237818 line | 230 | ATTGGGCCACG found at 11678 line |
| 48 | ATTGGACCACG found at 2532656 line | 231 | ATTGGGCCACG found at 149560 line |
| 49 | ATTGGACCACG found at 2619158 line | 232 | ATTGGGCCACG found at 205315 line |
| 50 | ATTGGACCACG found at 2777623 line | 233 | ATTGGGCCACG found at 690946 line |
| 51 | ATTGGGCCACG found at 493547 line | 234 | ATTGGGCCACG found at 699669 line |
| 52 | ATTGGGCCACG found at 1516117 line | 235 | ATTGGGCCACG found at 1096171 line |
| 53 | ATTGGGCCACG found at 2138803 line | 236 | ATTGGGCCACG found at 1337102 line |
| 54 | ATTGGGCCACG found at 2184621 line | 237 | ATTGGGCCACG found at 1339399 line |
| 55 | ATTGGGCCACG found at 2579315 line | 238 | ATTGGACCACG found at 188758 line |
| 56 | ATTGGGCCACG found at 2827239 line | 239 | ATTGGCCCACG found at 788764 line |
| 57 | ATTGGGCCACG found at 3267685 line | 240 | ATTGGCCCACG found at 1705585 line |
| 58 | ATTGGGCCACG found at 3340381 line | 241 | ATTGGCCCACG found at 1755631 line |
| 59 | ATTGGCCCACG found at 626808 line | 242 | ATTGGTCCACG found at 1710922 line |
| 60 | ATTGGCCCACG found at 699238 line | 243 | ATTGGTCCACG found at 1711109 line |
| 61 | ATTGGCCCACG found at 779776 line |  | **chromosome 13** |
| 62 | ATTGGCCCACG found at 962581 line | 244 | ATTGGTCCACG found at 436001 line |
| 63 | ATTGGCCCACG found at 1098696 line | 245 | ATTGGTCCACG found at 707891 line |
| 64 | ATTGGCCCACG found at 1199476 line | 246 | ATTGGTCCACG found at 1344924 line |
| 65 | ATTGGCCCACG found at 1641690 line | 247 | ATTGGACCACG found at 1155659 line |
| 66 | ATTGGCCCACG found at 2328755 line | 248 | ATTGGGCCACG found at 1436572 line |
| 67 | ATTGGCCCACG found at 3237285 line | 249 | ATTGGCCCACG found at 1444644 line |
|  | **chromosome 3** | 250 | ATTGGCCCACG found at 1542299 line |
| 68 | ATTGGTCCACG found at 230363 line |  | **chromosome 14** |
| 69 | ATTGGTCCACG found at 333663 line | 251 | ATTGGGCCACG found at 298224 line |
| 70 | ATTGGTCCACG found at 381371 line | 252 | ATTGGGCCACG found at 799206 line |
| 71 | ATTGGTCCACG found at 645039 line | 253 | ATTGGGCCACG found at 880883 line |
| 72 | ATTGGCCCACG found at 340560 line | 254 | ATTGGGCCACG found at 1300406 line |
| 73 | ATTGGCCCACG found at 553145 line | 255 | ATTGGGCCACG found at 1354853 line |
| 74 | ATTGGCCCACG found at 1849988 line | 256 | ATTGGCCCACG found at 493209 line |
| 75 | ATTGGCCCACG found at 1849989 line | 257 | ATTGGCCCACG found at 538008 line |
| 76 | ATTGGCCCACG found at 2458902 line | 258 | ATTGGCCCACG found at 1100270 line |
| 77 | ATTGGCCCACG found at 2700925 line | 259 | ATTGGCCCACG found at 1388158 line |
| 78 | ATTGGGCCACG found at 442351 line | 260 | ATTGGTCCACG found at 717403 line |
| 79 | ATTGGGCCACG found at 804503 line | 261 | ATTGGTCCACG found at 1082307 line |
| 80 | ATTGGGCCACG found at 1000778 line | 262 | ATTGGTCCACG found at 1211245 line |
| 81 | ATTGGGCCACG found at 1779492 line | 263 | ATTGGACCACG found at 774295 line |
| 82 | ATTGGACCACG found at 482425 line | 264 | ATTGGACCACG found at 789052 line |
| 83 | ATTGGACCACG found at 680313 line | 265 | ATTGGACCACG found at 1103566 line |
| 84 | ATTGGACCACG found at 1206961 line | 266 | ATTGGACCACG found at 1198936 line |
| 85 | ATTGGACCACG found at 2067270 line | 267 | ATTGGACCACG found at 1261930 line |
| 86 | ATTGGACCACG found at 2532215 line |  | **chromosome 15** |
| 87 | ATTGGACCACG found at 2615015 line | 268 | ATTGGTCCACG found at 594677 line |
| 88 | ATTGGACCACG found at 2732632 line | 269 | ATTGGGCCACG found at 764787 line |
|  | **chromosome 4** | 270 | ATTGGGCCACG found at 959808 line |
| 89 | ATTGGTCCACG found at 49761 line | 271 | ATTGGGCCACG found at 984404 line |
| 90 | ATTGGTCCACG found at 519146 line | 272 | ATTGGGCCACG found at 1379625 line |
| 91 | ATTGGTCCACG found at 617102 line | 273 | ATTGGCCCACG found at 784928 line |
| 92 | ATTGGTCCACG found at 912949 line | 274 | ATTGGCCCACG found at 862696 line |
| 93 | ATTGGACCACG found at 90987 line | 275 | ATTGGCCCACG found at 1131162 line |
| 94 | ATTGGACCACG found at 190260 line | 276 | ATTGGCCCACG found at 1306578 line |
| 95 | ATTGGACCACG found at 567399 line | 277 | ATTGGCCCACG found at 1405016 line |
| 96 | ATTGGACCACG found at 1420420 line | 278 | ATTGGACCACG found at 839484 line |
| 97 | ATTGGACCACG found at 1428473 line | 279 | ATTGGACCACG found at 1064562 line |
| 98 | ATTGGCCCACG found at 441153 line | 280 | ATTGGACCACG found at 1273565 line |
| 99 | ATTGGCCCACG found at 526245 line |  | **chromosome 16** |
| 100 | ATTGGCCCACG found at 671087 line | 281 | ATTGGGCCACG found at 97369 line |
| 101 | ATTGGCCCACG found at 2582164 line | 282 | ATTGGGCCACG found at 243382 line |
| 102 | ATTGGGCCACG found at 1135422 line | 283 | ATTGGGCCACG found at 791195 line |
| 103 | ATTGGGCCACG found at 2646839 line | 284 | ATTGGGCCACG found at 1170023 line |
|  | **chromosome 5** | 285 | ATTGGGCCACG found at 1171155 line |
| 104 | ATTGGTCCACG found at 41156 line | 286 | ATTGGGCCACG found at 1222105 line |
| 105 | ATTGGTCCACG found at 1219504 line | 287 | ATTGGACCACG found at 118659 line |
| 106 | ATTGGTCCACG found at 1800001 line | 288 | ATTGGACCACG found at 1079311 line |
| 107 | ATTGGTCCACG found at 2319794 line | 289 | ATTGGCCCACG found at 233466 line |
| 108 | ATTGGTCCACG found at 2341249 line | 290 | ATTGGCCCACG found at 311620 line |
| 109 | ATTGGTCCACG found at 2446045 line | 291 | ATTGGCCCACG found at 324192 line |
| 110 | ATTGGCCCACG found at 376224 line | 292 | ATTGGCCCACG found at 417306 line |
| 111 | ATTGGCCCACG found at 1315015 line | 293 | ATTGGCCCACG found at 1137084 line |
| 112 | ATTGGCCCACG found at 1657801 line | 294 | ATTGGTCCACG found at 851094 line |
| 113 | ATTGGCCCACG found at 2398485 line |  | **chromosome 17** |
| 114 | ATTGGCCCACG found at 2417164 line | 295 | ATTGGCCCACG found at 91507 line |
| 115 | ATTGGCCCACG found at 2463766 line | 296 | ATTGGCCCACG found at 932115 line |
| 116 | ATTGGCCCACG found at 2477366 line | 297 | ATTGGACCACG found at 110131 line |
| 117 | ATTGGGCCACG found at 733916 line | 298 | ATTGGACCACG found at 461245 line |
| 118 | ATTGGGCCACG found at 773771 line | 299 | ATTGGGCCACG found at 162509 line |
| 119 | ATTGGGCCACG found at 1117505 line | 300 | ATTGGGCCACG found at 403527 line |
| 120 | ATTGGGCCACG found at 2189221 line | 301 | ATTGGGCCACG found at 533719 line |
| 121 | ATTGGGCCACG found at 2223306 line | 302 | ATTGGGCCACG found at 1047065 line |
| 122 | ATTGGGCCACG found at 2232028 line | 303 | ATTGGGCCACG found at 1099784 line |
| 123 | ATTGGACCACG found at 1624936 line | 304 | ATTGGTCCACG found at 800714 line |
| 124 | ATTGGACCACG found at 2045234 line |  | **chromosome 18** |
|  | **chromosome 6** | 305 | ATTGGCCCACG found at 5109 line |
| 125 | ATTGGTCCACG found at 56651 line | 306 | ATTGGCCCACG found at 55912 line |
| 126 | ATTGGTCCACG found at 1526130 line | 307 | ATTGGCCCACG found at 191790 line |
| 127 | ATTGGTCCACG found at 1612315 line | 308 | ATTGGCCCACG found at 780541 line |
| 128 | ATTGGTCCACG found at 1615124 line | 309 | ATTGGCCCACG found at 1053893 line |
| 129 | ATTGGTCCACG found at 1772298 line | 310 | ATTGGACCACG found at 957658 line |
| 130 | ATTGGACCACG found at 92054 line |  | **chromosome 19** |
| 131 | ATTGGACCACG found at 173693 line | 311 | ATTGGCCCACG found at 46768 line |
| 132 | ATTGGGCCACG found at 141744 line | 312 | ATTGGCCCACG found at 138181 line |
| 133 | ATTGGGCCACG found at 908877 line | 313 | ATTGGCCCACG found at 613236 line |
| 134 | ATTGGGCCACG found at 1524997 line | 314 | ATTGGCCCACG found at 641660 line |
| 135 | ATTGGGCCACG found at 2021852 line | 315 | ATTGGCCCACG found at 687455 line |
| 136 | ATTGGGCCACG found at 2360719 line | 316 | ATTGGCCCACG found at 701985 line |
|  | **chromosome 7** | 317 | ATTGGCCCACG found at 794498 line |
| 137 | ATTGGTCCACG found at 2211 line | 318 | ATTGGGCCACG found at 188935 line |
| 138 | ATTGGTCCACG found at 69504 line | 319 | ATTGGGCCACG found at 231899 line |
| 139 | ATTGGTCCACG found at 1123090 line | 320 | ATTGGGCCACG found at 670916 line |
| 140 | ATTGGTCCACG found at 1242125 line | 321 | ATTGGACCACG found at 194989 line |
| 141 | ATTGGTCCACG found at 1440129 line | 322 | ATTGGACCACG found at 588381 line |
| 142 | ATTGGTCCACG found at 1687170 line | 323 | ATTGGTCCACG found at 589005 line |
| 143 | ATTGGTCCACG found at 2007590 line | 324 | ATTGGTCCACG found at 723264 line |
| 144 | ATTGGGCCACG found at 369999 line |  | **chromosome 20** |
| 145 | ATTGGGCCACG found at 547366 line | 325 | ATTGGGCCACG found at 71574 line |
| 146 | ATTGGGCCACG found at 603209 line | 326 | ATTGGGCCACG found at 521718 line |
| 147 | ATTGGGCCACG found at 948390 line | 327 | ATTGGGCCACG found at 824169 line |
| 148 | ATTGGGCCACG found at 1380024 line | 328 | ATTGGTCCACG found at 169006 line |
| 149 | ATTGGGCCACG found at 1386725 line | 329 | ATTGGTCCACG found at 832818 line |
| 150 | ATTGGGCCACG found at 1744824 line | 330 | ATTGGTCCACG found at 839017 line |
| 151 | ATTGGGCCACG found at 1796628 line | 331 | ATTGGCCCACG found at 505790 line |
| 152 | ATTGGGCCACG found at 2129412 line | 332 | ATTGGCCCACG found at 868862 line |
| 153 | ATTGGCCCACG found at 933314 line |  | **chromosome 21** |
| 154 | ATTGGCCCACG found at 1029633 line | 333 | ATTGGGCCACG found at 136607 line |
| 155 | ATTGGCCCACG found at 1354424 line | 334 | ATTGGGCCACG found at 264563 line |
| 156 | ATTGGCCCACG found at 1858574 line | 335 | ATTGGGCCACG found at 570204 line |
| 157 | ATTGGCCCACG found at 2186092 line | 336 | ATTGGCCCACG found at 346564 line |
| 158 | ATTGGACCACG found at 990813 line | 337 | ATTGGCCCACG found at 403778 line |
| 159 | ATTGGACCACG found at 1334672 line |  | **chromosome 22** |
| 160 | ATTGGACCACG found at 1800880 line | 338 | ATTGGGCCACG found at 324333 line |
| 161 | ATTGGACCACG found at 1907438 line | 339 | ATTGGACCACG found at 330279 line |
| 162 | ATTGGACCACG found at 2156149 line | 340 | ATTGGTCCACG found at 559562 line |
|  | **chromosome 8** | 341 | ATTGGCCCACG found at 570676 line |
| 163 | ATTGGGCCACG found at 85859 line | 342 | ATTGGCCCACG found at 697533 line |
| 164 | ATTGGGCCACG found at 798563 line | 343 | ATTGGCCCACG found at 697533 line |
| 165 | ATTGGGCCACG found at 1240816 line |  | **chromosome X** |
| 166 | ATTGGGCCACG found at 1713034 line | 344 | ATTGGGCCACG found at 140577 line |
| 167 | ATTGGGCCACG found at 1818715 line | 345 | ATTGGGCCACG found at 523765 line |
| 168 | ATTGGTCCACG found at 121619 line | 346 | ATTGGCCCACG found at 305752 line |
| 169 | ATTGGTCCACG found at 183941 line | 347 | ATTGGCCCACG found at 479046 line |
| 170 | ATTGGTCCACG found at 846500 line | 348 | ATTGGCCCACG found at 1026588 line |
| 171 | ATTGGTCCACG found at 1083271 line | 349 | ATTGGCCCACG found at 1082119 line |
| 172 | ATTGGTCCACG found at 1535970 line | 350 | ATTGGCCCACG found at 1274506 line |
| 173 | ATTGGTCCACG found at 1621381 line | 351 | ATTGGCCCACG found at 1513569 line |
| 174 | ATTGGCCCACG found at 173181 line | 352 | ATTGGTCCACG found at 348827 line |
| 175 | ATTGGCCCACG found at 369116 line | 353 | ATTGGTCCACG found at 1145251 line |
| 176 | ATTGGCCCACG found at 513544 line | 354 | ATTGGTCCACG found at 1169514 line |
| 177 | ATTGGCCCACG found at 1030569 line | 355 | ATTGGTCCACG found at 1940217 line |
| 178 | ATTGGACCACG found at 1527485 line | 356 | ATTGGTCCACG found at 1988885 line |
|  | **chromosome 9** | 357 | ATTGGTCCACG found at 2119691 line |
| 179 | ATTGGCCCACG found at 10165 line | 358 | ATTGGTCCACG found at 2124181 line |
| 180 | ATTGGCCCACG found at 388817 line | 359 | ATTGGACCACG found at 386488 line |
| 181 | ATTGGCCCACG found at 465097 line | 360 | ATTGGACCACG found at 1695790 line |
| 182 | ATTGGCCCACG found at 1496592 line | 361 | ATTGGACCACG found at 2101084 line |
| 183 | ATTGGCCCACG found at 1625497 line |  | **chromosome Y** |
| 184 | ATTGGCCCACG found at 1889510 line | 362 | ATTGGGCCACG found at 268143 line |
| **ERSE-III** | | | |
| **SNO** | **chromosome 1** | **SNO** |  |
| 1 | CCAATGTCTGTTGAACAGCTATCACGTTGAACCACG found at 52800 line | 209 | CCAATGAATCCTTTACCCGCAATGAGAATGTCCACG found at 650398 line |
| 2 | CCAATACTTGACAGCCCATTCATTACACCCGCCACG found at 69402 line | 210 | CCAATGAATCCTTTACCCGCAATGAGAATGTCCACG found at 657076 line |
| 3 | CCAATTCCCTTACGCGAGAGGAACACAAGCCCCACG found at 93035 line | 211 | CCAATGAATCCTTTACCCGCAATGAGAATGTCCACG found at 680811 line |
| 4 | CCAATTCTGCCACAGGCCCCGTCACCTTCTCCCACG found at 197504 line | 212 | CCAATGATCCAACCAGCTGGAGCAGCAGCAACCACG found at 674203 line |
| 5 | CCAATTGCCTGGTAACAACATGTTCACACTTCCACG found at 243376 line | 213 | CCAATACAGGGGAGGTCATCATAGGTAGAGCCCACG found at 934095 line |
| 6 | CCAATGGGTGCAGTTCTGAGACTTTGGCCTTCCACG found at 254040 line | 214 | CCAATGGTAATAGGTAGTTATACTACAATATCCACG found at 1209084 line |
| 7 | CCAATGGCCCCTGGAAGATAGGAGTCCACTTCCACG found at 273354 line | 215 | CCAATGAATCCTTTACCTGAAATGAGAATGTCCACG found at 1238675 line |
| 8 | CCAATGACAGGCTGATACAGGCTACAAGAGCCCACG found at 308359 line | 216 | CCAATTCTTCTCAGAGGCCGAGAATATTCTTCCACG found at 1429999 line |
| 9 | CCAATGAATACAATATCATCTCACAGATGAACCACG found at 423931 line | 217 | CCAATGACTGCACCTTTGTCTTTGTACATGTCCACG found at 1572312 line |
| 10 | CCAATGCCTTTGGGACCTCCAAATGCAATTACCACG found at 541670 line | 218 | CCAATAAATGGCTAAATCAGGAGTTACTATTCCACG found at 1575890 line |
| 11 | CCAATTAGGCACCCAGGACTATCCAATTTGGCCACG found at 609684 line | 219 | CCAATCTGCCAGGCCTCTGTGCCCTCACTGCCCACG found at 1854419 line |
| 12 | CCAATGTAATACTTGGACACAACAATATCTCCCACG found at 664780 line | 220 | CCAATGTGGCAGCTCCCCCGTCCTGGGACATCCACG found at 1868937 line |
| 13 | CCAATGGTCTAGGGCATTCCAGGATATTCCACCACG found at 706291 line |  | **chromosome 11** |
| 14 | CCAATTAGAATTACTTACAAGGTCATACATACCACG found at 710979 line | 221 | CCAATGGGAGCCGTGAGGAATGCTACTGGGGCCACG found at 4044 line |
| 15 | CCAATATCACACTTTGGCTCCCCAAAGATGTCCACG found at 1045304 line | 222 | CCAATTGGGTTCCCGCCCATGTTATTGGCCCCCACG found at 89045 line |
| 16 | CCAATGAAAAAGACCAAAGAGTTTCAATTAGCCACG found at 1567866 line | 223 | CCAATTTTTCTATTTTTAATACAGGGTTTCACCACG found at 254980 line |
| 17 | CCAATGGGGAAGACATAGAGAATGAGGTGTTCCACG found at 1640174 line | 224 | CCAATTCTTCCTAGCTCTGGAGCCTACCATGCCACG found at 415830 line |
| 18 | CCAATCTACAGGCCCTTACTTTTCCCTCCTACCACG found at 2104632 line | 225 | CCAATACTCCATCCTGCAATTCACCATCCTTCCACG found at 535954 line |
| 19 | CCAATAGTAAGAAAATTAAATGACATAAACTCCACG found at 2118823 line | 226 | CCAATGGTGCAATCTTGGCTCACTGCAACCTCCACG found at 769131 line |
| 20 | CCAATTTTCCCCAGGCAGCTGCGGTTGATGCCCACG found at 2128703 line | 227 | CCAATGACTTACTAGTTACTCACTGACTTGACCACG found at 815221 line |
| 21 | CCAATCTCCTGAGTCAACACTACCATGAAAACCACG found at 2148193 line | 228 | CCAATCTCAAAGTATTGCACACTGCATGACTCCACG found at 840465 line |
| 22 | CCAATGTCCCTTAGGGGAGTAAAATTCCTCTCCACG found at 2393887 line | 229 | CCAATCAAACTCCACGCCTCCACCCGAGGCGCCACG found at 843334 line |
| 23 | CCAATGTAGAAGCTGCTCATCTGGCCCAGGTCCACG found at 2983082 line | 230 | CCAATGCGGCTGCCAAGACCACGGCCAGCAACCACG found at 887292 line |
| 24 | CCAATTCAAAAGCCCAGATCCTCCCACAGAACCACG found at 3014731 line | 231 | CCAATGACCAAATAAGTAAAGAGGGGAGCCACCACG found at 1203649 line |
| 25 | CCAATGCCATCTCAGTCCATCTCACAATGCCCCACG found at 3046436 line | 232 | CCAATTACGCACTCTGAATCTGGGGAAATAACCACG found at 1258812 line |
| 26 | CCAATCCATATGACCTTTTATTCTTTTTTCCCCACG found at 3162679 line | 233 | CCAATCATAAATGGATATCCAAGACTGAAGTCCACG found at 1543365 line |
| 27 | CCAATCTCAGCTTTTGGTCTCAGCCATCAAGCCACG found at 3182617 line | 234 | CCAATGGCCCTGCTGACCCTGTAGATGTCCACCACG found at 1566127 line |
|  | **chromosome 2** | 235 | CCAATGCATGGAGCAGGTACAGTGCCTACCGCCACG found at 1703347 line |
| 28 | CCAATAATGTCAGCACAAACCCAATTTCTTTCCACG found at 77917 line | 236 | CCAATCAGGGTTATCAAGCCCAGGTTCCCCACCACG found at 1727966 line |
| 29 | CCAATCACCTCATCAGAGCAGGTGCTGGTATCCACG found at 92129 line | 237 | CCAATTTATGATACTTACTGAGACCTTGGCACCACG found at 1756566 line |
| 30 | CCAATTTTTCATTAGGGCCTGCAACTCACAACCACG found at 117258 line | 238 | CCAATTTCCTCACTTCTCCTTGGGGAGAATGCCACG found at 1758120 line |
| 31 | CCAATGTCTTCCCACAGCTTTATTTCCACAACCACG found at 224793 line | 239 | CCAATCTCCTTAGCTGGCCAATCTCCCTCTCCCACG found at 1760607 line |
| 32 | CCAATGGAACATCCCTCAGGCTGTCAATTGACCACG found at 308122 line | 240 | CCAATGTGAGAAAGACATGAGATCTGGGGAGCCACG found at 1797530 line |
| 33 | CCAATAGGTTTTGTGGAGGAGGAAGGAGGAGCCACG found at 355502 line |  | **chromosome 12** |
| 34 | CCAATACCTCCACCAGAGCAGGTGCTGGTATCCACG found at 428410 line | 241 | CCAATCTCAGTTCCAAAACGAAAAGGGATGGCCACG found at 366082 line |
| 35 | CCAATCAACAGAGATCCATATCTTCGATAAGCCACG found at 769306 line | 242 | CCAATGGCGCCATCTCAGTTCACTGCAACCTCCACG found at 459192 line |
| 36 | CCAATCCTATTCCTTTTCTAGCAGCAGGGTGCCACG found at 885727 line | 243 | CCAATTCCCACTTTTCATCACCCCCTGGCAACCACG found at 558843 line |
| 37 | CCAATATAGCTGGGACTACAGGCGCCCGCCTCCACG found at 892359 line | 244 | CCAATGCAATTGTGAAACTTGAAACCAGGACCCACG found at 569322 line |
| 38 | CCAATACTGCACAGCGGCCACTGTCCCTCTTCCACG found at 970682 line | 245 | CCAATCCAGAAATACCTGACTCCACAGACAGCCACG found at 636053 line |
| 39 | CCAATCCGTGACCGGCGCCGGAGTTTTGGGTCCACG found at 990520 line | 246 | CCAATTGACTCCACCTGTGCCTCACTTTCTCCCACG found at 689736 line |
| 40 | CCAATTGGGACCGCTGACTCGGGCTGGGTTCCCACG found at 1085867 line | 247 | CCAATGCCGCCAGTGTTCTAGCCCCTTTCTTCCACG found at 751260 line |
| 41 | CCAATCTGCAGTATTGATTTGAAAGATGATGCCACG found at 1388388 line | 248 | CCAATTTTCCAATATATTTATCGAAAAAAATCCACG found at 960062 line |
| 42 | CCAATGGGATGTCACCCAGTGTCCAGCCTTGCCACG found at 1391505 line | 249 | CCAATAAGCACCCTTGCAACACCACCCCCATCCACG found at 1035824 line |
| 43 | CCAATAGGAATGGATGCTCCGACAGACGAAGCCACG found at 1470186 line | 250 | CCAATGGGTCAGTTGCTATAGAACAACAACACCACG found at 1091754 line |
| 44 | CCAATGGAGCGCGGTTCAGATCCCAGATCTACCACG found at 1470746 line | 251 | CCAATGAAACAGAATATACCCAGAAATAAAGCCACG found at 1106586 line |
| 45 | CCAATCTCTGGCTGTCCTTTCATTCTCGACTCCACG found at 1482575 line | 252 | CCAATATACCTCAGCCTACACATGTGAATGCCCACG found at 1200653 line |
| 46 | CCAATGCTGGAGTAAGAGAAGAATCCTCAGACCACG found at 1513381 line | 253 | CCAATCCTATTGACTACTTGCCTTCCCCATGCCACG found at 1242269 line |
| 47 | CCAATTAGGCATCGTGAGCTCCACCAGCATGCCACG found at 1709725 line | 254 | CCAATTTCCACTGGCAGGGCTGCAGCTGCTTCCACG found at 1255161 line |
| 48 | CCAATCATAAATAAGATAAATGACCAATAACCCACG found at 1826239 line | 255 | CCAATTCAGGGGTCCTTGATCAGACAACCCTCCACG found at 1482168 line |
| 49 | CCAATTAGCATTAAAATGTCATAATTAATTACCACG found at 1989272 line | 256 | CCAATACCTTTGTTTGAGTAGCCCATCGTTTCCACG found at 1497107 line |
| 50 | CCAATGACTCTAAAGATTATGGAGATGATGACCACG found at 2272296 line | 257 | CCAATGAATGTATAGACCACAGCTTGTTTGTCCACG found at 1518557 line |
| 51 | CCAATCTAAACTCCTCCACCCCCTGGCCATACCACG found at 2429388 line | 258 | CCAATTTCAGCCTTCAGTTTTCTCAGAAATCCCACG found at 1526883 line |
| 52 | CCAATAAGTAGCTGCTCTTATAACCAAGAAGCCACG found at 2817086 line | 259 | CCAATAGCTGAACTTAATTCATCTGTAGTTCCCACG found at 1527871 line |
| 53 | CCAATTCTCTGTCTTTTGTCCTTTAGAAAACCCACG found at 2820613 line | 260 | CCAATGGAGGGACCGTCTGTGCGAGAACCGGCCACG found at 1578988 line |
| 54 | CCAATTATATGACATTTTGAAAAAGGCAAAACCACG found at 2918796 line | 261 | CCAATACTGATACATTATTATTAACTAAAGTCCACG found at 1655907 line |
| 55 | CCAATGGATTATTTGAAAAAGAGGAGCACACCCACG found at 3066010 line | 262 | CCAATTTGCACACGTGGCATCTCTAAGGGTCCCACG found at 1659605 line |
| 56 | CCAATAAAAGCCCCACATTCATCCTTCAAGTCCACG found at 3163457 line | 263 | CCAATAGGGAACTGACTAAATTATAGTAAATCCACG found at 1705033 line |
| 57 | CCAATCACTGACCACTGGGTGTAAACACTTTCCACG found at 3193524 line | 264 | CCAATTTAAGGTATCAGGCTGATTTTTAAAACCACG found at 1719213 line |
| 58 | CCAATGGGCACAGCCCCACCCCTCTTCACGACCACG found at 3309491 line | 265 | CCAATTTAGAGAATCCATATGTACACATCCTCCACG found at 1853399 line |
| 59 | CCAATCCTTGCTTCCAGGAACCCAGCAGCTCCCACG found at 3353745 line |  | **chromosome 13** |
|  | **chromosome 3** | 266 | CCAATCTCCACCTTCCAACACCCAGTGCCCTCCACG found at 269081 line |
| 60 | CCAATCACATAGATGATATTGCTAGACTCTGCCACG found at 27883 line | 267 | CCAATTCTTCAATTACACATAAAGCACTTAACCACG found at 354666 line |
| 61 | CCAATTCAGAAAAAACAGAAGGACTCATGGGCCACG found at 198272 line | 268 | CCAATCGAAAGGGCCTTATTTTTAACTCACCCCACG found at 406843 line |
| 62 | CCAATGTCCATAGTATTTGCTCCTTCACTTACCACG found at 216807 line | 269 | CCAATTTTGGAAGAGTTAGCTGGCAGAGTCTCCACG found at 595463 line |
| 63 | CCAATTTTCATACTCTTGGAAGTCATATTATCCACG found at 436658 line | 270 | CCAATGCCCACTTGCCTTGTCAAGTACCTGGCCACG found at 598206 line |
| 64 | CCAATACGAACGCTACAGCTTCCGCAGCTTCCCACG found at 460497 line | 271 | CCAATAACAATCAAAATGTATATGTTAAATTCCACG found at 647455 line |
| 65 | CCAATCCTGAGTATAAACTTCTATAAATATGCCACG found at 551273 line | 272 | CCAATGTACAACTCTCAATGCGGAGTTGCCGCCACG found at 726958 line |
| 66 | CCAATGCTTGAATTCAACCCAGATGAAAGTTCCACG found at 643210 line | 273 | CCAATAGCTGGGGCTCCTCAGGCAACCCCCTCCACG found at 740779 line |
| 67 | CCAATGTTTATTGCAGCAAGATTCACGATAGCCACG found at 932504 line | 274 | CCAATAGGGACTCTGTGTGGGGGCTCTGACCCCACG found at 891036 line |
| 68 | CCAATTCTTGGTATTAATAATAGAATTTGATCCACG found at 1011034 line | 275 | CCAATGCCCTTCCTCTTGGCCTCCTTTTCAGCCACG found at 923440 line |
| 69 | CCAATTATGCATTCTGGCACTGGGGAAATGACCACG found at 1080330 line | 276 | CCAATATATAGTTTCTCAAAGTGAGTCCTCACCACG found at 933032 line |
| 70 | CCAATTATGCATTCTGGCACTGGGGAAATGACCACG found at 1998986 line | 277 | CCAATCTGTCCTGTAAGTCCTGAGTGCAGACCCACG found at 1044909 line |
| 71 | CCAATACCTTCAGCTTAAAATATTCAATATGCCACG found at 1091977 line | 278 | CCAATCAGAGGTACTTTCAATTTTTCATCTGCCACG found at 1077922 line |
| 72 | CCAATTCTCTTTAGATGTCATTCCCCTGTTCCCACG found at 1476464 line | 279 | CCAATACCTGATAAAATAAAAAAGTTGCAGACCACG found at 1268715 line |
| 73 | CCAATAATGAACTTATCCATATAACAAAATACCACG found at 1649701 line | 280 | CCAATTCTACGTAACAGCAACTCCACTGTGTCCACG found at 1333944 line |
| 74 | CCAATATGTTTCTTACGTCGTGTGTTGAGAGCCACG found at 1665550 line | 281 | CCAATCAGAGATACGTACAATTTTCCATCTGCCACG found at 1385515 line |
| 75 | CCAATGGCCAGGGCGCCCCAGCCTTCCTGAGCCACG found at 1765864 line | 282 | CCAATGGTTGTTGTTTTGTTTATGATGAATGCCACG found at 1412340 line |
| 76 | CCAATTCTAATTATTTAGGGGGAAAAGAAATCCACG found at 2078068 line | 283 | CCAATCCACAAAGAATCTTCAAGTGGGCACTCCACG found at 1572620 line |
| 77 | CCAATAGTCAAGTTACAGAACATTCCTATCACCACG found at 2371521 line |  | **chromosome 14** |
| 78 | CCAATATTTTTTTACTTACAGAAATTTTAGGCCACG found at 2483296 line | 284 | CCAATAGCTTGAGTGGCAATGAAATACAGTGCCACG found at 517233 line |
| 79 | CCAATAGGTGCCGCGAAGCAGCAGCCCACCCCCACG found at 2556605 line | 285 | CCAATTTTGGAATTTGAGAACCATTTAGACTCCACG found at 663298 line |
| 80 | CCAATGACCACTTTTCTGGAAGACAGTTTTTCCACG found at 2616411 line | 286 | CCAATACTAACCATTAACTTTTGGCTCTGAGCCACG found at 751351 line |
|  | **chromosome 4** | 287 | CCAATAAGCCTTAACTAAAGCTATGACTTAACCACG found at 763378 line |
| 81 | CCAATCGTGATCGCTACTTACAGAGCACCCGCCACG found at 62218 line | 288 | CCAATATTTTTATTAGTTTATAATTACTAAGCCACG found at 877714 line |
| 82 | CCAATACAGCCCTGCTCATCTTATCGGGGGCCCACG found at 87938 line | 289 | CCAATCAGCTGTCTTGAACACCCACTTTGATCCACG found at 957981 line |
| 83 | CCAATGCGGCAGGCCCTCCACAACAGGGTCCCCACG found at 88244 line | 290 | CCAATCTGCCGTGTTGCATTTTCACTTTGAACCACG found at 1055037 line |
| 84 | CCAATGCAGCAGCCGCACTTCCCCGTCTGACCCACG found at 107519 line | 291 | CCAATGTGCTGAATTCATTGTCATTTAAACCCCACG found at 1103648 line |
| 85 | CCAATTAAATGGGTACTGGAGGCAGCCATGGCCACG found at 314015 line | 292 | CCAATTCAATGGTGCTTTTTCCAAAGCCCACCCACG found at 1181186 line |
| 86 | CCAATGGGGACATTGTGTGGGGGCTCCAAACCCACG found at 453496 line | 293 | CCAATTTCCCCAGAGGATATCATGAAAGCTTCCACG found at 1352381 line |
| 87 | CCAATCTGTGTCTTTGCTTTCACTTCATATGCCACG found at 624863 line | 294 | CCAATCAACACAGATAGAGGACCAGCCAAGTCCACG found at 1411922 line |
| 88 | CCAATCCCACTCCACTCGCAGCCCTAGGCAACCACG found at 1242622 line | 295 | CCAATGAGGAGAAAAGCACAGTGGAGGAGTGCCACG found at 1417229 line |
| 89 | CCAATATTCCAAGCTATTTGGACACCAAAGTCCACG found at 1359822 line | 296 | CCAATGCGATGTCAGTGTCCTGGTCCAATTCCCACG found at 1471922 line |
| 90 | CCAATGTTGTTCCCTCATTCTCCTATAAGCACCACG found at 1408325 line |  | **chromosome 15** |
| 91 | CCAATTAAATCAGAATCTCTGGGGCTGAGACCCACG found at 1557758 line | 297 | CCAATTCTCACCTGAGAGCCCTGCAGACGCCCCACG found at 289307 line |
| 92 | CCAATTACATGGTCCATTTTAGAATTAAGTGCCACG found at 1712647 line | 298 | CCAATTCTCACCTGAGAGCCCTGCAGACGCCCCACG found at 326355 line |
| 93 | CCAATCTGCTCAGGTTTGAATCCTAGCTGTACCACG found at 1809702 line | 299 | CCAATAAGCCTTCACTAAAGTTATGACTTAACCACG found at 330240 line |
| 94 | CCAATGTTTCCATTAATAGACAAGAGCACCACCACG found at 1953638 line | 300 | CCAATCGCCCATCTGTGGAAAAAAGTGTCTTCCACG found at 373639 line |
| 95 | CCAATGAATGCCCATTAGATCAGAGATTATGCCACG found at 1956538 line | 301 | CCAATGTAGAAGCTGCTCATCTGGCCCAGGTCCACG found at 398653 line |
| 96 | CCAATTCTGCAAGGTAACAAGTGCTGCAGAACCACG found at 1957643 line | 302 | CCAATGCTATCCCTCCCCCATCCCCCCCACCCCACG found at 621470 line |
| 97 | CCAATTATGCATTCTGGAACTGGGGAAATAACCACG found at 2175882 line | 303 | CCAATCATGTTATGATATTATTGGTTTTTGTCCACG found at 703943 line |
| 98 | CCAATTCACACACTAAGGGAGGTTTTTCAAACCACG found at 2262864 line | 304 | CCAATCAACCACCTGCCTTGGGGCCAGGTGGCCACG found at 934709 line |
| 99 | CCAATTTGGAAAGCATATTTCAGGATATTATCCACG found at 2417159 line | 305 | CCAATTACAAGGTTTTTCCCTCCTAATCTAACCACG found at 1018456 line |
| 100 | CCAATGGTATTGTAACATCAAACTGACAAGGCCACG found at 2469635 line | 306 | CCAATGTCATGTAGTAGAAAGCCCAGAAGTCCCACG found at 1096597 line |
| 101 | CCAATGACCGGAAGGGGTACAGGCAAGGAACCCACG found at 2590668 line |  | **chromosome 16** |
|  | **chromosome 5** | 307 | CCAATGCAGCCCAAGATAGGGGGACAGGACCCCACG found at 18538 line |
| 102 | CCAATATTATCTTCATTTTGCCATCAACCTGCCACG found at 110561 line | 308 | CCAATGGCCACCCCTCACACCACAAGCCAAGCCACG found at 38775 line |
| 103 | CCAATGTTATTATCCTGAAATGGTATCATTACCACG found at 353791 line | 309 | CCAATATTTCTCAAGTTCCCTTTTCAGTATGCCACG found at 106672 line |
| 104 | CCAATCAAGGCTGCAGTGAGCTGTGATCATGCCACG found at 427546 line | 310 | CCAATTATAGCTGATTATAATGGAAGATTGTCCACG found at 128701 line |
| 105 | CCAATCTGGTACCCACCCACCCACCCACCTACCACG found at 564994 line | 311 | CCAATTCATTCACTGAAGAGTCTGGACCACTCCACG found at 131296 line |
| 106 | CCAATCATCATTTCCAAATTCATGCCAAATGCCACG found at 934240 line | 312 | CCAATCAGAGGTAACTTCAGTTTTTCATCTGCCACG found at 308703 line |
| 107 | CCAATAACTTCATCTGGGAAGATAAAACTAACCACG found at 1197026 line | 313 | CCAATCCCTGCTTTAAAATGCAGAACTAGGACCACG found at 435908 line |
| 108 | CCAATCTTAGCTCCCTAGTGACCATAATGAACCACG found at 1375717 line | 314 | CCAATACGTAATCATTCCCCTTTCCTCTCTGCCACG found at 821713 line |
| 109 | CCAATTGTAAACAACCTAAAAATCCCCTCAGCCACG found at 1460331 line | 315 | CCAATGTATAACATCATGCATTCATTCATCTCCACG found at 872904 line |
| 110 | CCAATCTGACCTTCCTGCCTTTCCTCTGTCCCCACG found at 1539808 line | 316 | CCAATTAACCATTCCCATTTCTCCGTGCCTGCCACG found at 1020479 line |
| 111 | CCAATAAAGCAGAATGGTTTGTGGAAATAACCCACG found at 1687305 line | 317 | CCAATTAACTACATTGGGTGGGGAATTGAATCCACG found at 1031882 line |
| 112 | CCAATCCCCGCCACCTGTTCCTGTTTAAGGACCACG found at 1927496 line | 318 | CCAATAGCTTGAGCAAAGAAGTCAGGTAAGCCCACG found at 1072187 line |
| 113 | CCAATAAGCTTCCTGGTTGTGAAGGTCTCTGCCACG found at 1952201 line | 319 | CCAATCAGCCACATTCTAATCCCCTTCTCACCCACG found at 1100876 line |
| 114 | CCAATGAGCATTTCCTTTGAGTGTCATGTTGCCACG found at 2221392 line | 320 | CCAATTTAAGATGCACAGGATTACCTGGTCTCCACG found at 1210500 line |
| 115 | CCAATAATTTGTAGTTGATAACTCATTCGACCCACG found at 2250230 line |  | **chromosome 17** |
| 116 | CCAATTTCTCCAAGGGGAGAGGTTTATTAACCCACG found at 2256175 line | 321 | CCAATTATGCATTCTGGCACTGGGGAAATGACCACG found at 46239 line |
| 117 | CCAATCTCTCAGAAAAACACTGCCTGAGAGTCCACG found at 2337504 line | 322 | CCAATGCACAGCTGGCTGAGCAGGCCTGCCCCCACG found at 251971 line |
|  | **chromosome 6** | 323 | CCAATTGCCTTCCACACAACAGCAAGGCCTCCCACG found at 362669 line |
| 118 | CCAATTATTAGAAACATGCATATCAGGTGTCCCACG found at 124018 line | 324 | CCAATGGGGGGGTCCACACCTCCTGTCCATGCCACG found at 585527 line |
| 119 | CCAATACATCATTTCTTTCTCCTAAGATCCTCCACG found at 186116 line | 325 | CCAATCCTGATCTTATTGCTACCACCCATTACCACG found at 623245 line |
| 120 | CCAATTACTTTGCCTCTCTGTGCCATAATTTCCACG found at 491280 line | 326 | CCAATTTCTTTCATAAGCATAGATGCAGAGTCCACG found at 637196 line |
| 121 | CCAATGCCATCAGAACTAATTAAAAACAGAGCCACG found at 707002 line | 327 | CCAATGATTTTATAAAGAGGAGTTCCCCCGGCCACG found at 687474 line |
| 122 | CCAATCACAGATGGAAGGCTAAAATGTTTGTCCACG found at 739060 line | 328 | CCAATTCAGCCACTAATACCTTTGTATACTACCACG found at 736687 line |
| 123 | CCAATTGTGTTGAGATTACAGGCATGAGCCACCACG found at 796243 line | 329 | CCAATGGAGCTGAACGTCAGCGCCTCTGTCCCCACG found at 909542 line |
| 124 | CCAATATTGCAGGGTGTATACACCCCCCACCCCACG found at 813431 line | 330 | CCAATTAGGAATGGGAGCCTTGCTCACAGCCCCACG found at 1029819 line |
| 125 | CCAATCATTCCACTTGTACTCATTTTCGACCCCACG found at 994427 line | 331 | CCAATACAAGACACAGCCGCCGCCCCGTTCTCCACG found at 1099793 line |
| 126 | CCAATGTAGCTGGAACTACAGGTGCCCACCACCACG found at 1059451 line | 332 | CCAATCAGAAGGACTTCCAATTTTCCATCTGCCACG found at 1117633 line |
| 127 | CCAATGCCTTTACAAGCGAAGGTCGGAATAACCACG found at 1179688 line |  | **chromosome 18** |
| 128 | CCAATCAGAGGTACTTTCAATATTCCATCTGCCACG found at 1546869 line | 333 | CCAATGTGATCCTCCTGTCTTCCTGGGCCCCCCACG found at 35662 line |
| 129 | CCAATTTTATCAGACAGAGTTTATCTCCATTCCACG found at 1591740 line | 334 | CCAATACTACCTATGGCTTGCCTGGGCATACCCACG found at 90728 line |
| 130 | CCAATTTCAGCCAAGTGATTAAGATCAACAACCACG found at 1670684 line | 335 | CCAATTAAACAATTTTGGACTGGGGAAATTACCACG found at 92670 line |
| 131 | CCAATTCTGGACCATGAATCTAAATCTGGATCCACG found at 1718200 line | 336 | CCAATGCAAGGCTTCAGGAACAATTCCAGAGCCACG found at 122564 line |
| 132 | CCAATGTGTATTTTCCCAGCATAAATTGGATCCACG found at 1856257 line | 337 | CCAATTTGTAGCCTGACTGGAATTGCAGATACCACG found at 335086 line |
| 133 | CCAATTATGCATTCTGGCACTGGGGAAATGACCACG found at 1958544 line | 338 | CCAATAATTTTTTTAAATTTTTAAAATTGTACCACG found at 397272 line |
| 134 | CCAATAGCTTGCTTTGTTATCCGGAGGGTAGCCACG found at 2359070 line | 339 | CCAATCTAAGGTATTATGGAAAGAGAGAGCACCACG found at 522114 line |
|  | **chromosome 7** | 340 | CCAATGTCCACTGCTGGCACGCTGGGCCAAGCCACG found at 782547 line |
| 135 | CCAATGGTCAATTCTCAGTCTTCATCTTTCTCCACG found at 248438 line | 341 | CCAATGACATGCAATTTATCCATTAATAAACCCACG found at 790408 line |
| 136 | CCAATGTCAATAGGGGACCTTTCCCCACTTTCCACG found at 388780 line | 342 | CCAATACTTCCAAATATTCAGATGTTTGATGCCACG found at 816965 line |
| 137 | CCAATGTTCCCACCACCTCCAGGTGGGCTGCCCACG found at 414032 line | 343 | CCAATGGGCACTTTGTGTGAGGGCTCCAACCCCACG found at 851279 line |
| 138 | CCAATCATTTCAAACACTCTGGAGTCAAAGTCCACG found at 777775 line | 344 | CCAATCATCTTTGTATTAGTTATTTTGCATACCACG found at 899064 line |
| 139 | CCAATCAACTTCTGCCTTGAGTGACTCCAGGCCACG found at 1043738 line | 345 | CCAATCGCCGCTGTCCAGTGCTTGGCCAGGGCCACG found at 940538 line |
| 140 | CCAATACTCTGGATAACAGGATAACAGAGTGCCACG found at 1054890 line | 346 | CCAATGGATACAATAAAGTTCAGCTTCTTAACCACG found at 957765 line |
| 141 | CCAATATCACTCTCAGGTCCGGCGCAGTGGCCCACG found at 1399769 line | 347 | CCAATCACCACACACGCCCTTTCTGACTGAACCACG found at 1029264 line |
| 142 | CCAATCTGCCTCAAACAACTGGTATTCTAAACCACG found at 1552034 line |  | **chromosome 19** |
| 143 | CCAATCCTAGATGCTGCCGTGGGGTCGGAGCCCACG found at 1738950 line | 348 | CCAATGTCGGCTTCCTAGGTCTGACGCAGCACCACG found at 22812 line |
| 144 | CCAATCTCAGAAATGACACAAAGTCACTCTGCCACG found at 1776756 line | 349 | CCAATGTAGAAGCTGCTCATCTGGCCCAGGTCCACG found at 26113 line |
| 145 | CCAATTTATGCTGTTAATCTTTCTCCCGTCTCCACG found at 1915784 line | 350 | CCAATAGGAGGGGCGAATGACTCCACTGAGGCCACG found at 34391 line |
| 146 | CCAATATCTGGCTGCCCGGCTGCCCACGCCTCCACG found at 2071873 line | 351 | CCAATGGGAGTGCGCGCACCAGGGGATGCTGCCACG found at 161016 line |
| 147 | CCAATCACTGTGCCCAGAGAAGGCAGTGGAACCACG found at 2098383 line | 352 | CCAATGTCCTACTGGTCATATAGTGAGCATCCCACG found at 180263 line |
| 148 | CCAATGCACCTCCTGGTCTGTACTTTCAAGACCACG found at 2148128 line | 353 | CCAATGACCAGCCCAAAGGCCACAGAGAAGACCACG found at 209348 line |
| 149 | CCAATTCCCAGATAGTGAACAGTCACCCACTCCACG found at 2193541 line | 354 | CCAATCAATACCTGCTGCTGGCCCCAAGGAGCCACG found at 231097 line |
|  | **chromosome 8** | 355 | CCAATGTGCATGGCTCCGATCCCAGCGGGTGCCACG found at 471400 line |
| 150 | CCAATACTGCTGGGCAACCCAGGGAGACCAGCCACG found at 19933 line | 356 | CCAATTTGCCTAAGCAAGACCTTCCGGGCATCCACG found at 554370 line |
| 151 | CCAATGGTCCGGCCTCCCTCCCTCTCCTTCCCCACG found at 84491 line | 357 | CCAATCAGAGGCACTTTCCATCTTTCATCGGCCACG found at 560411 line |
| 152 | CCAATTACACCATACCCCATGCAAGATAGCACCACG found at 153806 line | 358 | CCAATCCCCTGAGCCCTGTGCAAATCAGACACCACG found at 598097 line |
| 153 | CCAATGAAAGCATGCAGTTTCCTGAAAGCTTCCACG found at 312221 line | 359 | CCAATATTGATACATTATTATTAACTAAAGTCCACG found at 617299 line |
| 154 | CCAATCAGAGGTACTTTCAATTTTTCACCTGCCACG found at 343354 line | 360 | CCAATGATGCTAAATTCCCTTATTTTATGTCCCACG found at 640090 line |
| 155 | CCAATAGGCACTCCGTGTGGGGCCTCTGACCCCACG found at 466706 line | 361 | CCAATGACACTGCTGCCAGCCCCTGGGGGGACCACG found at 681023 line |
| 156 | CCAATTTTTAACCCCAACAACCTTTGGAGCACCACG found at 520593 line | 362 | CCAATGTTATTACAAGACCTCACACCAGCAGCCACG found at 681863 line |
| 157 | CCAATTTGTTCATCAGCTTATGACTATATGACCACG found at 551030 line | 363 | CCAATCTATGTTGTGAATGCCCAGTTGAGACCCACG found at 760708 line |
| 158 | CCAATAAGTCATAACTAAGGTTATGACTTAACCACG found at 600566 line | 364 | CCAATGTATCCCTCCCAGCCTTCTCCTCACCCCACG found at 772521 line |
| 159 | CCAATATAAGGAACAAATCCCCTGGCTCTCCCCACG found at 769938 line |  | **chromosome 20** |
| 160 | CCAATACTATTTTGGCATTTCGTAGCACCAACCACG found at 787291 line | 365 | CCAATTAAAGATGATTTTTACAGTCAATGAGCCACG found at 64818 line |
| 161 | CCAATCACCCCCTCATTAAAGTGTTTACCTACCACG found at 802757 line | 366 | CCAATCGGCGGTGCCCGCGCAGGGTGCTACGCCACG found at 324207 line |
| 162 | CCAATTCCAACCAGGGTTCATCCTCACTATTCCACG found at 976438 line | 367 | CCAATCCCTGTGACTATCTACAGTATCCACCCCACG found at 455189 line |
| 163 | CCAATGAGAATCTGCGCGCAAATGAATAAAGCCACG found at 982558 line | 368 | CCAATCTCTTCTCGTGATAATGAGGGAGTTCCCACG found at 506238 line |
| 164 | CCAATTTAAAACTTTTAGAAAGAGCCCAGCTCCACG found at 1291313 line | 369 | CCAATTCCTTTCTCACCCACCCCACTCTCAGCCACG found at 542906 line |
| 165 | CCAATTAAACAAAATATGGCAAAGCCTGAGACCACG found at 1363953 line | 370 | CCAATGCCCCAAATACTGCCCCAGCCACCAACCACG found at 658183 line |
| 166 | CCAATCAGTCTTCCAAACAAGCCATTGTGGGCCACG found at 1392307 line | 371 | CCAATAGATCCATGTGCAAACAGATCAGGGGCCACG found at 807643 line |
| 167 | CCAATCAGAGGGGTTTTCAATTTTTCATCTGCCACG found at 1590189 line | 372 | CCAATAGGACAAGTTGGCATTGCCCAGACCACCACG found at 829688 line |
| 168 | CCAATATCATTCTGAGCCATCTGGAAACCTGCCACG found at 1660813 line | 373 | CCAATCTGAGCTCTTGTCTTTTCAAACCCCACCACG found at 839697 line |
| 169 | CCAATTTCCCAGGTTCGCAGACTCCAGTTATCCACG found at 1755356 line | 374 | CCAATCTCACTTTTGCCATCCTCCAGTGTGACCACG found at 854056 line |
| 170 | CCAATGTTGCTCTAGGTTCTGTGACAGTGACCCACG found at 1854167 line |  | **chromosome 21** |
| 171 | CCAATTTTTAGAAAGTAGTATCCCACAAGGTCCACG found at 1912180 line | 375 | CCAATCCCCACCTTCCAATACCCAGTGCCCTCCACG found at 211682 line |
| 172 | CCAATGTAGCTGGGAGTACAGGCACCCGCCACCACG found at 1970658 line | 376 | CCAATCCCCACCTTCCAATACCCAGTGCCCTCCACG found at 211954 line |
| 173 | CCAATCCCTCGCCACGGCAGCCTCTGTCCTCCCACG found at 1980933 line | 377 | CCAATCCATTGGTCATTCTGTCATTTTTTCACCACG found at 250879 line |
|  | **chromosome 9** | 378 | CCAATTCCGGACACAGTATGATAACCCCTAGCCACG found at 350416 line |
| 174 | CCAATGAAGCCATCTAAACTTGGGCTTTTTTCCACG found at 15433 line | 379 | CCAATTATACTCCTTTCCCTCCTTGGCCTTCCCACG found at 601869 line |
| 175 | CCAATAAAACAAAAGAAGTAATTCTATGAACCCACG found at 37297 line | 380 | CCAATTCCCATCCCCCAGCAGCGTGTTAGCGCCACG found at 656350 line |
| 176 | CCAATGCTGAAGACTGACGAATGTCAACTGGCCACG found at 39610 line |  | **chromosome 22** |
| 177 | CCAATTTTATCATCCATTAGAATGAGTTCTCCCACG found at 225838 line | 381 | CCAATCTCCAAGCACTGCTTGGCATCCGCGGCCACG found at 260815 line |
| 178 | CCAATAATCTTTTCTCCTGAAAGCACTGTCACCACG found at 311732 line | 382 | CCAATGTAGAAGCTGCTCATCTGGCTCAGGTCCACG found at 312113 line |
| 179 | CCAATAATTCTAAATGTTTCTTCTAACAATCCCACG found at 486234 line | 383 | CCAATACCCATCTTTGACTCATCACAGTCTTCCACG found at 389934 line |
| 180 | CCAATGCTCCTGGGGCCTCCCTCCCCCCCGCCCACG found at 614377 line | 384 | CCAATGTCCCCGGGACCAGCTCTTTCTCTGGCCACG found at 390219 line |
| 181 | CCAATAGAAAATATATGTATCAAGCAATTTCCCACG found at 1008405 line | 385 | CCAATATCTTTAGCTGGGAATGGAAATATCCCCACG found at 437878 line |
| 182 | CCAATGCCGTCTACTGGGCTGCTCGGCATGGCCACG found at 1253644 line | 386 | CCAATTGGAAGGGGCTTTGCCATCTGTCTCACCACG found at 643920 line |
| 183 | CCAATTAAGAACATGGCACAGCTCCAAGCCTCCACG found at 1275999 line | 387 | CCAATCAGAGGTACTTTCAGTTTTTCATCTGCCACG found at 656855 line |
| 184 | CCAATTCCACATGTAGTGAGCTCTATCCTTACCACG found at 1287773 line | 388 | CCAATTCTCCTGGCGTTTAGAAGACAAATCACCACG found at 657876 line |
| 185 | CCAATGACTCTCCTCTGGAGTGATAAAGAGTCCACG found at 1288815 line | 389 | CCAATGACAGATACACCGTCTGCTCAGGTCACCACG found at 692807 line |
| 186 | CCAATGAGAACCTGGGGCTGACCCTCAAAGGCCACG found at 1326249 line |  | **chromosome X** |
| 187 | CCAATTCCCGCTGTGCCTGCCTCCCACCGTCCCACG found at 1330585 line | 390 | CCAATGGCTTTGAAATTTTTCTATAAGGAAGCCACG found at 151130 line |
| 188 | CCAATTCGTGTGCCAGAGTAAATATGTTCGTCCACG found at 1521096 line | 391 | CCAATCTGGTGGCTCTTTTAGCCGACTTTAGCCACG found at 176747 line |
| 189 | CCAATATATTTCTTGTTGTATCATGATAGCACCACG found at 1535787 line | 392 | CCAATGTGTGTGAAAGGTGTGCTGGCCATGGCCACG found at 252747 line |
| 190 | CCAATGTCATTGATGGGCATTTATGTTAACTCCACG found at 1573567 line | 393 | CCAATAGGCTGCACAATCTGAAATGTCATGCCCACG found at 264174 line |
| 191 | CCAATAATCTGAAGCTTCAGAAGTCACAGTTCCACG found at 1621746 line | 394 | CCAATTATGCATTGTGGCACTAGGAAAATGACCACG found at 888351 line |
| 192 | CCAATCTTATGTAAACAGAGCCTCTTAACGTCCACG found at 1623674 line | 395 | CCAATTAAAAAAAAAAAACAAATCATAGCAACCACG found at 896351 line |
| 193 | CCAATATAGTACAATTTTATTACCTATAGGCCCACG found at 1638306 line | 396 | CCAATAAGCCTTTACTAAGGTTATAACTTAACCACG found at 896603 line |
| 194 | CCAATGAGTCAGTGGCCCTGGAGTGCCAGAGCCACG found at 1850559 line | 397 | CCAATCCATTTTCAGGCCTGACTTTTGTGAGCCACG found at 952534 line |
| 195 | CCAATGGCCACTAAAATCATCAGAGCTGTGCCCACG found at 1892093 line | 398 | CCAATTATGCATTCTGGAACTTGGACAATAACCACG found at 1022049 line |
| 196 | CCAATCTGGCTTCACGGCGGCGGAACTGACGCCACG found at 1896114 line | 399 | CCAATTACATAAACAGAATCAATGACAAAAACCACG found at 1216213 line |
| 197 | CCAATAATTCCAGCCACACAGCGTCAGGGAGCCACG found at 1943747 line | 400 | CCAATGACTGTAAAATGAAGGGATACATAGGCCACG found at 1254606 line |
|  | **chromosome 10** | 401 | CCAATTATGCATTCTGGCACTGGGGAAATGACCACG found at 1491516 line |
| 198 | CCAATCGATGTTGTGAGCGATATAGCATGAACCACG found at 45103 line | 402 | CCAATAGTTGGTTGTTTGAAAGGATAACAAACCACG found at 1519845 line |
| 199 | CCAATGTATTTTGCTTGTTAATGTCATTTACCCACG found at 49227 line | 403 | CCAATCGACTGAACCCAACTTATAATCAAACCCACG found at 1613966 line |
| 200 | CCAATGTTGCCTTAGCTGGACGATAGAGTGGCCACG found at 89718 line | 404 | CCAATCATAGGTACTTTCAATTTCCCATCGGCCACG found at 1789596 line |
| 201 | CCAATTGCTCTTCACTCCATTCCTGACCCTTCCACG found at 149469 line | 405 | CCAATTAATTAAAAATATCTGAGGGTGGGACCCACG found at 2033418 line |
| 202 | CCAATGGACTGGTTCAGCAGCAAGAACATGGCCACG found at 199186 line | 406 | CCAATCCCTAGGACAAATATGGTTTTGGGCTCCACG found at 2116358 line |
| 203 | CCAATGAGGCAAGAATAGTGCCAAGGTTCAGCCACG found at 264191 line |  | **chromosome Y** |
| 204 | CCAATCGATGCACACAGAAAACTCCTCTGGGCCACG found at 368128 line | 407 | CCAATGACTTACCAATTACTCACTGATTTGACCACG found at 112113 line |
| 205 | CCAATTCTCCCTTCCTCCCATCTCCTGGCAACCACG found at 384087 line | 408 | CCAATATTCCCTCTTTGATGGGGAAAAATGGCCACG found at 353943 line |
| 206 | CCAATGGTGGTTCAGAGAGGAGCCTCTAAACCCACG found at 390744 line | 409 | CCAATATTCCCTCTTTGATGGGGAAAAATGGCCACG found at 376382 line |
| 207 | CCAATGAGATGTGAGCATCTATTATCGTGCCCCACG found at 395425 line | 410 | CCAATGACTTACTGATTAGTCACTGACTTCACCACG found at 378416 line |
| 208 | CCAATTACATCTGTGGCCCAGGCAAGGGAAGCCACG found at 413565 line | 411 | CCAATTTAAATCCAATAGCTTTTCCACACCTCCACG found at 399467 line |
