# Supplementary material for: Identification of ASB7 as ER stress responsive gene through a genome wide in silico screening for genes with ERSE
Source: PLoS One. 2018 Apr 9;13(4):e0194310. doi: 10.1371/journal.pone.0194310 (PMC5890977; doi:10.1371/journal.pone.0194310)
Supplement: S2 Table — (DOCX) [file pone.0194310.s002.docx]

**S2 Table:**

Detailed information about the gene, gene name, ERSE element location, gene location and distance between the ERSE element and gene.

| **SNO** | **ERSE element** | **Gene** | **gene ID** | **Gene name** | **Hit position** | **Gene position** | **10 Kb** | **20 Kb** | **Remark** |
| --- | --- | --- | --- | --- | --- | --- | --- | --- | --- |
| **ERSE-I** | | | | | | | | | |
|  | chromosome 1 |  |  |  |  |  |  |  |  |
| 1 | CCAATCCCCGGGCACCACG found at 16172 line | B3GALT6 | [126792 (B3GALT6)](http://www.ncbi.nlm.nih.gov/gene/126792) | UDP-Gal:betaGal beta 1,3-galactosyltransferase polypeptide 6 | 1,164,255 to 1,164,273 | 1,167,629..1,170,421 | 3374 |  |  |
| 2 | CCAATTGTAATCCCCCACG found at 49113 line | SESN2 | ID: 83667 | sestrin 2 | 28525807 .. 28525825 | 28,585,963..28,609,002 |  |  | 60,156 |
| 3 | CCAATTGTAATCCCCCACG found at 396193 line | TPRG1L | [127262 (TPRG1L)](http://www.ncbi.nlm.nih.gov/gene/127262) | tumor protein p63 regulated 1-like | 3536037 .. 3536055 | 3,541,556..3,546,695 | 5519 |  |  |
| 4 | CCAATATGGTGAAACCACG found at 56437 line | DARS2 | ID: 55157 | aspartyl-tRNA synthetase 2, mitochondrial | 173812508 .. 173812526 | 173,793,719..173,827,682 |  |  | element present inside gene |
| 5 | CCAATATGGTGAAACCACG found at 356291 line | TMEM50A | ID: 23585 | transmembrane protein 50A | 25652803 .. 25652992 | 25,664,789..25,688,852 |  | 11989 |  |
| 6 | CCAATATGGTGAAACCACG found at 2414064 line | AJAP1 | ID: 55966 | adherens junctions associated protein 1 | 4063310 .. 4063328 | 4,715,105..4,843,851 |  |  | 651,795 |
| 7 | CCAATTTGCCTCATCCACG found at 64905 line | AJAP1 | ID: 55966 | adherens junctions associated protein 1 | 4673036 .. 4673054 | 4,715,105..4,843,851 |  |  | 42,069 |
| 8 | CCAATATGGGGAAACCACG found at 72760 line | 0 |  |  |  |  |  |  |  |
| 9 | CCAATCGTGGTGGTCCACG found at 180521 line | PRAMEF25 | ID: 441873 |  | 13107744 .. 13107762 | 13,140,447..13,145,000 |  |  | 32703 |
| 10 | CCAATCGTGGTGGTCCACG found at 182054 line | PRAMEF25 | ID: 441873 |  | 12997411 .. 12997429 | 13,140,447..13,145,000 |  |  | 143036 |
| 11 | CCAATATGCTTGTCCCACG found at 232552 line | NECAP2 | ID: 55707 | NECAP endocytosis associated 2 | 16743632 .. 16743650 | 16,767,167..16,786,585 |  |  | 23535 |
| 12 | CCAATCATGATGGCCCACG found at 265831 line | 0 |  |  |  |  |  |  |  |
| 13 | CCAATATGAACCATCCACG found at 703891 line | 0 |  |  |  |  |  |  |  |
| 14 | CCAATGAAGTTTGTCCACG found at 937151 line | 0 |  |  |  |  |  |  |  |
| 15 | CCAATCCCTTATTTCCACG found at 986878 line | 0 |  |  |  |  |  |  |  |
| 16 | CCAATCCCTTATTTCCACG found at 1134892 line | 0 |  |  |  |  |  |  |  |
| 17 | CCAATTTCAAACTTCCACG found at 1033004 line | 0 |  |  |  |  |  |  |  |
| 18 | CCAATCACCTTCTACCACG found at 1079206 line | AK5 | ID: 26289 | adenylate kinase 5 | 77702692 .. 77703499 | 77,747,662..78,025,654 |  |  | 44970 |
| 19 | CCAATTCATACTGGCCACG found at 1279476 line | 0 |  |  |  |  |  |  |  |
| 20 | CCAATTCTTTCGTTCCACG found at 1336222 line | 0 |  |  |  |  |  |  |  |
| 21 | CCAATTGAAGTGGTCCACG found at 1536971 line | SLC6A17 | ID: 388662 | solute carrier family 6 (neutral amino acid transporter), member 17 | 110661803 .. 110661821 | 110,693,132..110,744,824 |  |  | 31329 |
| 22 | CCAATGCAGGCAGACCACG found at 1677117 line | 0 |  |  |  |  |  |  |  |
| 23 | CCAATACCCCAGAGCCACG found at 2026934 line | 0 |  |  |  |  |  |  |  |
| 24 | CCAATACCCCAGAGCCACG found at 2048347 line | 0 |  |  |  |  |  |  |  |
| 25 | CCAATGAAATAAAGCCACG found at 2252455 line | NOS1AP | ID: 9722 | nitric oxide synthase 1 (neuronal) adaptor protein | 162176667 .. 162176685 | 162,039,581..162,339,813 |  |  | element present inside the gene |
| 26 | CCAATCAGGCTCTGCCACG found at 2299551 line | MGST3 | ID: 4259 | microsomal glutathione S-transferase 3 | 165567545 .. 165567563 | 165,600,110..165,625,373 |  |  | 32565 |
| 27 | CCAATTCTTTCTGGCCACG found at 2392948 line | DNM3 | ID: 26052 | dynamin 3 | 172292140 .. 172292158 | 171,810,618..172,387,606 |  |  | element present inside the gene |
| 28 | CCAATATCCTAAAGCCACG found at 2438948 line | 0 |  |  |  |  |  |  |  |
| 29 | CCAATCTCCTAAAACCACG found at 2456123 line | 0 |  |  |  |  |  |  |  |
| 30 | CCAATTAGAGACATCCACG found at 2494728 line | TDRD5 | ID: 163589 | tudor domain containing 5 | 179620304 .. 179620322 | 179,560,748..179,660,407 |  |  | element present inside the gene |
| 31 | CCAATATGCTGAAACCACG found at 2699696 line | 0 |  |  |  |  |  |  |  |
| 32 | CCAATGCACACCACCCACG found at 2793800 line | IGFN1 | [91156 (IGFN1)](http://www.ncbi.nlm.nih.gov/gene/91156) | immunoglobulin-like and fibronectin type III domain containing 1 | 201153485 .. 201153503 | 201,159,952..201,198,080 | 6467 |  |  |
| 33 | CCAATCACCTCCCACCACG found at 3098804 line | DISP1 | ID: 84976 | dispatched homolog 1 (Drosophila) | 223113760 .. 223113778 | 222,988,342..223,179,337 |  |  | element present inside the gene |
| 34 | CCAATGTGAAAAGGCCACG found at 3114385 line | 0 |  |  |  |  |  |  |  |
| 35 | CCAATGGCCAGGCACCACG found at 3140051 line | 0 |  |  |  |  |  |  |  |
| 36 | CCAATCTCCTGGTGCCACG found at 3150193 line | 0 |  |  |  |  |  |  |  |
| 37 | CCAATACTCAGTTCCCACG found at 3201884 line | 0 |  |  |  |  |  |  |  |
| 38 | CCAATGCACACTGGCCACG found at 3255058 line | SLC35F3 | ID: 148641 | solute carrier family 35, member F3 | 234364078 .. 234364096 | 234,040,679..234,460,262 |  |  | element present inside the gene |
| 39 | CCAATGAGGCGGGACCACG found at 3283967 line | 0 |  |  |  |  |  |  |  |
| 40 | CCAATTAACCAAAGCCACG found at 3305944 line | 0 |  |  |  |  |  |  |  |
| 41 | CCAATCAGCATTCTCCACG found at 3309770 line | 0 |  |  |  |  |  |  |  |
| 42 | CCAATAGGTAGGGACCACG found at 3371703 line | 0 |  |  |  |  |  |  |  |
| 43 | CCAATCATGTCTCACCACG found at 3374753 line | 0 |  |  |  |  |  |  |  |
| 44 | CCAATAAAAAAGTACCACG found at 3431003 line | 0 |  |  |  |  |  |  |  |
|  | Chromosome 2 |  |  |  |  |  |  |  |  |
| 45 | CCAATACAAACATCCCACG found at 23996 line | 0 |  |  |  |  |  |  |  |
| 46 | CCAATGCTGTCCGCCCACG found at 31324 line | 0 |  |  |  |  |  |  |  |
| 47 | CCAATCCCTAGGACCCACG found at 34017 line | 0 |  |  |  |  |  |  |  |
| 48 | CCAATTTCTTCCTTCCACG found at 39280 line | 0 |  |  |  |  |  |  |  |
| 49 | CCAATATGGTAAAACCACG found at 73878 line | 0 |  |  |  |  |  |  |  |
| 50 | CCAATAGACTTGTTCCACG found at 90898 line | 0 |  |  |  |  |  |  |  |
| 51 | CCAATCACCTCCCACCACG found at 119777 line | 0 |  |  |  |  |  |  |  |
| 52 | CCAATTTGGACAAGCCACG found at 165492 line | LPIN1 | ID: 23175 | lipin 1 | 11915273 .. 11915291 | 11,817,705..11,967,535 |  |  | element present inside the gene |
| 53 | CCAATCCCTTATTTCCACG found at 177330 line | 0 |  |  |  |  |  |  |  |
| 54 | CCAATCCCTTATTTCCACG found at 938333 line | 0 |  |  |  |  |  |  |  |
| 55 | CCAATCCCTTATTTCCACG found at 1073842 line | ETAA1 | ID: 54465 | Ewing tumor-associated antigen 1 | 67559823 .. 67559841 | 67,624,442..67,637,540 |  |  | 64601 |
| 56 | CCAATCCCTTATTTCCACG found at 3127259 line | TRIB2 | ID: 28951 | tribbles homolog 2 (Drosophila) | 12767654 .. 12767672 | 12,856,998..12,882,860 |  |  | 89326 |
| 57 | CCAATTAAAGGACACCACG found at 244929 line | VSNL1 | ID: 7447 | visinin-like 1 | 17634766 .. 17634784 | 17,721,807..17,837,706 |  |  | 87023 |
| 58 | CCAATGTCATGCTTCCACG found at 248544 line | GEN1 | ID: 348654 | GEN1 Holliday junction 5' flap endonuclease | 17895054 .. 17895072 | 17,935,152..17,966,632 |  |  | 40080 |
| 59 | CCAATGTGGTGAAACCACG found at 344343 line | NCOA1 | ID: 8648 | nuclear receptor coactivator 1 | 24792550 .. 24792568 | 24,714,919..24,993,571 |  |  | element present inside the gene |
| 60 | CCAATAAATGCTATCCACG found at 374363 line | KCNK3 | ID: 3777 | potassium channel, subfamily K, member 3 | 26954039 .. 26954057 | 26,915,581..26,954,066 |  |  | element present inside the gene |
| 61 | CCAATGGGAGACAACCACG found at 491805 line | 0 |  |  |  |  |  |  |  |
| 62 | CCAATTGTACATGACCACG found at 746785 line | 0 |  |  |  |  |  |  |  |
| 63 | CCAATCGCTTATTTCCACG found at 895568 line | 0 |  |  |  |  |  |  |  |
| 64 | CCAATCTCTCCTGCCCACG found at 912704 line | 0 |  |  |  |  |  |  |  |
| 65 | CCAATAATGTGATACCACG found at 989408 line | NAGK | ID: 55577 | N-acetylglucosamine kinase | 71237289 .. 71237307 | 71,295,408..71,305,998 |  |  | 58101 |
| 66 | CCAATCAGGGTTGCCCACG found at 1434480 line | SLC9A2 | ID: 6549 | solute carrier family 9, subfamily A (NHE2, cation proton antiporter 2), member 2 | 103282439 .. 103282457 | 103,236,166..103,327,809 |  |  | element present inside the gene |
| 67 | CCAATTTTCACACACCACG found at 1645507 line | 0 |  |  |  |  |  |  |  |
| 68 | CCAATGCTCACAGCCCACG found at 1664456 line | 0 |  |  |  |  |  |  |  |
| 69 | CCAATCACACTGGGCCACG found at 1666378 line | STEAP3 | [55240 (STEAP3)](http://www.ncbi.nlm.nih.gov/gene/55240) | STEAP family member 3, metalloreductase | 119979088 .. 119979106 | 119,981,384..120,023,228 | 2296 |  |  |
| 70 | CCAATGCAGAAAAGCCACG found at 1710564 line | 0 |  |  |  |  |  |  |  |
| 71 | CCAATTCTCTCCATCCACG found at 1782842 line | MYO7B | ID: 4648 | myosin VIIB | 128364497 .. 128364515 | 128,293,378..128,395,303 |  |  | element present inside the gene |
| 72 | CCAATCATCACCCACCACG found at 1818698 line | MZT2B | ID: 80097 | mitotic spindle organizing protein 2B | 130946100 .. 130946118 | 130,939,248..130,962,365 |  |  | element present inside the gene |
| 73 | CCAATCCAAGGAAGCCACG found at 1938262 line | 0 |  |  |  |  |  |  |  |
| 74 | CCAATGATGTGCACCCACG found at 2218461 line | TANC1 | ID: 85461 | tetratricopeptide repeat, ankyrin repeat and coiled-coil containing 1 | 159729079 .. 159729097 | 159,825,146..160,089,170 |  |  | 96067 |
| 75 | CCAATACCCGGCAGCCACG found at 2346017 line | 0 |  |  |  |  |  |  |  |
| 76 | CCAATATGGTGAAACCACG found at 2425248 line | 0 |  |  |  |  |  |  |  |
| 77 | CCAATCTCCAACTCCCACG found at 2528141 line | 0 |  |  |  |  |  |  |  |
| 78 | CCAATTGAGGAGTGCCACG found at 2665202 line | 0 |  |  |  |  |  |  |  |
| 79 | CCAATCATTGTGAACCACG found at 2950234 line | 0 |  |  |  |  |  |  |  |
| 80 | CCAATTCACTGAGACCACG found at 3109031 line | 0 |  |  |  |  |  |  |  |
| 81 | CCAATCATCTTCCACCACG found at 3294212 line | 0 |  |  |  |  |  |  |  |
| 82 | CCAATTCACGAAACCCACG found at 3295000 line | 0 |  |  |  |  |  |  |  |
| 83 | CCAATGCACTTTAGCCACG found at 3367898 line | BOK | [666 (BOK)](http://www.ncbi.nlm.nih.gov/gene/666) | BCL2-related ovarian killer | 242488511 .. 242488529 | 242,498,146..242,513,553 | 9635 |  |  |
|  | chromosome 3 |  |  |  |  |  |  |  |  |
| 84 | CCAATTTCTGAATCCCACG found at 24609 line | RPL23AP39 | [100271461 (RPL23AP39)](http://www.ncbi.nlm.nih.gov/gene/100271461) | ribosomal protein L23a pseudogene 39 | 1771696 .. 1771714 | 1,771,738..1,772,158 | 42 |  |  |
| 85 | CCAATTTCTTTCTTCCACG found at 36316 line | CNTN4 | ID: 152330 | contactin 4 | 2614636 .. 2614654 | 2,140,550..3,099,645 |  |  | element present inside the gene |
| 86 | CCAATGGTTTGACACCACG found at 107178 line | GRM7 | ID: 2917 | glutamate receptor, metabotropic 7 | 7716693 .. 7716711 | 6,902,802..7,783,218 |  |  | element present inside the gene |
| 87 | CCAATTTCCCCACTCCACG found at 110205 line | 0 |  |  |  |  |  |  |  |
| 88 | CCAATCCCTTATTTCCACG found at 114165 line | 0 |  |  |  |  |  |  |  |
| 89 | CCAATCCCTTATTTCCACG found at 563940 line | ENPP7P4 (pseudo) | ID: 2917 | ectonucleotide pyrophosphatase/phosphodiesterase 7 pseudogene 4 | 125583594 .. 125583612 | 125,567,066..125,628,257 |  |  | element present inside the gene |
| 90 | CCAATCCCTTATTTCCACG found at 1744218 line | 0 |  |  |  |  |  |  |  |
| 91 | CCAATCCCTTATTTCCACG found at 2636994 line | 0 |  |  |  |  |  |  |  |
| 92 | CCAATGGCTGGCAACCACG found at 295311 line | 0 |  |  |  |  |  |  |  |
| 93 | CCAATGCCCAGAAACCACG found at 311293 line | 0 |  |  |  |  |  |  |  |
| 94 | CCAATCGCCTCCCACCACG found at 317528 line | 0 |  |  |  |  |  |  |  |
| 95 | CCAATATGGTGAAACCACG found at 461775 line | 0 |  |  |  |  |  |  |  |
| 96 | CCAATGATCTTAACCCACG found at 777377 line | 0 |  |  |  |  |  |  |  |
| 97 | CCAATTGCATGTTTCCACG found at 793542 line | 0 |  |  |  |  |  |  |  |
| 98 | CCAATCTAGTTTCACCACG found at 964737 line | 0 |  |  |  |  |  |  |  |
| 99 | CCAATCAAAAATTACCACG found at 977466 line | 0 |  |  |  |  |  |  |  |
| 100 | CCAATCCACCGAATCCACG found at 1010162 line | 0 |  |  |  |  |  |  |  |
| 101 | CCAATAAGAGTAAGCCACG found at 1051577 line | LOC100128827 (pseudo) | [100128827 (LOC100128827)](http://www.ncbi.nlm.nih.gov/gene/100128827) | FSHD region gene 2 pseudogene | 197836918 .. 197836936 | 197,837,077..197,838,741 | 159 |  |  |
| 102 | CCAATAAGAGTAAGCCACG found at 2747737 line | FRG2C | [100288801 (FRG2C)](http://www.ncbi.nlm.nih.gov/gene/100288801) | FSHD region gene 2 family, member C | 75713416 .. 75713434 | 75,713,487..75,716,368 | 71 |  |  |
| 103 | CCAATTCGCTGTCTCCACG found at 2087009 line | EIF2A | ID: 83939 | eukaryotic translation initiation factor 2A, 65kDa | 150264497 .. 150264515 | 150,264,465..150,303,803 |  |  | element present inside the gene |
| 104 | CCAATGAAGAATACCCACG found at 2093786 line | CLRN1-AS1 (pseudo) | ID: 116933 | CLRN1 antisense RNA 1 | 150752487 .. 150752505 | 150,690,465..150,797,617 |  |  | element present inside pseudo gene |
| 105 | CCAATATGATGAAACCACG found at 2456361 line | 0 |  |  |  |  |  |  |  |
| 106 | CCAATCACTCCTCACCACG found at 2477008 line | KCNMB2 | ID: 10242 | potassium large conductance calcium-activated channel, subfamily M, beta member 2 | 178344484 .. 178344502 | 178,254,086..178,562,217 |  |  | element present inside the gene |
| 107 | CCAATGCCTAGTTTCCACG found at 2533039 line | 0 |  |  |  |  |  |  |  |
| 108 | CCAATAGATCATCCCCACG found at 2602376 line | 0 |  |  |  |  |  |  |  |
| 109 | CCAATAAAAAATGACCACG found at 2689927 line | 0 |  |  |  |  |  |  |  |
| 110 | CCAATTATTTAAGCCCACG found at 2690767 line | 0 |  |  |  |  |  |  |  |
|  | chromosome 4 |  |  |  |  |  |  |  |  |
| 111 | CCAATTAGCGCACACCACG found at 16122 line | 0 |  |  |  |  |  |  |  |
| 112 | CCAATCTCAACATGCCACG found at 104858 line | SORCS2 | ID: 57537 | sortilin-related VPS10 domain containing receptor 2 | 7549649 .. 7549667 | 7,194,374..7,744,564 |  |  | element present inside the gene |
| 113 | CCAATGTCAGCTGGCCACG found at 112300 line | 0 |  |  |  |  |  |  |  |
| 114 | CCAATTCAAGCCCTCCACG found at 155852 line | 0 |  |  |  |  |  |  |  |
| 115 | CCAATGGTTGGACCCCACG found at 240576 line | 0 |  |  |  |  |  |  |  |
| 116 | CCAATCACCTCCCACCACG found at 240991 line | 0 |  |  |  |  |  |  |  |
| 117 | CCAATCACCTCCCACCACG found at 1181928 line | 0 |  |  |  |  |  |  |  |
| 118 | CCAATTGCAGTTTCCCACG found at 335454 line | 0 |  |  |  |  |  |  |  |
| 119 | CCAATGTCTTAAAACCACG found at 482543 line | 0 |  |  |  |  |  |  |  |
| 120 | CCAATCGCCTTCCACCACG found at 486871 line | 0 |  |  |  |  |  |  |  |
| 121 | CCAATTTTCTGGGTCCACG found at 508997 line | 0 |  |  |  |  |  |  |  |
| 122 | CCAATATGGTGAAACCACG found at 590412 line | 0 |  |  |  |  |  |  |  |
| 123 | CCAATGTGGGCAAACCACG found at 664746 line | 0 |  |  |  |  |  |  |  |
| 124 | CCAATGACCAGGCCCCACG found at 735027 line | SPATA18 | ID: 132671 | spermatogenesis associated 18 | 52921840 .. 52921858 | 52,917,578..52,963,461 |  |  | element present inside the gene |
| 125 | CCAATCCCCCCACCCCACG found at 773111 line | 0 |  |  |  |  |  |  |  |
| 126 | CCAATTTATTGAAGCCACG found at 865498 line | LPHN3 | ID: 23284 | latrophilin 3 | 62315747 .. 62315765 | 62,066,949..62,938,168 |  |  | element present inside the gene |
| 127 | CCAATCACCTCTCACCACG found at 886556 line | 0 |  |  |  |  |  |  |  |
| 128 | CCAATTACCTCCCACCACG found at 997210 line | MOB1B | ID: 92597 | MOB kinase activator 1B | 71798973 .. 71798991 | 71,768,043..71,853,891 |  |  | element present inside the gene |
| 129 | CCAATGAAATCAGGCCACG found at 1052710 line | 0 |  |  |  |  |  |  |  |
| 130 | CCAATCAGCCCCTGCCACG found at 1380254 line | 0 |  |  |  |  |  |  |  |
| 131 | CCAATTCTTCATCTCCACG found at 1400974 line | 0 |  |  |  |  |  |  |  |
| 132 | CCAATATTTTAAGTCCACG found at 1505191 line | 0 |  |  |  |  |  |  |  |
| 133 | CCAATTTCCTCTTTCCACG found at 1856812 line | 0 |  |  |  |  |  |  |  |
| 134 | CCAATGCTGAGTTGCCACG found at 2142291 line | TRIM2 | ID: 23321 | tripartite motif containing 2 | 154244836 .. 154244854 | 154,073,650..154,260,474 |  |  | element present inside the gene |
|  | chromosome 5 |  |  |  |  |  |  |  |  |
| 135 | CCAATCCCTTATTTCCACG found at 100901 line | ATG10 | ID: 83734 | autophagy related 10 | 81250126 .. 81250144 | 81,267,844..81,551,958 |  | 17718 |  |
| 136 | CCAATCCCTTATTTCCACG found at 228074 line | IQGAP2 | ID: 10788 | IQ motif containing GTPase activating protein 2 | 75654171 .. 75654189 | 75,699,131..76,003,957 |  |  | 44960 |
| 137 | CCAATCCCTTATTTCCACG found at 257440 line | 0 |  |  |  |  |  |  |  |
| 138 | CCAATCCCTTATTTCCACG found at 1050754 line | 0 |  |  |  |  |  |  |  |
| 139 | CCAATCCCTTATTTCCACG found at 1128476 line | 0 |  |  |  |  |  |  |  |
| 140 | CCAATCACCAATCACCACG found at 153979 line | 0 |  |  |  |  |  |  |  |
| 141 | CCAATTACACTTCTCCACG found at 207224 line | 0 |  |  |  |  |  |  |  |
| 142 | CCAATCTCCTGTCTCCACG found at 229484 line | 0 |  |  |  |  |  |  |  |
| 143 | CCAATCCCCGCACCCCACG found at 283517 line | 0 |  |  |  |  |  |  |  |
| 144 | CCAATGTGGCAAAACCACG found at 761665 line | 0 |  |  |  |  |  |  |  |
| 145 | CCAATTTCTACTTCCCACG found at 842995 line | ZSWIM6 | ID: 57688 | zinc finger, SWIM-type containing 6 | 60695552 .. 60695570 | 60,628,100..60,841,999 |  |  | element present inside the gene |
| 146 | CCAATCCTCCACTCCCACG found at 948416 line | 0 |  |  |  |  |  |  |  |
| 147 | CCAATAAACACTCTCCACG found at 1026652 line | HEXB | ID: 3074 | hexosaminidase B (beta polypeptide) | 73918799 .. 73918817 | 73,935,547..74,017,113 |  | 16748 |  |
| 148 | CCAATTGGTCATGTCCACG found at 1118452 line | CKMT2 | [1160 (CKMT2)](http://www.ncbi.nlm.nih.gov/gene/1160) | creatine kinase, mitochondrial 2 (sarcomeric) | 80528392 .. 80528410 | 80,528,605..80,562,217 | 213 |  |  |
| 149 | CCAATCCCCTTGATCCACG found at 1553702 line | 0 |  |  |  |  |  |  |  |
| 150 | CCAATATGGTGAAACCACG found at 1588918 line | 0 |  |  |  |  |  |  |  |
| 151 | CCAATCACCTCTCCCCACG found at 1808354 line | 0 |  |  |  |  |  |  |  |
| 152 | CCAATGCCCCTTCCCCACG found at 1920323 line | CTNNA1 | ID: 1495 | catenin (cadherin-associated protein), alpha 1, 102kDa | 138263124 .. 138263142 | 138,089,075..138,270,723 |  |  | element present inside the gene |
| 153 | CCAATCTCAAATTTCCACG found at 1924081 line | 0 |  |  |  |  |  |  |  |
| 154 | CCAATGAATGTTTTCCACG found at 1954587 line | PCDHGA1 | ID: 56114 | protocadherin gamma subfamily A, 1 | 140730155 .. 140730173 | 140,709,388..140,892,546 |  |  | element present inside the gene |
|  |  | PCDHG@ | ID: 56115 | protocadherin gamma cluster | 140730155 .. 140730173 | 140,710,252..140,892,546 |  |  | element present inside the gene |
|  |  | PCDHGA2 | ID: 56113 | protocadherin gamma subfamily A, 2 | 140730155 .. 140730173 | 140,718,354..140,892,546 |  |  | element present inside the gene |
|  |  | PCDHGB1 | ID: 56104 | protocadherin gamma subfamily B, 1 | 140730155 .. 140730173 | 140,729,828..140,892,546 |  |  | element present inside the gene |
|  |  | PCDHGA3 | ID: 56112 | protocadherin gamma subfamily A, 3 | 140730155 .. 140730173 | 140,723,601..140,892,546 |  |  | element present inside the gene |
|  |  | PCDHGA4 | [56111 (PCDHGA4)](http://www.ncbi.nlm.nih.gov/gene/56111) | protocadherin gamma subfamily A, 4 | 140730155 .. 140730173 | 140,734,768..140,892,546 | 4613 |  |  |
|  |  | PCDHGB2 | [56103 (PCDHGB2)](http://www.ncbi.nlm.nih.gov/gene/56103) | protocadherin gamma subfamily B, 2 | 140730155 .. 140730173 | 140,739,409..140,892,546 | 9254 |  |  |
|  |  | PCDHGA5 | ID: 56110 | protocadherin gamma subfamily A, 5 | 140730155 .. 140730173 | 140,743,729..140,892,546 |  | 13574 |  |
|  |  | PCDHGA6 | ID: 56109 | protocadherin gamma subfamily A, 6 | 140730155 .. 140730173 | 140,749,811..140,892,546 |  | 19656 |  |
|  |  | PCDHGB3 | ID: 56102 | protocadherin gamma subfamily B, 3 | 140730155 .. 140730173 | 140,749,962..140,892,546 |  | 19807 |  |
| 155 | CCAATCACCTCCCACCACG found at 1959773 line | 0 |  |  |  |  |  |  |  |
| 156 | CCAATAGATAAGTGCCACG found at 2054828 line | 0 |  |  |  |  |  |  |  |
| 157 | CCAATGAATGGTAGCCACG found at 2193684 line | 0 |  |  |  |  |  |  |  |
| 158 | CCAATCCAATACTTCCACG found at 2272392 line | 0 |  |  |  |  |  |  |  |
| 159 | CCAATCTTCACACTCCACG found at 2487860 line | CANX | ID: 821 | calnexin | 179125779 .. 179125797 | 179,125,019..179,158,642 |  |  | element present inside the gene |
| 160 | CCAATCACCTCCCTCCACG found at 2506444 line | BTNL9 | [153579 (BTNL9)](http://www.ncbi.nlm.nih.gov/gene/153579) | butyrophilin-like 9 | 180463852 .. 180463870 | 180,467,203..180,488,523 | 3351 |  |  |
|  | chromosome 6 |  |  |  |  |  |  |  |  |
| 161 | CCAATCCCTAGGTACCACG found at 70620 line | PPP1R3G | 648791 | protein phosphatase 1, regulatory subunit 3G | 5084500 .. 5084518 | 5,085,720..5,087,455 | 1220 |  |  |
| 162 | CCAATGAATAAGCTCCACG found at 165490 line | HIVEP1 | [3096 (HIVEP1)](http://www.ncbi.nlm.nih.gov/gene/3096) | human immunodeficiency virus type I enhancer binding protein 1 | 11915136 .. 11915154 | 12,012,724..12,212,270 |  |  | 97588 |
| 163 | CCAATACATCTCTACCACG found at 253666 line | RNF144B | [255488 (RNF144B)](http://www.ncbi.nlm.nih.gov/gene/255488) | ring finger protein 144B | 18263843 .. 18263861 | 18,277,602..18,469,105 |  | 13759 |  |
| 164 | CCAATGAGGAGCTACCACG found at 265200 line | 0 |  |  |  |  |  |  |  |
| 165 | CCAATATGGTGAAACCACG found at 281534 line | 0 |  |  |  |  |  |  |  |
| 166 | CCAATCACCTCCCCCCACG found at 304588 line | 0 |  |  |  |  |  |  |  |
| 167 | CCAATTTTCTATACCCACG found at 392311 line | PGBD1 | [84547 (PGBD1)](http://www.ncbi.nlm.nih.gov/gene/84547) | piggyBac transposable element derived 1 | 28246297 .. 28246315 | 28,249,314..28,270,326 | 3017 |  |  |
| 168 | CCAATTATGTGCCACCACG found at 538766 line | DNAH8 | [1769 (DNAH8)](http://www.ncbi.nlm.nih.gov/gene/1769) | dynein, axonemal, heavy chain 8 | 38791045 .. 38791063 | 38,683,117..38,998,574 |  |  | element present inside the gene |
| 169 | CCAATCAAACTGAACCACG found at 551433 line | DAAM2 | [23500 (DAAM2)](http://www.ncbi.nlm.nih.gov/gene/23500) | dishevelled associated activator of morphogenesis 2 | 39703083 .. 39703101 | 39,760,159..39,872,653 |  |  | 57076 |
| 170 | CCAATCCATAACCACCACG found at 584679 line | GUCA1A | [2978 (GUCA1A)](http://www.ncbi.nlm.nih.gov/gene/2978) | guanylate cyclase activator 1A (retina) | 42096769 .. 42096787 | 42,123,144..42,147,794 |  |  | 26375 |
| 171 | CCAATACTGAATGACCACG found at 661393 line | GPR111 | [222611 (GPR111)](http://www.ncbi.nlm.nih.gov/gene/222611) | G protein-coupled receptor 111 | 47620176 .. 47620194 | 47,624,223..47,665,533 | 4047 |  |  |
| 172 | CCAATATCTGGTGCCCACG found at 815644 line | 0 |  |  |  |  |  |  |  |
| 173 | CCAATTACTTCAAACCACG found at 878238 line | 0 |  |  |  |  |  |  |  |
| 174 | CCAATCATCCTCTCCCACG found at 978123 line | 0 |  |  |  |  |  |  |  |
| 175 | CCAATATGGTGAGACCACG found at 987806 line | FAM135A | [57579 (FAM135A)](http://www.ncbi.nlm.nih.gov/gene/57579) | family with sequence similarity 135, member A | 71121943 .. 71121961 | 71,123,107..71,270,877 | 1164 |  |  |
| 176 | CCAATAAACCTTGGCCACG found at 1111464 line | 0 |  |  |  |  |  |  |  |
| 177 | CCAATGCTTAGGCTCCACG found at 1139481 line | 0 |  |  |  |  |  |  |  |
| 178 | CCAATCCCTTATTTCCACG found at 1364286 line | 0 |  |  |  |  |  |  |  |
| 179 | CCAATCCCTTATTTCCACG found at 1828412 line | 0 |  |  |  |  |  |  |  |
| 180 | CCAATCCCTTATTTCCACG found at 1836444 line | 0 |  |  |  |  |  |  |  |
| 181 | CCAATCCCTTATTTCCACG found at 1976916 line | 0 |  |  |  |  |  |  |  |
| 182 | CCAATAACTTGTGACCACG found at 1944822 line | 0 |  |  |  |  |  |  |  |
| 183 | CCAATCCCCAAAACCCACG found at 2059232 line | 0 |  |  |  |  |  |  |  |
| 184 | CCAATCACCTCCCTCCACG found at 2068849 line | 0 |  |  |  |  |  |  |  |
| 185 | CCAATAAAAATTCACCACG found at 2115340 line | ESR1 | [2099 (ESR1)](http://www.ncbi.nlm.nih.gov/gene/2099) | estrogen receptor 1 | 152304370 .. 152304388 | 152,011,631..152,424,409 |  |  | element present inside the gene |
| 186 | CCAATCTGTTTACTCCACG found at 2247769 line | 0 |  |  |  |  |  |  |  |
| 187 | CCAATTTAAATTAACCACG found at 2358073 line | 0 |  |  |  |  |  |  |  |
|  | chromosome 7 |  |  |  |  |  |  |  |  |
| 188 | CCAATCTGGCAGGACCACG found at 59193 line | SDK1 | [221935 (SDK1)](http://www.ncbi.nlm.nih.gov/gene/221935) | sidekick cell adhesion molecule 1 | 4261751 .. 4261769 | 3,341,080..4,308,632 |  |  | elements present inside the gene |
| 189 | CCAATAAGACCCCACCACG found at 79680 line | 0 |  |  |  |  |  |  |  |
| 190 | CCAATTAGAAGGTTCCACG found at 87188 line | 0 |  |  |  |  |  |  |  |
| 191 | CCAATCCCTTATTTCCACG found at 339405 line | 0 |  |  |  |  |  |  |  |
| 192 | CCAATCCCTTATTTCCACG found at 1743766 line | 0 |  |  |  |  |  |  |  |
| 193 | CCAATGCATAGTTACCACG found at 402250 line | 0 |  |  |  |  |  |  |  |
| 194 | CCAATATGATGAAACCACG found at 425010 line | GARS | [2617 (GARS)](http://www.ncbi.nlm.nih.gov/gene/2617) | glycyl-tRNA synthetase | 30600625 .. 30600643 | 30,634,181..30,673,649 |  |  | 33556 |
| 195 | CCAATCACCTCCCACCACG found at 451122 line | 0 |  |  |  |  |  |  |  |
| 196 | CCAATCACCTCCCACCACG found at 1589802 line | RPS14P10 (pseudo) | [647034 (RPS14P10)](http://www.ncbi.nlm.nih.gov/gene/647034) | ribosomal protein S14 pseudogene 10 | 131350056 .. 131350074 | 131,350,338..131,350,544 | 282 |  |  |
| 197 | CCAATCACCTCCCACCACG found at 1824308 line | 0 |  |  |  |  |  |  |  |
| 198 | CCAATCACCTCCCACCACG found at 1914078 line | 0 |  |  |  |  |  |  |  |
| 199 | CCAATGGTGAGGTTCCACG found at 631194 line | 0 |  |  |  |  |  |  |  |
| 200 | CCAATCAGAAAAGACCACG found at 781208 line | 0 |  |  |  |  |  |  |  |
| 201 | CCAATATGGTGAAGCCACG found at 875782 line | 0 |  |  |  |  |  |  |  |
| 202 | CCAATGGGGAAAGGCCACG found at 1050335 line | MDH2 | [4191 (MDH2)](http://www.ncbi.nlm.nih.gov/gene/4191) | malate dehydrogenase 2, NAD (mitochondrial) | 75623998 .. 75624016 | 75,677,182..75,696,826 |  |  | 53184 |
| 203 | CCAATTCCCTGAGACCACG found at 1053029 line | SRRM3 | [222183 (SRRM3)](http://www.ncbi.nlm.nih.gov/gene/222183) | serine/arginine repetitive matrix 3 | 75818001 .. 75818019 | 75,831,211..75,916,609 |  | 13210 |  |
| 204 | CCAATTACATTTTTCCACG found at 1085892 line | 0 |  |  |  |  |  |  |  |
| 205 | CCAATAGGTAAAGTCCACG found at 1239504 line | 0 |  |  |  |  |  |  |  |
| 206 | CCAATGTAAAATTACCACG found at 1253303 line | CDK14 | [5218 (CDK14)](http://www.ncbi.nlm.nih.gov/gene/5218) | cyclin-dependent kinase 14 | 90237659 .. 90237677 | 90,225,681..90,839,905 |  |  | element present inside the gene |
| 207 | CCAATTGGTGAATTCCACG found at 1318513 line | 0 |  |  |  |  |  |  |  |
| 208 | CCAATATGGTAAAACCACG found at 1331402 line | 0 |  |  |  |  |  |  |  |
| 209 | CCAATCACATTGGACCACG found at 1334672 line | 0 |  |  |  |  |  |  |  |
| 210 | CCAATTCTGCCGCACCACG found at 1356143 line | 0 |  |  |  |  |  |  |  |
| 211 | CCAATTTTTTGTGGCCACG found at 1587188 line | FOXP2 | [93986 (FOXP2)](http://www.ncbi.nlm.nih.gov/gene/93986) | forkhead box P2 | 114277386 .. 114277404 | 113,726,365..114,333,827 |  |  | element present inside the gene |
| 212 | CCAATCACCTCCAACCACG found at 1617011 line | MET | [4233 (MET)](http://www.ncbi.nlm.nih.gov/gene/4233) | met proto-oncogene | 116424694 .. 116424712 | 116,312,406..116,438,440 |  |  | element present inside the gene |
| 213 | CCAATGGGTAATACCCACG found at 1671072 line | KCND2 | [3751 (KCND2)](http://www.ncbi.nlm.nih.gov/gene/3751) | potassium voltage-gated channel, Shal-related subfamily, member 2 | 120317069 .. 120317087 | 119,913,689..120,390,387 |  |  | element present inside the gene |
| 214 | CCAATCTAGTCCCTCCACG found at 1742336 line | 0 |  |  |  |  |  |  |  |
| 215 | CCAATGTTAGTTACCCACG found at 1749231 line | 0 |  |  |  |  |  |  |  |
| 216 | CCAATATGGAGAAACCACG found at 1840609 line | 0 |  |  |  |  |  |  |  |
| 217 | CCAATTCTTCCTTTCCACG found at 1886662 line | 0 |  |  |  |  |  |  |  |
| 218 | CCAATATATAAGGACCACG found at 2045782 line | CNTNAP2 | [26047 (CNTNAP2)](http://www.ncbi.nlm.nih.gov/gene/26047) | contactin associated protein-like 2 | 147296163 .. 147296181 | 145,813,453..148,118,090 |  |  | element present inside the gene |
| 219 | CCAATGTCCTGGAGCCACG found at 2096321 line | CHPF2 | [54480 (CHPF2)](http://www.ncbi.nlm.nih.gov/gene/54480) | chondroitin polymerizing factor 2 | 150934983 .. 150935001 | 150,929,575..150,935,913 |  |  | element present inside the gene |
| 220 | CCAATGGACCAGGCCCACG found at 2126419 line | 0 |  |  |  |  |  |  |  |
| 221 | CCAATTTAAGGGGACCACG found at 2188335 line | 0 |  |  |  |  |  |  |  |
|  | chromosome 8 |  |  |  |  |  |  |  |  |
| 222 | CCAATGCACAGATGCCACG found at 39548 line | 0 |  |  |  |  |  |  |  |
| 223 | CCAATTATCATTCCCCACG found at 59165 line | 0 |  |  |  |  |  |  |  |
| 224 | CCAATTTATTCTCTCCACG found at 82364 line | 0 |  |  |  |  |  |  |  |
| 225 | CCAATCCCTTATTTCCACG found at 100847 line | 0 |  |  |  |  |  |  |  |
| 226 | CCAATCCCTTATTTCCACG found at 970644 line | HSPD1P3 Pseudo | [3332 (HSPD1P3)](http://www.ncbi.nlm.nih.gov/gene/3332) | heat shock 60kDa protein 1 (chaperonin) pseudogene 3 | 7260862 .. 7260880 | 7,248,860..7,278,554 |  |  | element present inside the gene |
| 227 | CCAATATGGTGAAACCACG found at 115240 line | XPO7 | [23039 (XPO7)](http://www.ncbi.nlm.nih.gov/gene/23039) | exportin 7 | 21741413 .. 21741431 | 21,777,180..21,864,096 |  |  | 35767 |
| 228 | CCAATATGGTGAAACCACG found at 284478 line | 0 |  |  |  |  |  |  |  |
| 229 | CCAATATGGTGAAACCACG found at 301966 line | 0 |  |  |  |  |  |  |  |
| 230 | CCAATCATACTTTTCCACG found at 122311 line | ERI1 | [90459 (ERI1)](http://www.ncbi.nlm.nih.gov/gene/90459) | exoribonuclease 1 | 8806293 .. 8806311 | 8,860,314..8,890,849 |  |  | 54021 |
| 231 | CCAATGCCTGAGCCCCACG found at 159462 line | GATA4 | [2626 (GATA4)](http://www.ncbi.nlm.nih.gov/gene/2626) | GATA binding protein 4 | 11481166 .. 11481184 | 11,534,433..11,617,510 |  |  | 53267 |
| 232 | CCAATCACCTCCCACCACG found at 375368 line | 0 |  |  |  |  |  |  |  |
| 233 | CCAATCACCTCCCACCACG found at 1999571 line | 0 |  |  |  |  |  |  |  |
| 234 | CCAATCCTCCACTCCCACG found at 426204 line | 0 |  |  |  |  |  |  |  |
| 235 | CCAATCTGCCTGAGCCACG found at 565581 line | 0 |  |  |  |  |  |  |  |
| 236 | CCAATTAGTAAAGCCCACG found at 729998 line | 0 |  |  |  |  |  |  |  |
| 237 | CCAATGCTACCCCTCCACG found at 868360 line | 0 |  |  |  |  |  |  |  |
| 238 | CCAATACCCTCTTACCACG found at 944187 line | CSPP1 | [79848 (CSPP1)](http://www.ncbi.nlm.nih.gov/gene/79848) | centrosome and spindle pole associated protein 1 | 67981320 .. 67981338 | 67,976,588..68,108,849 |  |  | element present inside the gene |
| 239 | CCAATCTTCATAATCCACG found at 976484 line | SULF1 | [23213 (SULF1)](http://www.ncbi.nlm.nih.gov/gene/23213) | sulfatase 1 | 70306745 .. 70306763 | 70,378,859..70,573,147 |  |  | 72114 |
| 240 | CCAATTCTTCATCTCCACG found at 986337 line | 0 |  |  |  |  |  |  |  |
| 241 | CCAATAAAAATAAACCACG found at 1015617 line | 0 |  |  |  |  |  |  |  |
| 242 | CCAATAAGTCCAGGCCACG found at 1333063 line | 0 |  |  |  |  |  |  |  |
| 243 | CCAATGCACTTATGCCACG found at 1404068 line | SPAG1 | [6674 (SPAG1)](http://www.ncbi.nlm.nih.gov/gene/6674) | sperm associated antigen 1 | 101092775 .. 101092793 | 101,170,263..101,254,132 |  |  | 77488 |
| 244 | CCAATGGGCCCCTTCCACG found at 1522562 line | 0 |  |  |  |  |  |  |  |
| 245 | CCAATTTTAATTTGCCACG found at 1565416 line | 0 |  |  |  |  |  |  |  |
| 246 | CCAATAGACAGCCTCCACG found at 1611773 line | 0 |  |  |  |  |  |  |  |
| 247 | CCAATTCATTCAGTCCACG found at 1641471 line | SLC30A8 | [169026 (SLC30A8)](http://www.ncbi.nlm.nih.gov/gene/169026) | solute carrier family 30 (zinc transporter), member 8 | 118185755 .. 118185773 | 117,962,512..118,188,953 |  |  | element present inside the gene |
| 248 | CCAATCCCAGCTCCCCACG found at 1972441 line | 0 |  |  |  |  |  |  |  |
| 249 | CCAATGGCTGAAGACCACG found at 1989452 line | 0 |  |  |  |  |  |  |  |
| 250 | CCAATCTCCGGCGCCCACG found at 2024277 line | LRRC14 | [9684 (LRRC14)](http://www.ncbi.nlm.nih.gov/gene/9684) | leucine rich repeat containing 14 | 145747820 .. 145747838 | 145,743,349..145,750,562 |  |  | element present inside the gene |
|  | chromosome 9 |  |  |  |  |  |  |  |  |
| 251 | CCAATATGTCATGGCCACG found at 276119 line | 0 |  |  |  |  |  |  |  |
| 252 | CCAATAGATTGAATCCACG found at 295882 line | 0 |  |  |  |  |  |  |  |
| 253 | CCAATAAAATGTAGCCACG found at 381818 line | IFNK | [56832 (IFNK)](http://www.ncbi.nlm.nih.gov/gene/56832) | interferon, kappa | 27490778 .. 27490796 | 27,524,312..27,526,496 |  |  | 33534 |
| 254 | CCAATATTCTCACCCCACG found at 530249 line | 0 |  |  |  |  |  |  |  |
| 255 | CCAATTAGCTGCTGCCACG found at 642955 line | 0 |  |  |  |  |  |  |  |
| 256 | CCAATTAGCTGCTGCCACG found at 940301 line | 0 |  |  |  |  |  |  |  |
| 257 | CCAATTTAATAACCCCACG found at 990769 line | PIP5K1B | [8395 (PIP5K1B)](http://www.ncbi.nlm.nih.gov/gene/8395) | phosphatidylinositol-4-phosphate 5-kinase, type I, beta | 71335255 .. 71335273 | 71,320,188..71,624,092 |  |  | element present inside the gene |
| 258 | CCAATATGATGAAACCACG found at 1077585 line | 0 |  |  |  |  |  |  |  |
| 259 | CCAATGGGTCCCTCCCACG found at 1092606 line | PCSK5 | [5125 (PCSK5)](http://www.ncbi.nlm.nih.gov/gene/5125) | proprotein convertase subtilisin/kexin type 5 | 78667477 .. 78667495 | 78,505,560..78,977,255 |  |  | element present inside the gene |
| 260 | CCAATATTTTACTGCCACG found at 1100758 line | 0 |  |  |  |  |  |  |  |
| 261 | CCAATACCCCTACTCCACG found at 1143378 line | TLE4 | [7091 (TLE4)](http://www.ncbi.nlm.nih.gov/gene/7091) | transducin-like enhancer of split 4 (E(sp1) homolog, Drosophila) | 82323064 .. 82323082 | 82,186,878..82,341,658 |  |  | element present inside the gene |
| 262 | CCAATCCCTTATTTCCACG found at 1161460 line | 0 |  |  |  |  |  |  |  |
| 263 | CCAATCCCTTATTTCCACG found at 1334875 line | 0 |  |  |  |  |  |  |  |
| 264 | CCAATGGCACCATGCCACG found at 1214195 line | NTRK2 | [4915 (NTRK2)](http://www.ncbi.nlm.nih.gov/gene/4915) | neurotrophic tyrosine kinase, receptor, type 2 | 87421895 .. 87421913 | 87,283,417..87,641,985 |  |  | element present inside the gene |
| 265 | CCAATGCTACTCAGCCACG found at 1296364 line | 0 |  |  |  |  |  |  |  |
| 266 | CCAATGAGCCCTTCCCACG found at 1318667 line | 0 |  |  |  |  |  |  |  |
| 267 | CCAATCTAGTCCTTCCACG found at 1331026 line | SUSD3 | [203328 (SUSD3)](http://www.ncbi.nlm.nih.gov/gene/203328) | sushi domain containing 3 | 95833750 .. 95833768 | 95,820,989..95,847,420 |  |  | element present inside the gene |
| 268 | CCAATCCATAGTGCCCACG found at 1359515 line | C9orf89 | [84270 (C9orf89)](http://www.ncbi.nlm.nih.gov/gene/84270) | chromosome 9 open reading frame 89 | 95833750 .. 95833768 | 95,858,450..95,875,565 |  |  | 24700 |
| 269 | CCAATCTCCAGTACCCACG found at 1404294 line | 0 |  |  |  |  |  |  |  |
| 270 | CCAATCTAATGTTTCCACG found at 1521533 line | 0 |  |  |  |  |  |  |  |
| 271 | CCAATGACTGAGCTCCACG found at 1547293 line | 0 |  |  |  |  |  |  |  |
| 272 | CCAATGATCCAGCACCACG found at 1663853 line | 0 |  |  |  |  |  |  |  |
| 273 | CCAATGGAGGATGTCCACG found at 1867593 line | 0 |  |  |  |  |  |  |  |
| 274 | CCAATTTAATCATGCCACG found at 1929372 line | 0 |  |  |  |  |  |  |  |
|  | chromosome 10 |  |  |  |  |  |  |  |  |
| 275 | CCAATGTTTAGCTCCCACG found at 124154 line | 0 |  |  |  |  |  |  |  |
| 276 | CCAATACCAAATGCCCACG found at 138077 line | 0 |  |  |  |  |  |  |  |
| 277 | CCAATGCCTCGAGCCCACG found at 178790 line | 0 |  |  |  |  |  |  |  |
| 278 | CCAATGATCACTTACCACG found at 458558 line | C10orf68 | [79741 (C10orf68)](http://www.ncbi.nlm.nih.gov/gene/79741) | chromosome 10 open reading frame 68 | 33016074 .. 33016092 | 32,735,057..33,171,805 |  |  | element present inside the gene |
| 279 | CCAATTTTGCCTGCCCACG found at 693144 line | WDFY4 | [57705 (WDFY4)](http://www.ncbi.nlm.nih.gov/gene/57705) | WDFY family member 4 | 49906278 .. 49906296 | 49,892,907..50,191,001 |  |  | element present inside the gene |
| 280 | CCAATATATAACCCCCACG found at 710207 line | TIMM23B | [653252 (TIMM23B)](http://www.ncbi.nlm.nih.gov/gene/653252) | translocase of inner mitochondrial membrane 23 homolog B (yeast) | 51356453 .. 51356471 | 51,371,396..51,734,455 |  | 14943 |  |
| 281 | CCAATATATAACCCCCACG found at 713286 line | 0 |  |  |  |  |  |  |  |
| 282 | CCAATCATTCTGACCCACG found at 723776 line | 0 |  |  |  |  |  |  |  |
| 283 | CCAATTACAAAGAGCCACG found at 771279 line | 0 |  |  |  |  |  |  |  |
| 284 | CCAATATGGTGAAACCACG found at 969744 line | 0 |  |  |  |  |  |  |  |
| 285 | CCAATATGGTGAAACCACG found at 1088118 line | 0 |  |  |  |  |  |  |  |
| 286 | CCAATTCCCAAACTCCACG found at 981388 line | DDX50 | [79009 (DDX50)](http://www.ncbi.nlm.nih.gov/gene/79009) | DEAD (Asp-Glu-Ala-Asp) box polypeptide 50 | 70659812 .. 70659830 | 70,661,034..70,706,603 | 1222 |  |  |
| 287 | CCAATCCGTGGGCCCCACG found at 1007760 line | SGPL1 | [8879 (SGPL1)](http://www.ncbi.nlm.nih.gov/gene/8879) | sphingosine-1-phosphate lyase 1 | 72558629 .. 72558647 | 72,575,704..72,640,946 |  | 17075 |  |
| 288 | CCAATTCTACTTAACCACG found at 1082994 line | C10orf11 | [83938 (C10orf11)](http://www.ncbi.nlm.nih.gov/gene/83938) | chromosome 10 open reading frame 11 | 77975435 .. 77975453 | 77,191,217..78,317,133 |  |  | element present inside the gene |
| 289 | CCAATCGCTGTCCCCCACG found at 1248054 line | 0 |  |  |  |  |  |  |  |
| 290 | CCAATCCCTTATTTCCACG found at 1278556 line | 0 |  |  |  |  |  |  |  |
| 291 | CCAATCCTGGTGCCCCACG found at 1348334 line | 0 |  |  |  |  |  |  |  |
| 292 | CCAATCACTCCTCACCACG found at 1390682 line | 0 |  |  |  |  |  |  |  |
| 293 | CCAATTTATACTACCCACG found at 1473624 line | CCDC147 | [159686 (CCDC147)](http://www.ncbi.nlm.nih.gov/gene/159686) | coiled-coil domain containing 147 | 106100793 .. 106100811 | 106,099,377..106,214,848 |  |  | element present inside the gene |
| 294 | CCAATGGAGTCTGGCCACG found at 1818294 line | 0 |  |  |  |  |  |  |  |
| 295 | CCAATCTGTGAGCCCCACG found at 1837747 line | 0 |  |  |  |  |  |  |  |
| 296 | CCAATGGTCTCCGGCCACG found at 1851396 line | 0 |  |  |  |  |  |  |  |
| 297 | CCAATCCCTAAACCCCACG found at 1867829 line | INPP5A | [3632 (INPP5A)](http://www.ncbi.nlm.nih.gov/gene/3632) | inositol polyphosphate-5-phosphatase, 40kDa | 134483588 .. 134483606 | 134,351,283..134,596,984 |  |  | element present inside the gene |
| 298 | CCAATTCTCACCGGCCACG found at 1872818 line | GPR123 | [84435 (GPR123)](http://www.ncbi.nlm.nih.gov/gene/84435) | G protein-coupled receptor 123 | 134842759 .. 134842777 | 134,884,433..134,945,179 |  |  | 41674 |
| 299 | CCAATGAGCTGCCCCCACG found at 1876117 line | 0 |  |  |  |  |  |  |  |
|  | chromosome 11 |  |  |  |  |  |  |  |  |
| 300 | CCAATACTGACATTCCACG found at 24879 line | 0 |  |  |  |  |  |  |  |
| 301 | CCAATGCCTGGGGTCCACG found at 44061 line | 0 |  |  |  |  |  |  |  |
| 302 | CCAATCCCTTATTTCCACG found at 50977 line | ART1 | [417 (ART1)](http://www.ncbi.nlm.nih.gov/gene/417) | ADP-ribosyltransferase 1 | 3670190 .. 3670208 | 3,663,487..3,685,844 |  |  | element present inside the gene |
| 303 | CCAATGGCATTTCTCCACG found at 79256 line | TRIM22 | [10346 (TRIM22)](http://www.ncbi.nlm.nih.gov/gene/10346) | tripartite motif containing 22 | 5706345 .. 5706363 | 5,710,817..5,732,093 | 4472 |  |  |
| 304 | CCAATCACCTCAGACCACG found at 182798 line | 0 |  |  |  |  |  |  |  |
| 305 | CCAATAGAAGAGTGCCACG found at 314193 line | 0 |  |  |  |  |  |  |  |
| 306 | CCAATTTGGGTGTTCCACG found at 468268 line | 0 |  |  |  |  |  |  |  |
| 307 | CCAATATTTTATATCCACG found at 617724 line | 0 |  |  |  |  |  |  |  |
| 308 | CCAATCAGGGACCTCCACG found at 622844 line | TSPAN18 | [90139 (TSPAN18)](http://www.ncbi.nlm.nih.gov/gene/90139) | tetraspanin 18 | 44844679 .. 44844697 | 44,748,731..44,953,978 |  |  | element present inside the gene |
| 309 | CCAATGTCCGGTGACCACG found at 647748 line | ATG13 | [9776 (ATG13)](http://www.ncbi.nlm.nih.gov/gene/9776) | autophagy related 13 | 46637721 .. 46637739 | 46,638,826..46,697,569 | 1105 |  |  |
| 310 | CCAATCATCTTGTACCACG found at 813428 line | 0 |  |  |  |  |  |  |  |
| 311 | CCAATATCCCTGGTCCACG found at 892433 line | 0 |  |  |  |  |  |  |  |
| 312 | CCAATGGGCCCGTGCCACG found at 897554 line | 0 |  |  |  |  |  |  |  |
| 313 | CCAATGGAAACAACCCACG found at 949555 line | PPP6R3 | [55291 (PPP6R3)](http://www.ncbi.nlm.nih.gov/gene/55291) | protein phosphatase 6, regulatory subunit 3 | 68367838 .. 68367856 | 68,228,186..68,382,802 |  |  | element present inside the gene |
| 314 | CCAATGCGTTTATCCCACG found at 951744 line | 0 |  |  |  |  |  |  |  |
| 315 | CCAATCGCCTCCCACCACG found at 960553 line | 0 |  |  |  |  |  |  |  |
| 316 | CCAATTCTTTATCTCCACG found at 973962 line | PPFIA1 | [8500 (PPFIA1)](http://www.ncbi.nlm.nih.gov/gene/8500) | protein tyrosine phosphatase, receptor type, f polypeptide (PTPRF), interacting protein (liprin), alpha 1 | 70125113 .. 70125131 | 70,116,806..70,230,607 |  |  | element present inside the gene |
| 317 | CCAATCCACCTCTGCCACG found at 1007084 line | ATG16L2 | [89849 (ATG16L2)](http://www.ncbi.nlm.nih.gov/gene/89849) | autophagy related 16-like 2 (S. cerevisiae) | 72509932 .. 72509950 | 72,525,451..72,553,793 |  | 15519 |  |
| 318 | CCAATTCACTGAGACCACG found at 1079940 line | THRSP | [7069 (THRSP)](http://www.ncbi.nlm.nih.gov/gene/7069) | thyroid hormone responsive | 77755561 .. 77755579 | 77,774,907..77,779,403 |  | 19346 |  |
| 319 | CCAATAAGTTTTCTCCACG found at 1282269 line | FAT3 | [120114 (FAT3)](http://www.ncbi.nlm.nih.gov/gene/120114) | FAT atypical cadherin 3 | 92323234 .. 92323252 | 92,047,446..92,629,636 |  |  | element present inside the gene |
| 320 | CCAATTCTAGGTTCCCACG found at 1306724 line | 0 |  |  |  |  |  |  |  |
| 321 | CCAATACAGTGGCCCCACG found at 1646335 line | 0 |  |  |  |  |  |  |  |
| 322 | CCAATATGGTGAAACCACG found at 1650679 line | FOXR1 | [283150 (FOXR1)](http://www.ncbi.nlm.nih.gov/gene/283150) | forkhead box R1 | 118848738 .. 118848756 | 118,842,417..118,851,997 |  |  | element present inside the gene |
| 323 | CCAATAAATGCAAACCACG found at 1668738 line | POU2F3 | [25833 (POU2F3)](http://www.ncbi.nlm.nih.gov/gene/25833) | POU class 2 homeobox 3 | 120149019 .. 120149037 | 120,107,349..120,190,653 |  |  | element present inside the gene |
| 324 | CCAATGTCCTTGAGCCACG found at 1732948 line | HEPN1 | [641654 (HEPN1)](http://www.ncbi.nlm.nih.gov/gene/641654) | hepatocellular carcinoma, down-regulated 1 | 124772127 .. 124772145 | 124,789,146..124,790,573 |  | 17019 |  |
| 325 | CCAATCTCCCTCTCCCACG found at 1760607 line | 0 |  |  |  |  |  |  |  |
| 326 | CCAATAAAGCTCTTCCACG found at 1840871 line | 0 |  |  |  |  |  |  |  |
|  | chromosome 12 |  |  |  |  |  |  |  |  |
| 327 | CCAATGAGAAGGGGCCACG found at 36465 line | CACNA1C | [775 (CACNA1C)](http://www.ncbi.nlm.nih.gov/gene/775) | calcium channel, voltage-dependent, L type, alpha 1C subunit | 2625385 .. 2625403 | 2,079,952..2,807,115 |  |  | element present inside the gene |
| 328 | CCAATGGATGATCCCCACG found at 85503 line | 0 |  |  |  |  |  |  |  |
| 329 | CCAATATGGTAAAACCACG found at 94836 line | COPS7A | [50813 (COPS7A)](http://www.ncbi.nlm.nih.gov/gene/50813) | COP9 signalosome subunit 7A | 6828048 .. 6828066 | 6,833,150..6,841,041 | 5102 |  |  |
| 330 | CCAATGTCAACTTACCACG found at 160097 line | 0 |  |  |  |  |  |  |  |
| 331 | CCAATCTGAGACACCCACG found at 176588 line | CREBL2 | [1389 (CREBL2)](http://www.ncbi.nlm.nih.gov/gene/1389) | cAMP responsive element binding protein-like 2 | 12714195 .. 12714213 | 12,764,831..12,798,041 |  |  | 50636 |
| 332 | CCAATAAACAAAAACCACG found at 176677 line | CREBL2 | [1389 (CREBL2)](http://www.ncbi.nlm.nih.gov/gene/1389) | cAMP responsive element binding protein-like 2 | 12720608 .. 12720626 | 12,764,831..12,798,041 |  |  | 44223 |
| 333 | CCAATTTTCATTTTCCACG found at 246464 line | 0 |  |  |  |  |  |  |  |
| 334 | CCAATTCTTCTTTTCCACG found at 375894 line | FGFR1OP2 | [26127 (FGFR1OP2)](http://www.ncbi.nlm.nih.gov/gene/26127) | FGFR1 oncogene partner 2 | 27064213 .. 27064231 | 27,091,305..27,119,581 |  |  | 27092 |
| 335 | CCAATGGGCTCTCACCACG found at 448324 line | BICD1 | [636 (BICD1)](http://www.ncbi.nlm.nih.gov/gene/636) | bicaudal D homolog 1 (Drosophila) | 32279221 .. 32279239 | 32,259,713..32,536,567 |  |  | element present inside the gene |
| 336 | CCAATGTGTAACCTCCACG found at 547672 line | 0 |  |  |  |  |  |  |  |
| 337 | CCAATTGTCTCTTACCACG found at 931256 line | 0 |  |  |  |  |  |  |  |
| 338 | CCAATGACCACCCCCCACG found at 976129 line | 0 |  |  |  |  |  |  |  |
| 339 | CCAATCCCTTATTTCCACG found at 1015254 line | 0 |  |  |  |  |  |  |  |
| 340 | CCAATCCCTTATTTCCACG found at 1212967 line | 0 |  |  |  |  |  |  |  |
| 341 | CCAATCCCTTATTTCCACG found at 1335333 line | 0 |  |  |  |  |  |  |  |
| 342 | CCAATGAGTCATTTCCACG found at 1052231 line | GLIPR1L1 | [256710 (GLIPR1L1)](http://www.ncbi.nlm.nih.gov/gene/256710) | GLI pathogenesis-related 1 like 1 | 75760525 .. 75760543 | 75,727,550..75,764,170 |  |  | element present inside the gene |
|  |  | GLIPR1L2 | [144321 (GLIPR1L2)](http://www.ncbi.nlm.nih.gov/gene/144321) | GLI pathogenesis-related 1 like 2 | 75760525 .. 75760543 | 75,784,850..75,826,468 |  |  | 24325 |
| 343 | CCAATCTCCCAATTCCACG found at 1388961 line | FAM71C | [196472 (FAM71C)](http://www.ncbi.nlm.nih.gov/gene/196472) | family with sequence similarity 71, member C | 100005078 .. 100005096 | 100,041,528..100,043,892 |  |  | 36450 |
| 344 | CCAATCGGAAGGAGCCACG found at 1448946 line | HSP90B1 | [7184 (HSP90B1)](http://www.ncbi.nlm.nih.gov/gene/7184) | heat shock protein 90kDa beta (Grp94), member 1 | 104323994 .. 104324012 | 104,324,112..104,341,708 | 118 |  |  |
|  |  | MIR3652 | [100500842 (MIR3652)](http://www.ncbi.nlm.nih.gov/gene/100500842) | microRNA 3652 | 104323994 .. 104324012 | 104,324,203..104,324,333 | 209 |  |  |
| 345 | CCAATGGGAGTGGCCCACG found at 1512625 line | FICD | [11153 (FICD)](http://www.ncbi.nlm.nih.gov/gene/11153) | FIC domain containing | 108908900 .. 108908918 | 108,909,051..108,913,380 | 151 |  |  |
| 346 | CCAATTAGCAGGTACCACG found at 1513764 line | 0 |  |  |  |  |  |  |  |
| 347 | CCAATGAGATGAAGCCACG found at 1623563 line | 0 |  |  |  |  |  |  |  |
|  | chromosome 13 |  |  |  |  |  |  |  |  |
| 348 | CCAATGGCCAGGCACCACG found at 374763 line | 0 |  |  |  |  |  |  |  |
| 349 | CCAATACGGCAAAACCACG found at 422389 line | 0 |  |  |  |  |  |  |  |
| 350 | CCAATATGGTGAAACCACG found at 627234 line | SMARCE1P5 (Pseudo) | [400129 (SMARCE1P5)](http://www.ncbi.nlm.nih.gov/gene/400129) | SWI/SNF related, matrix associated, actin dependent regulator of chromatin, subfamily e, member 1 pseudogene 5 | 45160720 .. 45160738 | 45,168,994..45,170,285 | 8274 |  |  |
| 351 | CCAATCAGATGCACCCACG found at 633513 line | 0 |  |  |  |  |  |  |  |
| 352 | CCAATGTGGGACTTCCACG found at 645884 line | 0 |  |  |  |  |  |  |  |
| 353 | CCAATCCAAGGAATCCACG found at 777931 line | 0 |  |  |  |  |  |  |  |
| 354 | CCAATGTGTACTTTCCACG found at 921489 line | 0 |  |  |  |  |  |  |  |
| 355 | CCAATTCTGAATACCCACG found at 938654 line | 0 |  |  |  |  |  |  |  |
| 356 | CCAATGATTAGCTCCCACG found at 969487 line | 0 |  |  |  |  |  |  |  |
| 357 | CCAATCACCTCCCACCACG found at 1051809 line | 0 |  |  |  |  |  |  |  |
| 358 | CCAATCCCTTATTTCCACG found at 1121652 line | 0 |  |  |  |  |  |  |  |
| 359 | CCAATCCCTTATTTCCACG found at 1208495 line | 0 |  |  |  |  |  |  |  |
| 360 | CCAATAATCTCATCCCACG found at 1216988 line | 0 |  |  |  |  |  |  |  |
| 361 | CCAATATGATGACACCACG found at 1383707 line | 0 |  |  |  |  |  |  |  |
| 362 | CCAATATGATGAAACCACG found at 1487944 line | 0 |  |  |  |  |  |  |  |
| 363 | CCAATCACCCCCCACCACG found at 1555132 line | TEX29 | [121793 (TEX29)](http://www.ncbi.nlm.nih.gov/gene/121793) | testis expressed 29 | 111969362 .. 111969380 | 111,973,015..111,996,594 | 3653 |  |  |
| 364 | CCAATATCTGTTCTCCACG found at 1586591 line | TFDP1 | [7027 (TFDP1)](http://www.ncbi.nlm.nih.gov/gene/7027) | transcription factor Dp-1 | 114234399 .. 114234417 | 114,239,003..114,295,788 | 4604 |  |  |
| 365 | CCAATGCGATGCTCCCACG found at 1593984 line | 0 |  |  |  |  |  |  |  |
|  | chromosome 14 |  |  |  |  |  |  |  |  |
| 366 | CCAATCCTCATGGTCCACG found at 343463 line | 0 |  |  |  |  |  |  |  |
| 367 | CCAATCCAAAGAATCCACG found at 518793 line | 0 |  |  |  |  |  |  |  |
| 368 | CCAATCCCTTATTTCCACG found at 536959 line | 0 |  |  |  |  |  |  |  |
| 369 | CCAATCCCTTATTTCCACG found at 536960 line | SSTR1 | [6751 (SSTR1)](http://www.ncbi.nlm.nih.gov/gene/6751) | somatostatin receptor 1 | 38661026 .. 38661044 | 38,677,204..38,682,268 |  | 16178 |  |
| 370 | CCAATCCCTTATTTCCACG found at 536960 line | 0 |  |  |  |  |  |  |  |
| 371 | CCAATCCCTTATTTCCACG found at 583144 line | 0 |  |  |  |  |  |  |  |
| 372 | CCAATACGGTGAAACCACG found at 551569 line | MIA2 | [117153 (MIA2)](http://www.ncbi.nlm.nih.gov/gene/117153) | melanoma inhibitory activity 2 | 39712881 .. 39712899 | 39,703,125..39,722,575 |  |  | element present inside the gene |
| 373 | CCAATGTGGTGAAACCACG found at 776446 line | 0 |  |  |  |  |  |  |  |
| 374 | CCAATTTTTAATTCCCACG found at 869588 line | 0 |  |  |  |  |  |  |  |
| 375 | CCAATCATGCCTTCCCACG found at 874500 line | 0 |  |  |  |  |  |  |  |
| 376 | CCAATATGGTGAAACCACG found at 892788 line | SYNE2 | [23224 (SYNE2)](http://www.ncbi.nlm.nih.gov/gene/23224) | spectrin repeat containing, nuclear envelope 2 | 64280581 .. 64280599 | 64,319,683..64,693,167 |  |  | 39102 |
| 377 | CCAATTCCAAGCACCCACG found at 960845 line | 0 |  |  |  |  |  |  |  |
| 378 | CCAATAGGTCTGTCCCACG found at 963276 line | 0 |  |  |  |  |  |  |  |
| 379 | CCAATATAACGAAACCACG found at 1035627 line | LIN52 | [91750 (LIN52)](http://www.ncbi.nlm.nih.gov/gene/91750) | lin-52 homolog (C. elegans) | 74565033 .. 74565051 | 74,551,656..74,667,117 |  |  | element present inside the gene |
| 380 | CCAATAATTTTTCTCCACG found at 1064360 line | GPATCH2L | [55668 (GPATCH2L)](http://www.ncbi.nlm.nih.gov/gene/55668) | G patch domain containing 2-like | 76633793 .. 76633811 | 76,618,255..76,671,239 |  |  | element present inside the gene |
| 381 | CCAATCAAACTCACCCACG found at 1241316 line | 0 |  |  |  |  |  |  |  |
| 382 | CCAATCGCTTATTTCCACG found at 1381287 line | 0 |  |  |  |  |  |  |  |
| 383 | CCAATACGGTAAAACCACG found at 1389584 line | 0 |  |  |  |  |  |  |  |
| 384 | CCAATCAGAGATTCCCACG found at 1489144 line | 0 |  |  |  |  |  |  |  |
|  | chromosome 15 |  |  |  |  |  |  |  |  |
| 385 | CCAATTCAGAGTTTCCACG found at 281787 line | 0 |  |  |  |  |  |  |  |
| 386 | CCAATTCAGAGTTTCCACG found at 295776 line | 0 |  |  |  |  |  |  |  |
| 387 | CCAATTTGAAAAGGCCACG found at 371294 line | 0 |  |  |  |  |  |  |  |
| 388 | CCAATGCTAGCTGCCCACG found at 380923 line | GABRG3 | [2567 (GABRG3)](http://www.ncbi.nlm.nih.gov/gene/2567) | gamma-aminobutyric acid (GABA) A receptor, gamma 3 | 27426327 .. 27426345 | 27,216,429..27,778,373 |  |  | element present inside the gene |
| 389 | CCAATTCAGTGTCTCCACG found at 409296 line | 0 |  |  |  |  |  |  |  |
| 390 | CCAATGATTCTAGTCCACG found at 450159 line | CHRNA7 (Partial stop) | [1139 (CHRNA7)](http://www.ncbi.nlm.nih.gov/gene/1139) | cholinergic receptor, nicotinic, alpha 7 (neuronal) | 32411362 .. 32411380 | 32,322,686..32,462,384 |  |  | element present inside the gene |
| 391 | CCAATGCGGGGCTTCCACG found at 459593 line | 0 |  |  |  |  |  |  |  |
| 392 | CCAATCCTGAGTGTCCACG found at 551195 line | 0 |  |  |  |  |  |  |  |
| 393 | CCAATATGGTGAAACCACG found at 617537 line | 0 |  |  |  |  |  |  |  |
| 394 | CCAATATGGTGAAACCACG found at 1224034 line | 0 |  |  |  |  |  |  |  |
| 395 | CCAATTATAGAGTCCCACG found at 763023 line | 0 |  |  |  |  |  |  |  |
| 396 | CCAATGTTAACCTCCCACG found at 796512 line | TCF12 | [6938 (TCF12)](http://www.ncbi.nlm.nih.gov/gene/6938) | transcription factor 12 | 57348767 .. 57348785 | 57,210,833..57,580,716 |  |  | element present inside the gene |
| 397 | CCAATATATCTTCCCCACG found at 806003 line | 0 |  |  |  |  |  |  |  |
| 398 | CCAATTCCAGCCGCCCACG found at 837420 line | FOXB1 | [27023 (FOXB1)](http://www.ncbi.nlm.nih.gov/gene/27023) | forkhead box B1 | 60294092 .. 60294110 | 60,296,421..60,298,142 | 2329 |  |  |
| 399 | CCAATTTCTGACTTCCACG found at 860147 line | 0 |  |  |  |  |  |  |  |
| 400 | CCAATCACAGCTATCCACG found at 1130692 line | C15orf26 | [161502 (C15orf26)](http://www.ncbi.nlm.nih.gov/gene/161502) | chromosome 15 open reading frame 26 | 81409690 .. 81409708 | 81,391,749..81,441,516 |  |  | element present inside the gene |
| 401 | CCAATGCACCTGGCCCACG found at 1274280 line | SV2B | [9899 (SV2B)](http://www.ncbi.nlm.nih.gov/gene/9899) | synaptic vesicle glycoprotein 2B | 91748071 .. 91748089 | 91,642,996..91,844,539 |  |  | element present inside the gene |
| 402 | CCAATGGTGAATTGCCACG found at 1323017 line | 0 |  |  |  |  |  |  |  |
| 403 | CCAATTGCTGAATCCCACG found at 1338659 line | 0 |  |  |  |  |  |  |  |
|  | chromosome 16 |  |  |  |  |  |  |  |  |
| 404 | CCAATGTGTTGAAACCACG found at 34568 line | CCNF | [899 (CCNF)](http://www.ncbi.nlm.nih.gov/gene/899) | cyclin F | 2488809 .. 2488827 | 2,479,395..2,508,859 |  |  | element present inside the gene |
| 405 | CCAATATCTGGCACCCACG found at 55128 line | 0 |  |  |  |  |  |  |  |
| 406 | CCAATCACAAAGGGCCACG found at 159190 line | 0 |  |  |  |  |  |  |  |
| 407 | CCAATGCCAATTTGCCACG found at 185231 line | 0 |  |  |  |  |  |  |  |
| 408 | CCAATTTGACTGGGCCACG found at 248730 line | 0 |  |  |  |  |  |  |  |
| 409 | CCAATCTATCCACTCCACG found at 263369 line | TMC7 | [79905 (TMC7)](http://www.ncbi.nlm.nih.gov/gene/79905) | transmembrane channel-like 7 | 18962455 .. 18962473 | 18,995,256..19,075,264 |  |  | 32801 |
| 410 | CCAATAAACAGTAGCCACG found at 316806 line | HS3ST2 | [9956 (HS3ST2)](http://www.ncbi.nlm.nih.gov/gene/9956) | heparan sulfate (glucosamine) 3-O-sulfotransferase 2 | 22809929 .. 22809947 | 22,825,860..22,927,659 |  | 15931 |  |
| 411 | CCAATCCTCCTCCTCCACG found at 414887 line | CDIPT-AS1 (Partial stop) | [440356 (CDIPT-AS1)](http://www.ncbi.nlm.nih.gov/gene/440356) | CDIPT antisense RNA 1 (head to head) | 29871740 .. 29871758 | 29,875,004..29,879,374 | 3264 |  |  |
| 412 | CCAATCACATCTTCCCACG found at 447153 line | 0 |  |  |  |  |  |  |  |
| 413 | CCAATCACATCTTCCCACG found at 460222 line | 0 |  |  |  |  |  |  |  |
| 414 | CCAATTTTATATACCCACG found at 729466 line | 0 |  |  |  |  |  |  |  |
| 415 | CCAATCCCTTAATTCCACG found at 834915 line | 0 |  |  |  |  |  |  |  |
| 416 | CCAATCAGACATGGCCACG found at 860545 line | 0 |  |  |  |  |  |  |  |
| 417 | CCAATTACTGTGACCCACG found at 862891 line | 0 |  |  |  |  |  |  |  |
| 418 | CCAATTAGAACACCCCACG found at 919250 line | 0 |  |  |  |  |  |  |  |
| 419 | CCAATTCAATCCACCCACG found at 921844 line | CDH5 | [1003 (CDH5)](http://www.ncbi.nlm.nih.gov/gene/1003) | cadherin 5, type 2 (vascular endothelium) | 66372652 .. 66372670 | 66,400,525..66,438,689 |  |  | 27873 |
| 420 | CCAATTTCCTTCTTCCACG found at 1111264 line | 0 |  |  |  |  |  |  |  |
| 421 | CCAATTAAATGACCCCACG found at 1183374 line | GSE1 | [23199 (GSE1)](http://www.ncbi.nlm.nih.gov/gene/23199) | Gse1 coiled-coil protein | 85202784 .. 85202802 | 85,203,152..85,709,812 | 368 |  |  |
| 422 | CCAATCCCCACCCTCCACG found at 1230760 line | ZC3H18 | [124245 (ZC3H18)](http://www.ncbi.nlm.nih.gov/gene/124245) | zinc finger CCCH-type containing 18 | 88614605 .. 88614623 | 88,636,789..88,698,372 |  |  | 22184 |
| 423 | CCAATGGGCAGAGCCCACG found at 1233365 line | 0 |  |  |  |  |  |  |  |
| 424 | CCAATAACCAACAGCCACG found at 1233874 line | CDT1 | [81620 (CDT1)](http://www.ncbi.nlm.nih.gov/gene/81620) | chromatin licensing and DNA replication factor 1 | 88838796 .. 88838814 | 88,870,186..88,875,666 |  |  | 31390 |
| 425 | CCAATCCCTGGCTTCCACG found at 1237892 line | ACSF3 | [197322 (ACSF3)](http://www.ncbi.nlm.nih.gov/gene/197322) | acyl-CoA synthetase family member 3 | 89128124 .. 89128142 | 89,160,217..89,222,171 |  |  | 32093 |
|  | chromosome 17 |  |  |  |  |  |  |  |  |
| 426 | CCAATATGGTGAAACCACG found at 139353 line | 0 |  |  |  |  |  |  |  |
| 427 | CCAATCCCTTATTTCCACG found at 164950 line | MAP2K4 | [6416 (MAP2K4)](http://www.ncbi.nlm.nih.gov/gene/6416) | mitogen-activated protein kinase kinase 4 | 11876303 .. 11876321 | 11,924,135..12,047,148 |  |  | 47832 |
| 428 | CCAATTCTCACTCACCACG found at 220197 line | ADORA2B | [136 (ADORA2B)](http://www.ncbi.nlm.nih.gov/gene/136) | adenosine A2b receptor | 15854097 .. 15854115 | 15,848,231..15,879,210 |  |  | element present inside the gene |
| 429 | CCAATGTCACACAACCACG found at 263108 line | 0 |  |  |  |  |  |  |  |
| 430 | CCAATACATTTATACCACG found at 274196 line | 0 |  |  |  |  |  |  |  |
| 431 | CCAATTTATCAGCTCCACG found at 579507 line | 0 |  |  |  |  |  |  |  |
| 432 | CCAATGAGGCCTTCCCACG found at 599760 line | NMT1 | [4836 (NMT1)](http://www.ncbi.nlm.nih.gov/gene/4836) | N-myristoyltransferase 1 | 43182600 .. 43182618 | 43,138,322..43,186,384 |  |  | element present inside the gene |
| 433 | CCAATGTGTTGAAACCACG found at 660225 line | NGFR | [4804 (NGFR)](http://www.ncbi.nlm.nih.gov/gene/4804) | nerve growth factor receptor | 47536077 .. 47536095 | 47,572,655..47,592,382 |  |  | 36578 |
| 434 | CCAATAAACATCTCCCACG found at 703421 line | 0 |  |  |  |  |  |  |  |
| 435 | CCAATATAGTGAAACCACG found at 827676 line | 0 |  |  |  |  |  |  |  |
| 436 | CCAATTTATTTGACCCACG found at 929889 line | 0 |  |  |  |  |  |  |  |
| 437 | CCAATCTTCTTTGACCACG found at 976531 line | 0 |  |  |  |  |  |  |  |
| 438 | CCAATCAGGCAGATCCACG found at 983669 line | 0 |  |  |  |  |  |  |  |
| 439 | CCAATATCCATGTGCCACG found at 1073851 line | 0 |  |  |  |  |  |  |  |
| 440 | CCAATGGCCCGACCCCACG found at 1080051 line | 0 |  |  |  |  |  |  |  |
| 441 | CCAATCCAGAAAGACCACG found at 1083418 line | CCDC40 | [55036 (CCDC40)](http://www.ncbi.nlm.nih.gov/gene/55036) | coiled-coil domain containing 40 | 78006000 .. 78006018 | 78,010,431..78,074,412 | 4431 |  |  |
| 442 | CCAATCCCCATCCCCCACG found at 1108027 line | 0 |  |  |  |  |  |  |  |
|  | chromosome 18 |  |  |  |  |  |  |  |  |
| 443 | CCAATGGTAAGTGGCCACG found at 83448 line | 0 |  |  |  |  |  |  |  |
| 444 | CCAATATGGTGAAACCACG found at 165437 line | 0 |  |  |  |  |  |  |  |
| 445 | CCAATATGGTGAAACCACG found at 603520 line | 0 |  |  |  |  |  |  |  |
| 446 | CCAATATGGTGAAACCACG found at 654474 line | 0 |  |  |  |  |  |  |  |
| 447 | CCAATCTGCTATGGCCACG found at 312531 line | 0 |  |  |  |  |  |  |  |
| 448 | CCAATTATTCTTAACCACG found at 320617 line | 0 |  |  |  |  |  |  |  |
| 449 | CCAATGTTTCTGGACCACG found at 446677 line | DTNA | [1837 (DTNA)](http://www.ncbi.nlm.nih.gov/gene/1837) | dystrobrevin, alpha | 32160620 .. 32160638 | 32,073,254..32,471,808 |  |  | element present inside the gene |
| 450 | CCAATTATAAAGTCCCACG found at 568931 line | 0 |  |  |  |  |  |  |  |
| 451 | CCAATCGGGGCGGTCCACG found at 618679 line | KATNAL2 | [83473 (KATNAL2)](http://www.ncbi.nlm.nih.gov/gene/83473) | katanin p60 subunit A-like 2 | 44556629 .. 44556647 | 44,497,565..44,628,614 |  |  | element present inside the gene |
| 452 | CCAATCGGGGCGGTCCACG found at 618762 line | KATNAL2 | [83473 (KATNAL2)](http://www.ncbi.nlm.nih.gov/gene/83473) | katanin p60 subunit A-like 2 | 44550714 .. 44550732 | 44,497,565..44,628,614 |  |  | element present inside the gene |
| 453 | CCAATCGGGGCGGTCCACG found at 618844 line | KATNAL2 | [83473 (KATNAL2)](http://www.ncbi.nlm.nih.gov/gene/83473) | katanin p60 subunit A-like 2 | 44544787 .. 44544805 | 44,497,565..44,628,614 |  |  | element present inside the gene |
| 454 | CCAATTTGTTGGTCCCACG found at 643118 line | CTIF | [9811 (CTIF)](http://www.ncbi.nlm.nih.gov/gene/9811) | CBP80/20-dependent translation initiation factor | 46304377 .. 46304395 | 46,065,427..46,389,588 |  |  | element present inside the gene |
| 455 | CCAATATTCTTTGCCCACG found at 679296 line | 0 |  |  |  |  |  |  |  |
| 456 | CCAATAAAAATATCCCACG found at 829388 line | 0 |  |  |  |  |  |  |  |
| 457 | CCAATTACTTTCCACCACG found at 974308 line | 0 |  |  |  |  |  |  |  |
|  | chromosome 19 |  |  |  |  |  |  |  |  |
| 458 | CCAATCCCGGCTTCCCACG found at 4774 line | 0 |  |  |  |  |  |  |  |
| 459 | CCAATGCCAAGACCCCACG found at 29224 line | 0 |  |  |  |  |  |  |  |
| 460 | CCAATCCGCGACACCCACG found at 45782 line | CELF5 (Partial stop) | [60680 (CELF5)](http://www.ncbi.nlm.nih.gov/gene/60680) | CUGBP, Elav-like family member 5 | 3296210 .. 3296228 | 3,224,701..3,297,074 |  |  | element present inside the gene |
| 461 | CCAATTCCACAGCTCCACG found at 61743 line | HDGFRP2 | [84717 (HDGFRP2)](http://www.ncbi.nlm.nih.gov/gene/84717) | hepatoma-derived growth factor-related protein 2 | 4445341 .. 4445359 | 4,472,255..4,502,223 |  |  | 26914 |
| 462 | CCAATAAACCGGCACCACG found at 71015 line | KDM4B | [23030 (KDM4B)](http://www.ncbi.nlm.nih.gov/gene/23030) | lysine (K)-specific demethylase 4B | 5112991 .. 5113009 | 4,969,123..5,153,609 |  |  | element present inside the gene |
| 463 | CCAATATGGTGAAACCACG found at 219070 line | CYP4F12 | [66002 (CYP4F12)](http://www.ncbi.nlm.nih.gov/gene/66002) | cytochrome P450, family 4, subfamily F, polypeptide 12 | 15772892 .. 15772910 | 15,783,567..15,807,984 | 10675 |  |  |
| 464 | CCAATGTTTAGCTCCCACG found at 394547 line | 0 |  |  |  |  |  |  |  |
| 465 | CCAATTCTCTTCTCCCACG found at 433244 line | 0 |  |  |  |  |  |  |  |
| 466 | CCAATACGGTGAAACCACG found at 460325 line | NUDT19 | [390916 (NUDT19)](http://www.ncbi.nlm.nih.gov/gene/390916) | nudix (nucleoside diphosphate linked moiety X)-type motif 19 | 33143307 .. 33143325 | 33,157,186..33,204,702 |  | 13879 |  |
| 467 | CCAATGTAGTGAAACCACG found at 485093 line | UBA2 | [10054 (UBA2)](http://www.ncbi.nlm.nih.gov/gene/10054) | ubiquitin-like modifier activating enzyme 2 | 34926546 .. 34926564 | 34,919,264..34,960,798 |  |  | element present inside the gene |
| 468 | CCAATGTGGTGAAACCACG found at 518418 line | ZNF345 | [25850 (ZNF345)](http://www.ncbi.nlm.nih.gov/gene/25850) | zinc finger protein 345 | 37325999 .. 37326017 | 37,341,260..37,384,120 |  | 15261 |  |
| 469 | CCAATTCACTGAGACCACG found at 587577 line | CEACAM3 | [1084 (CEACAM3)](http://www.ncbi.nlm.nih.gov/gene/1084) | carcinoembryonic antigen-related cell adhesion molecule 3 | 42305413 .. 42305431 | 42,300,522..42,315,591 |  |  | element present inside the gene |
| 470 | CCAATCCATAATGACCACG found at 653179 line | 0 |  |  |  |  |  |  |  |
| 471 | CCAATGTTGCTTCTCCACG found at 723875 line | 0 |  |  |  |  |  |  |  |
| 472 | CCAATCAGGACGCTCCACG found at 729605 line | 0 |  |  |  |  |  |  |  |
| 473 | CCAATCATAATCCCCCACG found at 779660 line | ZNF580 | [51157 (ZNF580)](http://www.ncbi.nlm.nih.gov/gene/51157) | zinc finger protein 580 | 56135432 .. 56135450 | 56,146,361..56,154,836 | 10929 |  |  |
| 474 | CCAATCAAATGGCACCACG found at 782611 line | NLRP4 | [147945 (NLRP4)](http://www.ncbi.nlm.nih.gov/gene/147945) | NLR family, pyrin domain containing 4 | 56347853 .. 56347871 | 56,347,944..56,393,221 | 91 |  |  |
| 475 | CCAATGAAGCCCCTCCACG found at 804478 line | ZNF17 | [7565 (ZNF17)](http://www.ncbi.nlm.nih.gov/gene/7565) | zinc finger protein 17 | 57922288 .. 57922306 | 57,922,529..57,933,307 | 241 |  |  |
|  | chromosome 20 |  |  |  |  |  |  |  |  |
| 476 | CCAATGCGCAGGTGCCACG found at 125674 line | PLCB4 | [5332 (PLCB4)](http://www.ncbi.nlm.nih.gov/gene/5332) | phospholipase C, beta 4 | 9048427 .. 9048445 | 9,049,357..9,461,463 | 241 |  |  |
| 477 | CCAATTGATAGGGACCACG found at 162257 line | 0 |  |  |  |  |  |  |  |
| 478 | CCAATAAGGTGAAACCACG found at 162748 line | 0 |  |  |  |  |  |  |  |
| 479 | CCAATATGGTGAAACCACG found at 495232 line | 0 |  |  |  |  |  |  |  |
| 480 | CCAATATGGTGAAACCACG found at 683816 line | 0 |  |  |  |  |  |  |  |
| 481 | CCAATCTGCTTTAGCCACG found at 503214 line | 0 |  |  |  |  |  |  |  |
| 482 | CCAATCCCTTATTTCCACG found at 616517 line | DNTTIP1 | [116092 (DNTTIP1)](http://www.ncbi.nlm.nih.gov/gene/116092) | deoxynucleotidyltransferase, terminal, interacting protein 1 | 44389068 .. 44389086 | 44,420,576..44,440,066 |  |  | 31508 |
| 483 | CCAATCTACTGCATCCACG found at 651810 line | 0 |  |  |  |  |  |  |  |
| 484 | CCAATAGTGCAGGGCCACG found at 691900 line | 0 |  |  |  |  |  |  |  |
| 485 | CCAATGGGCTGAAACCACG found at 754313 line | 0 |  |  |  |  |  |  |  |
|  | chromosome 21 |  |  |  |  |  |  |  |  |
| 486 | CCAATTCAAATATGCCACG found at 363580 line | 0 |  |  |  |  |  |  |  |
| 487 | CCAATCAGCCTTGTCCACG found at 408656 line | 0 |  |  |  |  |  |  |  |
| 488 | CCAATGGGTCCCTCCCACG found at 452798 line | 0 |  |  |  |  |  |  |  |
| 489 | CCAATATTGCTTACCCACG found at 492620 line | SLC5A3 | [6526 (SLC5A3)](http://www.ncbi.nlm.nih.gov/gene/6526) | solute carrier family 5 (sodium/myo-inositol cotransporter), member 3 | 35468537 .. 35468555 | 35,445,503..35,515,334 |  |  | element present inside the gene |
|  |  | MRPS6 | [64968 (MRPS6)](http://www.ncbi.nlm.nih.gov/gene/64968) | mitochondrial ribosomal protein S6 | 35468537 .. 35468555 | 35,445,823..35,515,334 |  |  | element present inside the gene |
| 490 | CCAATCCCCACCCCCCACG found at 596040 line | 0 |  |  |  |  |  |  |  |
|  | chromosome 22 |  |  |  |  |  |  |  |  |
| 491 | CCAATCATATTTCACCACG found at 262772 line | 0 |  |  |  |  |  |  |  |
| 492 | CCAATGGAGGCAACCCACG found at 263369 line | DGCR5 (Partial stop) | [26220 (DGCR5)](http://www.ncbi.nlm.nih.gov/gene/26220) | DiGeorge syndrome critical region gene 5 (non-protein coding) | 18962438 .. 18962456 | 18,958,011..18,982,142 |  |  | element present inside the gene |
| 493 | CCAATCTGGGTTTGCCACG found at 276937 line | COMT | [1312 (COMT)](http://www.ncbi.nlm.nih.gov/gene/1312) | catechol-O-methyltransferase | 19939351 .. 19939369 | 19,929,263..19,957,498 |  |  | element present inside the gene |
| 494 | CCAATCAGATTCTCCCACG found at 298211 line | 0 |  |  |  |  |  |  |  |
| 495 | CCAATCAGATTCTCCCACG found at 319108 line | BCR | [613 (BCR)](http://www.ncbi.nlm.nih.gov/gene/613) | breakpoint cluster region | 23654711 .. 23654729 | 23,522,402..23,660,224 |  |  | element present inside the gene |
| 496 | CCAATCAGATTCTCCCACG found at 328539 line | IGL | [3535 (IGL)](http://www.ncbi.nlm.nih.gov/gene/3535) | immunoglobulin lambda locus | 22975625 .. 22975643 | 22,380,474..23,265,085 |  |  | element present inside the gene |
|  |  | GGTLC2 | [91227 (GGTLC2)](http://www.ncbi.nlm.nih.gov/gene/91227) | gamma-glutamyltransferase light chain 2 | 22975625 .. 22975643 | 22,987,059..22,990,368 |  | 11434 |  |
| 497 | CCAATCAGATTCTCCCACG found at 347832 line | 0 |  |  |  |  |  |  |  |
| 498 | CCAATGTGGGTGAGCCACG found at 391006 line | 0 |  |  |  |  |  |  |  |
| 499 | CCAATCGCTTATTTCCACG found at 456943 line | 0 |  |  |  |  |  |  |  |
| 500 | CCAATATGGTGAAACCACG found at 470829 line | 0 |  |  |  |  |  |  |  |
| 501 | CCAATAGCAAGAGTCCACG found at 510603 line | 0 |  |  |  |  |  |  |  |
| 502 | CCAATGGGGAGGCCCCACG found at 549238 line | FUNDC2P4 (Pseudo) | [100127979 (FUNDC2P4)](http://www.ncbi.nlm.nih.gov/gene/100127979) | FUN14 domain containing 2 pseudogene 4 | 39545013 .. 39545031 | 39,551,541..39,552,101 | 6528 |  |  |
| 503 | CCAATGCCCTCAGCCCACG found at 646056 line | PPARA | [5465 (PPARA)](http://www.ncbi.nlm.nih.gov/gene/5465) | peroxisome proliferator-activated receptor alpha | 46515925 .. 46515943 | 46,546,458..46,639,653 |  |  | 30533 |
| 504 | CCAATGACGACAGTCCACG found at 691186 line | 0 |  |  |  |  |  |  |  |
|  | chromosome x |  |  |  |  |  |  |  |  |
| 505 | CCAATCAGTGATCTCCACG found at 110514 line | 0 |  |  |  |  |  |  |  |
| 506 | CCAATTTCGTTTTCCCACG found at 202048 line | GLRA2 | [2742 (GLRA2)](http://www.ncbi.nlm.nih.gov/gene/2742) | glycine receptor, alpha 2 | 14547313 .. 14547331 | 14,547,420..14,749,934 | 107 |  |  |
| 507 | CCAATAACCAGCACCCACG found at 238809 line | 0 |  |  |  |  |  |  |  |
| 508 | CCAATTCCAAGCTTCCACG found at 247717 line | 0 |  |  |  |  |  |  |  |
| 509 | CCAATAACAGTACTCCACG found at 486447 line | 0 |  |  |  |  |  |  |  |
| 510 | CCAATCAGTATAAACCACG found at 560173 line | 0 |  |  |  |  |  |  |  |
| 511 | CCAATTTCCTTATCCCACG found at 585208 line | 0 |  |  |  |  |  |  |  |
| 512 | CCAATATGGTGAAACCACG found at 621380 line | 0 |  |  |  |  |  |  |  |
| 513 | CCAATATGGTGAAACCACG found at 2008143 line | KDM6A | [7403 (KDM6A)](http://www.ncbi.nlm.nih.gov/gene/7403) | lysine (K)-specific demethylase 6A | 44739230 .. 44739248 | 44,732,419..44,972,024 |  |  | element present inside the gene |
| 514 | CCAATGTGGCAAAGCCACG found at 647757 line | RP2 | [6102 (RP2)](http://www.ncbi.nlm.nih.gov/gene/6102) | retinitis pigmentosa 2 (X-linked recessive) | 46638365 .. 46638383 | 46,696,347..46,741,793 |  |  | 57982 |
| 515 | CCAATCTCAGCCCTCCACG found at 658764 line | ARAF | [369 (ARAF)](http://www.ncbi.nlm.nih.gov/gene/369) | v-raf murine sarcoma 3611 viral oncogene homolog | 47430880 .. 47430898 | 47,420,499..47,431,320 |  |  | element present inside the gene |
|  |  | TIMP1 | [7076 (TIMP1)](http://www.ncbi.nlm.nih.gov/gene/7076) | TIMP metallopeptidase inhibitor 1 | 47430880 .. 47430898 | 47,441,690..47,446,190 | 10810 |  |  |
| 516 | CCAATTTGGATTGACCACG found at 959770 line | EDA | [1896 (EDA)](http://www.ncbi.nlm.nih.gov/gene/1896) | ectodysplasin A | 69103313 .. 69103331 | 68,835,911..69,259,322 |  |  | element present inside the gene |
| 517 | CCAATCCCTTATTTCCACG found at 1278899 line | 0 |  |  |  |  |  |  |  |
| 518 | CCAATCCCTTATTTCCACG found at 1685514 line | 0 |  |  |  |  |  |  |  |
| 519 | CCAATCACCTCCCACCACG found at 1287748 line | 0 |  |  |  |  |  |  |  |
| 520 | CCAATCACCTCCCACCACG found at 1761277 line | 0 |  |  |  |  |  |  |  |
| 521 | CCAATATTTAGCTCCCACG found at 1320515 line | 0 |  |  |  |  |  |  |  |
| 522 | CCAATTTATAGTAACCACG found at 1531667 line | PAK3 | [5063 (PAK3)](http://www.ncbi.nlm.nih.gov/gene/5063) | p21 protein (Cdc42/Rac)-activated kinase 3 | 110279931 .. 110279949 | 110,187,513..110,470,590 |  |  | element present inside the gene |
| 523 | CCAATTAATCATCTCCACG found at 1537373 line | 0 |  |  |  |  |  |  |  |
| 524 | CCAATGTCCAGGTACCACG found at 1610089 line | 0 |  |  |  |  |  |  |  |
| 525 | CCAATTGTGCCAGTCCACG found at 1662050 line | 0 |  |  |  |  |  |  |  |
| 526 | CCAATCTCTGCCTCCCACG found at 1711030 line | STAG2 | [10735 (STAG2)](http://www.ncbi.nlm.nih.gov/gene/10735) | stromal antigen 2 | 123194057 .. 123194075 | 123,094,475..123,236,506 |  |  | element present inside the gene |
| 527 | CCAATCATTCTGGTCCACG found at 1729396 line | 0 |  |  |  |  |  |  |  |
| 528 | CCAATTAAAACTCCCCACG found at 1866086 line | 0 |  |  |  |  |  |  |  |
| 529 | CCAATGGTGCACTGCCACG found at 1888262 line | 0 |  |  |  |  |  |  |  |
| 530 | CCAATCATACTTCTCCACG found at 2004183 line | SPANXN1 | [494118 (SPANXN1)](http://www.ncbi.nlm.nih.gov/gene/494118) | SPANX family, member N1 | 144301089 .. 144301107 | 144,329,107..144,337,728 |  |  | 28018 |
|  | chromosome Y |  |  |  |  |  |  |  |  |
| 531 | CCAATCGCTTATTTCCACG found at 80700 line | 0 |  |  |  |  |  |  |  |
| 532 | CCAATGTGAATACTCCACG found at 117300 line | 0 |  |  |  |  |  |  |  |
| 533 | CCAATGGGACCTGACCACG found at 133732 line | 0 |  |  |  |  |  |  |  |
| 534 | CCAATGTATGATCTCCACG found at 254420 line | 0 |  |  |  |  |  |  |  |
| 535 | CCAATACAGGAGCACCACG found at 373953 line | 0 |  |  |  |  |  |  |  |
|  |  |  |  |  |  |  |  |  |  |
|  |  |  |  | **ERSE-II** |  |  |  |  |  |
|  | chrom 1-------33 match |  |  |  |  |  |  |  |  |
| 1 | ATTGGACCACG found at 80952 line | PAPPA2 | [60676 (PAPPA2)](http://www.ncbi.nlm.nih.gov/gene/60676) | pappalysin 2 | 176703973 .. 176703983 | 176,432,307..176,814,737 |  |  | element present inside the gene |
| 2 | ATTGGACCACG found at 107845 line | ATP1A2 | [477 (ATP1A2)](http://www.ncbi.nlm.nih.gov/gene/477) | ATPase, Na+/K+ transporting, alpha 2 polypeptide | 160080318 .. 160080328 | 160,085,520..160,113,381 | 5202 |  |  |
| 3 | ATTGGACCACG found at 1598543 line | 0 |  |  |  |  |  |  |  |
| 4 | ATTGGACCACG found at 2223339 line | CAMTA1 (Partial stop) | [23261 (CAMTA1)](http://www.ncbi.nlm.nih.gov/gene/23261) | calmodulin binding transcription activator 1 | 7764690 .. 7764700 | 6,845,384..7,829,766 |  |  | element present inside the gene |
| 5 | ATTGGACCACG found at 2454224 line | 0 |  |  |  |  |  |  |  |
| 6 | ATTGGCCCACG found at 158362 line | 0 |  |  |  |  |  |  |  |
| 7 | ATTGGCCCACG found at 217378 line | RABGAP1L | [9910 (RABGAP1L)](http://www.ncbi.nlm.nih.gov/gene/9910) | RAB GTPase activating protein 1-like | 174741066 .. 174741076 | 174,128,552..174,964,445 |  |  | element present inside the gene |
| 8 | ATTGGCCCACG found at 277603 line | 0 |  |  |  |  |  |  |  |
| 9 | ATTGGCCCACG found at 931449 line | SGIP1 | [84251 (SGIP1)](http://www.ncbi.nlm.nih.gov/gene/84251) | SH3-domain GRB2-like (endophilin) interacting protein 1 | 67064223 .. 67064233 | 66,999,066..67,210,768 |  |  | element present inside the gene |
| 10 | ATTGGCCCACG found at 1181268 line | HTR6 | [3362 (HTR6)](http://www.ncbi.nlm.nih.gov/gene/3362) | 5-hydroxytryptamine (serotonin) receptor 6, G protein-coupled | 19987265 .. 19987275 | 19,991,780..20,006,055 | 4515 |  |  |
| 11 | ATTGGCCCACG found at 2426961 line | FHAD1 | [114827 (FHAD1)](http://www.ncbi.nlm.nih.gov/gene/114827) | forkhead-associated (FHA) phosphopeptide binding domain 1 | 15651081 .. 15651091 | 15,573,730..15,724,767 |  |  | element present inside the gene |
| 12 | ATTGGCCCACG found at 3184467 line | 0 |  |  |  |  |  |  |  |
| 13 | ATTGGGCCACG found at 185367 line | KIF26B | [55083 (KIF26B)](http://www.ncbi.nlm.nih.gov/gene/55083) | kinesin family member 26B | 245765575 .. 245765585 | 245,318,287..245,866,428 |  |  | element present inside the gene |
| 14 | ATTGGGCCACG found at 218091 line | 0 |  |  |  |  |  |  |  |
| 15 | ATTGGGCCACG found at 615624 line | 0 |  |  |  |  |  |  |  |
| 16 | ATTGGGCCACG found at 881019 line | PAPPA2 | [60676 (PAPPA2)](http://www.ncbi.nlm.nih.gov/gene/60676) | pappalysin 2 | 176583487 .. 176583497 | 176,432,307..176,814,737 |  |  | element present inside the gene |
| 17 | ATTGGGCCACG found at 921313 line | 0 |  |  |  |  |  |  |  |
| 18 | ATTGGGCCACG found at 999485 line | 0 |  |  |  |  |  |  |  |
| 19 | ATTGGGCCACG found at 1546086 line | 0 |  |  |  |  |  |  |  |
| 20 | ATTGGGCCACG found at 2380410 line | PDE4B | [5142 (PDE4B)](http://www.ncbi.nlm.nih.gov/gene/5142) | phosphodiesterase 4B, cAMP-specific | 66334387 .. 66334397 | 66,258,193..66,840,262 |  |  | element present inside the gene |
| 21 | ATTGGGCCACG found at 2452550 line | 0 |  |  |  |  |  |  |  |
| 22 | ATTGGGCCACG found at 2524822 line | ST3GAL3 (Partial stop) | [6487 (ST3GAL3)](http://www.ncbi.nlm.nih.gov/gene/6487) | ST3 beta-galactoside alpha-2,3-sialyltransferase 3 | 44324790 .. 44324800 | 44,173,204..44,396,837 |  |  | element present inside the gene |
| 23 | ATTGGGCCACG found at 2687029 line | FHAD1 | [114827 (FHAD1)](http://www.ncbi.nlm.nih.gov/gene/114827) | forkhead-associated (FHA) phosphopeptide binding domain 1 | 15702448 .. 15702458 | 15,573,730..15,724,767 |  |  | element present inside the gene |
| 24 | ATTGGGCCACG found at 3413412 line | PRAMEF5 | [343068 (PRAMEF5)](http://www.ncbi.nlm.nih.gov/gene/343068) | PRAME family member 5 | 13346283 .. 13346293 | 13,359,819..13,369,057 |  | 13536 |  |
| 25 | ATTGGTCCACG found at 444164 line | 0 |  |  |  |  |  |  |  |
| 26 | ATTGGTCCACG found at 1232536 line | SRGAP2 (Partial stop) | [23380 (SRGAP2)](http://www.ncbi.nlm.nih.gov/gene/23380) | SLIT-ROBO Rho GTPase activating protein 2 | 206620702 .. 206620712 | 206,516,197..206,637,783 |  |  | element present inside the gene |
| 27 | ATTGGTCCACG found at 1428703 line | 0 |  |  |  |  |  |  |  |
| 28 | ATTGGTCCACG found at 1485311 line | 0 |  |  |  |  |  |  |  |
| 29 | ATTGGTCCACG found at 1672503 line | 0 |  |  |  |  |  |  |  |
| 30 | ATTGGTCCACG found at 2297405 line | 0 |  |  |  |  |  |  |  |
| 31 | ATTGGTCCACG found at 2555339 line | 0 |  |  |  |  |  |  |  |
| 32 | ATTGGTCCACG found at 2869734 line | 0 |  |  |  |  |  |  |  |
| 33 | ATTGGTCCACG found at 3189285 line | 0 |  |  |  |  |  |  |  |
|  | chrom 2-----34 match |  |  |  |  |  |  |  |  |
| 34 | ATTGGTCCACG found at 4803 line | COPS8 | [10920 (COPS8)](http://www.ncbi.nlm.nih.gov/gene/10920) | COP9 signalosome subunit 8 | 237972967 .. 237972977 | 237,993,870..238,007,489 |  | 20903 |  |
| 35 | ATTGGTCCACG found at 219909 line | MARCH7 | [64844 (MARCH7)](http://www.ncbi.nlm.nih.gov/gene/64844) | membrane-associated ring finger (C3HC4) 7, E3 ubiquitin protein ligase | 160524257 .. 160524267 | 160,568,980..160,625,084 |  |  | 44723 |
| 36 | ATTGGTCCACG found at 698545 line | 0 |  |  |  |  |  |  |  |
| 37 | ATTGGTCCACG found at 897344 line | ANKRD36 | [375248 (ANKRD36)](http://www.ncbi.nlm.nih.gov/gene/375248) | ankyrin repeat domain 36 | 97919499 .. 97919509 | 97,778,923..97,930,258 |  |  | element present inside the gene |
| 38 | ATTGGTCCACG found at 1359995 line | 0 |  |  |  |  |  |  |  |
| 39 | ATTGGTCCACG found at 1806619 line | 0 |  |  |  |  |  |  |  |
| 40 | ATTGGTCCACG found at 2229505 line | 0 |  |  |  |  |  |  |  |
| 41 | ATTGGTCCACG found at 3305182 line | 0 |  |  |  |  |  |  |  |
| 42 | ATTGGACCACG found at 71703 line | 0 |  |  |  |  |  |  |  |
| 43 | ATTGGACCACG found at 422153 line | 0 |  |  |  |  |  |  |  |
| 44 | ATTGGACCACG found at 749491 line | ITGA4 | [3676 (ITGA4)](http://www.ncbi.nlm.nih.gov/gene/3676) | integrin, alpha 4 (antigen CD49D, alpha 4 subunit of VLA-4 receptor) | 182351100 .. 182351110 | 182,321,619..182,402,474 |  |  | element present inside the gene |
| 45 | ATTGGACCACG found at 1102365 line | 0 |  |  |  |  |  |  |  |
| 46 | ATTGGACCACG found at 2075773 line | EPC2 | [26122 (EPC2)](http://www.ncbi.nlm.nih.gov/gene/26122) | enhancer of polycomb homolog 2 (Drosophila) | 149455555 .. 149455565 | 149,402,560..149,545,136 |  |  | element present inside the gene |
| 47 | ATTGGACCACG found at 2237818 line | 0 |  |  |  |  |  |  |  |
| 48 | ATTGGACCACG found at 2532656 line | CHAC2 | [494143 (CHAC2)](http://www.ncbi.nlm.nih.gov/gene/494143) | ChaC, cation transport regulator homolog 2 (E. coli) | 53963244 .. 53963254 | 53,994,929..54,002,320 |  |  | 31685 |
| 49 | ATTGGACCACG found at 2619158 line | LBH | [81606 (LBH)](http://www.ncbi.nlm.nih.gov/gene/81606) | limb bud and heart development | 30394922 .. 30394932 | 30,454,397..30,482,899 |  |  | 59475 |
| 50 | ATTGGACCACG found at 2777623 line | 0 |  |  |  |  |  |  |  |
| 51 | ATTGGGCCACG found at 493547 line | 0 |  |  |  |  |  |  |  |
| 52 | ATTGGGCCACG found at 1516117 line | 0 |  |  |  |  |  |  |  |
| 53 | ATTGGGCCACG found at 2138803 line | FAM117B | [150864 (FAM117B)](http://www.ncbi.nlm.nih.gov/gene/150864) | family with sequence similarity 117, member B | 203561100 .. 203561110 | 203,499,901..203,634,480 |  |  | element present inside the gene |
| 54 | ATTGGGCCACG found at 2184621 line | ZNF804A | [91752 (ZNF804A)](http://www.ncbi.nlm.nih.gov/gene/91752) | zinc finger protein 804A | 185710568 .. 185710578 | 185,463,093..185,804,214 |  |  | element present inside the gene |
| 55 | ATTGGGCCACG found at 2579315 line | GPD2 | [2820 (GPD2)](http://www.ncbi.nlm.nih.gov/gene/2820) | glycerol-3-phosphate dehydrogenase 2 (mitochondrial) | 157292613 .. 157292623 | 157,291,965..157,442,915 |  |  | element present inside the gene |
| 56 | ATTGGGCCACG found at 2827239 line | 0 |  |  |  |  |  |  |  |
| 57 | ATTGGGCCACG found at 3267685 line | LIMS1 | [3987 (LIMS1)](http://www.ncbi.nlm.nih.gov/gene/3987) | LIM and senescent cell antigen-like domains 1 | 109160330 .. 109160340 | 109,150,811..109,303,702 |  |  | element present inside the gene |
| 58 | ATTGGGCCACG found at 3340381 line | 0 |  |  |  |  |  |  |  |
| 59 | ATTGGCCCACG found at 626808 line | DIS3L2 (Partial stop) | [129563 (DIS3L2)](http://www.ncbi.nlm.nih.gov/gene/129563) | DIS3 mitotic control homolog (S. cerevisiae)-like 2 | 233084368 .. 233084378 | 232,826,293..233,208,678 |  |  | element present inside the gene |
| 60 | ATTGGCCCACG found at 699238 line | 0 |  |  |  |  |  |  |  |
| 61 | ATTGGCCCACG found at 779776 line | 0 |  |  |  |  |  |  |  |
| 62 | ATTGGCCCACG found at 962581 line | PTCD3 | [55037 (PTCD3)](http://www.ncbi.nlm.nih.gov/gene/55037) | pentatricopeptide repeat domain 3 | 86362167 .. 86362177 | 86,333,305..86,369,280 |  |  | element present inside the gene |
| 63 | ATTGGCCCACG found at 1098696 line | 0 |  |  |  |  |  |  |  |
| 64 | ATTGGCCCACG found at 1199476 line | ANTXR1 | [84168 (ANTXR1)](http://www.ncbi.nlm.nih.gov/gene/84168) | anthrax toxin receptor 1 | 69305738 .. 69305748 | 69,240,276..69,476,459 |  |  | element present inside the gene |
| 65 | ATTGGCCCACG found at 1641690 line | 0 |  |  |  |  |  |  |  |
| 66 | ATTGGCCCACG found at 2328755 line | 0 |  |  |  |  |  |  |  |
| 67 | ATTGGCCCACG found at 3237285 line | 0 |  |  |  |  |  |  |  |
|  | chrom 3---------21 match |  |  |  |  |  |  |  |  |
| 68 | ATTGGTCCACG found at 230363 line | CCRL2 | [9034 (CCRL2)](http://www.ncbi.nlm.nih.gov/gene/9034) | chemokine (C-C motif) receptor-like 2 | 46442704 .. 46442714 | 46,448,721..46,454,488 | 6017 |  |  |
| 69 | ATTGGTCCACG found at 333663 line | 0 |  |  |  |  |  |  |  |
| 70 | ATTGGTCCACG found at 381371 line | 0 |  |  |  |  |  |  |  |
| 71 | ATTGGTCCACG found at 645039 line | 0 |  |  |  |  |  |  |  |
| 72 | ATTGGCCCACG found at 340560 line | 0 |  |  |  |  |  |  |  |
| 73 | ATTGGCCCACG found at 553145 line | 0 |  |  |  |  |  |  |  |
| 74 | ATTGGCCCACG found at 1849988 line | 0 |  |  |  |  |  |  |  |
| 75 | ATTGGCCCACG found at 1849989 line | 0 |  |  |  |  |  |  |  |
| 76 | ATTGGCCCACG found at 2458902 line | MYRIP | [25924 (MYRIP)](http://www.ncbi.nlm.nih.gov/gene/25924) | myosin VIIA and Rab interacting protein | 39826311 .. 39826321 | 39,850,405..40,301,812 |  |  | 24094 |
| 77 | ATTGGCCCACG found at 2700925 line | THRB-AS1 | [644990 (THRB-AS1)](http://www.ncbi.nlm.nih.gov/gene/644990) | THRB antisense RNA 1 | 24520170 .. 24520180 | 24,535,356..24,541,502 |  | 15186 |  |
| 78 | ATTGGGCCACG found at 442351 line | EEFSEC | [60678 (EEFSEC)](http://www.ncbi.nlm.nih.gov/gene/60678) | eukaryotic elongation factor, selenocysteine-tRNA-specific | 128123278 .. 128123288 | 127,872,302..128,127,489 |  |  | element present inside the gene |
| 79 | ATTGGGCCACG found at 804503 line | 0 |  |  |  |  |  |  |  |
| 80 | ATTGGGCCACG found at 1000778 line | 0 |  |  |  |  |  |  |  |
| 81 | ATTGGGCCACG found at 1779492 line | 0 |  |  |  |  |  |  |  |
| 82 | ATTGGACCACG found at 482425 line | 0 |  |  |  |  |  |  |  |
| 83 | ATTGGACCACG found at 680313 line | LPP | [4026 (LPP)](http://www.ncbi.nlm.nih.gov/gene/4026) | LIM domain containing preferred translocation partner in lipoma | 188280989 .. 188280999 | 187,871,097..188,608,460 |  |  | element present inside the gene |
| 84 | ATTGGACCACG found at 1206961 line | 0 |  |  |  |  |  |  |  |
| 85 | ATTGGACCACG found at 2067270 line | HPS3 | [84343 (HPS3)](http://www.ncbi.nlm.nih.gov/gene/84343) | Hermansky-Pudlak syndrome 3 | 148843329 .. 148843339 | 148,847,371..148,891,305 | 4042 |  |  |
| 86 | ATTGGACCACG found at 2532215 line | 0 |  |  |  |  |  |  |  |
| 87 | ATTGGACCACG found at 2615015 line | ARIH2 | [10425 (ARIH2)](http://www.ncbi.nlm.nih.gov/gene/10425) | ariadne homolog 2 (Drosophila) | 48982447 .. 48982457 | 48,956,265..49,022,974 |  |  | element present inside the gene |
| 88 | ATTGGACCACG found at 2732632 line | 0 |  |  |  |  |  |  |  |
|  | chrom 4----------15 match |  |  |  |  |  |  |  |  |
| 89 | ATTGGTCCACG found at 49761 line | 0 |  |  |  |  |  |  |  |
| 90 | ATTGGTCCACG found at 519146 line | 0 |  |  |  |  |  |  |  |
| 91 | ATTGGTCCACG found at 617102 line | 0 |  |  |  |  |  |  |  |
| 92 | ATTGGTCCACG found at 912949 line | 0 |  |  |  |  |  |  |  |
| 93 | ATTGGACCACG found at 90987 line | BANK1 | [55024 (BANK1)](http://www.ncbi.nlm.nih.gov/gene/55024) | B-cell scaffold protein with ankyrin repeats 1 | 102849910 .. 102849920 | 102,711,764..102,995,969 |  |  | element present inside the gene |
| 94 | ATTGGACCACG found at 190260 line | 0 |  |  |  |  |  |  |  |
| 95 | ATTGGACCACG found at 567399 line | 0 |  |  |  |  |  |  |  |
| 96 | ATTGGACCACG found at 1420420 line | 0 |  |  |  |  |  |  |  |
| 97 | ATTGGACCACG found at 1428473 line | MAN2B2 | [23324 (MAN2B2)](http://www.ncbi.nlm.nih.gov/gene/23324) | mannosidase, alpha, class 2B, member 2 | 6550969 .. 6550979 | 6,576,902..6,624,188 |  |  | 25933 |
| 98 | ATTGGCCCACG found at 441153 line | 0 |  |  |  |  |  |  |  |
| 99 | ATTGGCCCACG found at 526245 line | SLAIN2 | [57606 (SLAIN2)](http://www.ncbi.nlm.nih.gov/gene/57606) | SLAIN motif family, member 2 | 48318172 .. 48318182 | 48,343,497..48,428,218 |  |  | 25325 |
| 100 | ATTGGCCCACG found at 671087 line | TBC1D1 | [23216 (TBC1D1)](http://www.ncbi.nlm.nih.gov/gene/23216) | TBC1 (tre-2/USP6, BUB2, cdc16) domain family, member 1 | 37889484 .. 37889494 | 37,889,606..38,140,796 | 122 |  |  |
| 101 | ATTGGCCCACG found at 2582164 line | 0 |  |  |  |  |  |  |  |
| 102 | ATTGGGCCACG found at 1135422 line | 0 |  |  |  |  |  |  |  |
| 103 | ATTGGGCCACG found at 2646839 line | C4orf22 | [255119 (C4orf22)](http://www.ncbi.nlm.nih.gov/gene/255119) | chromosome 4 open reading frame 22 | 81750232 .. 81750242 | 81,256,861..81,884,910 |  |  | element present inside the gene |
|  | chrom 5-----------21 match |  |  |  |  |  |  |  |  |
| 104 | ATTGGTCCACG found at 41156 line | 0 |  |  |  |  |  |  |  |
| 105 | ATTGGTCCACG found at 1219504 line | 0 |  |  |  |  |  |  |  |
| 106 | ATTGGTCCACG found at 1800001 line | TENM2 | [57451 (TENM2)](http://www.ncbi.nlm.nih.gov/gene/57451) | teneurin transmembrane protein 2 | 167025056 .. 167025066 | 166,406,083..167,691,162 |  |  | element present inside the gene |
| 107 | ATTGGTCCACG found at 2319794 line | 0 |  |  |  |  |  |  |  |
| 108 | ATTGGTCCACG found at 2341249 line | 0 |  |  |  |  |  |  |  |
| 109 | ATTGGTCCACG found at 2446045 line | 0 |  |  |  |  |  |  |  |
| 110 | ATTGGCCCACG found at 376224 line | ZNF454 | [285676 (ZNF454)](http://www.ncbi.nlm.nih.gov/gene/285676) | zinc finger protein 454 | 178370200 .. 178370210 | 178,368,194..178,393,218 |  |  | element present inside the gene |
| 111 | ATTGGCCCACG found at 1315015 line | 0 |  |  |  |  |  |  |  |
| 112 | ATTGGCCCACG found at 1657801 line | 0 |  |  |  |  |  |  |  |
| 113 | ATTGGCCCACG found at 2398485 line | 0 |  |  |  |  |  |  |  |
| 114 | ATTGGCCCACG found at 2417164 line | 0 |  |  |  |  |  |  |  |
| 115 | ATTGGCCCACG found at 2463766 line | FAM81B | [153643 (FAM81B)](http://www.ncbi.nlm.nih.gov/gene/153643) | family with sequence similarity 81, member B | 94680995 .. 94681005 | 94,727,048..94,786,145 |  |  | 46053 |
| 116 | ATTGGCCCACG found at 2477366 line | 0 |  |  |  |  |  |  |  |
| 117 | ATTGGGCCACG found at 733916 line | 0 |  |  |  |  |  |  |  |
| 118 | ATTGGGCCACG found at 773771 line | 0 |  |  |  |  |  |  |  |
| 119 | ATTGGGCCACG found at 1117505 line | 0 |  |  |  |  |  |  |  |
| 120 | ATTGGGCCACG found at 2189221 line | RASGRF2 | [5924 (RASGRF2)](http://www.ncbi.nlm.nih.gov/gene/5924) | Ras protein-specific guanine nucleotide-releasing factor 2 | 80460258 .. 80460268 | 80,256,491..80,525,981 |  |  | element present inside the gene |
| 121 | ATTGGGCCACG found at 2223306 line | 0 |  |  |  |  |  |  |  |
| 122 | ATTGGGCCACG found at 2232028 line | NDUFS4 | [4724 (NDUFS4)](http://www.ncbi.nlm.nih.gov/gene/4724) | NADH dehydrogenase (ubiquinone) Fe-S protein 4, 18kDa (NADH-coenzyme Q reductase) | 52841840 .. 52841850 | 52,856,464..52,979,171 |  | 14624 |  |
| 123 | ATTGGACCACG found at 1624936 line | SCGB3A2 | [117156 (SCGB3A2)](http://www.ncbi.nlm.nih.gov/gene/117156) | secretoglobin, family 3A, member 2 | 147256727 .. 147256737 | 147,258,274..147,261,756 | 1547 |  |  |
| 124 | ATTGGACCACG found at 2045234 line | 0 |  |  |  |  |  |  |  |
|  | chrom 6-----------12 match |  |  |  |  |  |  |  |  |
| 125 | ATTGGTCCACG found at 56651 line | RNF146 (Partial stop) | [81847 (RNF146)](http://www.ncbi.nlm.nih.gov/gene/81847) | ring finger protein 146 | 127605341 .. 127605351 | 127,587,827..127,609,707 |  |  | element present inside the gene |
| 126 | ATTGGTCCACG found at 1526130 line | 0 |  |  |  |  |  |  |  |
| 127 | ATTGGTCCACG found at 1612315 line | 0 |  |  |  |  |  |  |  |
| 128 | ATTGGTCCACG found at 1615124 line | 0 |  |  |  |  |  |  |  |
| 129 | ATTGGTCCACG found at 1772298 line | C6orf201 | [404220 (C6orf201)](http://www.ncbi.nlm.nih.gov/gene/404220) | chromosome 6 open reading frame 201 | 4078717 .. 4078727 | 4,079,440..4,131,000 | 723 |  |  |
| 130 | ATTGGACCACG found at 92054 line | 0 |  |  |  |  |  |  |  |
| 131 | ATTGGACCACG found at 173693 line | LY86 | [9450 (LY86)](http://www.ncbi.nlm.nih.gov/gene/9450) | lymphocyte antigen 86 | 6627799 .. 6627809 | 6,588,934..6,655,216 |  |  | element present inside the gene |
| 132 | ATTGGGCCACG found at 141744 line | 0 |  |  |  |  |  |  |  |
| 133 | ATTGGGCCACG found at 908877 line | 0 |  |  |  |  |  |  |  |
| 134 | ATTGGGCCACG found at 1524997 line | 0 |  |  |  |  |  |  |  |
| 135 | ATTGGGCCACG found at 2021852 line | 0 |  |  |  |  |  |  |  |
| 136 | ATTGGGCCACG found at 2360719 line | 0 |  |  |  |  |  |  |  |
|  | chrom 7-------------26 match |  |  |  |  |  |  |  |  |
| 137 | ATTGGTCCACG found at 2211 line | 0 |  |  |  |  |  |  |  |
| 138 | ATTGGTCCACG found at 69504 line | 0 |  |  |  |  |  |  |  |
| 139 | ATTGGTCCACG found at 1123090 line | 0 |  |  |  |  |  |  |  |
| 140 | ATTGGTCCACG found at 1242125 line | 0 |  |  |  |  |  |  |  |
| 141 | ATTGGTCCACG found at 1440129 line | 0 |  |  |  |  |  |  |  |
| 142 | ATTGGTCCACG found at 1687170 line | 0 |  |  |  |  |  |  |  |
| 143 | ATTGGTCCACG found at 2007590 line | 0 |  |  |  |  |  |  |  |
| 144 | ATTGGGCCACG found at 369999 line | 0 |  |  |  |  |  |  |  |
| 145 | ATTGGGCCACG found at 547366 line | NRF1 | [4899 (NRF1)](http://www.ncbi.nlm.nih.gov/gene/4899) | nuclear respiratory factor 1 | 129357069 .. 129357079 | 129,251,555..129,396,922 |  |  | element present inside the gene |
| 146 | ATTGGGCCACG found at 603209 line | 0 |  |  |  |  |  |  |  |
| 147 | ATTGGGCCACG found at 948390 line | 0 |  |  |  |  |  |  |  |
| 148 | ATTGGGCCACG found at 1380024 line | 0 |  |  |  |  |  |  |  |
| 149 | ATTGGGCCACG found at 1386725 line | 0 |  |  |  |  |  |  |  |
| 150 | ATTGGGCCACG found at 1744824 line | HECW1 | [23072 (HECW1)](http://www.ncbi.nlm.nih.gov/gene/23072) | HECT, C2 and WW domain containing E3 ubiquitin protein ligase 1 | 43430958 .. 43430968 | 43,152,198..43,603,213 |  |  | element present inside the gene |
| 151 | ATTGGGCCACG found at 1796628 line | POU6F2 | [11281 (POU6F2)](http://www.ncbi.nlm.nih.gov/gene/11281) | POU class 6 homeobox 2 | 39410257 .. 39410267 | 39,017,609..39,504,390 |  |  | element present inside the gene |
| 152 | ATTGGGCCACG found at 2129412 line | 0 |  |  |  |  |  |  |  |
| 153 | ATTGGCCCACG found at 933314 line | 0 |  |  |  |  |  |  |  |
| 154 | ATTGGCCCACG found at 1029633 line | LRGUK | [136332 (LRGUK)](http://www.ncbi.nlm.nih.gov/gene/136332) | leucine-rich repeats and guanylate kinase domain containing | 133817219 .. 133817229 | 133,812,105..133,948,933 |  |  | element present inside the gene |
| 155 | ATTGGCCCACG found at 1354424 line | 0 |  |  |  |  |  |  |  |
| 156 | ATTGGCCCACG found at 1858574 line | GTF2I | [2969 (GTF2I)](http://www.ncbi.nlm.nih.gov/gene/2969) | general transcription factor IIi | 74133460 .. 74133470 | 74,071,991..74,175,022 |  |  | element present inside the gene |
| 157 | ATTGGCCCACG found at 2186092 line | 0 |  |  |  |  |  |  |  |
| 158 | ATTGGACCACG found at 990813 line | EN2 | [2020 (EN2)](http://www.ncbi.nlm.nih.gov/gene/2020) | engrailed homeobox 2 | 155242596 .. 155242606 | 155,250,824..155,257,526 | 8228 |  |  |
| 159 | ATTGGACCACG found at 1334672 line | 0 |  |  |  |  |  |  |  |
| 160 | ATTGGACCACG found at 1800880 line | 0 |  |  |  |  |  |  |  |
| 161 | ATTGGACCACG found at 1907438 line | 0 |  |  |  |  |  |  |  |
| 162 | ATTGGACCACG found at 2156149 line | 0 |  |  |  |  |  |  |  |
|  | chrom 8----------16 match |  |  |  |  |  |  |  |  |
| 163 | ATTGGGCCACG found at 85859 line | 0 |  |  |  |  |  |  |  |
| 164 | ATTGGGCCACG found at 798563 line | 0 |  |  |  |  |  |  |  |
| 165 | ATTGGGCCACG found at 1240816 line | 0 |  |  |  |  |  |  |  |
| 166 | ATTGGGCCACG found at 1713034 line | 0 |  |  |  |  |  |  |  |
| 167 | ATTGGGCCACG found at 1818715 line | 0 |  |  |  |  |  |  |  |
| 168 | ATTGGTCCACG found at 121619 line | 0 |  |  |  |  |  |  |  |
| 169 | ATTGGTCCACG found at 183941 line | 0 |  |  |  |  |  |  |  |
| 170 | ATTGGTCCACG found at 846500 line | 0 |  |  |  |  |  |  |  |
| 171 | ATTGGTCCACG found at 1083271 line | 0 |  |  |  |  |  |  |  |
| 172 | ATTGGTCCACG found at 1535970 line | 0 |  |  |  |  |  |  |  |
| 173 | ATTGGTCCACG found at 1621381 line | 0 |  |  |  |  |  |  |  |
| 174 | ATTGGCCCACG found at 173181 line | RDH10 | [157506 (RDH10)](http://www.ncbi.nlm.nih.gov/gene/157506) | retinol dehydrogenase 10 (all-trans) | 74200812 .. 74200822 | 74,206,837..74,237,520 | 6025 |  |  |
| 175 | ATTGGCCCACG found at 369116 line | 0 |  |  |  |  |  |  |  |
| 176 | ATTGGCCCACG found at 513544 line | 0 |  |  |  |  |  |  |  |
| 177 | ATTGGCCCACG found at 1030569 line | 0 |  |  |  |  |  |  |  |
| 178 | ATTGGACCACG found at 1527485 line | 0 |  |  |  |  |  |  |  |
|  | chrom 9---------20 match |  |  |  |  |  |  |  |  |
| 179 | ATTGGCCCACG found at 10165 line | 0 |  |  |  |  |  |  |  |
| 180 | ATTGGCCCACG found at 388817 line | COL27A1 | [85301 (COL27A1)](http://www.ncbi.nlm.nih.gov/gene/85301) | collagen, type XXVII, alpha 1 | 117035645 .. 117035655 | 116,918,231..117,072,975 |  |  | element present inside the gene |
| 181 | ATTGGCCCACG found at 465097 line | 0 |  |  |  |  |  |  |  |
| 182 | ATTGGCCCACG found at 1496592 line | ANKRD18B | [441459 (ANKRD18B)](http://www.ncbi.nlm.nih.gov/gene/441459) | ankyrin repeat domain 18B | 33486842 .. 33486852 | 33,524,347..33,602,658 |  |  | 37505 |
| 183 | ATTGGCCCACG found at 1625497 line | 0 |  |  |  |  |  |  |  |
| 184 | ATTGGCCCACG found at 1889510 line | KANK1 | [23189 (KANK1)](http://www.ncbi.nlm.nih.gov/gene/23189) | KN motif and ankyrin repeat domains 1 | 731780 .. 731790 | 470,294..746,106 |  |  | element present inside the gene |
| 185 | ATTGGGCCACG found at 133927 line | 0 |  |  |  |  |  |  |  |
| 186 | ATTGGGCCACG found at 492916 line | 0 |  |  |  |  |  |  |  |
| 187 | ATTGGGCCACG found at 600061 line | 0 |  |  |  |  |  |  |  |
| 188 | ATTGGGCCACG found at 630480 line | PCSK5 | [5125 (PCSK5)](http://www.ncbi.nlm.nih.gov/gene/5125) | proprotein convertase subtilisin/kexin type 5 | 78578075 .. 78578085 | 78,505,560..78,977,255 |  |  | element present inside the gene |
| 189 | ATTGGGCCACG found at 1091364 line | 0 |  |  |  |  |  |  |  |
| 190 | ATTGGGCCACG found at 1143771 line | 0 |  |  |  |  |  |  |  |
| 191 | ATTGGGCCACG found at 1634115 line | RUSC2 | [9853 (RUSC2)](http://www.ncbi.nlm.nih.gov/gene/9853) | RUN and SH3 domain containing 2 | 35489848 .. 35489858 | 35,490,007..35,561,895 | 159 |  |  |
| 192 | ATTGGGCCACG found at 1890952 line | 0 |  |  |  |  |  |  |  |
| 193 | ATTGGTCCACG found at 227638 line | 0 |  |  |  |  |  |  |  |
| 194 | ATTGGTCCACG found at 1220089 line | 0 |  |  |  |  |  |  |  |
| 195 | ATTGGTCCACG found at 1809548 line | 0 |  |  |  |  |  |  |  |
| 196 | ATTGGACCACG found at 1561741 line | GPR107 | [57720 (GPR107)](http://www.ncbi.nlm.nih.gov/gene/57720) | G protein-coupled receptor 107 | 132770967 .. 132770977 | 132,815,985..132,902,448 |  |  | 45018 |
| 197 | ATTGGACCACG found at 1674670 line | 0 |  |  |  |  |  |  |  |
| 198 | ATTGGACCACG found at 1844043 line | PALM2 | [114299 (PALM2)](http://www.ncbi.nlm.nih.gov/gene/114299) | paralemmin 2 | 112445246 .. 112445256 | 112,403,068..112,713,756 |  |  | element present inside the gene |
|  | chrom 10-----------11 match |  |  |  |  |  |  |  |  |
| 199 | ATTGGCCCACG found at 26454 line | 0 |  |  |  |  |  |  |  |
| 200 | ATTGGCCCACG found at 1140238 line | SLC18A2 | [6571 (SLC18A2)](http://www.ncbi.nlm.nih.gov/gene/6571) | solute carrier family 18 (vesicular monoamine transporter), member 2 | 119025958 .. 119025968 | 119,000,584..119,038,941 |  |  | element present inside the gene |
| 201 | ATTGGCCCACG found at 1653140 line | DYDC2 (Partial stop) | [84332 (DYDC2)](http://www.ncbi.nlm.nih.gov/gene/84332) | DPY30 domain containing 2 | 82097005 .. 82097015 | 82,104,501..82,127,829 | 7496 |  |  |
| 202 | ATTGGCCCACG found at 1775820 line | 0 |  |  |  |  |  |  |  |
| 203 | ATTGGACCACG found at 98805 line | 0 |  |  |  |  |  |  |  |
| 204 | ATTGGACCACG found at 148023 line | ZNF365 | [22891 (ZNF365)](http://www.ncbi.nlm.nih.gov/gene/22891) | zinc finger protein 365 | 64301081 .. 64301091 | 64,133,916..64,431,771 |  |  | element present inside the gene |
| 205 | ATTGGACCACG found at 893072 line | 0 |  |  |  |  |  |  |  |
| 206 | ATTGGACCACG found at 1852157 line | 0 |  |  |  |  |  |  |  |
| 207 | ATTGGTCCACG found at 775336 line | 0 |  |  |  |  |  |  |  |
| 208 | ATTGGTCCACG found at 924994 line | 0 |  |  |  |  |  |  |  |
| 209 | ATTGGGCCACG found at 1093404 line | 0 |  |  |  |  |  |  |  |
|  | chrom 11------------20 match |  |  |  |  |  |  |  |  |
| 210 | ATTGGGCCACG found at 40905 line | 0 |  |  |  |  |  |  |  |
| 211 | ATTGGGCCACG found at 41774 line | 0 |  |  |  |  |  |  |  |
| 212 | ATTGGGCCACG found at 338009 line | FDX1 | [2230 (FDX1)](http://www.ncbi.nlm.nih.gov/gene/2230) | ferredoxin 1 | 110290809 .. 110290819 | 110,300,661..110,335,608 | 9852 |  |  |
| 213 | ATTGGGCCACG found at 826405 line | 0 |  |  |  |  |  |  |  |
| 214 | ATTGGGCCACG found at 922590 line | 0 |  |  |  |  |  |  |  |
| 215 | ATTGGGCCACG found at 1317471 line | ENDOD1 | [23052 (ENDOD1)](http://www.ncbi.nlm.nih.gov/gene/23052) | endonuclease domain containing 1 | 94857799 .. 94857809 | 94,822,974..94,865,815 |  |  | element present inside the gene |
| 216 | ATTGGGCCACG found at 1477268 line | RBM4 | [5936 (RBM4)](http://www.ncbi.nlm.nih.gov/gene/5936) | RNA binding motif protein 4 | 66426360 .. 66426370 | 66,406,088..66,435,856 |  |  | element present inside the gene |
| 217 | ATTGGGCCACG found at 1516949 line | STX3 | [6809 (STX3)](http://www.ncbi.nlm.nih.gov/gene/6809) | syntaxin 3 | 59501035 .. 59501045 | 59,522,479..59,573,355 |  |  | 21444 |
| 218 | ATTGGGCCACG found at 1531818 line | 0 |  |  |  |  |  |  |  |
| 219 | ATTGGGCCACG found at 1815194 line | 0 |  |  |  |  |  |  |  |
| 220 | ATTGGGCCACG found at 1844348 line | SLC22A18 | [5002 (SLC22A18)](http://www.ncbi.nlm.nih.gov/gene/5002) | solute carrier family 22, member 18 | 2945010 .. 2945020 | 2,920,951..2,946,476 |  |  | element present inside the gene |
| 221 | ATTGGCCCACG found at 79292 line | UBASH3B | [84959 (UBASH3B)](http://www.ncbi.nlm.nih.gov/gene/84959) | ubiquitin associated and SH3 domain containing B | 122543419 .. 122543429 | 122,526,398..122,685,187 |  |  | element present inside the gene |
| 222 | ATTGGCCCACG found at 958520 line | 0 |  |  |  |  |  |  |  |
| 223 | ATTGGCCCACG found at 1701994 line | TRIM22 | [10346 (TRIM22)](http://www.ncbi.nlm.nih.gov/gene/10346) | tripartite motif containing 22 | 5708901 .. 5708911 | 5,710,817..5,732,093 | 1916 |  |  |
| 224 | ATTGGACCACG found at 375281 line | VPS11 | [55823 (VPS11)](http://www.ncbi.nlm.nih.gov/gene/55823) | vacuolar protein sorting 11 homolog (S. cerevisiae) | 118928091 .. 118928101 | 118,938,493..118,952,688 | 10402 |  |  |
| 225 | ATTGGACCACG found at 774178 line | VPS11 | [55823 (VPS11)](http://www.ncbi.nlm.nih.gov/gene/55823) | vacuolar protein sorting 11 homolog (S. cerevisiae) | 118927985 .. 118927995 | 118,938,493..118,952,688 | 10508 |  |  |
| 226 | ATTGGACCACG found at 895706 line | 0 |  |  |  |  |  |  |  |
| 227 | ATTGGACCACG found at 1277187 line | 0 |  |  |  |  |  |  |  |
| 228 | ATTGGACCACG found at 1651779 line | 0 |  |  |  |  |  |  |  |
| 229 | ATTGGACCACG found at 1651781 line | BBOX1 | [8424 (BBOX1)](http://www.ncbi.nlm.nih.gov/gene/8424) | butyrobetaine (gamma), 2-oxoglutarate dioxygenase (gamma-butyrobetaine hydroxylase) 1 | 27020133 .. 27020143 | 27,062,252..27,149,354 |  |  | 42119 |
|  | chrom 12------------14 match |  |  |  |  |  |  |  |  |
| 230 | ATTGGGCCACG found at 11678 line | 0 |  |  |  |  |  |  |  |
| 231 | ATTGGGCCACG found at 149560 line | 0 |  |  |  |  |  |  |  |
| 232 | ATTGGGCCACG found at 205315 line | 0 |  |  |  |  |  |  |  |
| 233 | ATTGGGCCACG found at 690946 line | 0 |  |  |  |  |  |  |  |
| 234 | ATTGGGCCACG found at 699669 line | SPATS2 | [65244 (SPATS2)](http://www.ncbi.nlm.nih.gov/gene/65244) | spermatogenesis associated, serine-rich 2 | 49748022 .. 49748032 | 49,760,688..49,921,208 |  | 12666 |  |
| 235 | ATTGGGCCACG found at 1096171 line | 0 |  |  |  |  |  |  |  |
| 236 | ATTGGGCCACG found at 1337102 line | 0 |  |  |  |  |  |  |  |
| 237 | ATTGGGCCACG found at 1339399 line | WNK1 | [65125 (WNK1)](http://www.ncbi.nlm.nih.gov/gene/65125) | WNK lysine deficient protein kinase 1 | 840725 .. 840735 | 861,759..1,020,618 |  |  | 21034 |
| 238 | ATTGGACCACG found at 188758 line | 0 |  |  |  |  |  |  |  |
| 239 | ATTGGCCCACG found at 788764 line | 0 |  |  |  |  |  |  |  |
| 240 | ATTGGCCCACG found at 1705585 line | 0 |  |  |  |  |  |  |  |
| 241 | ATTGGCCCACG found at 1755631 line | 0 |  |  |  |  |  |  |  |
| 242 | ATTGGTCCACG found at 1710922 line | 0 |  |  |  |  |  |  |  |
| 243 | ATTGGTCCACG found at 1711109 line | 0 |  |  |  |  |  |  |  |
|  | chrom 13---------7 match |  |  |  |  |  |  |  |  |
| 244 | ATTGGTCCACG found at 436001 line | HS6ST3 | [266722 (HS6ST3)](http://www.ncbi.nlm.nih.gov/gene/266722) | heparan sulfate 6-O-sulfotransferase 3 | 96834375 .. 96834385 | 96,743,093..97,491,816 |  |  | element present inside the gene |
| 245 | ATTGGTCCACG found at 707891 line | 0 |  |  |  |  |  |  |  |
| 246 | ATTGGTCCACG found at 1344924 line | 0 |  |  |  |  |  |  |  |
| 247 | ATTGGACCACG found at 1155659 line | 0 |  |  |  |  |  |  |  |
| 248 | ATTGGGCCACG found at 1436572 line | BIVM | [54841 (BIVM)](http://www.ncbi.nlm.nih.gov/gene/54841) | basic, immunoglobulin-like variable motif containing | 103433074 .. 103433084 | 103,451,399..103,493,888 |  | 18325 |  |
| 249 | ATTGGCCCACG found at 1444644 line | COL4A2 | [1284 (COL4A2)](http://www.ncbi.nlm.nih.gov/gene/1284) | collagen, type IV, alpha 2 | 111045430 .. 111045440 | 110,959,631..111,165,374 |  |  | element present inside the gene |
| 250 | ATTGGCCCACG found at 1542299 line | 0 |  |  |  |  |  |  |  |
|  | chrom 14----------17 match |  |  |  |  |  |  |  |  |
| 251 | ATTGGGCCACG found at 298224 line | 0 |  |  |  |  |  |  |  |
| 252 | ATTGGGCCACG found at 799206 line | 0 |  |  |  |  |  |  |  |
| 253 | ATTGGGCCACG found at 880883 line | 0 |  |  |  |  |  |  |  |
| 254 | ATTGGGCCACG found at 1300406 line | 0 |  |  |  |  |  |  |  |
| 255 | ATTGGGCCACG found at 1354853 line | 0 |  |  |  |  |  |  |  |
| 256 | ATTGGCCCACG found at 493209 line | CCNK | [8812 (CCNK)](http://www.ncbi.nlm.nih.gov/gene/8812) | cyclin K | 99947275 .. 99947285 | 99,947,695..99,977,852 | 420 |  |  |
| 257 | ATTGGCCCACG found at 538008 line | NRXN3 | [9369 (NRXN3)](http://www.ncbi.nlm.nih.gov/gene/9369) | neurexin 3 | 79219299 .. 79219309 | 78,636,716..80,334,633 |  |  | element present inside the gene |
| 258 | ATTGGCCCACG found at 1100270 line | 0 |  |  |  |  |  |  |  |
| 259 | ATTGGCCCACG found at 1388158 line | FAM177A1 | [283635 (FAM177A1)](http://www.ncbi.nlm.nih.gov/gene/283635) | family with sequence similarity 177, member A1 | 35510958 .. 35510968 | 35,514,113..35,552,589 | 3155 |  |  |
| 260 | ATTGGTCCACG found at 717403 line | 0 |  |  |  |  |  |  |  |
| 261 | ATTGGTCCACG found at 1082307 line | AHSA1 | [10598 (AHSA1)](http://www.ncbi.nlm.nih.gov/gene/10598) | AHA1, activator of heat shock 90kDa protein ATPase homolog 1 (yeast) | 77925979 .. 77925989 | 77,924,373..77,935,815 |  |  | element present inside the gene |
| 262 | ATTGGTCCACG found at 1211245 line | 0 |  |  |  |  |  |  |  |
| 263 | ATTGGACCACG found at 774295 line | CALM1 | [801 (CALM1)](http://www.ncbi.nlm.nih.gov/gene/801) | calmodulin 1 (phosphorylase kinase, delta) | 90858851 .. 90858861 | 90,863,327..90,874,619 | 4476 |  |  |
| 264 | ATTGGACCACG found at 789052 line | 0 |  |  |  |  |  |  |  |
| 265 | ATTGGACCACG found at 1103566 line | NRXN3 | [9369 (NRXN3)](http://www.ncbi.nlm.nih.gov/gene/9369) | neurexin 3 | 79456639 .. 79456649 | 78,636,716..80,334,633 |  |  | element present inside the gene |
| 266 | ATTGGACCACG found at 1198936 line | 0 |  |  |  |  |  |  |  |
| 267 | ATTGGACCACG found at 1261930 line | FBXO34 | [55030 (FBXO34)](http://www.ncbi.nlm.nih.gov/gene/55030) | F-box protein 34 | 55749120 .. 55749130 | 55,738,021..55,820,329 |  |  | element present inside the gene |
|  | chrom 15-------------13 match |  |  |  |  |  |  |  |  |
| 268 | ATTGGTCCACG found at 594677 line | SNAP23 | [8773 (SNAP23)](http://www.ncbi.nlm.nih.gov/gene/8773) | synaptosomal-associated protein, 23kDa | 42816592 .. 42816602 | 42,783,442..42,825,259 |  |  | element present inside the gene |
| 269 | ATTGGGCCACG found at 764787 line | IGF1R | [3480 (IGF1R)](http://www.ncbi.nlm.nih.gov/gene/3480) | insulin-like growth factor 1 receptor | 99332866 .. 99332876 | 99,192,272..99,507,759 |  |  | element present inside the gene |
| 270 | ATTGGGCCACG found at 959808 line | 0 |  |  |  |  |  |  |  |
| 271 | ATTGGGCCACG found at 984404 line | 0 |  |  |  |  |  |  |  |
| 272 | ATTGGGCCACG found at 1379625 line | 0 |  |  |  |  |  |  |  |
| 273 | ATTGGCCCACG found at 784928 line | ASB7 | [140460 (ASB7)](http://www.ncbi.nlm.nih.gov/gene/140460) | ankyrin repeat and SOCS box containing 7 | 101161062 .. 101161072 | 101,142,755..101,191,906 |  |  | element present inside the gene |
| 274 | ATTGGCCCACG found at 862696 line | 0 |  |  |  |  |  |  |  |
| 275 | ATTGGCCCACG found at 1131162 line | IL16 | [3603 (IL16)](http://www.ncbi.nlm.nih.gov/gene/3603) | interleukin 16 | 81443521 .. 81443531 | 81,474,941..81,605,104 |  |  | 31420 |
| 276 | ATTGGCCCACG found at 1306578 line | 0 |  |  |  |  |  |  |  |
| 277 | ATTGGCCCACG found at 1405016 line | TEX9 | [374618 (TEX9)](http://www.ncbi.nlm.nih.gov/gene/374618) | testis expressed 9 | 56514691 .. 56514701 | 56,536,207..56,739,032 |  |  | 21516 |
| 278 | ATTGGACCACG found at 839484 line | SV2B | [9899 (SV2B)](http://www.ncbi.nlm.nih.gov/gene/9899) | synaptic vesicle glycoprotein 2B | 91696532 .. 91696542 | 91,642,996..91,844,539 |  |  | element present inside the gene |
| 279 | ATTGGACCACG found at 1064562 line | 0 |  |  |  |  |  |  |  |
| 280 | ATTGGACCACG found at 1273565 line | 0 |  |  |  |  |  |  |  |
|  | chrom 16-----------14 match |  |  |  |  |  |  |  |  |
| 281 | ATTGGGCCACG found at 97369 line | BANP | [54971 (BANP)](http://www.ncbi.nlm.nih.gov/gene/54971) | BTG3 associated nuclear protein | 87991410 .. 87991420 | 87,984,231..88,110,924 |  |  | element present inside the gene |
| 282 | ATTGGGCCACG found at 243382 line | WFDC1 | [58189 (WFDC1)](http://www.ncbi.nlm.nih.gov/gene/58189) | WAP four-disulfide core domain 1 | 84323070 .. 84323080 | 84,328,244..84,363,450 | 5174 |  |  |
| 283 | ATTGGGCCACG found at 791195 line | 0 |  |  |  |  |  |  |  |
| 284 | ATTGGGCCACG found at 1170023 line | HERPUD1 | [9709 (HERPUD1)](http://www.ncbi.nlm.nih.gov/gene/9709) | homocysteine-inducible, endoplasmic reticulum stress-inducible, ubiquitin-like domain member 1 | 56965937 .. 56965947 | 56,965,974..56,977,793 | 37 |  |  |
| 285 | ATTGGGCCACG found at 1171155 line | 0 |  |  |  |  |  |  |  |
| 286 | ATTGGGCCACG found at 1222105 line | RBFOX1 | [54715 (RBFOX1)](http://www.ncbi.nlm.nih.gov/gene/54715) | RNA binding protein, fox-1 homolog (C. elegans) 1 | 7010479 .. 7010489 | 5,289,469..7,763,342 |  |  | element present inside the gene |
| 287 | ATTGGACCACG found at 118659 line | NUDT7 | [283927 (NUDT7)](http://www.ncbi.nlm.nih.gov/gene/283927) | nudix (nucleoside diphosphate linked moiety X)-type motif 7 | 77710305 .. 77710315 | 77,756,389..77,809,332 |  |  | 46084 |
| 288 | ATTGGACCACG found at 1079311 line | 0 |  |  |  |  |  |  |  |
| 289 | ATTGGCCCACG found at 233466 line | PLCG2 | [5336 (PLCG2)](http://www.ncbi.nlm.nih.gov/gene/5336) | phospholipase C, gamma 2 (phosphatidylinositol-specific) | 81869925 .. 81869935 | 81,812,899..81,996,290 |  |  | element present inside the gene |
| 290 | ATTGGCCCACG found at 311620 line | ALDOA | [226 (ALDOA)](http://www.ncbi.nlm.nih.gov/gene/226) | aldolase A, fructose-bisphosphate | 30045931 .. 30045941 | 30,064,411..30,081,741 |  | 18480 |  |
| 291 | ATTGGCCCACG found at 324192 line | SCNN1B | [6338 (SCNN1B)](http://www.ncbi.nlm.nih.gov/gene/6338) | sodium channel, non-voltage-gated 1, beta subunit | 23341685 .. 23341695 | 23,313,591..23,392,620 |  |  | element present inside the gene |
| 292 | ATTGGCCCACG found at 417306 line | 0 |  |  |  |  |  |  |  |
| 293 | ATTGGCCCACG found at 1137084 line | 0 |  |  |  |  |  |  |  |
| 294 | ATTGGTCCACG found at 851094 line | 0 |  |  |  |  |  |  |  |
|  | chrom 17-------------10 match |  |  |  |  |  |  |  |  |
| 295 | ATTGGCCCACG found at 91507 line | 0 |  |  |  |  |  |  |  |
| 296 | ATTGGCCCACG found at 932115 line | 0 |  |  |  |  |  |  |  |
| 297 | ATTGGACCACG found at 110131 line | 0 |  |  |  |  |  |  |  |
| 298 | ATTGGACCACG found at 461245 line | ALOX15B | [247 (ALOX15B)](http://www.ncbi.nlm.nih.gov/gene/247) | arachidonate 15-lipoxygenase, type B | 7929320 .. 7929330 | 7,942,343..7,952,452 |  | 13023 |  |
| 299 | ATTGGGCCACG found at 162509 line | 0 |  |  |  |  |  |  |  |
| 300 | ATTGGGCCACG found at 403527 line | (SEPT9) | [10801 (SEPT9)](http://www.ncbi.nlm.nih.gov/gene/10801) | septin 9 | 75388580 .. 75388590 | 75,277,492..75,496,678 |  |  | element present inside the gene |
| 301 | ATTGGGCCACG found at 533719 line | WIPF2 | [147179 (WIPF2)](http://www.ncbi.nlm.nih.gov/gene/147179) | WAS/WASL interacting protein family, member 2 | 38427610 .. 38427620 | 38,375,556..38,438,944 |  |  | element present inside the gene |
| 302 | ATTGGGCCACG found at 1047065 line | 0 |  |  |  |  |  |  |  |
| 303 | ATTGGGCCACG found at 1099784 line | DNAH9 | [1770 (DNAH9)](http://www.ncbi.nlm.nih.gov/gene/1770) | dynein, axonemal, heavy chain 9 | 11700546 .. 11700556 | 11,501,748..11,873,065 |  |  | element present inside the gene |
| 304 | ATTGGTCCACG found at 800714 line | DHX40 | [79665 (DHX40)](http://www.ncbi.nlm.nih.gov/gene/79665) | DEAH (Asp-Glu-Ala-His) box polypeptide 40 | 57651287 .. 57651297 | 57,642,886..57,685,713 |  |  | element present inside the gene |
|  | chrom 18----------6 match |  |  |  |  |  |  |  |  |
| 305 | ATTGGCCCACG found at 5109 line | 0 |  |  |  |  |  |  |  |
| 306 | ATTGGCCCACG found at 55912 line | 0 |  |  |  |  |  |  |  |
| 307 | ATTGGCCCACG found at 191790 line | 0 |  |  |  |  |  |  |  |
| 308 | ATTGGCCCACG found at 780541 line | 0 |  |  |  |  |  |  |  |
| 309 | ATTGGCCCACG found at 1053893 line | 0 |  |  |  |  |  |  |  |
| 310 | ATTGGACCACG found at 957658 line | 0 |  |  |  |  |  |  |  |
|  | chrom 19-----------14 match |  |  |  |  |  |  |  |  |
| 311 | ATTGGCCCACG found at 46768 line | 0 |  |  |  |  |  |  |  |
| 312 | ATTGGCCCACG found at 138181 line | ZNF473 | [25888 (ZNF473)](http://www.ncbi.nlm.nih.gov/gene/25888) | zinc finger protein 473 | 50542825 .. 50542835 | 50,528,786..50,552,033 |  |  | element present inside the gene |
| 313 | ATTGGCCCACG found at 613236 line | RUVBL2 | [10856 (RUVBL2)](http://www.ncbi.nlm.nih.gov/gene/10856) | RuvB-like 2 (E. coli) | 49496640 .. 49496650 | 49,496,738..49,519,183 | 98 |  |  |
| 314 | ATTGGCCCACG found at 641660 line | QPCTL | [54814 (QPCTL)](http://www.ncbi.nlm.nih.gov/gene/54814) | glutaminyl-peptide cyclotransferase-like | 46199395 .. 46199405 | 46,195,741..46,207,248 |  |  | element present inside the gene |
| 315 | ATTGGCCCACG found at 687455 line | 0 |  |  |  |  |  |  |  |
| 316 | ATTGGCCCACG found at 701985 line | PIN1 (Partial stop) | [5300 (PIN1)](http://www.ncbi.nlm.nih.gov/gene/5300) | peptidylprolyl cis/trans isomerase, NIMA-interacting 1 | 9948946 .. 9948956 | 9,945,883..9,960,365 |  |  | element present inside the gene |
| 317 | ATTGGCCCACG found at 794498 line | NFIC | [4782 (NFIC)](http://www.ncbi.nlm.nih.gov/gene/4782) | nuclear factor I/C (CCAAT-binding transcription factor) | 3367208 .. 3367218 | 3,359,561..3,469,215 |  |  | element present inside the gene |
| 318 | ATTGGGCCACG found at 188935 line | CRX | [1406 (CRX)](http://www.ncbi.nlm.nih.gov/gene/1406) | cone-rod homeobox | 48305810 .. 48305820 | 48,323,807..48,346,587 |  | 17997 |  |
| 319 | ATTGGGCCACG found at 231899 line | 0 |  |  |  |  |  |  |  |
| 320 | ATTGGGCCACG found at 670916 line | 0 |  |  |  |  |  |  |  |
| 321 | ATTGGACCACG found at 194989 line | RPS19 | [6223 (RPS19)](http://www.ncbi.nlm.nih.gov/gene/6223) | ribosomal protein S19 | 42363310 .. 42363320 | 42,363,988..42,375,484 | 678 |  |  |
| 322 | ATTGGACCACG found at 588381 line | CC2D1A | [54862 (CC2D1A)](http://www.ncbi.nlm.nih.gov/gene/54862) | coiled-coil and C2 domain containing 1A | 14039085 .. 14039095 | 14,016,956..14,041,693 |  |  | element present inside the gene |
| 323 | ATTGGTCCACG found at 589005 line | ZNF175 | [7728 (ZNF175)](http://www.ncbi.nlm.nih.gov/gene/7728) | zinc finger protein 175 | 52074921 .. 52074931 | 52,074,531..52,092,991 |  |  | element present inside the gene |
| 324 | ATTGGTCCACG found at 723264 line | ARHGEF1 | [9138 (ARHGEF1)](http://www.ncbi.nlm.nih.gov/gene/9138) | Rho guanine nucleotide exchange factor (GEF) 1 | 42408210 .. 42408220 | 42,387,240..42,411,604 |  |  | element present inside the gene |
|  | chrom 20-----------8 match |  |  |  |  |  |  |  |  |
| 325 | ATTGGGCCACG found at 71574 line | 0 |  |  |  |  |  |  |  |
| 326 | ATTGGGCCACG found at 521718 line | FAM83D | [81610 (FAM83D)](http://www.ncbi.nlm.nih.gov/gene/81610) | family with sequence similarity 83, member D | 37563563 .. 37563573 | 37,554,955..37,581,703 |  |  | element present inside the gene |
| 327 | ATTGGGCCACG found at 824169 line | CDS2 | [8760 (CDS2)](http://www.ncbi.nlm.nih.gov/gene/8760) | CDP-diacylglycerol synthase (phosphatidate cytidylyltransferase) 2 | 5153212 .. 5153222 | 5,107,407..5,178,533 |  |  | element present inside the gene |
| 328 | ATTGGTCCACG found at 169006 line | CDH4 | [1002 (CDH4)](http://www.ncbi.nlm.nih.gov/gene/1002) | cadherin 4, type 1, R-cadherin (retinal) | 60409102 .. 60409112 | 59,827,482..60,515,673 |  |  | element present inside the gene |
| 329 | ATTGGTCCACG found at 832818 line | CDH4 | [1002 (CDH4)](http://www.ncbi.nlm.nih.gov/gene/1002) | cadherin 4, type 1, R-cadherin (retinal) | 59962774 .. 59962784 | 59,827,482..60,515,673 |  |  | element present inside the gene |
| 330 | ATTGGTCCACG found at 839017 line | 0 |  |  |  |  |  |  |  |
| 331 | ATTGGCCCACG found at 505790 line | DNAJC5 | [80331 (DNAJC5)](http://www.ncbi.nlm.nih.gov/gene/80331) | DnaJ (Hsp40) homolog, subfamily C, member 5 | 62557912 .. 62557922 | 62,526,455..62,567,384 |  |  | element present inside the gene |
| 332 | ATTGGCCCACG found at 868862 line | CTNNBL1 | [56259 (CTNNBL1)](http://www.ncbi.nlm.nih.gov/gene/56259) | catenin, beta like 1 | 36416785 .. 36416795 | 36,322,357..36,500,531 |  |  | element present inside the gene |
|  | chrom 21------------5 match |  |  |  |  |  |  |  |  |
| 333 | ATTGGGCCACG found at 136607 line | 0 |  |  |  |  |  |  |  |
| 334 | ATTGGGCCACG found at 264563 line | 0 |  |  |  |  |  |  |  |
| 335 | ATTGGGCCACG found at 570204 line | 0 |  |  |  |  |  |  |  |
| 336 | ATTGGCCCACG found at 346564 line | 0 |  |  |  |  |  |  |  |
| 337 | ATTGGCCCACG found at 403778 line | 0 |  |  |  |  |  |  |  |
|  | chrom 22----------6 match |  |  |  |  |  |  |  |  |
| 338 | ATTGGGCCACG found at 324333 line | 0 |  |  |  |  |  |  |  |
| 339 | ATTGGACCACG found at 330279 line | 0 |  |  |  |  |  |  |  |
| 340 | ATTGGTCCACG found at 559562 line | GRAP2 | [9402 (GRAP2)](http://www.ncbi.nlm.nih.gov/gene/9402) | GRB2-related adaptor protein 2 | 40288332 .. 40288342 | 40,297,086..40,369,347 | 8754 |  |  |
| 341 | ATTGGCCCACG found at 570676 line | ZBED4 | [9889 (ZBED4)](http://www.ncbi.nlm.nih.gov/gene/9889) | zinc finger, BED-type containing 4 | 50222258 .. 50222268 | 50,247,497..50,283,726 |  |  | 25239 |
| 342 | ATTGGCCCACG found at 697533 line | 0 |  |  |  |  |  |  |  |
| 343 | ATTGGCCCACG found at 697533 line | 0 |  |  |  |  |  |  |  |
|  | chrom X----------18 match |  |  |  |  |  |  |  |  |
| 344 | ATTGGGCCACG found at 140577 line | 0 |  |  |  |  |  |  |  |
| 345 | ATTGGGCCACG found at 523765 line | CLCN4 | [1183 (CLCN4)](http://www.ncbi.nlm.nih.gov/gene/1183) | chloride channel, voltage-sensitive 4 | 10121437 .. 10121447 | 10,124,985..10,205,700 | 3548 |  |  |
| 346 | ATTGGCCCACG found at 305752 line | 0 |  |  |  |  |  |  |  |
| 347 | ATTGGCCCACG found at 479046 line | PCDH11X | [27328 (PCDH11X)](http://www.ncbi.nlm.nih.gov/gene/27328) | protocadherin 11 X-linked | 91764313 .. 91764323 | 91,034,260..91,878,229 |  |  | element present inside the gene |
| 348 | ATTGGCCCACG found at 1026588 line | 0 |  |  |  |  |  |  |  |
| 349 | ATTGGCCCACG found at 1082119 line | 0 |  |  |  |  |  |  |  |
| 350 | ATTGGCCCACG found at 1274506 line | 0 |  |  |  |  |  |  |  |
| 351 | ATTGGCCCACG found at 1513569 line | SMS | [6611 (SMS)](http://www.ncbi.nlm.nih.gov/gene/6611) | spermine synthase | 22014044 .. 22014054 | 21,958,691..22,025,798 |  |  | element present inside the gene |
| 352 | ATTGGTCCACG found at 348827 line | SLC6A8 | [6535 (SLC6A8)](http://www.ncbi.nlm.nih.gov/gene/6535) | solute carrier family 6 (neurotransmitter transporter), member 8 | 152940886 .. 152940896 | 152,953,752..152,962,048 |  | 12866 |  |
| 353 | ATTGGTCCACG found at 1145251 line | ZNF275 | [10838 (ZNF275)](http://www.ncbi.nlm.nih.gov/gene/10838) | zinc finger protein 275 | 152617631 .. 152617641 | 152,599,613..152,618,384 |  |  | element present inside the gene |
| 354 | ATTGGTCCACG found at 1169514 line | 0 |  |  |  |  |  |  |  |
| 355 | ATTGGTCCACG found at 1940217 line | 0 |  |  |  |  |  |  |  |
| 356 | ATTGGTCCACG found at 1988885 line | 0 |  |  |  |  |  |  |  |
| 357 | ATTGGTCCACG found at 2119691 line | 0 |  |  |  |  |  |  |  |
| 358 | ATTGGTCCACG found at 2124181 line | 0 |  |  |  |  |  |  |  |
| 359 | ATTGGACCACG found at 386488 line | 0 |  |  |  |  |  |  |  |
| 360 | ATTGGACCACG found at 1695790 line | 0 |  |  |  |  |  |  |  |
| 361 | ATTGGACCACG found at 2101084 line | MAGEB10 | [139422 (MAGEB10)](http://www.ncbi.nlm.nih.gov/gene/139422) | melanoma antigen family B, 10 | 27827028 .. 27827038 | 27,826,107..27,841,131 |  |  | element present inside the gene |
|  | chrom Y----------1 match |  |  |  |  |  |  |  |  |
| 362 | ATTGGGCCACG found at 268143 line | SURF6P1 (Pseudo) | [643470 (SURF6P1)](http://www.ncbi.nlm.nih.gov/gene/643470) | surfeit 6 pseudogene 1 | 19306155 .. 19306165 | 19,296,350..19,306,649 |  |  | element present inside the gene |
|  |  |  |  |  |  |  |  |  |  |
|  |  |  |  | **ERSE-III** |  |  |  |  |  |
|  | chrom 1-------27 match |  |  |  |  |  |  |  |  |
| 1 | CCAATGTCTGTTGAACAGCTATCACGTTGAACCACG found at 52800 line | DFFB | [1677 (DFFB)](http://www.ncbi.nlm.nih.gov/gene/1677) | DNA fragmentation factor, 40kDa, beta polypeptide (caspase-activated DNase) | 3801473 .. 3801508 | 3,773,845..3,801,993 |  |  | element present inside the gene |
| 2 | CCAATACTTGACAGCCCATTCATTACACCCGCCACG found at 69402 line | 0 |  |  |  |  |  |  |  |
| 3 | CCAATTCCCTTACGCGAGAGGAACACAAGCCCCACG found at 93035 line | 0 |  |  |  |  |  |  |  |
| 4 | CCAATTCTGCCACAGGCCCCGTCACCTTCTCCCACG found at 197504 line | KAZN | [23254 (KAZN)](http://www.ncbi.nlm.nih.gov/gene/23254) | kazrin, periplakin interacting protein | 14220183 .. 14220218 | 14,219,646..15,444,544 |  |  | element present inside the gene |
| 5 | CCAATTGCCTGGTAACAACATGTTCACACTTCCACG found at 243376 line | PADI1 | [29943 (PADI1)](http://www.ncbi.nlm.nih.gov/gene/29943) | peptidyl arginine deiminase, type I | 17522967 .. 17523002 | 17,531,621..17,572,501 | 8654 |  |  |
| 6 | CCAATGGGTGCAGTTCTGAGACTTTGGCCTTCCACG found at 254040 line | 0 |  |  |  |  |  |  |  |
| 7 | CCAATGGCCCCTGGAAGATAGGAGTCCACTTCCACG found at 273354 line | 0 |  |  |  |  |  |  |  |
| 8 | CCAATGACAGGCTGATACAGGCTACAAGAGCCCACG found at 308359 line | 0 |  |  |  |  |  |  |  |
| 9 | CCAATGAATACAATATCATCTCACAGATGAACCACG found at 423931 line | 0 |  |  |  |  |  |  |  |
| 10 | CCAATGCCTTTGGGACCTCCAAATGCAATTACCACG found at 541670 line | 0 |  |  |  |  |  |  |  |
| 11 | CCAATTAGGCACCCAGGACTATCCAATTTGGCCACG found at 609684 line | SZT2 | [23334 (SZT2)](http://www.ncbi.nlm.nih.gov/gene/23334) | seizure threshold 2 homolog (mouse) | 43897123 .. 43897158 | 43,855,556..43,919,918 |  |  | element present inside the gene |
| 12 | CCAATGTAATACTTGGACACAACAATATCTCCCACG found at 664780 line | 0 |  |  |  |  |  |  |  |
| 13 | CCAATGGTCTAGGGCATTCCAGGATATTCCACCACG found at 706291 line | 0 |  |  |  |  |  |  |  |
| 14 | CCAATTAGAATTACTTACAAGGTCATACATACCACG found at 710979 line | 0 |  |  |  |  |  |  |  |
| 15 | CCAATATCACACTTTGGCTCCCCAAAGATGTCCACG found at 1045304 line | 0 |  |  |  |  |  |  |  |
| 16 | CCAATGAAAAAGACCAAAGAGTTTCAATTAGCCACG found at 1567866 line | CTTNBP2NL | [55917 (CTTNBP2NL)](http://www.ncbi.nlm.nih.gov/gene/55917) | CTTNBP2 N-terminal like | 112886219 .. 112886254 | 112,938,800..113,003,786 |  | 52581 |  |
| 17 | CCAATGGGGAAGACATAGAGAATGAGGTGTTCCACG found at 1640174 line | VPS25P1 (Pseudo) | [441899 (VPS25P1)](http://www.ncbi.nlm.nih.gov/gene/441899) | vacuolar protein sorting 25 homolog (S. cerevisiae) pseudogene 1 | 118092423 .. 118092458 | 118,092,037..118,092,537 |  |  | element present inside the gene |
| 18 | CCAATCTACAGGCCCTTACTTTTCCCTCCTACCACG found at 2104632 line | TUFT1 | [7286 (TUFT1)](http://www.ncbi.nlm.nih.gov/gene/7286) | tuftelin 1 | 151533359 .. 151533394 | 151,512,781..151,556,059 |  |  | element present inside the gene |
| 19 | CCAATAGTAAGAAAATTAAATGACATAAACTCCACG found at 2118823 line | 0 |  |  |  |  |  |  |  |
| 20 | CCAATTTTCCCCAGGCAGCTGCGGTTGATGCCCACG found at 2128703 line | 0 |  |  |  |  |  |  |  |
| 21 | CCAATCTCCTGAGTCAACACTACCATGAAAACCACG found at 2148193 line | 0 |  |  |  |  |  |  |  |
| 22 | CCAATGTCCCTTAGGGGAGTAAAATTCCTCTCCACG found at 2393887 line | DNM3 | [26052 (DNM3)](http://www.ncbi.nlm.nih.gov/gene/26052) | dynamin 3 | 172359719 .. 172359754 | 171,810,618..172,387,606 |  |  | element present inside the gene |
| 23 | CCAATGTAGAAGCTGCTCATCTGGCCCAGGTCCACG found at 2983082 line | CENPF | [1063 (CENPF)](http://www.ncbi.nlm.nih.gov/gene/1063) | centromere protein F, 350/400kDa | 214781786 .. 214781821 | 214,776,522..214,837,914 |  |  | element present inside the gene |
| 24 | CCAATTCAAAAGCCCAGATCCTCCCACAGAACCACG found at 3014731 line | 0 |  |  |  |  |  |  |  |
| 25 | CCAATGCCATCTCAGTCCATCTCACAATGCCCCACG found at 3046436 line | LYPLAL1 | [127018 (LYPLAL1)](http://www.ncbi.nlm.nih.gov/gene/127018) | lysophospholipase-like 1 | 219343306 .. 219343341 | 219,347,192..219,386,207 | 3886 |  |  |
| 26 | CCAATCCATATGACCTTTTATTCTTTTTTCCCCACG found at 3162679 line | ZNF678 | [339500 (ZNF678)](http://www.ncbi.nlm.nih.gov/gene/339500) | zinc finger protein 678 | 227712788 .. 227712823 | 227,751,220..227,865,144 |  | 38432 |  |
| 27 | CCAATCTCAGCTTTTGGTCTCAGCCATCAAGCCACG found at 3182617 line | 0 |  |  |  |  |  |  |  |
|  | chrom 2----------32 match |  |  |  |  |  |  |  |  |
| 28 | CCAATAATGTCAGCACAAACCCAATTTCTTTCCACG found at 77917 line | 0 |  |  |  |  |  |  |  |
| 29 | CCAATCACCTCATCAGAGCAGGTGCTGGTATCCACG found at 92129 line | 0 |  |  |  |  |  |  |  |
| 30 | CCAATTTTTCATTAGGGCCTGCAACTCACAACCACG found at 117258 line | 0 |  |  |  |  |  |  |  |
| 31 | CCAATGTCTTCCCACAGCTTTATTTCCACAACCACG found at 224793 line | 0 |  |  |  |  |  |  |  |
| 32 | CCAATGGAACATCCCTCAGGCTGTCAATTGACCACG found at 308122 line | 0 |  |  |  |  |  |  |  |
| 33 | CCAATAGGTTTTGTGGAGGAGGAAGGAGGAGCCACG found at 355502 line | 0 |  |  |  |  |  |  |  |
| 34 | CCAATACCTCCACCAGAGCAGGTGCTGGTATCCACG found at 428410 line | LCLAT1 | [253558 (LCLAT1)](http://www.ncbi.nlm.nih.gov/gene/253558) | lysocardiolipin acyltransferase 1 | 30845428 .. 30845463 | 30,670,102..30,867,091 |  |  | element present inside the gene |
| 35 | CCAATCAACAGAGATCCATATCTTCGATAAGCCACG found at 769306 line | 0 |  |  |  |  |  |  |  |
| 36 | CCAATCCTATTCCTTTTCTAGCAGCAGGGTGCCACG found at 885727 line | MDH1 | [4190 (MDH1)](http://www.ncbi.nlm.nih.gov/gene/4190) | malate dehydrogenase 1, NAD (soluble) | 63772249 .. 63772284 | 63,815,743..63,834,331 |  |  | 43494 |
| 37 | CCAATATAGCTGGGACTACAGGCGCCCGCCTCCACG found at 892359 line | 0 |  |  |  |  |  |  |  |
| 38 | CCAATACTGCACAGCGGCCACTGTCCCTCTTCCACG found at 970682 line | 0 |  |  |  |  |  |  |  |
| 39 | CCAATCCGTGACCGGCGCCGGAGTTTTGGGTCCACG found at 990520 line | MPHOSPH10 | [10199 (MPHOSPH10)](http://www.ncbi.nlm.nih.gov/gene/10199) | M-phase phosphoprotein 10 (U3 small nucleolar ribonucleoprotein) | 71317352 .. 71317387 | 71,357,444..71,377,232 |  |  | 40092 |
| 40 | CCAATTGGGACCGCTGACTCGGGCTGGGTTCCCACG found at 1085867 line | 0 |  |  |  |  |  |  |  |
| 41 | CCAATCTGCAGTATTGATTTGAAAGATGATGCCACG found at 1388388 line | EIF5B | [9669 (EIF5B)](http://www.ncbi.nlm.nih.gov/gene/9669) | eukaryotic translation initiation factor 5B | 99963820 .. 99963855 | 99,953,821..100,016,728 |  |  | element present inside the gene |
| 42 | CCAATGGGATGTCACCCAGTGTCCAGCCTTGCCACG found at 1391505 line | 0 |  |  |  |  |  |  |  |
| 43 | CCAATAGGAATGGATGCTCCGACAGACGAAGCCACG found at 1470186 line | GPR45 | [11250 (GPR45)](http://www.ncbi.nlm.nih.gov/gene/11250) | G protein-coupled receptor 45 | 105853291 .. 105853326 | 105,839,595..105,860,085 |  |  | element present inside the gene |
| 44 | CCAATGGAGCGCGGTTCAGATCCCAGATCTACCACG found at 1470746 line | 0 |  |  |  |  |  |  |  |
| 45 | CCAATCTCTGGCTGTCCTTTCATTCTCGACTCCACG found at 1482575 line | 0 |  |  |  |  |  |  |  |
| 46 | CCAATGCTGGAGTAAGAGAAGAATCCTCAGACCACG found at 1513381 line | SULT1C4 | [27233 (SULT1C4)](http://www.ncbi.nlm.nih.gov/gene/27233) | sulfotransferase family, cytosolic, 1C, member 4 | 108963296 .. 108963331 | 108,994,413..109,004,296 |  |  | 31117 |
| 47 | CCAATTAGGCATCGTGAGCTCCACCAGCATGCCACG found at 1709725 line | 0 |  |  |  |  |  |  |  |
| 48 | CCAATCATAAATAAGATAAATGACCAATAACCCACG found at 1826239 line | AMER3 | [205147 (AMER3)](http://www.ncbi.nlm.nih.gov/gene/205147) | APC membrane recruitment protein 3 | 131489108 .. 131489143 | 131,513,077..131,525,707 |  |  | 23969 |
|  |  | GPR148 | [344561 (GPR148)](http://www.ncbi.nlm.nih.gov/gene/344561) | G protein-coupled receptor 148 | 131489108 .. 131489143 | 131,513,077..131,525,707 |  |  | gene present before the element |
| 49 | CCAATTAGCATTAAAATGTCATAATTAATTACCACG found at 1989272 line | 0 |  |  |  |  |  |  |  |
| 50 | CCAATGACTCTAAAGATTATGGAGATGATGACCACG found at 2272296 line | 0 |  |  |  |  |  |  |  |
| 51 | CCAATCTAAACTCCTCCACCCCCTGGCCATACCACG found at 2429388 line | 0 |  |  |  |  |  |  |  |
| 52 | CCAATAAGTAGCTGCTCTTATAACCAAGAAGCCACG found at 2817086 line | 0 |  |  |  |  |  |  |  |
| 53 | CCAATTCTCTGTCTTTTGTCCTTTAGAAAACCCACG found at 2820613 line | 0 |  |  |  |  |  |  |  |
| 54 | CCAATTATATGACATTTTGAAAAAGGCAAAACCACG found at 2918796 line | 0 |  |  |  |  |  |  |  |
| 55 | CCAATGGATTATTTGAAAAAGAGGAGCACACCCACG found at 3066010 line | 0 |  |  |  |  |  |  |  |
| 56 | CCAATAAAAGCCCCACATTCATCCTTCAAGTCCACG found at 3163457 line | RHBDD1 | [84236 (RHBDD1)](http://www.ncbi.nlm.nih.gov/gene/84236) | rhomboid domain containing 1 | 227768755 .. 227768790 | 227,700,652..227,863,926 |  |  | element present inside the gene |
| 57 | CCAATCACTGACCACTGGGTGTAAACACTTTCCACG found at 3193524 line | 0 |  |  |  |  |  |  |  |
| 58 | CCAATGGGCACAGCCCCACCCCTCTTCACGACCACG found at 3309491 line | 0 |  |  |  |  |  |  |  |
| 59 | CCAATCCTTGCTTCCAGGAACCCAGCAGCTCCCACG found at 3353745 line | DUSP28 | [285193 (DUSP28)](http://www.ncbi.nlm.nih.gov/gene/285193) | dual specificity phosphatase 28 | 241469485 .. 241469520 | 241,499,471..241,503,431 |  |  | 29986 |
|  | chrom 3-------------21 match |  |  |  |  |  |  |  |  |
| 60 | CCAATCACATAGATGATATTGCTAGACTCTGCCACG found at 27883 line | 0 |  |  |  |  |  |  |  |
| 61 | CCAATTCAGAAAAAACAGAAGGACTCATGGGCCACG found at 198272 line | 0 |  |  |  |  |  |  |  |
| 62 | CCAATGTCCATAGTATTTGCTCCTTCACTTACCACG found at 216807 line | BTD | [686 (BTD)](http://www.ncbi.nlm.nih.gov/gene/686) | biotinidase | 15609975 .. 15610010 | 15,642,864..15,689,147 |  |  | 32889 |
| 63 | CCAATTTTCATACTCTTGGAAGTCATATTATCCACG found at 436658 line | 0 |  |  |  |  |  |  |  |
| 64 | CCAATACGAACGCTACAGCTTCCGCAGCTTCCCACG found at 460497 line | CRTAP | [10491 (CRTAP)](http://www.ncbi.nlm.nih.gov/gene/10491) | cartilage associated protein | 33155647 .. 33155682 | 33,155,450..33,189,265 |  |  | element present inside the gene |
| 65 | CCAATCCTGAGTATAAACTTCTATAAATATGCCACG found at 551273 line | 0 |  |  |  |  |  |  |  |
| 66 | CCAATGCTTGAATTCAACCCAGATGAAAGTTCCACG found at 643210 line | 0 |  |  |  |  |  |  |  |
| 67 | CCAATGTTTATTGCAGCAAGATTCACGATAGCCACG found at 932504 line | 0 |  |  |  |  |  |  |  |
| 68 | CCAATTCTTGGTATTAATAATAGAATTTGATCCACG found at 1011034 line | 0 |  |  |  |  |  |  |  |
| 69 | CCAATTATGCATTCTGGCACTGGGGAAATGACCACG found at 1080330 line | 0 |  |  |  |  |  |  |  |
| 70 | CCAATTATGCATTCTGGCACTGGGGAAATGACCACG found at 1998986 line | 0 |  |  |  |  |  |  |  |
| 71 | CCAATACCTTCAGCTTAAAATATTCAATATGCCACG found at 1091977 line | 0 |  |  |  |  |  |  |  |
| 72 | CCAATTCTCTTTAGATGTCATTCCCCTGTTCCCACG found at 1476464 line | 0 |  |  |  |  |  |  |  |
| 73 | CCAATAATGAACTTATCCATATAACAAAATACCACG found at 1649701 line | 0 |  |  |  |  |  |  |  |
| 74 | CCAATATGTTTCTTACGTCGTGTGTTGAGAGCCACG found at 1665550 line | 0 |  |  |  |  |  |  |  |
| 75 | CCAATGGCCAGGGCGCCCCAGCCTTCCTGAGCCACG found at 1765864 line | 0 |  |  |  |  |  |  |  |
| 76 | CCAATTCTAATTATTTAGGGGGAAAAGAAATCCACG found at 2078068 line | RNF13 | [11342 (RNF13)](http://www.ncbi.nlm.nih.gov/gene/11342) | ring finger protein 13 | 149620797 .. 149620832 | 149,530,475..149,679,926 |  |  | element present inside the gene |
| 77 | CCAATAGTCAAGTTACAGAACATTCCTATCACCACG found at 2371521 line | 0 |  |  |  |  |  |  |  |
| 78 | CCAATATTTTTTTACTTACAGAAATTTTAGGCCACG found at 2483296 line | PIK3CA | [5290 (PIK3CA)](http://www.ncbi.nlm.nih.gov/gene/5290) | phosphatidylinositol-4,5-bisphosphate 3-kinase, catalytic subunit alpha | 178797182 .. 178797217 | 178,866,311..178,952,500 |  |  | 69129 |
| 79 | CCAATAGGTGCCGCGAAGCAGCAGCCCACCCCCACG found at 2556605 line | POLR2H | [5437 (POLR2H)](http://www.ncbi.nlm.nih.gov/gene/5437) | polymerase (RNA) II (DNA directed) polypeptide H | 184075410 .. 184075445 | 184,079,502..184,086,383 | 4092 |  |  |
| 80 | CCAATGACCACTTTTCTGGAAGACAGTTTTTCCACG found at 2616411 line | LPP | [4026 (LPP)](http://www.ncbi.nlm.nih.gov/gene/4026) | LIM domain containing preferred translocation partner in lipoma | 188381502 .. 188381537 | 187,871,097..188,608,460 |  |  | element present inside the gene |
|  | chrom 4-------------21 match |  |  |  |  |  |  |  |  |
| 81 | CCAATCGTGATCGCTACTTACAGAGCACCCGCCACG found at 62218 line | 0 |  |  |  |  |  |  |  |
| 82 | CCAATACAGCCCTGCTCATCTTATCGGGGGCCCACG found at 87938 line | 0 |  |  |  |  |  |  |  |
| 83 | CCAATGCGGCAGGCCCTCCACAACAGGGTCCCCACG found at 88244 line | 0 |  |  |  |  |  |  |  |
| 84 | CCAATGCAGCAGCCGCACTTCCCCGTCTGACCCACG found at 107519 line | SORCS2 | [57537 (SORCS2)](http://www.ncbi.nlm.nih.gov/gene/57537) | sortilin-related VPS10 domain containing receptor 2 | 7741230 .. 7741265 | 7,194,374..7,744,564 |  |  | element present inside the gene |
| 85 | CCAATTAAATGGGTACTGGAGGCAGCCATGGCCACG found at 314015 line | 0 |  |  |  |  |  |  |  |
| 86 | CCAATGGGGACATTGTGTGGGGGCTCCAAACCCACG found at 453496 line | 0 |  |  |  |  |  |  |  |
| 87 | CCAATCTGTGTCTTTGCTTTCACTTCATATGCCACG found at 624863 line | 0 |  |  |  |  |  |  |  |
| 88 | CCAATCCCACTCCACTCGCAGCCCTAGGCAACCACG found at 1242622 line | HERC3 | [8916 (HERC3)](http://www.ncbi.nlm.nih.gov/gene/8916) | HECT and RLD domain containing E3 ubiquitin protein ligase 3 | 89468665 .. 89468700 | 89,513,574..89,629,693 |  |  | 44909 |
| 89 | CCAATATTCCAAGCTATTTGGACACCAAAGTCCACG found at 1359822 line | 0 |  |  |  |  |  |  |  |
| 90 | CCAATGTTGTTCCCTCATTCTCCTATAAGCACCACG found at 1408325 line | 0 |  |  |  |  |  |  |  |
| 91 | CCAATTAAATCAGAATCTCTGGGGCTGAGACCCACG found at 1557758 line | 0 |  |  |  |  |  |  |  |
| 92 | CCAATTACATGGTCCATTTTAGAATTAAGTGCCACG found at 1712647 line | ADAD1 | [132612 (ADAD1)](http://www.ncbi.nlm.nih.gov/gene/132612) | adenosine deaminase domain containing 1 (testis-specific) | 123310429 .. 123310464 | 123,300,121..123,350,957 |  |  | element present inside the gene |
| 93 | CCAATCTGCTCAGGTTTGAATCCTAGCTGTACCACG found at 1809702 line | 0 |  |  |  |  |  |  |  |
| 94 | CCAATGTTTCCATTAATAGACAAGAGCACCACCACG found at 1953638 line | MGST2 | [4258 (MGST2)](http://www.ncbi.nlm.nih.gov/gene/4258) | microsomal glutathione S-transferase 2 | 140661831 .. 140661866 | 140,586,922..140,661,899 |  |  | element present inside the gene |
| 95 | CCAATGAATGCCCATTAGATCAGAGATTATGCCACG found at 1956538 line | 0 |  |  |  |  |  |  |  |
| 96 | CCAATTCTGCAAGGTAACAAGTGCTGCAGAACCACG found at 1957643 line | 0 |  |  |  |  |  |  |  |
| 97 | CCAATTATGCATTCTGGAACTGGGGAAATAACCACG found at 2175882 line | GUCY1B3 | [2983 (GUCY1B3)](http://www.ncbi.nlm.nih.gov/gene/2983) | guanylate cyclase 1, soluble, beta 3 | 156663347 .. 156663382 | 156,680,126..156,728,783 |  | 16779 |  |
| 98 | CCAATTCACACACTAAGGGAGGTTTTTCAAACCACG found at 2262864 line | 0 |  |  |  |  |  |  |  |
| 99 | CCAATTTGGAAAGCATATTTCAGGATATTATCCACG found at 2417159 line | GALNT7 | [51809 (GALNT7)](http://www.ncbi.nlm.nih.gov/gene/51809) | UDP-N-acetyl-alpha-D-galactosamine:polypeptide N-acetylgalactosaminyltransferase 7 (GalNAc-T7) | 174035301 .. 174035336 | 174,089,904..174,245,118 |  |  | 54603 |
| 100 | CCAATGGTATTGTAACATCAAACTGACAAGGCCACG found at 2469635 line | 0 |  |  |  |  |  |  |  |
| 101 | CCAATGACCGGAAGGGGTACAGGCAAGGAACCCACG found at 2590668 line | 0 |  |  |  |  |  |  |  |
|  | chrom 5--------------16 match |  |  |  |  |  |  |  |  |
| 102 | CCAATATTATCTTCATTTTGCCATCAACCTGCCACG found at 110561 line | 0 |  |  |  |  |  |  |  |
| 103 | CCAATGTTATTATCCTGAAATGGTATCATTACCACG found at 353791 line | 0 |  |  |  |  |  |  |  |
| 104 | CCAATCAAGGCTGCAGTGAGCTGTGATCATGCCACG found at 427546 line | 0 |  |  |  |  |  |  |  |
| 105 | CCAATCTGGTACCCACCCACCCACCCACCTACCACG found at 564994 line | PTGER4 | [5734 (PTGER4)](http://www.ncbi.nlm.nih.gov/gene/5734) | prostaglandin E receptor 4 (subtype EP4) | 40679469 .. 40679504 | 40,680,032..40,696,962 | 528 |  |  |
| 106 | CCAATCATCATTTCCAAATTCATGCCAAATGCCACG found at 934240 line | 0 |  |  |  |  |  |  |  |
| 107 | CCAATAACTTCATCTGGGAAGATAAAACTAACCACG found at 1197026 line | 0 |  |  |  |  |  |  |  |
| 108 | CCAATCTTAGCTCCCTAGTGACCATAATGAACCACG found at 1375717 line | 0 |  |  |  |  |  |  |  |
| 109 | CCAATTGTAAACAACCTAAAAATCCCCTCAGCCACG found at 1460331 line | 0 |  |  |  |  |  |  |  |
| 110 | CCAATCTGACCTTCCTGCCTTTCCTCTGTCCCCACG found at 1539808 line | 0 |  |  |  |  |  |  |  |
| 111 | CCAATAAAGCAGAATGGTTTGTGGAAATAACCCACG found at 1687305 line | ZNF474 | [133923 (ZNF474)](http://www.ncbi.nlm.nih.gov/gene/133923) | zinc finger protein 474 | 121485805 .. 121485840 | 121,465,215..121,489,266 |  |  | element present inside the gene |
|  |  | LOC100505841 | 100505841 (LOC100505841) | zinc finger protein 474-like | 121485805 .. 121485840 | 121,495,871..121,518,358 | 10066 |  |  |
| 112 | CCAATCCCCGCCACCTGTTCCTGTTTAAGGACCACG found at 1927496 line | 0 |  |  |  |  |  |  |  |
| 113 | CCAATAAGCTTCCTGGTTGTGAAGGTCTCTGCCACG found at 1952201 line | PCDHB@ | [56116 (PCDHB@)](http://www.ncbi.nlm.nih.gov/gene/56116) | protocadherin beta cluster | 140558384 .. 140558419 | 140,430,979..140,627,802 |  |  | element present inside the gene |
|  |  | PCDHB8 | [56128 (PCDHB8)](http://www.ncbi.nlm.nih.gov/gene/56128) | protocadherin beta 8 | 140558384 .. 140558419 | 140,557,371..140,560,081 |  |  | element present inside the gene |
|  |  | PCDHB16 | [57717 (PCDHB16)](http://www.ncbi.nlm.nih.gov/gene/57717) | protocadherin beta 16 | 140558384 .. 140558419 | 140,560,980..140,566,710 | 2596 |  |  |
|  |  | PCDHB9 | [56127 (PCDHB9)](http://www.ncbi.nlm.nih.gov/gene/56127) | protocadherin beta 9 | 140558384 .. 140558419 | 140,566,893..140,571,111 | 8384 |  |  |
|  |  | PCDHB10 | [56126 (PCDHB10)](http://www.ncbi.nlm.nih.gov/gene/56126) | protocadherin beta 10 | 140558384 .. 140558419 | 140,571,952..140,575,213 |  | 13568 |  |
|  |  | PCDHB11 | [56125 (PCDHB11)](http://www.ncbi.nlm.nih.gov/gene/56125) | protocadherin beta 11 | 140558384 .. 140558419 | 140,578,916..140,582,618 |  | 20532 |  |
|  |  | PCDHB7 | [56129 (PCDHB7)](http://www.ncbi.nlm.nih.gov/gene/56129) | protocadherin beta 7 | 140558384 .. 140558419 | 140,552,243..140,555,957 |  |  | gene present before the element |
|  |  | PCDHB12 | [56124 (PCDHB12)](http://www.ncbi.nlm.nih.gov/gene/56124) | protocadherin beta 12 | 140558384 .. 140558419 | 140,587,914..140,592,143 |  |  | 29530 |
|  |  | PCDHB13 | [56123 (PCDHB13)](http://www.ncbi.nlm.nih.gov/gene/56123) | protocadherin beta 13 | 140558384 .. 140558419 | 140,593,509..140,596,993 |  |  | 35125 |
|  |  | PCDHB14 | [56122 (PCDHB14)](http://www.ncbi.nlm.nih.gov/gene/56122) | protocadherin beta 14 | 140558384 .. 140558419 | 140,603,078..140,605,860 |  |  | 44694 |
|  |  | PCDHB15 | [56121 (PCDHB15)](http://www.ncbi.nlm.nih.gov/gene/56121) | protocadherin beta 15 | 140558384 .. 140558419 | 140,625,147..140,627,802 |  |  | 66763 |
| 114 | CCAATGAGCATTTCCTTTGAGTGTCATGTTGCCACG found at 2221392 line | 0 |  |  |  |  |  |  |  |
| 115 | CCAATAATTTGTAGTTGATAACTCATTCGACCCACG found at 2250230 line | 0 |  |  |  |  |  |  |  |
| 116 | CCAATTTCTCCAAGGGGAGAGGTTTATTAACCCACG found at 2256175 line | 0 |  |  |  |  |  |  |  |
| 117 | CCAATCTCTCAGAAAAACACTGCCTGAGAGTCCACG found at 2337504 line | 0 |  |  |  |  |  |  |  |
|  | chrom 6-----------17 match |  |  |  |  |  |  |  |  |
| 118 | CCAATTATTAGAAACATGCATATCAGGTGTCCCACG found at 124018 line | 0 |  |  |  |  |  |  |  |
| 119 | CCAATACATCATTTCTTTCTCCTAAGATCCTCCACG found at 186116 line | 0 |  |  |  |  |  |  |  |
| 120 | CCAATTACTTTGCCTCTCTGTGCCATAATTTCCACG found at 491280 line | PPARD | [5467 (PPARD)](http://www.ncbi.nlm.nih.gov/gene/5467) | peroxisome proliferator-activated receptor delta | 35372018 .. 35372053 | 35,310,335..35,395,968 |  |  | element present inside the gene |
| 121 | CCAATGCCATCAGAACTAATTAAAAACAGAGCCACG found at 707002 line | 0 |  |  |  |  |  |  |  |
| 122 | CCAATCACAGATGGAAGGCTAAAATGTTTGTCCACG found at 739060 line | 0 |  |  |  |  |  |  |  |
| 123 | CCAATTGTGTTGAGATTACAGGCATGAGCCACCACG found at 796243 line | 0 |  |  |  |  |  |  |  |
| 124 | CCAATATTGCAGGGTGTATACACCCCCCACCCCACG found at 813431 line | 0 |  |  |  |  |  |  |  |
| 125 | CCAATCATTCCACTTGTACTCATTTTCGACCCCACG found at 994427 line | 0 |  |  |  |  |  |  |  |
| 126 | CCAATGTAGCTGGAACTACAGGTGCCCACCACCACG found at 1059451 line | SENP6 | [26054 (SENP6)](http://www.ncbi.nlm.nih.gov/gene/26054) | SUMO1/sentrin specific peptidase 6 | 76280315 .. 76280350 | 76,311,596..76,427,997 |  |  | 31281 |
| 127 | CCAATGCCTTTACAAGCGAAGGTCGGAATAACCACG found at 1179688 line | 0 |  |  |  |  |  |  |  |
| 128 | CCAATCAGAGGTACTTTCAATATTCCATCTGCCACG found at 1546869 line | SLC16A10 | [117247 (SLC16A10)](http://www.ncbi.nlm.nih.gov/gene/117247) | solute carrier family 16 (aromatic amino acid transporter), member 10 | 111374481 .. 111374516 | 111,408,728..111,544,608 |  |  | 34247 |
| 129 | CCAATTTTATCAGACAGAGTTTATCTCCATTCCACG found at 1591740 line | 0 |  |  |  |  |  |  |  |
| 130 | CCAATTTCAGCCAAGTGATTAAGATCAACAACCACG found at 1670684 line | 0 |  |  |  |  |  |  |  |
| 131 | CCAATTCTGGACCATGAATCTAAATCTGGATCCACG found at 1718200 line | 0 |  |  |  |  |  |  |  |
| 132 | CCAATGTGTATTTTCCCAGCATAAATTGGATCCACG found at 1856257 line | EYA4 | [2070 (EYA4)](http://www.ncbi.nlm.nih.gov/gene/2070) | eyes absent homolog 4 (Drosophila) | 133650366 .. 133650401 | 133,561,512..133,853,258 |  |  | element present inside the gene |
| 133 | CCAATTATGCATTCTGGCACTGGGGAAATGACCACG found at 1958544 line | 0 |  |  |  |  |  |  |  |
| 134 | CCAATAGCTTGCTTTGTTATCCGGAGGGTAGCCACG found at 2359070 line | 0 |  |  |  |  |  |  |  |
|  | chrom 7------------15 match |  |  |  |  |  |  |  |  |
| 135 | CCAATGGTCAATTCTCAGTCTTCATCTTTCTCCACG found at 248438 line | 0 |  |  |  |  |  |  |  |
| 136 | CCAATGTCAATAGGGGACCTTTCCCCACTTTCCACG found at 388780 line | 0 |  |  |  |  |  |  |  |
| 137 | CCAATGTTCCCACCACCTCCAGGTGGGCTGCCCACG found at 414032 line | WIPF3 | [644150 (WIPF3)](http://www.ncbi.nlm.nih.gov/gene/644150) | WAS/WASL interacting protein family, member 3 | 29810191 .. 29810226 | 29,846,170..29,956,682 |  |  | 35979 |
| 138 | CCAATCATTTCAAACACTCTGGAGTCAAAGTCCACG found at 777775 line | ZNF713 | [349075 (ZNF713)](http://www.ncbi.nlm.nih.gov/gene/349075) | zinc finger protein 713 | 55999667 .. 55999702 | 55,955,148..56,008,519 |  |  | element present inside the gene |
|  |  | MRPS17 | [51373 (MRPS17)](http://www.ncbi.nlm.nih.gov/gene/51373) | mitochondrial ribosomal protein S17 | 55999667 .. 55999702 | 56,019,611..56,023,034 |  | 19944 |  |
| 139 | CCAATCAACTTCTGCCTTGAGTGACTCCAGGCCACG found at 1043738 line | 0 |  |  |  |  |  |  |  |
| 140 | CCAATACTCTGGATAACAGGATAACAGAGTGCCACG found at 1054890 line | 0 |  |  |  |  |  |  |  |
| 141 | CCAATATCACTCTCAGGTCCGGCGCAGTGGCCCACG found at 1399769 line | AP1S1 | [1174 (AP1S1)](http://www.ncbi.nlm.nih.gov/gene/1174) | adaptor-related protein complex 1, sigma 1 subunit | 100783275 .. 100783310 | 100,797,685..100,804,557 |  | 14410 |  |
| 142 | CCAATCTGCCTCAAACAACTGGTATTCTAAACCACG found at 1552034 line | 0 |  |  |  |  |  |  |  |
| 143 | CCAATCCTAGATGCTGCCGTGGGGTCGGAGCCCACG found at 1738950 line | 0 |  |  |  |  |  |  |  |
| 144 | CCAATCTCAGAAATGACACAAAGTCACTCTGCCACG found at 1776756 line | 0 |  |  |  |  |  |  |  |
| 145 | CCAATTTATGCTGTTAATCTTTCTCCCGTCTCCACG found at 1915784 line | 0 |  |  |  |  |  |  |  |
| 146 | CCAATATCTGGCTGCCCGGCTGCCCACGCCTCCACG found at 2071873 line | 0 |  |  |  |  |  |  |  |
| 147 | CCAATCACTGTGCCCAGAGAAGGCAGTGGAACCACG found at 2098383 line | WDR86-AS1 (Partial stop) | [100131176 (WDR86-AS1)](http://www.ncbi.nlm.nih.gov/gene/100131176) | WDR86 antisense RNA 1 | 151083441 .. 151083476 | 151,106,322..151,110,134 |  |  | 22881 |
| 148 | CCAATGCACCTCCTGGTCTGTACTTTCAAGACCACG found at 2148128 line | DPP6 (Partial start) | [1804 (DPP6)](http://www.ncbi.nlm.nih.gov/gene/1804) | dipeptidyl-peptidase 6 | 154665091 .. 154665126 | 154,400,205..154,685,995 |  |  | element present inside the gene |
| 149 | CCAATTCCCAGATAGTGAACAGTCACCCACTCCACG found at 2193541 line | 0 |  |  |  |  |  |  |  |
|  | chrom 8----------24 match |  |  |  |  |  |  |  |  |
| 150 | CCAATACTGCTGGGCAACCCAGGGAGACCAGCCACG found at 19933 line | DLGAP2 | [9228 (DLGAP2)](http://www.ncbi.nlm.nih.gov/gene/9228) | discs, large (Drosophila) homolog-associated protein 2 | 1435076 .. 1435111 | 877,021..1,656,642 |  |  | element present inside the gene |
| 151 | CCAATGGTCCGGCCTCCCTCCCTCTCCTTCCCCACG found at 84491 line | 0 |  |  |  |  |  |  |  |
| 152 | CCAATTACACCATACCCCATGCAAGATAGCACCACG found at 153806 line | MTMR9 | [66036 (MTMR9)](http://www.ncbi.nlm.nih.gov/gene/66036) | myotubularin related protein 9 | 11073897 .. 11073932 | 11,142,000..11,185,655 |  |  | 68103 |
| 153 | CCAATGAAAGCATGCAGTTTCCTGAAAGCTTCCACG found at 312221 line | 0 |  |  |  |  |  |  |  |
| 154 | CCAATCAGAGGTACTTTCAATTTTTCACCTGCCACG found at 343354 line | NEFM | [4741 (NEFM)](http://www.ncbi.nlm.nih.gov/gene/4741) | neurofilament, medium polypeptide | 24721402 .. 24721437 | 24,770,712..24,776,607 |  |  | 49310 |
| 155 | CCAATAGGCACTCCGTGTGGGGCCTCTGACCCCACG found at 466706 line | 0 |  |  |  |  |  |  |  |
| 156 | CCAATTTTTAACCCCAACAACCTTTGGAGCACCACG found at 520593 line | 0 |  |  |  |  |  |  |  |
| 157 | CCAATTTGTTCATCAGCTTATGACTATATGACCACG found at 551030 line | 0 |  |  |  |  |  |  |  |
| 158 | CCAATAAGTCATAACTAAGGTTATGACTTAACCACG found at 600566 line | 0 |  |  |  |  |  |  |  |
| 159 | CCAATATAAGGAACAAATCCCCTGGCTCTCCCCACG found at 769938 line | 0 |  |  |  |  |  |  |  |
| 160 | CCAATACTATTTTGGCATTTCGTAGCACCAACCACG found at 787291 line | TGS1 | [96764 (TGS1)](http://www.ncbi.nlm.nih.gov/gene/96764) | trimethylguanosine synthase 1 | 56684861 .. 56684896 | 56,685,791..56,738,007 | 930 |  |  |
| 161 | CCAATCACCCCCTCATTAAAGTGTTTACCTACCACG found at 802757 line | 0 |  |  |  |  |  |  |  |
| 162 | CCAATTCCAACCAGGGTTCATCCTCACTATTCCACG found at 976438 line | SULF1 | [23213 (SULF1)](http://www.ncbi.nlm.nih.gov/gene/23213) | sulfatase 1 | 70303413 .. 70303448 | 70,378,859..70,573,147 |  |  | 75446 |
| 163 | CCAATGAGAATCTGCGCGCAAATGAATAAAGCCACG found at 982558 line | 0 |  |  |  |  |  |  |  |
| 164 | CCAATTTAAAACTTTTAGAAAGAGCCCAGCTCCACG found at 1291313 line | 0 |  |  |  |  |  |  |  |
| 165 | CCAATTAAACAAAATATGGCAAAGCCTGAGACCACG found at 1363953 line | 0 |  |  |  |  |  |  |  |
| 166 | CCAATCAGTCTTCCAAACAAGCCATTGTGGGCCACG found at 1392307 line | VPS13B | [157680 (VPS13B)](http://www.ncbi.nlm.nih.gov/gene/157680) | vacuolar protein sorting 13 homolog B (yeast) | 100246002 .. 100246037 | 100,025,299..100,890,447 |  |  | element present inside the gene |
| 167 | CCAATCAGAGGGGTTTTCAATTTTTCATCTGCCACG found at 1590189 line | 0 |  |  |  |  |  |  |  |
| 168 | CCAATATCATTCTGAGCCATCTGGAAACCTGCCACG found at 1660813 line | 0 |  |  |  |  |  |  |  |
| 169 | CCAATTTCCCAGGTTCGCAGACTCCAGTTATCCACG found at 1755356 line | 0 |  |  |  |  |  |  |  |
| 170 | CCAATGTTGCTCTAGGTTCTGTGACAGTGACCCACG found at 1854167 line | 0 |  |  |  |  |  |  |  |
| 171 | CCAATTTTTAGAAAGTAGTATCCCACAAGGTCCACG found at 1912180 line | 0 |  |  |  |  |  |  |  |
| 172 | CCAATGTAGCTGGGAGTACAGGCACCCGCCACCACG found at 1970658 line | 0 |  |  |  |  |  |  |  |
| 173 | CCAATCCCTCGCCACGGCAGCCTCTGTCCTCCCACG found at 1980933 line | 0 |  |  |  |  |  |  |  |
|  | chrom 9----------24 match |  |  |  |  |  |  |  |  |
| 174 | CCAATGAAGCCATCTAAACTTGGGCTTTTTTCCACG found at 15433 line | 0 |  |  |  |  |  |  |  |
| 175 | CCAATAAAACAAAAGAAGTAATTCTATGAACCCACG found at 37297 line | KCNV2 | [169522 (KCNV2)](http://www.ncbi.nlm.nih.gov/gene/169522) | potassium channel, subfamily V, member 2 | 2685280 .. 2685315 | 2,717,526..2,730,037 |  |  | 32246 |
| 176 | CCAATGCTGAAGACTGACGAATGTCAACTGGCCACG found at 39610 line | 0 |  |  |  |  |  |  |  |
| 177 | CCAATTTTATCATCCATTAGAATGAGTTCTCCCACG found at 225838 line | 0 |  |  |  |  |  |  |  |
| 178 | CCAATAATCTTTTCTCCTGAAAGCACTGTCACCACG found at 311732 line | DMRTA1 | [63951 (DMRTA1)](http://www.ncbi.nlm.nih.gov/gene/63951) | DMRT-like family A1 | 22444568 .. 22444603 | 22,446,840..22,452,472 | 2272 |  |  |
| 179 | CCAATAATTCTAAATGTTTCTTCTAACAATCCCACG found at 486234 line | 0 |  |  |  |  |  |  | DNAJB5 DnaJ (Hsp40) homolog, subfamily B, member 5 - gene present before the element |
| 180 | CCAATGCTCCTGGGGCCTCCCTCCCCCCCGCCCACG found at 614377 line | 0 |  |  |  |  |  |  |  |
| 181 | CCAATAGAAAATATATGTATCAAGCAATTTCCCACG found at 1008405 line | MAMDC2 | [256691 (MAMDC2)](http://www.ncbi.nlm.nih.gov/gene/256691) | MAM domain containing 2 | 72605004 .. 72605039 | 72,658,435..72,841,899 |  |  | 53431 |
| 182 | CCAATGCCGTCTACTGGGCTGCTCGGCATGGCCACG found at 1253644 line | DAPK1 | [1612 (DAPK1)](http://www.ncbi.nlm.nih.gov/gene/1612) | death-associated protein kinase 1 | 90262227 .. 90262262 | 90,112,601..90,323,566 |  |  | element present inside the gene |
| 183 | CCAATTAAGAACATGGCACAGCTCCAAGCCTCCACG found at 1275999 line | 0 |  |  |  |  |  |  |  |
| 184 | CCAATTCCACATGTAGTGAGCTCTATCCTTACCACG found at 1287773 line | 0 |  |  |  |  |  |  |  |
| 185 | CCAATGACTCTCCTCTGGAGTGATAAAGAGTCCACG found at 1288815 line | 0 |  |  |  |  |  |  |  |
| 186 | CCAATGAGAACCTGGGGCTGACCCTCAAAGGCCACG found at 1326249 line | 0 |  |  |  |  |  |  |  |
| 187 | CCAATTCCCGCTGTGCCTGCCTCCCACCGTCCCACG found at 1330585 line | SUSD3 | [203328 (SUSD3)](http://www.ncbi.nlm.nih.gov/gene/203328) | sushi domain containing 3 | 95801982 .. 95802017 | 95,820,989..95,847,420 |  | 19007 |  |
| 188 | CCAATTCGTGTGCCAGAGTAAATATGTTCGTCCACG found at 1521096 line | 0 |  |  |  |  |  |  |  |
| 189 | CCAATATATTTCTTGTTGTATCATGATAGCACCACG found at 1535787 line | 0 |  |  |  |  |  |  |  |
| 190 | CCAATGTCATTGATGGGCATTTATGTTAACTCCACG found at 1573567 line | 0 |  |  |  |  |  |  |  |
| 191 | CCAATAATCTGAAGCTTCAGAAGTCACAGTTCCACG found at 1621746 line | ZNF618 | [114991 (ZNF618)](http://www.ncbi.nlm.nih.gov/gene/114991) | zinc finger protein 618 | 116765609 .. 116765644 | 116,638,562..116,818,875 |  |  | element present inside the gene |
| 192 | CCAATCTTATGTAAACAGAGCCTCTTAACGTCCACG found at 1623674 line | COL27A1 | [85301 (COL27A1)](http://www.ncbi.nlm.nih.gov/gene/85301) | collagen, type XXVII, alpha 1 | 116904396 .. 116904431 | 116,918,231..117,072,975 |  | 13835 |  |
| 193 | CCAATATAGTACAATTTTATTACCTATAGGCCCACG found at 1638306 line | (DEC1) | [50514 (DEC1)](http://www.ncbi.nlm.nih.gov/gene/50514) | deleted in esophageal cancer 1 | 117957925 .. 117957960 | 117,904,097..118,164,923 |  |  | element present inside the gene |
| 194 | CCAATGAGTCAGTGGCCCTGGAGTGCCAGAGCCACG found at 1850559 line | 0 |  |  |  |  |  |  |  |
| 195 | CCAATGGCCACTAAAATCATCAGAGCTGTGCCCACG found at 1892093 line | 0 |  |  |  |  |  |  |  |
| 196 | CCAATCTGGCTTCACGGCGGCGGAACTGACGCCACG found at 1896114 line | DBH | [1621 (DBH)](http://www.ncbi.nlm.nih.gov/gene/1621) | dopamine beta-hydroxylase (dopamine beta-monooxygenase) | 136520120 .. 136520155 | 136,501,485..136,524,466 |  |  | element present inside the gene |
| 197 | CCAATAATTCCAGCCACACAGCGTCAGGGAGCCACG found at 1943747 line | UAP1L1 | [91373 (UAP1L1)](http://www.ncbi.nlm.nih.gov/gene/91373) | UDP-N-acteylglucosamine pyrophosphorylase 1-like 1 | 139949627 .. 139949662 | 139,971,944..139,978,990 |  |  | 22317 |
|  | chrom 10------------23 match |  |  |  |  |  |  |  |  |
| 198 | CCAATCGATGTTGTGAGCGATATAGCATGAACCACG found at 45103 line | 0 |  |  |  |  |  |  |  |
| 199 | CCAATGTATTTTGCTTGTTAATGTCATTTACCCACG found at 49227 line | 0 |  |  |  |  |  |  |  |
| 200 | CCAATGTTGCCTTAGCTGGACGATAGAGTGGCCACG found at 89718 line | 0 |  |  |  |  |  |  |  |
| 201 | CCAATTGCTCTTCACTCCATTCCTGACCCTTCCACG found at 149469 line | 0 |  |  |  |  |  |  |  |
| 202 | CCAATGGACTGGTTCAGCAGCAAGAACATGGCCACG found at 199186 line | 0 |  |  |  |  |  |  |  |
| 203 | CCAATGAGGCAAGAATAGTGCCAAGGTTCAGCCACG found at 264191 line | 0 |  |  |  |  |  |  |  |
| 204 | CCAATCGATGCACACAGAAAACTCCTCTGGGCCACG found at 368128 line | GAD2 | [2572 (GAD2)](http://www.ncbi.nlm.nih.gov/gene/2572) | glutamate decarboxylase 2 (pancreatic islets and brain, 65kDa) | 26505122 .. 26505157 | 26,505,236..26,593,491 | 114 |  |  |
| 205 | CCAATTCTCCCTTCCTCCCATCTCCTGGCAACCACG found at 384087 line | 0 |  |  |  |  |  |  |  |
| 206 | CCAATGGTGGTTCAGAGAGGAGCCTCTAAACCCACG found at 390744 line | 0 |  |  |  |  |  |  |  |
| 207 | CCAATGAGATGTGAGCATCTATTATCGTGCCCCACG found at 395425 line | 0 |  |  |  |  |  |  |  |
| 208 | CCAATTACATCTGTGGCCCAGGCAAGGGAAGCCACG found at 413565 line | 0 |  |  |  |  |  |  |  |
| 209 | CCAATGAATCCTTTACCCGCAATGAGAATGTCCACG found at 650398 line | 0 |  |  |  |  |  |  |  |
| 210 | CCAATGAATCCTTTACCCGCAATGAGAATGTCCACG found at 657076 line | 0 |  |  |  |  |  |  |  |
| 211 | CCAATGAATCCTTTACCCGCAATGAGAATGTCCACG found at 680811 line | 0 |  |  |  |  |  |  |  |
| 212 | CCAATGATCCAACCAGCTGGAGCAGCAGCAACCACG found at 674203 line | 0 |  |  |  |  |  |  |  |
| 213 | CCAATACAGGGGAGGTCATCATAGGTAGAGCCCACG found at 934095 line | 0 |  |  |  |  |  |  |  |
| 214 | CCAATGGTAATAGGTAGTTATACTACAATATCCACG found at 1209084 line | 0 |  |  |  |  |  |  |  |
| 215 | CCAATGAATCCTTTACCTGAAATGAGAATGTCCACG found at 1238675 line | 0 |  |  |  |  |  |  |  |
| 216 | CCAATTCTTCTCAGAGGCCGAGAATATTCTTCCACG found at 1429999 line | 0 |  |  |  |  |  |  |  |
| 217 | CCAATGACTGCACCTTTGTCTTTGTACATGTCCACG found at 1572312 line | 0 |  |  |  |  |  |  |  |
| 218 | CCAATAAATGGCTAAATCAGGAGTTACTATTCCACG found at 1575890 line | 0 |  |  |  |  |  |  |  |
| 219 | CCAATCTGCCAGGCCTCTGTGCCCTCACTGCCCACG found at 1854419 line | 0 |  |  |  |  |  |  |  |
| 220 | CCAATGTGGCAGCTCCCCCGTCCTGGGACATCCACG found at 1868937 line | INPP5A | [3632 (INPP5A)](http://www.ncbi.nlm.nih.gov/gene/3632) | inositol polyphosphate-5-phosphatase, 40kDa | 134563363 .. 134563398 | 134,351,283..134,596,984 |  |  | element present inside the gene |
|  | chrom 11-----------20 match |  |  |  |  |  |  |  |  |
| 221 | CCAATGGGAGCCGTGAGGAATGCTACTGGGGCCACG found at 4044 line | ATHL1 | [80162 (ATHL1)](http://www.ncbi.nlm.nih.gov/gene/80162) | ATH1, acid trehalase-like 1 (yeast) | 291064 .. 291099 | 288,480..297,511 |  |  | element present inside the gene |
| 222 | CCAATTGGGTTCCCGCCCATGTTATTGGCCCCCACG found at 89045 line | SMPD1 | [6609 (SMPD1)](http://www.ncbi.nlm.nih.gov/gene/6609) | sphingomyelin phosphodiesterase 1, acid lysosomal | 6411101 .. 6411136 | 6,411,644..6,416,228 | 543 |  |  |
| 223 | CCAATTTTTCTATTTTTAATACAGGGTTTCACCACG found at 254980 line | GTF2H1 | [2965 (GTF2H1)](http://www.ncbi.nlm.nih.gov/gene/2965) | general transcription factor IIH, polypeptide 1, 62kDa | 18358465 .. 18358500 | 18,343,816..18,388,590 |  |  | element present inside the gene |
| 224 | CCAATTCTTCCTAGCTCTGGAGCCTACCATGCCACG found at 415830 line | 0 |  |  |  |  |  |  |  |
| 225 | CCAATACTCCATCCTGCAATTCACCATCCTTCCACG found at 535954 line | 0 |  |  |  |  |  |  |  |
| 226 | CCAATGGTGCAATCTTGGCTCACTGCAACCTCCACG found at 769131 line | 0 |  |  |  |  |  |  |  |
| 227 | CCAATGACTTACTAGTTACTCACTGACTTGACCACG found at 815221 line | GLYATL1 | [92292 (GLYATL1)](http://www.ncbi.nlm.nih.gov/gene/92292) | glycine-N-acyltransferase-like 1 | 58695778 .. 58695813 | 58,695,102..58,724,543 |  |  | element present inside the gene |
| 228 | CCAATCTCAAAGTATTGCACACTGCATGACTCCACG found at 840465 line | MS4A15 (Partial stop) | [219995 (MS4A15)](http://www.ncbi.nlm.nih.gov/gene/219995) | membrane-spanning 4-domains, subfamily A, member 15 | 60513371 .. 60513406 | 60,524,340..60,544,204 | 10969 |  |  |
| 229 | CCAATCAAACTCCACGCCTCCACCCGAGGCGCCACG found at 843334 line | 0 |  |  |  |  |  |  |  |
| 230 | CCAATGCGGCTGCCAAGACCACGGCCAGCAACCACG found at 887292 line | FLRT1 | [23769 (FLRT1)](http://www.ncbi.nlm.nih.gov/gene/23769) | fibronectin leucine rich transmembrane protein 1 | 63884902 .. 63884937 | 63,803,442..63,886,655 |  |  | element present inside the gene |
| 231 | CCAATGACCAAATAAGTAAAGAGGGGAGCCACCACG found at 1203649 line | 0 |  |  |  |  |  |  |  |
| 232 | CCAATTACGCACTCTGAATCTGGGGAAATAACCACG found at 1258812 line | 0 |  |  |  |  |  |  |  |
| 233 | CCAATCATAAATGGATATCCAAGACTGAAGTCCACG found at 1543365 line | C11orf53 | [341032 (C11orf53)](http://www.ncbi.nlm.nih.gov/gene/341032) | chromosome 11 open reading frame 53 | 111122162 .. 111122197 | 111,126,707..111,156,973 | 4545 |  |  |
| 234 | CCAATGGCCCTGCTGACCCTGTAGATGTCCACCACG found at 1566127 line | 0 |  |  |  |  |  |  |  |
| 235 | CCAATGCATGGAGCAGGTACAGTGCCTACCGCCACG found at 1703347 line | UBASH3B | [84959 (UBASH3B)](http://www.ncbi.nlm.nih.gov/gene/84959) | ubiquitin associated and SH3 domain containing B | 122640835 .. 122640870 | 122,526,398..122,685,187 |  |  | element present inside the gene |
| 236 | CCAATCAGGGTTATCAAGCCCAGGTTCCCCACCACG found at 1727966 line | 0 |  |  |  |  |  |  |  |
| 237 | CCAATTTATGATACTTACTGAGACCTTGGCACCACG found at 1756566 line | 0 |  |  |  |  |  |  |  |
| 238 | CCAATTTCCTCACTTCTCCTTGGGGAGAATGCCACG found at 1758120 line | 0 |  |  |  |  |  |  |  |
| 239 | CCAATCTCCTTAGCTGGCCAATCTCCCTCTCCCACG found at 1760607 line | 0 |  |  |  |  |  |  |  |
| 240 | CCAATGTGAGAAAGACATGAGATCTGGGGAGCCACG found at 1797530 line | 0 |  |  |  |  |  |  |  |
|  | chrom 12---------25 match |  |  |  |  |  |  |  |  |
| 241 | CCAATCTCAGTTCCAAAACGAAAAGGGATGGCCACG found at 366082 line | SSPN | [8082 (SSPN)](http://www.ncbi.nlm.nih.gov/gene/8082) | sarcospan | 26357798 .. 26357833 | 26,348,032..26,387,710 |  |  | element present inside the gene |
| 242 | CCAATGGCGCCATCTCAGTTCACTGCAACCTCCACG found at 459192 line | 0 |  |  |  |  |  |  |  |
| 243 | CCAATTCCCACTTTTCATCACCCCCTGGCAACCACG found at 558843 line | 0 |  |  |  |  |  |  |  |
| 244 | CCAATGCAATTGTGAAACTTGAAACCAGGACCCACG found at 569322 line | 0 |  |  |  |  |  |  |  |
| 245 | CCAATCCAGAAATACCTGACTCCACAGACAGCCACG found at 636053 line | ANO6 | [196527 (ANO6)](http://www.ncbi.nlm.nih.gov/gene/196527) | anoctamin 6 | 45795680 .. 45795715 | 45,609,770..45,834,187 |  |  | element present inside the gene |
| 246 | CCAATTGACTCCACCTGTGCCTCACTTTCTCCCACG found at 689736 line | TUBA1C | [84790 (TUBA1C)](http://www.ncbi.nlm.nih.gov/gene/84790) | tubulin, alpha 1c | 49660860 .. 49660895 | 49,621,715..49,667,117 |  |  | element present inside the gene |
| 247 | CCAATGCCGCCAGTGTTCTAGCCCCTTTCTTCCACG found at 751260 line | 0 |  |  |  |  |  |  |  |
| 248 | CCAATTTTCCAATATATTTATCGAAAAAAATCCACG found at 960062 line | NUP107 | [57122 (NUP107)](http://www.ncbi.nlm.nih.gov/gene/57122) | nucleoporin 107kDa | 69124365 .. 69124400 | 69,080,731..69,136,473 |  |  | element present inside the gene |
| 249 | CCAATAAGCACCCTTGCAACACCACCCCCATCCACG found at 1035824 line | 0 |  |  |  |  |  |  |  |
| 250 | CCAATGGGTCAGTTGCTATAGAACAACAACACCACG found at 1091754 line | NAV3 | [89795 (NAV3)](http://www.ncbi.nlm.nih.gov/gene/89795) | neuron navigator 3 | 78606149 .. 78606184 | 78,224,685..78,606,790 |  |  | element present inside the gene |
| 251 | CCAATGAAACAGAATATACCCAGAAATAAAGCCACG found at 1106586 line | SYT1 | [6857 (SYT1)](http://www.ncbi.nlm.nih.gov/gene/6857) | synaptotagmin I | 79674093 .. 79674128 | 79,257,773..79,845,788 |  |  | element present inside the gene |
| 252 | CCAATATACCTCAGCCTACACATGTGAATGCCCACG found at 1200653 line | 0 |  |  |  |  |  |  |  |
| 253 | CCAATCCTATTGACTACTTGCCTTCCCCATGCCACG found at 1242269 line | 0 |  |  |  |  |  |  |  |
| 254 | CCAATTTCCACTGGCAGGGCTGCAGCTGCTTCCACG found at 1255161 line | 0 |  |  |  |  |  |  |  |
| 255 | CCAATTCAGGGGTCCTTGATCAGACAACCCTCCACG found at 1482168 line | TCP11L2 | [255394 (TCP11L2)](http://www.ncbi.nlm.nih.gov/gene/255394) | t-complex 11, testis-specific-like 2 | 106715955 .. 106715990 | 106,696,570..106,741,365 |  |  | element present inside the gene |
| 256 | CCAATACCTTTGTTTGAGTAGCCCATCGTTTCCACG found at 1497107 line | BTBD11 | [121551 (BTBD11)](http://www.ncbi.nlm.nih.gov/gene/121551) | BTB (POZ) domain containing 11 | 107791617 .. 107791652 | 107,712,197..108,053,419 |  |  | element present inside the gene |
| 257 | CCAATGAATGTATAGACCACAGCTTGTTTGTCCACG found at 1518557 line | 0 |  |  |  |  |  |  |  |
| 258 | CCAATTTCAGCCTTCAGTTTTCTCAGAAATCCCACG found at 1526883 line | UBE3B | [89910 (UBE3B)](http://www.ncbi.nlm.nih.gov/gene/89910) | ubiquitin protein ligase E3B | 109935487 .. 109935522 | 109,915,215..109,974,510 |  |  | element present inside the gene |
| 259 | CCAATAGCTGAACTTAATTCATCTGTAGTTCCCACG found at 1527871 line | MVK | [4598 (MVK)](http://www.ncbi.nlm.nih.gov/gene/4598) | mevalonate kinase | 110006575 .. 110006610 | 110,011,500..110,035,071 | 4925 |  |  |
| 260 | CCAATGGAGGGACCGTCTGTGCGAGAACCGGCCACG found at 1578988 line | TPCN1 | [53373 (TPCN1)](http://www.ncbi.nlm.nih.gov/gene/53373) | two pore segment channel 1 | 113687009 .. 113687044 | 113,659,260..113,736,390 |  |  | element present inside the gene |
| 261 | CCAATACTGATACATTATTATTAACTAAAGTCCACG found at 1655907 line | 0 |  |  |  |  |  |  |  |
| 262 | CCAATTTGCACACGTGGCATCTCTAAGGGTCCCACG found at 1659605 line | SRRM4 | [84530 (SRRM4)](http://www.ncbi.nlm.nih.gov/gene/84530) | serine/arginine repetitive matrix 4 | 119491472 .. 119491507 | 119,419,300..119,600,856 |  |  | element present inside the gene |
| 263 | CCAATAGGGAACTGACTAAATTATAGTAAATCCACG found at 1705033 line | 0 |  |  |  |  |  |  |  |
| 264 | CCAATTTAAGGTATCAGGCTGATTTTTAAAACCACG found at 1719213 line | 0 |  |  |  |  |  |  |  |
| 265 | CCAATTTAGAGAATCCATATGTACACATCCTCCACG found at 1853399 line | 0 |  |  |  |  |  |  |  |
|  | chrom 13---------18 match |  |  |  |  |  |  |  |  |
| 266 | CCAATCTCCACCTTCCAACACCCAGTGCCCTCCACG found at 269081 line | 0 |  |  |  |  |  |  |  |
| 267 | CCAATTCTTCAATTACACATAAAGCACTTAACCACG found at 354666 line | 0 |  |  |  |  |  |  |  |
| 268 | CCAATCGAAAGGGCCTTATTTTTAACTCACCCCACG found at 406843 line | 0 |  |  |  |  |  |  |  |
| 269 | CCAATTTTGGAAGAGTTAGCTGGCAGAGTCTCCACG found at 595463 line | AKAP11 | [11215 (AKAP11)](http://www.ncbi.nlm.nih.gov/gene/11215) | A kinase (PRKA) anchor protein 11 | 42873206 .. 42873241 | 42,846,261..42,897,403 |  |  | element present inside the gene |
| 270 | CCAATGCCCACTTGCCTTGTCAAGTACCTGGCCACG found at 598206 line | TNFSF11 | [8600 (TNFSF11)](http://www.ncbi.nlm.nih.gov/gene/8600) | tumor necrosis factor (ligand) superfamily, member 11 | 43070719 .. 43070754 | 43,136,872..43,182,149 |  |  | 66153 |
| 271 | CCAATAACAATCAAAATGTATATGTTAAATTCCACG found at 647455 line | CPB2-AS1 | [100509894 (CPB2-AS1)](http://www.ncbi.nlm.nih.gov/gene/100509894) | CPB2 antisense RNA 1 | 46616625 .. 46616660 | 46,626,983..46,675,482 | 10358 |  |  |
| 272 | CCAATGTACAACTCTCAATGCGGAGTTGCCGCCACG found at 726958 line | WDFY2 | [115825 (WDFY2)](http://www.ncbi.nlm.nih.gov/gene/115825) | WD repeat and FYVE domain containing 2 | 52340866 .. 52340901 | 52,158,484..52,340,935 |  |  | element present inside the gene |
| 273 | CCAATAGCTGGGGCTCCTCAGGCAACCCCCTCCACG found at 740779 line | 0 |  |  |  |  |  |  |  |
| 274 | CCAATAGGGACTCTGTGTGGGGGCTCTGACCCCACG found at 891036 line | 0 |  |  |  |  |  |  |  |
| 275 | CCAATGCCCTTCCTCTTGGCCTCCTTTTCAGCCACG found at 923440 line | 0 |  |  |  |  |  |  |  |
| 276 | CCAATATATAGTTTCTCAAAGTGAGTCCTCACCACG found at 933032 line | 0 |  |  |  |  |  |  |  |
| 277 | CCAATCTGTCCTGTAAGTCCTGAGTGCAGACCCACG found at 1044909 line | 0 |  |  |  |  |  |  |  |
| 278 | CCAATCAGAGGTACTTTCAATTTTTCATCTGCCACG found at 1077922 line | 0 |  |  |  |  |  |  |  |
| 279 | CCAATACCTGATAAAATAAAAAAGTTGCAGACCACG found at 1268715 line | 0 |  |  |  |  |  |  |  |
| 280 | CCAATTCTACGTAACAGCAACTCCACTGTGTCCACG found at 1333944 line | 0 |  |  |  |  |  |  |  |
| 281 | CCAATCAGAGATACGTACAATTTTCCATCTGCCACG found at 1385515 line | 0 |  |  |  |  |  |  |  |
| 282 | CCAATGGTTGTTGTTTTGTTTATGATGAATGCCACG found at 1412340 line | 0 |  |  |  |  |  |  |  |
| 283 | CCAATCCACAAAGAATCTTCAAGTGGGCACTCCACG found at 1572620 line | 0 |  |  |  |  |  |  |  |
|  | chrom 14------------13 match |  |  |  |  |  |  |  |  |
| 284 | CCAATAGCTTGAGTGGCAATGAAATACAGTGCCACG found at 517233 line | 0 |  |  |  |  |  |  |  |
| 285 | CCAATTTTGGAATTTGAGAACCATTTAGACTCCACG found at 663298 line | 0 |  |  |  |  |  |  |  |
| 286 | CCAATACTAACCATTAACTTTTGGCTCTGAGCCACG found at 751351 line | 0 |  |  |  |  |  |  |  |
| 287 | CCAATAAGCCTTAACTAAAGCTATGACTTAACCACG found at 763378 line | CGRRF1 | [10668 (CGRRF1)](http://www.ncbi.nlm.nih.gov/gene/10668) | cell growth regulator with ring finger domain 1 | 54963071 .. 54963106 | 54,976,587..55,005,334 |  | 13516 |  |
| 288 | CCAATATTTTTATTAGTTTATAATTACTAAGCCACG found at 877714 line | 0 |  |  |  |  |  |  |  |
| 289 | CCAATCAGCTGTCTTGAACACCCACTTTGATCCACG found at 957981 line | RAD51B | [5890 (RAD51B)](http://www.ncbi.nlm.nih.gov/gene/5890) | RAD51 paralog B | 68974479 .. 68974514 | 68,286,496..69,149,889 |  |  | element present inside the gene |
| 290 | CCAATCTGCCGTGTTGCATTTTCACTTTGAACCACG found at 1055037 line | BATF | [10538 (BATF)](http://www.ncbi.nlm.nih.gov/gene/10538) | basic leucine zipper transcription factor, ATF-like | 75962559 .. 75962594 | 75,988,784..76,013,335 |  |  | 26225 |
| 291 | CCAATGTGCTGAATTCATTGTCATTTAAACCCCACG found at 1103648 line | NRXN3 | [9369 (NRXN3)](http://www.ncbi.nlm.nih.gov/gene/9369) | neurexin 3 | 79462570 .. 79462605 | 78,636,716..80,334,633 |  |  | element present inside the gene |
| 292 | CCAATTCAATGGTGCTTTTTCCAAAGCCCACCCACG found at 1181186 line | 0 |  |  |  |  |  |  |  |
| 293 | CCAATTTCCCCAGAGGATATCATGAAAGCTTCCACG found at 1352381 line | 0 |  |  |  |  |  |  |  |
| 294 | CCAATCAACACAGATAGAGGACCAGCCAAGTCCACG found at 1411922 line | 0 |  |  |  |  |  |  |  |
| 295 | CCAATGAGGAGAAAAGCACAGTGGAGGAGTGCCACG found at 1417229 line | 0 |  |  |  |  |  |  |  |
| 296 | CCAATGCGATGTCAGTGTCCTGGTCCAATTCCCACG found at 1471922 line | TMEM121 | [80757 (TMEM121)](http://www.ncbi.nlm.nih.gov/gene/80757) | transmembrane protein 121 | 105978279 .. 105978314 | 105,992,953..105,996,554 |  | 14674 |  |
|  | chrom 15------------10 match |  |  |  |  |  |  |  |  |
| 297 | CCAATTCTCACCTGAGAGCCCTGCAGACGCCCCACG found at 289307 line | 0 |  |  |  |  |  |  |  |
| 298 | CCAATTCTCACCTGAGAGCCCTGCAGACGCCCCACG found at 326355 line | 0 |  |  |  |  |  |  |  |
| 299 | CCAATAAGCCTTCACTAAAGTTATGACTTAACCACG found at 330240 line | MKRN3 | [7681 (MKRN3)](http://www.ncbi.nlm.nih.gov/gene/7681) | makorin ring finger protein 3 | 23777193 .. 23777228 | 23,810,454..23,820,764 |  |  | 33261 |
| 300 | CCAATCGCCCATCTGTGGAAAAAAGTGTCTTCCACG found at 373639 line | 0 |  |  |  |  |  |  |  |
| 301 | CCAATGTAGAAGCTGCTCATCTGGCCCAGGTCCACG found at 398653 line | 0 |  |  |  |  |  |  |  |
| 302 | CCAATGCTATCCCTCCCCCATCCCCCCCACCCCACG found at 621470 line | CTDSPL2 | [51496 (CTDSPL2)](http://www.ncbi.nlm.nih.gov/gene/51496) | CTD (carboxy-terminal domain, RNA polymerase II, polypeptide A) small phosphatase like 2 | 44745753 .. 44745788 | 44,719,579..44,819,455 |  |  | element present inside the gene |
| 303 | CCAATCATGTTATGATATTATTGGTTTTTGTCCACG found at 703943 line | USP8 | [9101 (USP8)](http://www.ncbi.nlm.nih.gov/gene/9101) | ubiquitin specific peptidase 8 | 50683755 .. 50683790 | 50,716,579..50,793,280 |  |  | 32824 |
| 304 | CCAATCAACCACCTGCCTTGGGGCCAGGTGGCCACG found at 934709 line | 0 |  |  |  |  |  |  |  |
| 305 | CCAATTACAAGGTTTTTCCCTCCTAATCTAACCACG found at 1018456 line | NEO1 | [4756 (NEO1)](http://www.ncbi.nlm.nih.gov/gene/4756) | neogenin 1 | 73328710 .. 73328745 | 73,344,056..73,597,547 |  | 15346 |  |
| 306 | CCAATGTCATGTAGTAGAAAGCCCAGAAGTCCCACG found at 1096597 line | 0 |  |  |  |  |  |  |  |
|  | chrom 16----------14 match |  |  |  |  |  |  |  |  |
| 307 | CCAATGCAGCCCAAGATAGGGGGACAGGACCCCACG found at 18538 line | UBE2I | [7329 (UBE2I)](http://www.ncbi.nlm.nih.gov/gene/7329) | ubiquitin-conjugating enzyme E2I | 1334599 .. 1334634 | 1,357,420..1,377,019 |  |  | 22821 |
| 308 | CCAATGGCCACCCCTCACACCACAAGCCAAGCCACG found at 38775 line | SRRM2 | [23524 (SRRM2)](http://www.ncbi.nlm.nih.gov/gene/23524) | serine/arginine repetitive matrix 2 | 2791656 .. 2791691 | 2,802,330..2,821,413 | 10674 |  |  |
| 309 | CCAATATTTCTCAAGTTCCCTTTTCAGTATGCCACG found at 106672 line | RBFOX1 | [54715 (RBFOX1)](http://www.ncbi.nlm.nih.gov/gene/54715) | RNA binding protein, fox-1 homolog (C. elegans) 1 | 7680280 .. 7680315 | 5,289,469..7,763,342 |  |  | element present inside the gene |
| 310 | CCAATTATAGCTGATTATAATGGAAGATTGTCCACG found at 128701 line | 0 |  |  |  |  |  |  |  |
| 311 | CCAATTCATTCACTGAAGAGTCTGGACCACTCCACG found at 131296 line | 0 |  |  |  |  |  |  |  |
| 312 | CCAATCAGAGGTAACTTCAGTTTTTCATCTGCCACG found at 308703 line | EEF2K | [29904 (EEF2K)](http://www.ncbi.nlm.nih.gov/gene/29904) | eukaryotic elongation factor-2 kinase | 22226476 .. 22226511 | 22,217,592..22,300,066 |  |  | element present inside the gene |
| 313 | CCAATCCCTGCTTTAAAATGCAGAACTAGGACCACG found at 435908 line | ITGAX | [3687 (ITGAX)](http://www.ncbi.nlm.nih.gov/gene/3687) | integrin, alpha X (complement component 3 receptor 4 subunit) | 31385248 .. 31385283 | 31,366,488..31,394,320 |  |  | element present inside the gene |
| 314 | CCAATACGTAATCATTCCCCTTTCCTCTCTGCCACG found at 821713 line | 0 |  |  |  |  |  |  |  |
| 315 | CCAATGTATAACATCATGCATTCATTCATCTCCACG found at 872904 line | 0 |  |  |  |  |  |  |  |
| 316 | CCAATTAACCATTCCCATTTCTCCGTGCCTGCCACG found at 1020479 line | 0 |  |  |  |  |  |  |  |
| 317 | CCAATTAACTACATTGGGTGGGGAATTGAATCCACG found at 1031882 line | PSMD7 | [5713 (PSMD7)](http://www.ncbi.nlm.nih.gov/gene/5713) | proteasome (prosome, macropain) 26S subunit, non-ATPase, 7 | 74295406 .. 74295441 | 74,330,673..74,340,186 |  |  | 35267 |
| 318 | CCAATAGCTTGAGCAAAGAAGTCAGGTAAGCCCACG found at 1072187 line | MON1B | [22879 (MON1B)](http://www.ncbi.nlm.nih.gov/gene/22879) | MON1 homolog B (yeast) | 77197366 .. 77197401 | 77,224,821..77,233,543 |  |  | 27455 |
| 319 | CCAATCAGCCACATTCTAATCCCCTTCTCACCCACG found at 1100876 line | 0 |  |  |  |  |  |  |  |
| 320 | CCAATTTAAGATGCACAGGATTACCTGGTCTCCACG found at 1210500 line | 0 |  |  |  |  |  |  |  |
|  | chrom 17-----------12 match |  |  |  |  |  |  |  |  |
| 321 | CCAATTATGCATTCTGGCACTGGGGAAATGACCACG found at 46239 line | ASPA | [443 (ASPA)](http://www.ncbi.nlm.nih.gov/gene/443) | aspartoacylase | 3329085 .. 3329120 | 3,377,404..3,402,700 |  |  | 48319 |
| 322 | CCAATGCACAGCTGGCTGAGCAGGCCTGCCCCCACG found at 251971 line | LLGL1 | [3996 (LLGL1)](http://www.ncbi.nlm.nih.gov/gene/3996) | lethal giant larvae homolog 1 (Drosophila) | 18141780 .. 18141815 | 18,128,907..18,148,189 |  |  | element present inside the gene |
| 323 | CCAATTGCCTTCCACACAACAGCAAGGCCTCCCACG found at 362669 line | 0 |  |  |  |  |  |  |  |
| 324 | CCAATGGGGGGGTCCACACCTCCTGTCCATGCCACG found at 585527 line | 0 |  |  |  |  |  |  |  |
| 325 | CCAATCCTGATCTTATTGCTACCACCCATTACCACG found at 623245 line | WNT9B | [7484 (WNT9B)](http://www.ncbi.nlm.nih.gov/gene/7484) | wingless-type MMTV integration site family, member 9B | 44873484 .. 44873519 | 44,928,952..44,964,096 |  |  | 55468 |
| 326 | CCAATTTCTTTCATAAGCATAGATGCAGAGTCCACG found at 637196 line | LRRC46 | [90506 (LRRC46)](http://www.ncbi.nlm.nih.gov/gene/90506) | leucine rich repeat containing 46 | 45877970 .. 45878005 | 45,908,993..45,915,079 |  |  | 31023 |
| 327 | CCAATGATTTTATAAAGAGGAGTTCCCCCGGCCACG found at 687474 line | 0 |  |  |  |  |  |  |  |
| 328 | CCAATTCAGCCACTAATACCTTTGTATACTACCACG found at 736687 line | STXBP4 | [252983 (STXBP4)](http://www.ncbi.nlm.nih.gov/gene/252983) | syntaxin binding protein 4 | 53041317 .. 53041352 | 53,046,119..53,241,646 | 4802 |  |  |
| 329 | CCAATGGAGCTGAACGTCAGCGCCTCTGTCCCCACG found at 909542 line | PITPNC1 | [26207 (PITPNC1)](http://www.ncbi.nlm.nih.gov/gene/26207) | phosphatidylinositol transfer protein, cytoplasmic 1 | 65486898 .. 65486933 | 65,373,397..65,693,379 |  |  | element present inside the gene |
| 330 | CCAATTAGGAATGGGAGCCTTGCTCACAGCCCCACG found at 1029819 line | RNF157-AS1 | [100507218 (RNF157-AS1)](http://www.ncbi.nlm.nih.gov/gene/100507218) | RNF157 antisense RNA 1 | 74146870 .. 74146905 | 74,136,637..74,150,729 |  |  | element present inside the gene |
| 331 | CCAATACAAGACACAGCCGCCGCCCCGTTCTCCACG found at 1099793 line | 0 |  |  |  |  |  |  |  |
| 332 | CCAATCAGAAGGACTTCCAATTTTCCATCTGCCACG found at 1117633 line | FOXK2 | [3607 (FOXK2)](http://www.ncbi.nlm.nih.gov/gene/3607) | forkhead box K2 | 80469429 .. 80469464 | 80,477,594..80,562,483 | 8165 |  |  |
|  | chrom 18---------15 match |  |  |  |  |  |  |  |  |
| 333 | CCAATGTGATCCTCCTGTCTTCCTGGGCCCCCCACG found at 35662 line | NDC80 | [10403 (NDC80)](http://www.ncbi.nlm.nih.gov/gene/10403) | NDC80 kinetochore complex component | 2567531 .. 2567566 | 2,571,510..2,616,634 | 3979 |  |  |
| 334 | CCAATACTACCTATGGCTTGCCTGGGCATACCCACG found at 90728 line | 0 |  |  |  |  |  |  |  |
| 335 | CCAATTAAACAATTTTGGACTGGGGAAATTACCACG found at 92670 line | ARHGAP28 | [79822 (ARHGAP28)](http://www.ncbi.nlm.nih.gov/gene/79822) | Rho GTPase activating protein 28 | 6672133 .. 6672168 | 6,729,717..6,915,715 |  |  | 57584 |
| 336 | CCAATGCAAGGCTTCAGGAACAATTCCAGAGCCACG found at 122564 line | SOGA2 | [23255 (SOGA2)](http://www.ncbi.nlm.nih.gov/gene/23255) | SOGA family member 2 | 8824474 .. 8824509 | 8,705,659..8,832,776 |  |  | element present inside the gene |
| 337 | CCAATTTGTAGCCTGACTGGAATTGCAGATACCACG found at 335086 line | 0 |  |  |  |  |  |  |  |
| 338 | CCAATAATTTTTTTAAATTTTTAAAATTGTACCACG found at 397272 line | 0 |  |  |  |  |  |  |  |
| 339 | CCAATCTAAGGTATTATGGAAAGAGAGAGCACCACG found at 522114 line | 0 |  |  |  |  |  |  |  |
| 340 | CCAATGTCCACTGCTGGCACGCTGGGCCAAGCCACG found at 782547 line | MALT1 | [10892 (MALT1)](http://www.ncbi.nlm.nih.gov/gene/10892) | mucosa associated lymphoid tissue lymphoma translocation gene 1 | 56343290 .. 56343325 | 56,338,618..56,417,371 |  |  | element present inside the gene |
| 341 | CCAATGACATGCAATTTATCCATTAATAAACCCACG found at 790408 line | 0 |  |  |  |  |  |  |  |
| 342 | CCAATACTTCCAAATATTCAGATGTTTGATGCCACG found at 816965 line | 0 |  |  |  |  |  |  |  |
| 343 | CCAATGGGCACTTTGTGTGAGGGCTCCAACCCCACG found at 851279 line | 0 |  |  |  |  |  |  |  |
| 344 | CCAATCATCTTTGTATTAGTTATTTTGCATACCACG found at 899064 line | 0 |  |  |  |  |  |  |  |
| 345 | CCAATCGCCGCTGTCCAGTGCTTGGCCAGGGCCACG found at 940538 line | 0 |  |  |  |  |  |  |  |
| 346 | CCAATGGATACAATAAAGTTCAGCTTCTTAACCACG found at 957765 line | 0 |  |  |  |  |  |  |  |
| 347 | CCAATCACCACACACGCCCTTTCTGACTGAACCACG found at 1029264 line | 0 |  |  |  |  |  |  |  |
|  | chrom 19------------17 match |  |  |  |  |  |  |  |  |
| 348 | CCAATGTCGGCTTCCTAGGTCTGACGCAGCACCACG found at 22812 line | 0 |  |  |  |  |  |  |  |
| 349 | CCAATGTAGAAGCTGCTCATCTGGCCCAGGTCCACG found at 26113 line | SCAMP | [113178 (SCAMP4)](http://www.ncbi.nlm.nih.gov/gene/113178) | secretory carrier membrane protein 4 | 1880049 .. 1880084 | 1,905,213..1,926,012 |  |  | 25164 |
|  |  | ADAT3 | [113179 (ADAT3)](http://www.ncbi.nlm.nih.gov/gene/113179) | adenosine deaminase, tRNA-specific 3 | 1880049 .. 1880084 | 1,905,371..1,913,446 |  |  | 25322 |
| 350 | CCAATAGGAGGGGCGAATGACTCCACTGAGGCCACG found at 34391 line | GADD45B | [4616 (GADD45B)](http://www.ncbi.nlm.nih.gov/gene/4616) | growth arrest and DNA-damage-inducible, beta | 2476043 .. 2476078 | 2,476,123..2,478,257 | 80 |  |  |
| 351 | CCAATGGGAGTGCGCGCACCAGGGGATGCTGCCACG found at 161016 line | 0 |  |  |  |  |  |  |  |
| 352 | CCAATGTCCTACTGGTCATATAGTGAGCATCCCACG found at 180263 line | MAST1 | [22983 (MAST1)](http://www.ncbi.nlm.nih.gov/gene/22983) | microtubule associated serine/threonine kinase 1 | 12978828 .. 12978863 | 12,949,259..12,985,766 |  |  | element present inside the gene |
| 353 | CCAATGACCAGCCCAAAGGCCACAGAGAAGACCACG found at 209348 line | CCDC105 | [126402 (CCDC105)](http://www.ncbi.nlm.nih.gov/gene/126402) | coiled-coil domain containing 105 | 15072901 .. 15072936 | 15,121,539..15,134,083 |  |  | 48638 |
| 354 | CCAATCAATACCTGCTGCTGGCCCCAAGGAGCCACG found at 231097 line | 0 |  |  |  |  |  |  |  |
| 355 | CCAATGTGCATGGCTCCGATCCCAGCGGGTGCCACG found at 471400 line | 0 |  |  |  |  |  |  |  |
| 356 | CCAATTTGCCTAAGCAAGACCTTCCGGGCATCCACG found at 554370 line | PLEKHG2 | [64857 (PLEKHG2)](http://www.ncbi.nlm.nih.gov/gene/64857) | pleckstrin homology domain containing, family G (with RhoGef domain) member 2 | 39914532 .. 39914567 | 39,903,222..39,919,055 |  |  | element present inside the gene |
| 357 | CCAATCAGAGGCACTTTCCATCTTTCATCGGCCACG found at 560411 line | SUPT5H | [6829 (SUPT5H)](http://www.ncbi.nlm.nih.gov/gene/6829) | suppressor of Ty 5 homolog (S. cerevisiae) | 39914532 .. 39914567 | 39,936,186..39,967,310 |  |  | 21654 |
| 358 | CCAATCCCCTGAGCCCTGTGCAAATCAGACACCACG found at 598097 line | LIPE-AS1 (Partial stop) | [100996307 (LIPE-AS1)](http://www.ncbi.nlm.nih.gov/gene/100996307) | LIPE antisense RNA 1 | 43062866 .. 43062901 | 42,901,300..43,156,507 |  |  | element present inside the gene |
| 359 | CCAATATTGATACATTATTATTAACTAAAGTCCACG found at 617299 line | ZNF221 | [7638 (ZNF221)](http://www.ncbi.nlm.nih.gov/gene/7638) | zinc finger protein 221 | 44445376 .. 44445411 | 44,455,375..44,471,752 | 9999 |  |  |
| 360 | CCAATGATGCTAAATTCCCTTATTTTATGTCCCACG found at 640090 line | 0 |  |  |  |  |  |  |  |
| 361 | CCAATGACACTGCTGCCAGCCCCTGGGGGGACCACG found at 681023 line | SULT2B1 | [6820 (SULT2B1)](http://www.ncbi.nlm.nih.gov/gene/6820) | sulfotransferase family, cytosolic, 2B, member 1 | 49033506 .. 49033541 | 49,055,429..49,102,684 |  |  | 21923 |
| 362 | CCAATGTTATTACAAGACCTCACACCAGCAGCCACG found at 681863 line | SULT2B1 | [6820 (SULT2B1)](http://www.ncbi.nlm.nih.gov/gene/6820) | sulfotransferase family, cytosolic, 2B, member 1 | 49093996 .. 49094031 | 49,055,429..49,102,684 |  |  | element present inside the gene |
| 363 | CCAATCTATGTTGTGAATGCCCAGTTGAGACCCACG found at 760708 line | 0 |  |  |  |  |  |  |  |
| 364 | CCAATGTATCCCTCCCAGCCTTCTCCTCACCCCACG found at 772521 line | 0 |  |  |  |  |  |  |  |
|  | chrom 20----------10 match |  |  |  |  |  |  |  |  |
| 365 | CCAATTAAAGATGATTTTTACAGTCAATGAGCCACG found at 64818 line | PRNP | [5621 (PRNP)](http://www.ncbi.nlm.nih.gov/gene/5621) | prion protein | 4666794 .. 4666829 | 4,666,797..4,682,235 |  |  | element present inside the gene |
| 366 | CCAATCGGCGGTGCCCGCGCAGGGTGCTACGCCACG found at 324207 line | GZF1 | [64412 (GZF1)](http://www.ncbi.nlm.nih.gov/gene/64412) | GDNF-inducible zinc finger protein 1 | 23342767 .. 23342802 | 23,342,769..23,353,683 |  |  | element present inside the gene |
| 367 | CCAATCCCTGTGACTATCTACAGTATCCACCCCACG found at 455189 line | 0 |  |  |  |  |  |  |  |
| 368 | CCAATCTCTTCTCGTGATAATGAGGGAGTTCCCACG found at 506238 line | CTNNBL1 | [56259 (CTNNBL1)](http://www.ncbi.nlm.nih.gov/gene/56259) | catenin, beta like 1 | 36448995 .. 36449030 | 36,322,357..36,500,531 |  |  | element present inside the gene |
| 369 | CCAATTCCTTTCTCACCCACCCCACTCTCAGCCACG found at 542906 line | 0 |  |  |  |  |  |  |  |
| 370 | CCAATGCCCCAAATACTGCCCCAGCCACCAACCACG found at 658183 line | 0 |  |  |  |  |  |  |  |
| 371 | CCAATAGATCCATGTGCAAACAGATCAGGGGCCACG found at 807643 line | PHACTR3 | [116154 (PHACTR3)](http://www.ncbi.nlm.nih.gov/gene/116154) | phosphatase and actin regulator 3 | 58150208 .. 58150243 | 58,152,564..58,422,766 | 2356 |  |  |
| 372 | CCAATAGGACAAGTTGGCATTGCCCAGACCACCACG found at 829688 line | 0 |  |  |  |  |  |  |  |
| 373 | CCAATCTGAGCTCTTGTCTTTTCAAACCCCACCACG found at 839697 line | CDH4 | [1002 (CDH4)](http://www.ncbi.nlm.nih.gov/gene/1002) | cadherin 4, type 1, R-cadherin (retinal) | 60458065 .. 60458100 | 59,827,482..60,515,673 |  |  | element present inside the gene |
| 374 | CCAATCTCACTTTTGCCATCCTCCAGTGTGACCACG found at 854056 line | 0 |  |  |  |  |  |  |  |
|  | chrom 21……………..6 match |  |  |  |  |  |  |  |  |
| 375 | CCAATCCCCACCTTCCAATACCCAGTGCCCTCCACG found at 211682 line | 0 |  |  |  |  |  |  |  |
| 376 | CCAATCCCCACCTTCCAATACCCAGTGCCCTCCACG found at 211954 line | 0 |  |  |  |  |  |  |  |
| 377 | CCAATCCATTGGTCATTCTGTCATTTTTTCACCACG found at 250879 line | 0 |  |  |  |  |  |  |  |
| 378 | CCAATTCCGGACACAGTATGATAACCCCTAGCCACG found at 350416 line | 0 |  |  |  |  |  |  |  |
| 379 | CCAATTATACTCCTTTCCCTCCTTGGCCTTCCCACG found at 601869 line | 0 |  |  |  |  |  |  |  |
| 380 | CCAATTCCCATCCCCCAGCAGCGTGTTAGCGCCACG found at 656350 line | PCBP3 | [54039 (PCBP3)](http://www.ncbi.nlm.nih.gov/gene/54039) | poly(rC) binding protein 3 | 47257049 .. 47257084 | 47,268,023..47,362,368 | 10974 |  |  |
|  | chrom 22-----------9 match |  |  |  |  |  |  |  |  |
| 381 | CCAATCTCCAAGCACTGCTTGGCATCCGCGGCCACG found at 260815 line | 0 |  |  |  |  |  |  |  |
| 382 | CCAATGTAGAAGCTGCTCATCTGGCTCAGGTCCACG found at 312113 line | IGL | [3535 (IGL)](http://www.ncbi.nlm.nih.gov/gene/3535) | immunoglobulin lambda locus | 22472004 .. 22472039 | 22,380,474..23,265,085 |  |  | element present inside the gene |
| 383 | CCAATACCCATCTTTGACTCATCACAGTCTTCCACG found at 389934 line | 0 |  |  |  |  |  |  |  |
| 384 | CCAATGTCCCCGGGACCAGCTCTTTCTCTGGCCACG found at 390219 line | 0 |  |  |  |  |  |  |  |
| 385 | CCAATATCTTTAGCTGGGAATGGAAATATCCCCACG found at 437878 line | INPP5J | [27124 (INPP5J)](http://www.ncbi.nlm.nih.gov/gene/27124) | inositol polyphosphate-5-phosphatase J | 31527086 .. 31527121 | 31,518,893..31,530,683 |  |  | element present inside the gene |
| 386 | CCAATTGGAAGGGGCTTTGCCATCTGTCTCACCACG found at 643920 line | 0 |  |  |  |  |  |  |  |
| 387 | CCAATCAGAGGTACTTTCAGTTTTTCATCTGCCACG found at 656855 line | TBC1D22A | [25771 (TBC1D22A)](http://www.ncbi.nlm.nih.gov/gene/25771) | TBC1 domain family, member 22A | 47293410 .. 47293445 | 47,158,514..47,571,342 |  |  | element present inside the gene |
| 388 | CCAATTCTCCTGGCGTTTAGAAGACAAATCACCACG found at 657876 line | TBC1D22A | [25771 (TBC1D22A)](http://www.ncbi.nlm.nih.gov/gene/25771) |  | 47366965 .. 47367000 | 47,158,514..47,571,342 |  |  | element present inside the gene |
| 389 | CCAATGACAGATACACCGTCTGCTCAGGTCACCACG found at 692807 line | 0 |  |  |  |  |  |  |  |
|  | chrom X-------------17 match |  |  |  |  |  |  |  |  |
| 390 | CCAATGGCTTTGAAATTTTTCTATAAGGAAGCCACG found at 151130 line | 0 |  |  |  |  |  |  |  |
| 391 | CCAATCTGGTGGCTCTTTTAGCCGACTTTAGCCACG found at 176747 line | FRMPD4 | [9758 (FRMPD4)](http://www.ncbi.nlm.nih.gov/gene/9758) | FERM and PDZ domain containing 4 | 12725664 .. 12725699 | 12,156,585..12,742,642 |  |  | element present inside the gene |
| 392 | CCAATGTGTGTGAAAGGTGTGCTGGCCATGGCCACG found at 252747 line | 0 |  |  |  |  |  |  |  |
| 393 | CCAATAGGCTGCACAATCTGAAATGTCATGCCCACG found at 264174 line | 0 |  |  |  |  |  |  |  |
| 394 | CCAATTATGCATTGTGGCACTAGGAAAATGACCACG found at 888351 line | 0 |  |  |  |  |  |  |  |
| 395 | CCAATTAAAAAAAAAAAACAAATCATAGCAACCACG found at 896351 line | 0 |  |  |  |  |  |  |  |
| 396 | CCAATAAGCCTTTACTAAGGTTATAACTTAACCACG found at 896603 line | 0 |  |  |  |  |  |  |  |
| 397 | CCAATCCATTTTCAGGCCTGACTTTTGTGAGCCACG found at 952534 line | 0 |  |  |  |  |  |  |  |
| 398 | CCAATTATGCATTCTGGAACTTGGACAATAACCACG found at 1022049 line | SLC16A2 | [6567 (SLC16A2)](http://www.ncbi.nlm.nih.gov/gene/6567) | solute carrier family 16, member 2 (thyroid hormone transporter) | 73587406 .. 73587441 | 73,641,328..73,753,764 |  |  | 53922 |
| 399 | CCAATTACATAAACAGAATCAATGACAAAAACCACG found at 1216213 line | 0 |  |  |  |  |  |  |  |
| 400 | CCAATGACTGTAAAATGAAGGGATACATAGGCCACG found at 1254606 line | 0 |  |  |  |  |  |  |  |
| 401 | CCAATTATGCATTCTGGCACTGGGGAAATGACCACG found at 1491516 line | ATG4A | [115201 (ATG4A)](http://www.ncbi.nlm.nih.gov/gene/115201) | autophagy related 4A, cysteine peptidase | 107389047 .. 107389082 | 107,334,899..107,397,901 |  |  | element present inside the gene |
| 402 | CCAATAGTTGGTTGTTTGAAAGGATAACAAACCACG found at 1519845 line | 0 |  |  |  |  |  |  |  |
| 403 | CCAATCGACTGAACCCAACTTATAATCAAACCCACG found at 1613966 line | 0 |  |  |  |  |  |  |  |
| 404 | CCAATCATAGGTACTTTCAATTTCCCATCGGCCACG found at 1789596 line | XPNPEP2 | [7512 (XPNPEP2)](http://www.ncbi.nlm.nih.gov/gene/7512) | X-prolyl aminopeptidase (aminopeptidase P) 2, membrane-bound | 128850783 .. 128850818 | 128,872,946..128,903,525 |  |  | 22163 |
| 405 | CCAATTAATTAAAAATATCTGAGGGTGGGACCCACG found at 2033418 line | 0 |  |  |  |  |  |  |  |
| 406 | CCAATCCCTAGGACAAATATGGTTTTGGGCTCCACG found at 2116358 line | 0 |  |  |  |  |  |  |  |
|  | chrom Y-----------5 match |  |  |  |  |  |  |  |  |
| 407 | CCAATGACTTACCAATTACTCACTGATTTGACCACG found at 112113 line | 0 |  |  |  |  |  |  |  |
| 408 | CCAATATTCCCTCTTTGATGGGGAAAAATGGCCACG found at 353943 line | 0 |  |  |  |  |  |  |  |
| 409 | CCAATATTCCCTCTTTGATGGGGAAAAATGGCCACG found at 376382 line | 0 |  |  |  |  |  |  |  |
| 410 | CCAATGACTTACTGATTAGTCACTGACTTCACCACG found at 378416 line | 0 |  |  |  |  |  |  |  |
| 411 | CCAATTTAAATCCAATAGCTTTTCCACACCTCCACG found at 399467 line | 0 |  |  |  |  |  |  |  |
