# Supplementary material for: Identification of ASB7 as ER stress responsive gene through a genome wide in silico screening for genes with ERSE
Source: PLoS One. 2018 Apr 9;13(4):e0194310. doi: 10.1371/journal.pone.0194310 (PMC5890977; doi:10.1371/journal.pone.0194310)
Supplement: S3 Table — (DOC) [file pone.0194310.s003.doc]

**S3 Table:**

The extracted corresponding closest gene present within 10kb region and also extracted the gene which is having ERSE element inside the gene.

| **SNO** | **ERSE element** | **Gene** | **gene ID** | **Gene name** | **Hit position** | **Gene position** | **10 Kb** | **Remark** |
| --- | --- | --- | --- | --- | --- | --- | --- | --- |
|  |  |  |  | **ERSE-I** |  |  |  |  |
| 1 | CCAATCCCCGGGCACCACG found at 16172 line | B3GALT6 | [126792 (B3GALT6)](http://www.ncbi.nlm.nih.gov/gene/126792) | UDP-Gal:betaGal beta 1,3-galactosyltransferase polypeptide 6 | 1,164,255.. 1,164,273 | 1,167,629..1,170,421 | 3374 |  |
| 2 | CCAATTGTAATCCCCCACG found at 396193 line | TPRG1L | [127262 (TPRG1L)](http://www.ncbi.nlm.nih.gov/gene/127262) | tumor protein p63 regulated 1-like | 3536037 .. 3536055 | 3,541,556..3,546,695 | 5519 |  |
| 3 | CCAATATGGTGAAACCACG found at 56437 line | DARS2 | ID: 55157 | aspartyl-tRNA synthetase 2, mitochondrial | 173812508 .. 173812526 | 173,793,719..173,827,682 |  | element present inside gene |
| 4 | CCAATGAAATAAAGCCACG found at 2252455 line | NOS1AP | ID: 9722 | nitric oxide synthase 1 (neuronal) adaptor protein | 162176667 .. 162176685 | 162,039,581..162,339,813 |  | element present inside the gene |
| 5 | CCAATTCTTTCTGGCCACG found at 2392948 line | DNM3 | ID: 26052 | dynamin 3 | 172292140 .. 172292158 | 171,810,618..172,387,606 |  | element present inside the gene |
| 6 | CCAATTAGAGACATCCACG found at 2494728 line | TDRD5 | ID: 163589 | tudor domain containing 5 | 179620304 .. 179620322 | 179,560,748..179,660,407 |  | element present inside the gene |
| 7 | CCAATGCACACCACCCACG found at 2793800 line | IGFN1 | [91156 (IGFN1)](http://www.ncbi.nlm.nih.gov/gene/91156) | immunoglobulin-like and fibronectin type III domain containing 1 | 201153485 .. 201153503 | 201,159,952..201,198,080 | 6467 |  |
| 8 | CCAATCACCTCCCACCACG found at 3098804 line | DISP1 | ID: 84976 | dispatched homolog 1 (Drosophila) | 223113760 .. 223113778 | 222,988,342..223,179,337 |  | element present inside the gene |
| 9 | CCAATGCACACTGGCCACG found at 3255058 line | SLC35F3 | ID: 148641 | solute carrier family 35, member F3 | 234364078 .. 234364096 | 234,040,679..234,460,262 |  | element present inside the gene |
| 10 | CCAATTTGGACAAGCCACG found at 165492 line | LPIN1 | ID: 23175 | lipin 1 | 11915273 .. 11915291 | 11,817,705..11,967,535 |  | element present inside the gene |
| 11 | CCAATGTGGTGAAACCACG found at 344343 line | NCOA1 | ID: 8648 | nuclear receptor coactivator 1 | 24792550 .. 24792568 | 24,714,919..24,993,571 |  | element present inside the gene |
| 12 | CCAATAAATGCTATCCACG found at 374363 line | KCNK3 | ID: 3777 | potassium channel, subfamily K, member 3 | 26954039 .. 26954057 | 26,915,581..26,954,066 |  | element present inside the gene |
| 13 | CCAATCAGGGTTGCCCACG found at 1434480 line | SLC9A2 | ID: 6549 | solute carrier family 9, subfamily A (NHE2, cation proton antiporter 2), member 2 | 103282439 .. 103282457 | 103,236,166..103,327,809 |  | element present inside the gene |
| 14 | CCAATCACACTGGGCCACG found at 1666378 line | STEAP3 | [55240 (STEAP3)](http://www.ncbi.nlm.nih.gov/gene/55240) | STEAP family member 3, metalloreductase | 119979088 .. 119979106 | 119,981,384..120,023,228 | 2296 |  |
| 15 | CCAATTCTCTCCATCCACG found at 1782842 line | MYO7B | ID: 4648 | myosin VIIB | 128364497 .. 128364515 | 128,293,378..128,395,303 |  | element present inside the gene |
| 16 | CCAATCATCACCCACCACG found at 1818698 line | MZT2B | ID: 80097 | mitotic spindle organizing protein 2B | 130946100 .. 130946118 | 130,939,248..130,962,365 |  | element present inside the gene |
| 17 | CCAATGCACTTTAGCCACG found at 3367898 line | BOK | [666 (BOK)](http://www.ncbi.nlm.nih.gov/gene/666) | BCL2-related ovarian killer | 242488511 .. 242488529 | 242,498,146..242,513,553 | 9635 |  |
| 18 | CCAATTTCTGAATCCCACG found at 24609 line | RPL23AP39 | [100271461 (RPL23AP39)](http://www.ncbi.nlm.nih.gov/gene/100271461) | ribosomal protein L23a pseudogene 39 | 1771696 .. 1771714 | 1,771,738..1,772,158 | 42 |  |
| 19 | CCAATTTCTTTCTTCCACG found at 36316 line | CNTN4 | ID: 152330 | contactin 4 | 2614636 .. 2614654 | 2,140,550..3,099,645 |  | element present inside the gene |
| 20 | CCAATGGTTTGACACCACG found at 107178 line | GRM7 | ID: 2917 | glutamate receptor, metabotropic 7 | 7716693 .. 7716711 | 6,902,802..7,783,218 |  | element present inside the gene |
| 21 | CCAATCCCTTATTTCCACG found at 563940 line | ENPP7P4 (pseudo) | ID: 2917 | ectonucleotide pyrophosphatase/phosphodiesterase 7 pseudogene 4 | 125583594 .. 125583612 | 125,567,066..125,628,257 |  | element present inside the gene |
| 22 | CCAATAAGAGTAAGCCACG found at 1051577 line | LOC100128827 (pseudo) | [100128827 (LOC100128827)](http://www.ncbi.nlm.nih.gov/gene/100128827) | FSHD region gene 2 pseudogene | 197836918 .. 197836936 | 197,837,077..197,838,741 | 159 |  |
| 23 | CCAATAAGAGTAAGCCACG found at 2747737 line | FRG2C | [100288801 (FRG2C)](http://www.ncbi.nlm.nih.gov/gene/100288801) | FSHD region gene 2 family, member C | 75713416 .. 75713434 | 75,713,487..75,716,368 | 71 |  |
| 24 | CCAATTCGCTGTCTCCACG found at 2087009 line | EIF2A | ID: 83939 | eukaryotic translation initiation factor 2A, 65kDa | 150264497 .. 150264515 | 150,264,465..150,303,803 |  | element present inside the gene |
| 25 | CCAATGAAGAATACCCACG found at 2093786 line | CLRN1-AS1 (pseudo) | ID: 116933 | CLRN1 antisense RNA 1 | 150752487 .. 150752505 | 150,690,465..150,797,617 |  | element present inside pseudo gene |
| 26 | CCAATCACTCCTCACCACG found at 2477008 line | KCNMB2 | ID: 10242 | potassium large conductance calcium-activated channel, subfamily M, beta member 2 | 178344484 .. 178344502 | 178,254,086..178,562,217 |  | element present inside the gene |
| 27 | CCAATCTCAACATGCCACG found at 104858 line | SORCS2 | ID: 57537 | sortilin-related VPS10 domain containing receptor 2 | 7549649 .. 7549667 | 7,194,374..7,744,564 |  | element present inside the gene |
| 28 | CCAATGACCAGGCCCCACG found at 735027 line | SPATA18 | ID: 132671 | spermatogenesis associated 18 | 52921840 .. 52921858 | 52,917,578..52,963,461 |  | element present inside the gene |
| 29 | CCAATTTATTGAAGCCACG found at 865498 line | LPHN3 | ID: 23284 | latrophilin 3 | 62315747 .. 62315765 | 62,066,949..62,938,168 |  | element present inside the gene |
| 30 | CCAATTACCTCCCACCACG found at 997210 line | MOB1B | ID: 92597 | MOB kinase activator 1B | 71798973 .. 71798991 | 71,768,043..71,853,891 |  | element present inside the gene |
| 31 | CCAATGCTGAGTTGCCACG found at 2142291 line | TRIM2 | ID: 23321 | tripartite motif containing 2 | 154244836 .. 154244854 | 154,073,650..154,260,474 |  | element present inside the gene |
| 32 | CCAATTTCTACTTCCCACG found at 842995 line | ZSWIM6 | ID: 57688 | zinc finger, SWIM-type containing 6 | 60695552 .. 60695570 | 60,628,100..60,841,999 |  | element present inside the gene |
| 33 | CCAATTGGTCATGTCCACG found at 1118452 line | CKMT2 | [1160 (CKMT2)](http://www.ncbi.nlm.nih.gov/gene/1160) | creatine kinase, mitochondrial 2 (sarcomeric) | 80528392 .. 80528410 | 80,528,605..80,562,217 | 213 |  |
| 34 | CCAATGCCCCTTCCCCACG found at 1920323 line | CTNNA1 | ID: 1495 | catenin (cadherin-associated protein), alpha 1, 102kDa | 138263124 .. 138263142 | 138,089,075..138,270,723 |  | element present inside the gene |
| 35 | CCAATGAATGTTTTCCACG found at 1954587 line | PCDHGA1 | ID: 56114 | protocadherin gamma subfamily A, 1 | 140730155 .. 140730173 | 140,709,388..140,892,546 |  | element present inside the gene |
| 36 |  | PCDHG@ | ID: 56115 | protocadherin gamma cluster | 140730155 .. 140730173 | 140,710,252..140,892,546 |  | element present inside the gene |
| 37 |  | PCDHGA2 | ID: 56113 | protocadherin gamma subfamily A, 2 | 140730155 .. 140730173 | 140,718,354..140,892,546 |  | element present inside the gene |
| 38 |  | PCDHGB1 | ID: 56104 | protocadherin gamma subfamily B, 1 | 140730155 .. 140730173 | 140,729,828..140,892,546 |  | element present inside the gene |
| 39 |  | PCDHGA3 | ID: 56112 | protocadherin gamma subfamily A, 3 | 140730155 .. 140730173 | 140,723,601..140,892,546 |  | element present inside the gene |
| 40 |  | PCDHGA4 | [56111 (PCDHGA4)](http://www.ncbi.nlm.nih.gov/gene/56111) | protocadherin gamma subfamily A, 4 | 140730155 .. 140730173 | 140,734,768..140,892,546 | 4613 |  |
| 41 |  | PCDHGB2 | [56103 (PCDHGB2)](http://www.ncbi.nlm.nih.gov/gene/56103) | protocadherin gamma subfamily B, 2 | 140730155 .. 140730173 | 140,739,409..140,892,546 | 9254 |  |
| 42 | CCAATCTTCACACTCCACG found at 2487860 line | CANX | ID: 821 | calnexin | 179125779 .. 179125797 | 179,125,019..179,158,642 |  | element present inside the gene |
| 43 | CCAATCACCTCCCTCCACG found at 2506444 line | BTNL9 | [153579 (BTNL9)](http://www.ncbi.nlm.nih.gov/gene/153579) | butyrophilin-like 9 | 180463852 .. 180463870 | 180,467,203..180,488,523 | 3351 |  |
| 44 | CCAATCCCTAGGTACCACG found at 70620 line | PPP1R3G | 648791 | protein phosphatase 1, regulatory subunit 3G | 5084500 .. 5084518 | 5,085,720..5,087,455 | 1220 |  |
| 45 | CCAATTTTCTATACCCACG found at 392311 line | PGBD1 | [84547 (PGBD1)](http://www.ncbi.nlm.nih.gov/gene/84547) | piggyBac transposable element derived 1 | 28246297 .. 28246315 | 28,249,314..28,270,326 | 3017 |  |
| 46 | CCAATTATGTGCCACCACG found at 538766 line | DNAH8 | [1769 (DNAH8)](http://www.ncbi.nlm.nih.gov/gene/1769) | dynein, axonemal, heavy chain 8 | 38791045 .. 38791063 | 38,683,117..38,998,574 |  | element present inside the gene |
| 47 | CCAATACTGAATGACCACG found at 661393 line | GPR111 | [222611 (GPR111)](http://www.ncbi.nlm.nih.gov/gene/222611) | G protein-coupled receptor 111 | 47620176 .. 47620194 | 47,624,223..47,665,533 | 4047 |  |
| 48 | CCAATATGGTGAGACCACG found at 987806 line | FAM135A | [57579 (FAM135A)](http://www.ncbi.nlm.nih.gov/gene/57579) | family with sequence similarity 135, member A | 71121943 .. 71121961 | 71,123,107..71,270,877 | 1164 |  |
| 49 | CCAATAAAAATTCACCACG found at 2115340 line | ESR1 | [2099 (ESR1)](http://www.ncbi.nlm.nih.gov/gene/2099) | estrogen receptor 1 | 152304370 .. 152304388 | 152,011,631..152,424,409 |  | element present inside the gene |
| 50 | CCAATCTGGCAGGACCACG found at 59193 line | SDK1 | [221935 (SDK1)](http://www.ncbi.nlm.nih.gov/gene/221935) | sidekick cell adhesion molecule 1 | 4261751 .. 4261769 | 3,341,080..4,308,632 |  | elements present inside the gene |
| 51 | CCAATCACCTCCCACCACG found at 1589802 line | RPS14P10 (pseudo) | [647034 (RPS14P10)](http://www.ncbi.nlm.nih.gov/gene/647034) | ribosomal protein S14 pseudogene 10 | 131350056 .. 131350074 | 131,350,338..131,350,544 | 282 |  |
| 52 | CCAATGTAAAATTACCACG found at 1253303 line | CDK14 | [5218 (CDK14)](http://www.ncbi.nlm.nih.gov/gene/5218) | cyclin-dependent kinase 14 | 90237659 .. 90237677 | 90,225,681..90,839,905 |  | element present inside the gene |
| 53 | CCAATTTTTTGTGGCCACG found at 1587188 line | FOXP2 | [93986 (FOXP2)](http://www.ncbi.nlm.nih.gov/gene/93986) | forkhead box P2 | 114277386 .. 114277404 | 113,726,365..114,333,827 |  | element present inside the gene |
| 54 | CCAATCACCTCCAACCACG found at 1617011 line | MET | [4233 (MET)](http://www.ncbi.nlm.nih.gov/gene/4233) | met proto-oncogene | 116424694 .. 116424712 | 116,312,406..116,438,440 |  | element present inside the gene |
| 55 | CCAATGGGTAATACCCACG found at 1671072 line | KCND2 | [3751 (KCND2)](http://www.ncbi.nlm.nih.gov/gene/3751) | potassium voltage-gated channel, Shal-related subfamily, member 2 | 120317069 .. 120317087 | 119,913,689..120,390,387 |  | element present inside the gene |
| 56 | CCAATATATAAGGACCACG found at 2045782 line | CNTNAP2 | [26047 (CNTNAP2)](http://www.ncbi.nlm.nih.gov/gene/26047) | contactin associated protein-like 2 | 147296163 .. 147296181 | 145,813,453..148,118,090 |  | element present inside the gene |
| 57 | CCAATGTCCTGGAGCCACG found at 2096321 line | CHPF2 | [54480 (CHPF2)](http://www.ncbi.nlm.nih.gov/gene/54480) | chondroitin polymerizing factor 2 | 150934983 .. 150935001 | 150,929,575..150,935,913 |  | element present inside the gene |
| 58 | CCAATCCCTTATTTCCACG found at 970644 line | HSPD1P3 Pseudo | [3332 (HSPD1P3)](http://www.ncbi.nlm.nih.gov/gene/3332) | heat shock 60kDa protein 1 (chaperonin) pseudogene 3 | 7260862 .. 7260880 | 7,248,860..7,278,554 |  | element present inside the gene |
| 59 | CCAATACCCTCTTACCACG found at 944187 line | CSPP1 | [79848 (CSPP1)](http://www.ncbi.nlm.nih.gov/gene/79848) | centrosome and spindle pole associated protein 1 | 67981320 .. 67981338 | 67,976,588..68,108,849 |  | element present inside the gene |
| 60 | CCAATTCATTCAGTCCACG found at 1641471 line | SLC30A8 | [169026 (SLC30A8)](http://www.ncbi.nlm.nih.gov/gene/169026) | solute carrier family 30 (zinc transporter), member 8 | 118185755 .. 118185773 | 117,962,512..118,188,953 |  | element present inside the gene |
| 61 | CCAATCTCCGGCGCCCACG found at 2024277 line | LRRC14 | [9684 (LRRC14)](http://www.ncbi.nlm.nih.gov/gene/9684) | leucine rich repeat containing 14 | 145747820 .. 145747838 | 145,743,349..145,750,562 |  | element present inside the gene |
| 62 | CCAATTTAATAACCCCACG found at 990769 line | PIP5K1B | [8395 (PIP5K1B)](http://www.ncbi.nlm.nih.gov/gene/8395) | phosphatidylinositol-4-phosphate 5-kinase, type I, beta | 71335255 .. 71335273 | 71,320,188..71,624,092 |  | element present inside the gene |
| 63 | CCAATGGGTCCCTCCCACG found at 1092606 line | PCSK5 | [5125 (PCSK5)](http://www.ncbi.nlm.nih.gov/gene/5125) | proprotein convertase subtilisin/kexin type 5 | 78667477 .. 78667495 | 78,505,560..78,977,255 |  | element present inside the gene |
| 64 | CCAATACCCCTACTCCACG found at 1143378 line | TLE4 | [7091 (TLE4)](http://www.ncbi.nlm.nih.gov/gene/7091) | transducin-like enhancer of split 4 (E(sp1) homolog, Drosophila) | 82323064 .. 82323082 | 82,186,878..82,341,658 |  | element present inside the gene |
| 65 | CCAATGGCACCATGCCACG found at 1214195 line | NTRK2 | [4915 (NTRK2)](http://www.ncbi.nlm.nih.gov/gene/4915) | neurotrophic tyrosine kinase, receptor, type 2 | 87421895 .. 87421913 | 87,283,417..87,641,985 |  | element present inside the gene |
| 66 | CCAATCTAGTCCTTCCACG found at 1331026 line | SUSD3 | [203328 (SUSD3)](http://www.ncbi.nlm.nih.gov/gene/203328) | sushi domain containing 3 | 95833750 .. 95833768 | 95,820,989..95,847,420 |  | element present inside the gene |
| 67 | CCAATGATCACTTACCACG found at 458558 line | C10orf68 | [79741 (C10orf68)](http://www.ncbi.nlm.nih.gov/gene/79741) | chromosome 10 open reading frame 68 | 33016074 .. 33016092 | 32,735,057..33,171,805 |  | element present inside the gene |
| 68 | CCAATTTTGCCTGCCCACG found at 693144 line | WDFY4 | [57705 (WDFY4)](http://www.ncbi.nlm.nih.gov/gene/57705) | WDFY family member 4 | 49906278 .. 49906296 | 49,892,907..50,191,001 |  | element present inside the gene |
| 69 | CCAATTCCCAAACTCCACG found at 981388 line | DDX50 | [79009 (DDX50)](http://www.ncbi.nlm.nih.gov/gene/79009) | DEAD (Asp-Glu-Ala-Asp) box polypeptide 50 | 70659812 .. 70659830 | 70,661,034..70,706,603 | 1222 |  |
| 70 | CCAATTCTACTTAACCACG found at 1082994 line | C10orf11 | [83938 (C10orf11)](http://www.ncbi.nlm.nih.gov/gene/83938) | chromosome 10 open reading frame 11 | 77975435 .. 77975453 | 77,191,217..78,317,133 |  | element present inside the gene |
| 71 | CCAATTTATACTACCCACG found at 1473624 line | CCDC147 | [159686 (CCDC147)](http://www.ncbi.nlm.nih.gov/gene/159686) | coiled-coil domain containing 147 | 106100793 .. 106100811 | 106,099,377..106,214,848 |  | element present inside the gene |
| 72 | CCAATCCCTAAACCCCACG found at 1867829 line | INPP5A | [3632 (INPP5A)](http://www.ncbi.nlm.nih.gov/gene/3632) | inositol polyphosphate-5-phosphatase, 40kDa | 134483588 .. 134483606 | 134,351,283..134,596,984 |  | element present inside the gene |
| 73 | CCAATCCCTTATTTCCACG found at 50977 line | ART1 | [417 (ART1)](http://www.ncbi.nlm.nih.gov/gene/417) | ADP-ribosyltransferase 1 | 3670190 .. 3670208 | 3,663,487..3,685,844 |  | element present inside the gene |
| 74 | CCAATGGCATTTCTCCACG found at 79256 line | TRIM22 | [10346 (TRIM22)](http://www.ncbi.nlm.nih.gov/gene/10346) | tripartite motif containing 22 | 5706345 .. 5706363 | 5,710,817..5,732,093 | 4472 |  |
| 75 | CCAATCAGGGACCTCCACG found at 622844 line | TSPAN18 | [90139 (TSPAN18)](http://www.ncbi.nlm.nih.gov/gene/90139) | tetraspanin 18 | 44844679 .. 44844697 | 44,748,731..44,953,978 |  | element present inside the gene |
| 76 | CCAATGTCCGGTGACCACG found at 647748 line | ATG13 | [9776 (ATG13)](http://www.ncbi.nlm.nih.gov/gene/9776) | autophagy related 13 | 46637721 .. 46637739 | 46,638,826..46,697,569 | 1105 |  |
| 77 | CCAATGGAAACAACCCACG found at 949555 line | PPP6R3 | [55291 (PPP6R3)](http://www.ncbi.nlm.nih.gov/gene/55291) | protein phosphatase 6, regulatory subunit 3 | 68367838 .. 68367856 | 68,228,186..68,382,802 |  | element present inside the gene |
| 78 | CCAATTCTTTATCTCCACG found at 973962 line | PPFIA1 | [8500 (PPFIA1)](http://www.ncbi.nlm.nih.gov/gene/8500) | protein tyrosine phosphatase, receptor type, f polypeptide (PTPRF), interacting protein (liprin), alpha 1 | 70125113 .. 70125131 | 70,116,806..70,230,607 |  | element present inside the gene |
| 79 | CCAATAAGTTTTCTCCACG found at 1282269 line | FAT3 | [120114 (FAT3)](http://www.ncbi.nlm.nih.gov/gene/120114) | FAT atypical cadherin 3 | 92323234 .. 92323252 | 92,047,446..92,629,636 |  | element present inside the gene |
| 80 | CCAATATGGTGAAACCACG found at 1650679 line | FOXR1 | [283150 (FOXR1)](http://www.ncbi.nlm.nih.gov/gene/283150) | forkhead box R1 | 118848738 .. 118848756 | 118,842,417..118,851,997 |  | element present inside the gene |
| 81 | CCAATAAATGCAAACCACG found at 1668738 line | POU2F3 | [25833 (POU2F3)](http://www.ncbi.nlm.nih.gov/gene/25833) | POU class 2 homeobox 3 | 120149019 .. 120149037 | 120,107,349..120,190,653 |  | element present inside the gene |
| 82 | CCAATGAGAAGGGGCCACG found at 36465 line | CACNA1C | [775 (CACNA1C)](http://www.ncbi.nlm.nih.gov/gene/775) | calcium channel, voltage-dependent, L type, alpha 1C subunit | 2625385 .. 2625403 | 2,079,952..2,807,115 |  | element present inside the gene |
| 83 | CCAATATGGTAAAACCACG found at 94836 line | COPS7A | [50813 (COPS7A)](http://www.ncbi.nlm.nih.gov/gene/50813) | COP9 signalosome subunit 7A | 6828048 .. 6828066 | 6,833,150..6,841,041 | 5102 |  |
| 84 | CCAATGGGCTCTCACCACG found at 448324 line | BICD1 | [636 (BICD1)](http://www.ncbi.nlm.nih.gov/gene/636) | bicaudal D homolog 1 (Drosophila) | 32279221 .. 32279239 | 32,259,713..32,536,567 |  | element present inside the gene |
| 85 | CCAATGAGTCATTTCCACG found at 1052231 line | GLIPR1L1 | [256710 (GLIPR1L1)](http://www.ncbi.nlm.nih.gov/gene/256710) | GLI pathogenesis-related 1 like 1 | 75760525 .. 75760543 | 75,727,550..75,764,170 |  | element present inside the gene |
| 86 | CCAATCGGAAGGAGCCACG found at 1448946 line | HSP90B1 | [7184 (HSP90B1)](http://www.ncbi.nlm.nih.gov/gene/7184) | heat shock protein 90kDa beta (Grp94), member 1 | 104323994 .. 104324012 | 104,324,112..104,341,708 | 118 |  |
| 87 |  | MIR3652 | [100500842 (MIR3652)](http://www.ncbi.nlm.nih.gov/gene/100500842) | microRNA 3652 | 104323994 .. 104324012 | 104,324,203..104,324,333 | 209 |  |
| 88 | CCAATGGGAGTGGCCCACG found at 1512625 line | FICD | [11153 (FICD)](http://www.ncbi.nlm.nih.gov/gene/11153) | FIC domain containing | 108908900 .. 108908918 | 108,909,051..108,913,380 | 151 |  |
| 89 | CCAATATGGTGAAACCACG found at 627234 line | SMARCE1P5 (Pseudo) | [400129 (SMARCE1P5)](http://www.ncbi.nlm.nih.gov/gene/400129) | SWI/SNF related, matrix associated, actin dependent regulator of chromatin, subfamily e, member 1 pseudogene 5 | 45160720 .. 45160738 | 45,168,994..45,170,285 | 8274 |  |
| 90 | CCAATCACCCCCCACCACG found at 1555132 line | TEX29 | [121793 (TEX29)](http://www.ncbi.nlm.nih.gov/gene/121793) | testis expressed 29 | 111969362 .. 111969380 | 111,973,015..111,996,594 | 3653 |  |
| 91 | CCAATATCTGTTCTCCACG found at 1586591 line | TFDP1 | [7027 (TFDP1)](http://www.ncbi.nlm.nih.gov/gene/7027) | transcription factor Dp-1 | 114234399 .. 114234417 | 114,239,003..114,295,788 | 4604 |  |
| 92 | CCAATACGGTGAAACCACG found at 551569 line | MIA2 | [117153 (MIA2)](http://www.ncbi.nlm.nih.gov/gene/117153) | melanoma inhibitory activity 2 | 39712881 .. 39712899 | 39,703,125..39,722,575 |  | element present inside the gene |
| 93 | CCAATATAACGAAACCACG found at 1035627 line | LIN52 | [91750 (LIN52)](http://www.ncbi.nlm.nih.gov/gene/91750) | lin-52 homolog (C. elegans) | 74565033 .. 74565051 | 74,551,656..74,667,117 |  | element present inside the gene |
| 94 | CCAATAATTTTTCTCCACG found at 1064360 line | GPATCH2L | [55668 (GPATCH2L)](http://www.ncbi.nlm.nih.gov/gene/55668) | G patch domain containing 2-like | 76633793 .. 76633811 | 76,618,255..76,671,239 |  | element present inside the gene |
| 95 | CCAATGCTAGCTGCCCACG found at 380923 line | GABRG3 | [2567 (GABRG3)](http://www.ncbi.nlm.nih.gov/gene/2567) | gamma-aminobutyric acid (GABA) A receptor, gamma 3 | 27426327 .. 27426345 | 27,216,429..27,778,373 |  | element present inside the gene |
| 96 | CCAATGATTCTAGTCCACG found at 450159 line | CHRNA7 (Partial stop) | [1139 (CHRNA7)](http://www.ncbi.nlm.nih.gov/gene/1139) | cholinergic receptor, nicotinic, alpha 7 (neuronal) | 32411362 .. 32411380 | 32,322,686..32,462,384 |  | element present inside the gene |
| 97 | CCAATGTTAACCTCCCACG found at 796512 line | TCF12 | [6938 (TCF12)](http://www.ncbi.nlm.nih.gov/gene/6938) | transcription factor 12 | 57348767 .. 57348785 | 57,210,833..57,580,716 |  | element present inside the gene |
| 98 | CCAATTCCAGCCGCCCACG found at 837420 line | FOXB1 | [27023 (FOXB1)](http://www.ncbi.nlm.nih.gov/gene/27023) | forkhead box B1 | 60294092 .. 60294110 | 60,296,421..60,298,142 | 2329 |  |
| 99 | CCAATCACAGCTATCCACG found at 1130692 line | C15orf26 | [161502 (C15orf26)](http://www.ncbi.nlm.nih.gov/gene/161502) | chromosome 15 open reading frame 26 | 81409690 .. 81409708 | 81,391,749..81,441,516 |  | element present inside the gene |
| 100 | CCAATGCACCTGGCCCACG found at 1274280 line | SV2B | [9899 (SV2B)](http://www.ncbi.nlm.nih.gov/gene/9899) | synaptic vesicle glycoprotein 2B | 91748071 .. 91748089 | 91,642,996..91,844,539 |  | element present inside the gene |
| 101 | CCAATGTGTTGAAACCACG found at 34568 line | CCNF | [899 (CCNF)](http://www.ncbi.nlm.nih.gov/gene/899) | cyclin F | 2488809 .. 2488827 | 2,479,395..2,508,859 |  | element present inside the gene |
| 102 | CCAATCCTCCTCCTCCACG found at 414887 line | CDIPT-AS1 (Partial stop) | [440356 (CDIPT-AS1)](http://www.ncbi.nlm.nih.gov/gene/440356) | CDIPT antisense RNA 1 (head to head) | 29871740 .. 29871758 | 29,875,004..29,879,374 | 3264 |  |
| 103 | CCAATTAAATGACCCCACG found at 1183374 line | GSE1 | [23199 (GSE1)](http://www.ncbi.nlm.nih.gov/gene/23199) | Gse1 coiled-coil protein | 85202784 .. 85202802 | 85,203,152..85,709,812 | 368 |  |
| 104 | CCAATTCTCACTCACCACG found at 220197 line | ADORA2B | [136 (ADORA2B)](http://www.ncbi.nlm.nih.gov/gene/136) | adenosine A2b receptor | 15854097 .. 15854115 | 15,848,231..15,879,210 |  | element present inside the gene |
| 105 | CCAATGAGGCCTTCCCACG found at 599760 line | NMT1 | [4836 (NMT1)](http://www.ncbi.nlm.nih.gov/gene/4836) | N-myristoyltransferase 1 | 43182600 .. 43182618 | 43,138,322..43,186,384 |  | element present inside the gene |
| 106 | CCAATCCAGAAAGACCACG found at 1083418 line | CCDC40 | [55036 (CCDC40)](http://www.ncbi.nlm.nih.gov/gene/55036) | coiled-coil domain containing 40 | 78006000 .. 78006018 | 78,010,431..78,074,412 | 4431 |  |
| 107 | CCAATGTTTCTGGACCACG found at 446677 line | DTNA | [1837 (DTNA)](http://www.ncbi.nlm.nih.gov/gene/1837) | dystrobrevin, alpha | 32160620 .. 32160638 | 32,073,254..32,471,808 |  | element present inside the gene |
| 108 | CCAATCGGGGCGGTCCACG found at 618679 line | KATNAL2 | [83473 (KATNAL2)](http://www.ncbi.nlm.nih.gov/gene/83473) | katanin p60 subunit A-like 2 | 44556629 .. 44556647 | 44,497,565..44,628,614 |  | element present inside the gene |
| 109 | CCAATCGGGGCGGTCCACG found at 618762 line | KATNAL2 | [83473 (KATNAL2)](http://www.ncbi.nlm.nih.gov/gene/83473) | katanin p60 subunit A-like 2 | 44550714 .. 44550732 | 44,497,565..44,628,614 |  | element present inside the gene |
| 110 | CCAATCGGGGCGGTCCACG found at 618844 line | KATNAL2 | [83473 (KATNAL2)](http://www.ncbi.nlm.nih.gov/gene/83473) | katanin p60 subunit A-like 2 | 44544787 .. 44544805 | 44,497,565..44,628,614 |  | element present inside the gene |
| 111 | CCAATTTGTTGGTCCCACG found at 643118 line | CTIF | [9811 (CTIF)](http://www.ncbi.nlm.nih.gov/gene/9811) | CBP80/20-dependent translation initiation factor | 46304377 .. 46304395 | 46,065,427..46,389,588 |  | element present inside the gene |
| 112 | CCAATCCGCGACACCCACG found at 45782 line | CELF5 (Partial stop) | [60680 (CELF5)](http://www.ncbi.nlm.nih.gov/gene/60680) | CUGBP, Elav-like family member 5 | 3296210 .. 3296228 | 3,224,701..3,297,074 |  | element present inside the gene |
| 113 | CCAATAAACCGGCACCACG found at 71015 line | KDM4B | [23030 (KDM4B)](http://www.ncbi.nlm.nih.gov/gene/23030) | lysine (K)-specific demethylase 4B | 5112991 .. 5113009 | 4,969,123..5,153,609 |  | element present inside the gene |
| 114 | CCAATATGGTGAAACCACG found at 219070 line | CYP4F12 | [66002 (CYP4F12)](http://www.ncbi.nlm.nih.gov/gene/66002) | cytochrome P450, family 4, subfamily F, polypeptide 12 | 15772892 .. 15772910 | 15,783,567..15,807,984 | 10675 |  |
| 115 | CCAATGTAGTGAAACCACG found at 485093 line | UBA2 | [10054 (UBA2)](http://www.ncbi.nlm.nih.gov/gene/10054) | ubiquitin-like modifier activating enzyme 2 | 34926546 .. 34926564 | 34,919,264..34,960,798 |  | element present inside the gene |
| 116 | CCAATTCACTGAGACCACG found at 587577 line | CEACAM3 | [1084 (CEACAM3)](http://www.ncbi.nlm.nih.gov/gene/1084) | carcinoembryonic antigen-related cell adhesion molecule 3 | 42305413 .. 42305431 | 42,300,522..42,315,591 |  | element present inside the gene |
| 117 | CCAATCATAATCCCCCACG found at 779660 line | ZNF580 | [51157 (ZNF580)](http://www.ncbi.nlm.nih.gov/gene/51157) | zinc finger protein 580 | 56135432 .. 56135450 | 56,146,361..56,154,836 | 10929 |  |
| 118 | CCAATCAAATGGCACCACG found at 782611 line | NLRP4 | [147945 (NLRP4)](http://www.ncbi.nlm.nih.gov/gene/147945) | NLR family, pyrin domain containing 4 | 56347853 .. 56347871 | 56,347,944..56,393,221 | 91 |  |
| 119 | CCAATGAAGCCCCTCCACG found at 804478 line | ZNF17 | [7565 (ZNF17)](http://www.ncbi.nlm.nih.gov/gene/7565) | zinc finger protein 17 | 57922288 .. 57922306 | 57,922,529..57,933,307 | 241 |  |
| 120 | CCAATGCGCAGGTGCCACG found at 125674 line | PLCB4 | [5332 (PLCB4)](http://www.ncbi.nlm.nih.gov/gene/5332) | phospholipase C, beta 4 | 9048427 .. 9048445 | 9,049,357..9,461,463 | 241 |  |
| 121 | CCAATATTGCTTACCCACG found at 492620 line | SLC5A3 | [6526 (SLC5A3)](http://www.ncbi.nlm.nih.gov/gene/6526) | solute carrier family 5 (sodium/myo-inositol cotransporter), member 3 | 35468537 .. 35468555 | 35,445,503..35,515,334 |  | element present inside the gene |
| 122 |  | MRPS6 | [64968 (MRPS6)](http://www.ncbi.nlm.nih.gov/gene/64968) | mitochondrial ribosomal protein S6 | 35468537 .. 35468555 | 35,445,823..35,515,334 |  | element present inside the gene |
| 123 | CCAATGGAGGCAACCCACG found at 263369 line | DGCR5 (Partial stop) | [26220 (DGCR5)](http://www.ncbi.nlm.nih.gov/gene/26220) | DiGeorge syndrome critical region gene 5 (non-protein coding) | 18962438 .. 18962456 | 18,958,011..18,982,142 |  | element present inside the gene |
| 124 | CCAATCTGGGTTTGCCACG found at 276937 line | COMT | [1312 (COMT)](http://www.ncbi.nlm.nih.gov/gene/1312) | catechol-O-methyltransferase | 19939351 .. 19939369 | 19,929,263..19,957,498 |  | element present inside the gene |
| 125 | CCAATCAGATTCTCCCACG found at 319108 line | BCR | [613 (BCR)](http://www.ncbi.nlm.nih.gov/gene/613) | breakpoint cluster region | 23654711 .. 23654729 | 23,522,402..23,660,224 |  | element present inside the gene |
| 126 | CCAATCAGATTCTCCCACG found at 328539 line | IGL | [3535 (IGL)](http://www.ncbi.nlm.nih.gov/gene/3535) | immunoglobulin lambda locus | 22975625 .. 22975643 | 22,380,474..23,265,085 |  | element present inside the gene |
| 127 | CCAATGGGGAGGCCCCACG found at 549238 line | FUNDC2P4 (Pseudo) | [100127979 (FUNDC2P4)](http://www.ncbi.nlm.nih.gov/gene/100127979) | FUN14 domain containing 2 pseudogene 4 | 39545013 .. 39545031 | 39,551,541..39,552,101 | 6528 |  |
| 128 | CCAATTTCGTTTTCCCACG found at 202048 line | GLRA2 | [2742 (GLRA2)](http://www.ncbi.nlm.nih.gov/gene/2742) | glycine receptor, alpha 2 | 14547313 .. 14547331 | 14,547,420..14,749,934 | 107 |  |
| 129 | CCAATATGGTGAAACCACG found at 2008143 line | KDM6A | [7403 (KDM6A)](http://www.ncbi.nlm.nih.gov/gene/7403) | lysine (K)-specific demethylase 6A | 44739230 .. 44739248 | 44,732,419..44,972,024 |  | element present inside the gene |
| 130 | CCAATCTCAGCCCTCCACG found at 658764 line | ARAF | [369 (ARAF)](http://www.ncbi.nlm.nih.gov/gene/369) | v-raf murine sarcoma 3611 viral oncogene homolog | 47430880 .. 47430898 | 47,420,499..47,431,320 |  | element present inside the gene |
| 131 |  | TIMP1 | [7076 (TIMP1)](http://www.ncbi.nlm.nih.gov/gene/7076) | TIMP metallopeptidase inhibitor 1 | 47430880 .. 47430898 | 47,441,690..47,446,190 | 10810 |  |
| 132 | CCAATTTGGATTGACCACG found at 959770 line | EDA | [1896 (EDA)](http://www.ncbi.nlm.nih.gov/gene/1896) | ectodysplasin A | 69103313 .. 69103331 | 68,835,911..69,259,322 |  | element present inside the gene |
| 133 | CCAATTTATAGTAACCACG found at 1531667 line | PAK3 | [5063 (PAK3)](http://www.ncbi.nlm.nih.gov/gene/5063) | p21 protein (Cdc42/Rac)-activated kinase 3 | 110279931 .. 110279949 | 110,187,513..110,470,590 |  | element present inside the gene |
| 134 | CCAATCTCTGCCTCCCACG found at 1711030 line | STAG2 | [10735 (STAG2)](http://www.ncbi.nlm.nih.gov/gene/10735) | stromal antigen 2 | 123194057 .. 123194075 | 123,094,475..123,236,506 |  | element present inside the gene |
|  |  |  |  |  |  |  |  |  |
|  | **ERSE-II** |  |  |  |  |  |  |  |
| **SNO** | **ERSE element** | **Gene** | **gene ID** | **Gene name** | **Hit position** | **Gene position** | **10 Kb** | **Remark** |
| 1 | ATTGGACCACG found at 80952 line | PAPPA2 | [60676 (PAPPA2)](http://www.ncbi.nlm.nih.gov/gene/60676) | pappalysin 2 | 176703973 .. 176703983 | 176,432,307..176,814,737 |  | element present inside the gene |
| 2 | ATTGGACCACG found at 107845 line | ATP1A2 | [477 (ATP1A2)](http://www.ncbi.nlm.nih.gov/gene/477) | ATPase, Na+/K+ transporting, alpha 2 polypeptide | 160080318 .. 160080328 | 160,085,520..160,113,381 | 5202 |  |
| 3 | ATTGGACCACG found at 2223339 line | CAMTA1 (Partial stop) | [23261 (CAMTA1)](http://www.ncbi.nlm.nih.gov/gene/23261) | calmodulin binding transcription activator 1 | 7764690 .. 7764700 | 6,845,384..7,829,766 |  | element present inside the gene |
| 4 | ATTGGCCCACG found at 217378 line | RABGAP1L | [9910 (RABGAP1L)](http://www.ncbi.nlm.nih.gov/gene/9910) | RAB GTPase activating protein 1-like | 174741066 .. 174741076 | 174,128,552..174,964,445 |  | element present inside the gene |
| 5 | ATTGGCCCACG found at 931449 line | SGIP1 | [84251 (SGIP1)](http://www.ncbi.nlm.nih.gov/gene/84251) | SH3-domain GRB2-like (endophilin) interacting protein 1 | 67064223 .. 67064233 | 66,999,066..67,210,768 |  | element present inside the gene |
| 6 | ATTGGCCCACG found at 1181268 line | HTR6 | [3362 (HTR6)](http://www.ncbi.nlm.nih.gov/gene/3362) | 5-hydroxytryptamine (serotonin) receptor 6, G protein-coupled | 19987265 .. 19987275 | 19,991,780..20,006,055 | 4515 |  |
| 7 | ATTGGCCCACG found at 2426961 line | FHAD1 | [114827 (FHAD1)](http://www.ncbi.nlm.nih.gov/gene/114827) | forkhead-associated (FHA) phosphopeptide binding domain 1 | 15651081 .. 15651091 | 15,573,730..15,724,767 |  | element present inside the gene |
| 8 | ATTGGGCCACG found at 185367 line | KIF26B | [55083 (KIF26B)](http://www.ncbi.nlm.nih.gov/gene/55083) | kinesin family member 26B | 245765575 .. 245765585 | 245,318,287..245,866,428 |  | element present inside the gene |
| 9 | ATTGGGCCACG found at 881019 line | PAPPA2 | [60676 (PAPPA2)](http://www.ncbi.nlm.nih.gov/gene/60676) | pappalysin 2 | 176583487 .. 176583497 | 176,432,307..176,814,737 |  | element present inside the gene |
| 10 | ATTGGGCCACG found at 2380410 line | PDE4B | [5142 (PDE4B)](http://www.ncbi.nlm.nih.gov/gene/5142) | phosphodiesterase 4B, cAMP-specific | 66334387 .. 66334397 | 66,258,193..66,840,262 |  | element present inside the gene |
| 11 | ATTGGGCCACG found at 2524822 line | ST3GAL3 (Partial stop) | [6487 (ST3GAL3)](http://www.ncbi.nlm.nih.gov/gene/6487) | ST3 beta-galactoside alpha-2,3-sialyltransferase 3 | 44324790 .. 44324800 | 44,173,204..44,396,837 |  | element present inside the gene |
| 12 | ATTGGGCCACG found at 2687029 line | FHAD1 | [114827 (FHAD1)](http://www.ncbi.nlm.nih.gov/gene/114827) | forkhead-associated (FHA) phosphopeptide binding domain 1 | 15702448 .. 15702458 | 15,573,730..15,724,767 |  | element present inside the gene |
| 13 | ATTGGTCCACG found at 1232536 line | SRGAP2 (Partial stop) | [23380 (SRGAP2)](http://www.ncbi.nlm.nih.gov/gene/23380) | SLIT-ROBO Rho GTPase activating protein 2 | 206620702 .. 206620712 | 206,516,197..206,637,783 |  | element present inside the gene |
| 14 | ATTGGTCCACG found at 897344 line | ANKRD36 | [375248 (ANKRD36)](http://www.ncbi.nlm.nih.gov/gene/375248) | ankyrin repeat domain 36 | 97919499 .. 97919509 | 97,778,923..97,930,258 |  | element present inside the gene |
| 15 | ATTGGACCACG found at 749491 line | ITGA4 | [3676 (ITGA4)](http://www.ncbi.nlm.nih.gov/gene/3676) | integrin, alpha 4 (antigen CD49D, alpha 4 subunit of VLA-4 receptor) | 182351100 .. 182351110 | 182,321,619..182,402,474 |  | element present inside the gene |
| 16 | ATTGGACCACG found at 2075773 line | EPC2 | [26122 (EPC2)](http://www.ncbi.nlm.nih.gov/gene/26122) | enhancer of polycomb homolog 2 (Drosophila) | 149455555 .. 149455565 | 149,402,560..149,545,136 |  | element present inside the gene |
| 17 | ATTGGGCCACG found at 2138803 line | FAM117B | [150864 (FAM117B)](http://www.ncbi.nlm.nih.gov/gene/150864) | family with sequence similarity 117, member B | 203561100 .. 203561110 | 203,499,901..203,634,480 |  | element present inside the gene |
| 18 | ATTGGGCCACG found at 2184621 line | ZNF804A | [91752 (ZNF804A)](http://www.ncbi.nlm.nih.gov/gene/91752) | zinc finger protein 804A | 185710568 .. 185710578 | 185,463,093..185,804,214 |  | element present inside the gene |
| 19 | ATTGGGCCACG found at 2579315 line | GPD2 | [2820 (GPD2)](http://www.ncbi.nlm.nih.gov/gene/2820) | glycerol-3-phosphate dehydrogenase 2 (mitochondrial) | 157292613 .. 157292623 | 157,291,965..157,442,915 |  | element present inside the gene |
| 20 | ATTGGGCCACG found at 3267685 line | LIMS1 | [3987 (LIMS1)](http://www.ncbi.nlm.nih.gov/gene/3987) | LIM and senescent cell antigen-like domains 1 | 109160330 .. 109160340 | 109,150,811..109,303,702 |  | element present inside the gene |
| 21 | ATTGGCCCACG found at 626808 line | DIS3L2 (Partial stop) | [129563 (DIS3L2)](http://www.ncbi.nlm.nih.gov/gene/129563) | DIS3 mitotic control homolog (S. cerevisiae)-like 2 | 233084368 .. 233084378 | 232,826,293..233,208,678 |  | element present inside the gene |
| 22 | ATTGGCCCACG found at 962581 line | PTCD3 | [55037 (PTCD3)](http://www.ncbi.nlm.nih.gov/gene/55037) | pentatricopeptide repeat domain 3 | 86362167 .. 86362177 | 86,333,305..86,369,280 |  | element present inside the gene |
| 23 | ATTGGCCCACG found at 1199476 line | ANTXR1 | [84168 (ANTXR1)](http://www.ncbi.nlm.nih.gov/gene/84168) | anthrax toxin receptor 1 | 69305738 .. 69305748 | 69,240,276..69,476,459 |  | element present inside the gene |
| 24 | ATTGGTCCACG found at 230363 line | CCRL2 | [9034 (CCRL2)](http://www.ncbi.nlm.nih.gov/gene/9034) | chemokine (C-C motif) receptor-like 2 | 46442704 .. 46442714 | 46,448,721..46,454,488 | 6017 |  |
| 25 | ATTGGGCCACG found at 442351 line | EEFSEC | [60678 (EEFSEC)](http://www.ncbi.nlm.nih.gov/gene/60678) | eukaryotic elongation factor, selenocysteine-tRNA-specific | 128123278 .. 128123288 | 127,872,302..128,127,489 |  | element present inside the gene |
| 26 | ATTGGACCACG found at 680313 line | LPP | [4026 (LPP)](http://www.ncbi.nlm.nih.gov/gene/4026) | LIM domain containing preferred translocation partner in lipoma | 188280989 .. 188280999 | 187,871,097..188,608,460 |  | element present inside the gene |
| 27 | ATTGGACCACG found at 2067270 line | HPS3 | [84343 (HPS3)](http://www.ncbi.nlm.nih.gov/gene/84343) | Hermansky-Pudlak syndrome 3 | 148843329 .. 148843339 | 148,847,371..148,891,305 | 4042 |  |
| 28 | ATTGGACCACG found at 2615015 line | ARIH2 | [10425 (ARIH2)](http://www.ncbi.nlm.nih.gov/gene/10425) | ariadne homolog 2 (Drosophila) | 48982447 .. 48982457 | 48,956,265..49,022,974 |  | element present inside the gene |
| 29 | ATTGGACCACG found at 90987 line | BANK1 | [55024 (BANK1)](http://www.ncbi.nlm.nih.gov/gene/55024) | B-cell scaffold protein with ankyrin repeats 1 | 102849910 .. 102849920 | 102,711,764..102,995,969 |  | element present inside the gene |
| 30 | ATTGGCCCACG found at 671087 line | TBC1D1 | [23216 (TBC1D1)](http://www.ncbi.nlm.nih.gov/gene/23216) | TBC1 (tre-2/USP6, BUB2, cdc16) domain family, member 1 | 37889484 .. 37889494 | 37,889,606..38,140,796 | 122 |  |
| 31 | ATTGGGCCACG found at 2646839 line | C4orf22 | [255119 (C4orf22)](http://www.ncbi.nlm.nih.gov/gene/255119) | chromosome 4 open reading frame 22 | 81750232 .. 81750242 | 81,256,861..81,884,910 |  | element present inside the gene |
| 32 | ATTGGTCCACG found at 1800001 line | TENM2 | [57451 (TENM2)](http://www.ncbi.nlm.nih.gov/gene/57451) | teneurin transmembrane protein 2 | 167025056 .. 167025066 | 166,406,083..167,691,162 |  | element present inside the gene |
| 33 | ATTGGCCCACG found at 376224 line | ZNF454 | [285676 (ZNF454)](http://www.ncbi.nlm.nih.gov/gene/285676) | zinc finger protein 454 | 178370200 .. 178370210 | 178,368,194..178,393,218 |  | element present inside the gene |
| 34 | ATTGGGCCACG found at 2189221 line | RASGRF2 | [5924 (RASGRF2)](http://www.ncbi.nlm.nih.gov/gene/5924) | Ras protein-specific guanine nucleotide-releasing factor 2 | 80460258 .. 80460268 | 80,256,491..80,525,981 |  | element present inside the gene |
| 35 | ATTGGACCACG found at 1624936 line | SCGB3A2 | [117156 (SCGB3A2)](http://www.ncbi.nlm.nih.gov/gene/117156) | secretoglobin, family 3A, member 2 | 147256727 .. 147256737 | 147,258,274..147,261,756 | 1547 |  |
| 36 | ATTGGTCCACG found at 56651 line | RNF146 (Partial stop) | [81847 (RNF146)](http://www.ncbi.nlm.nih.gov/gene/81847) | ring finger protein 146 | 127605341 .. 127605351 | 127,587,827..127,609,707 |  | element present inside the gene |
| 37 | ATTGGTCCACG found at 1772298 line | C6orf201 | [404220 (C6orf201)](http://www.ncbi.nlm.nih.gov/gene/404220) | chromosome 6 open reading frame 201 | 4078717 .. 4078727 | 4,079,440..4,131,000 | 723 |  |
| 38 | ATTGGACCACG found at 173693 line | LY86 | [9450 (LY86)](http://www.ncbi.nlm.nih.gov/gene/9450) | lymphocyte antigen 86 | 6627799 .. 6627809 | 6,588,934..6,655,216 |  | element present inside the gene |
| 39 | ATTGGGCCACG found at 547366 line | NRF1 | [4899 (NRF1)](http://www.ncbi.nlm.nih.gov/gene/4899) | nuclear respiratory factor 1 | 129357069 .. 129357079 | 129,251,555..129,396,922 |  | element present inside the gene |
| 40 | ATTGGGCCACG found at 1744824 line | HECW1 | [23072 (HECW1)](http://www.ncbi.nlm.nih.gov/gene/23072) | HECT, C2 and WW domain containing E3 ubiquitin protein ligase 1 | 43430958 .. 43430968 | 43,152,198..43,603,213 |  | element present inside the gene |
| 41 | ATTGGGCCACG found at 1796628 line | POU6F2 | [11281 (POU6F2)](http://www.ncbi.nlm.nih.gov/gene/11281) | POU class 6 homeobox 2 | 39410257 .. 39410267 | 39,017,609..39,504,390 |  | element present inside the gene |
| 42 | ATTGGCCCACG found at 1029633 line | LRGUK | [136332 (LRGUK)](http://www.ncbi.nlm.nih.gov/gene/136332) | leucine-rich repeats and guanylate kinase domain containing | 133817219 .. 133817229 | 133,812,105..133,948,933 |  | element present inside the gene |
| 43 | ATTGGCCCACG found at 1858574 line | GTF2I | [2969 (GTF2I)](http://www.ncbi.nlm.nih.gov/gene/2969) | general transcription factor IIi | 74133460 .. 74133470 | 74,071,991..74,175,022 |  | element present inside the gene |
| 44 | ATTGGACCACG found at 990813 line | EN2 | [2020 (EN2)](http://www.ncbi.nlm.nih.gov/gene/2020) | engrailed homeobox 2 | 155242596 .. 155242606 | 155,250,824..155,257,526 | 8228 |  |
| 45 | ATTGGCCCACG found at 173181 line | RDH10 | [157506 (RDH10)](http://www.ncbi.nlm.nih.gov/gene/157506) | retinol dehydrogenase 10 (all-trans) | 74200812 .. 74200822 | 74,206,837..74,237,520 | 6025 |  |
| 46 | ATTGGCCCACG found at 388817 line | COL27A1 | [85301 (COL27A1)](http://www.ncbi.nlm.nih.gov/gene/85301) | collagen, type XXVII, alpha 1 | 117035645 .. 117035655 | 116,918,231..117,072,975 |  | element present inside the gene |
| 47 | ATTGGCCCACG found at 1889510 line | KANK1 | [23189 (KANK1)](http://www.ncbi.nlm.nih.gov/gene/23189) | KN motif and ankyrin repeat domains 1 | 731780 .. 731790 | 470,294..746,106 |  | element present inside the gene |
| 48 | ATTGGGCCACG found at 630480 line | PCSK5 | [5125 (PCSK5)](http://www.ncbi.nlm.nih.gov/gene/5125) | proprotein convertase subtilisin/kexin type 5 | 78578075 .. 78578085 | 78,505,560..78,977,255 |  | element present inside the gene |
| 49 | ATTGGGCCACG found at 1634115 line | RUSC2 | [9853 (RUSC2)](http://www.ncbi.nlm.nih.gov/gene/9853) | RUN and SH3 domain containing 2 | 35489848 .. 35489858 | 35,490,007..35,561,895 | 159 |  |
| 50 | ATTGGACCACG found at 1844043 line | PALM2 | [114299 (PALM2)](http://www.ncbi.nlm.nih.gov/gene/114299) | paralemmin 2 | 112445246 .. 112445256 | 112,403,068..112,713,756 |  | element present inside the gene |
| 51 | ATTGGCCCACG found at 1140238 line | SLC18A2 | [6571 (SLC18A2)](http://www.ncbi.nlm.nih.gov/gene/6571) | solute carrier family 18 (vesicular monoamine transporter), member 2 | 119025958 .. 119025968 | 119,000,584..119,038,941 |  | element present inside the gene |
| 52 | ATTGGCCCACG found at 1653140 line | DYDC2 (Partial stop) | [84332 (DYDC2)](http://www.ncbi.nlm.nih.gov/gene/84332) | DPY30 domain containing 2 | 82097005 .. 82097015 | 82,104,501..82,127,829 | 7496 |  |
| 53 | ATTGGACCACG found at 148023 line | ZNF365 | [22891 (ZNF365)](http://www.ncbi.nlm.nih.gov/gene/22891) | zinc finger protein 365 | 64301081 .. 64301091 | 64,133,916..64,431,771 |  | element present inside the gene |
| 54 | ATTGGGCCACG found at 338009 line | FDX1 | [2230 (FDX1)](http://www.ncbi.nlm.nih.gov/gene/2230) | ferredoxin 1 | 110290809 .. 110290819 | 110,300,661..110,335,608 | 9852 |  |
| 55 | ATTGGGCCACG found at 1317471 line | ENDOD1 | [23052 (ENDOD1)](http://www.ncbi.nlm.nih.gov/gene/23052) | endonuclease domain containing 1 | 94857799 .. 94857809 | 94,822,974..94,865,815 |  | element present inside the gene |
| 56 | ATTGGGCCACG found at 1477268 line | RBM4 | [5936 (RBM4)](http://www.ncbi.nlm.nih.gov/gene/5936) | RNA binding motif protein 4 | 66426360 .. 66426370 | 66,406,088..66,435,856 |  | element present inside the gene |
| 57 | ATTGGGCCACG found at 1844348 line | SLC22A18 | [5002 (SLC22A18)](http://www.ncbi.nlm.nih.gov/gene/5002) | solute carrier family 22, member 18 | 2945010 .. 2945020 | 2,920,951..2,946,476 |  | element present inside the gene |
| 58 | ATTGGCCCACG found at 79292 line | UBASH3B | [84959 (UBASH3B)](http://www.ncbi.nlm.nih.gov/gene/84959) | ubiquitin associated and SH3 domain containing B | 122543419 .. 122543429 | 122,526,398..122,685,187 |  | element present inside the gene |
| 59 | ATTGGCCCACG found at 1701994 line | TRIM22 | [10346 (TRIM22)](http://www.ncbi.nlm.nih.gov/gene/10346) | tripartite motif containing 22 | 5708901 .. 5708911 | 5,710,817..5,732,093 | 1916 |  |
| 60 | ATTGGACCACG found at 375281 line | VPS11 | [55823 (VPS11)](http://www.ncbi.nlm.nih.gov/gene/55823) | vacuolar protein sorting 11 homolog (S. cerevisiae) | 118928091 .. 118928101 | 118,938,493..118,952,688 | 10402 |  |
| 61 | ATTGGACCACG found at 774178 line | VPS11 | [55823 (VPS11)](http://www.ncbi.nlm.nih.gov/gene/55823) | vacuolar protein sorting 11 homolog (S. cerevisiae) | 118927985 .. 118927995 | 118,938,493..118,952,688 | 10508 |  |
| 62 | ATTGGTCCACG found at 436001 line | HS6ST3 | [266722 (HS6ST3)](http://www.ncbi.nlm.nih.gov/gene/266722) | heparan sulfate 6-O-sulfotransferase 3 | 96834375 .. 96834385 | 96,743,093..97,491,816 |  | element present inside the gene |
| 63 | ATTGGCCCACG found at 1444644 line | COL4A2 | [1284 (COL4A2)](http://www.ncbi.nlm.nih.gov/gene/1284) | collagen, type IV, alpha 2 | 111045430 .. 111045440 | 110,959,631..111,165,374 |  | element present inside the gene |
| 64 | ATTGGCCCACG found at 493209 line | CCNK | [8812 (CCNK)](http://www.ncbi.nlm.nih.gov/gene/8812) | cyclin K | 99947275 .. 99947285 | 99,947,695..99,977,852 | 420 |  |
| 65 | ATTGGCCCACG found at 538008 line | NRXN3 | [9369 (NRXN3)](http://www.ncbi.nlm.nih.gov/gene/9369) | neurexin 3 | 79219299 .. 79219309 | 78,636,716..80,334,633 |  | element present inside the gene |
| 66 | ATTGGCCCACG found at 1388158 line | FAM177A1 | [283635 (FAM177A1)](http://www.ncbi.nlm.nih.gov/gene/283635) | family with sequence similarity 177, member A1 | 35510958 .. 35510968 | 35,514,113..35,552,589 | 3155 |  |
| 67 | ATTGGTCCACG found at 1082307 line | AHSA1 | [10598 (AHSA1)](http://www.ncbi.nlm.nih.gov/gene/10598) | AHA1, activator of heat shock 90kDa protein ATPase homolog 1 (yeast) | 77925979 .. 77925989 | 77,924,373..77,935,815 |  | element present inside the gene |
| 68 | ATTGGACCACG found at 774295 line | CALM1 | [801 (CALM1)](http://www.ncbi.nlm.nih.gov/gene/801) | calmodulin 1 (phosphorylase kinase, delta) | 90858851 .. 90858861 | 90,863,327..90,874,619 | 4476 |  |
| 69 | ATTGGACCACG found at 1103566 line | NRXN3 | [9369 (NRXN3)](http://www.ncbi.nlm.nih.gov/gene/9369) | neurexin 3 | 79456639 .. 79456649 | 78,636,716..80,334,633 |  | element present inside the gene |
| 70 | ATTGGACCACG found at 1261930 line | FBXO34 | [55030 (FBXO34)](http://www.ncbi.nlm.nih.gov/gene/55030) | F-box protein 34 | 55749120 .. 55749130 | 55,738,021..55,820,329 |  | element present inside the gene |
| 71 | ATTGGTCCACG found at 594677 line | SNAP23 | [8773 (SNAP23)](http://www.ncbi.nlm.nih.gov/gene/8773) | synaptosomal-associated protein, 23kDa | 42816592 .. 42816602 | 42,783,442..42,825,259 |  | element present inside the gene |
| 72 | ATTGGGCCACG found at 764787 line | IGF1R | [3480 (IGF1R)](http://www.ncbi.nlm.nih.gov/gene/3480) | insulin-like growth factor 1 receptor | 99332866 .. 99332876 | 99,192,272..99,507,759 |  | element present inside the gene |
| 73 | ATTGGCCCACG found at 784928 line | ASB7 | [140460 (ASB7)](http://www.ncbi.nlm.nih.gov/gene/140460) | ankyrin repeat and SOCS box containing 7 | 101161062 .. 101161072 | 101,142,755..101,191,906 |  | element present inside the gene |
| 74 | ATTGGACCACG found at 839484 line | SV2B | [9899 (SV2B)](http://www.ncbi.nlm.nih.gov/gene/9899) | synaptic vesicle glycoprotein 2B | 91696532 .. 91696542 | 91,642,996..91,844,539 |  | element present inside the gene |
| 75 | ATTGGGCCACG found at 97369 line | BANP | [54971 (BANP)](http://www.ncbi.nlm.nih.gov/gene/54971) | BTG3 associated nuclear protein | 87991410 .. 87991420 | 87,984,231..88,110,924 |  | element present inside the gene |
| 76 | ATTGGGCCACG found at 243382 line | WFDC1 | [58189 (WFDC1)](http://www.ncbi.nlm.nih.gov/gene/58189) | WAP four-disulfide core domain 1 | 84323070 .. 84323080 | 84,328,244..84,363,450 | 5174 |  |
| 77 | ATTGGGCCACG found at 1170023 line | HERPUD1 | [9709 (HERPUD1)](http://www.ncbi.nlm.nih.gov/gene/9709) | homocysteine-inducible, endoplasmic reticulum stress-inducible, ubiquitin-like domain member 1 | 56965937 .. 56965947 | 56,965,974..56,977,793 | 37 |  |
| 78 | ATTGGGCCACG found at 1222105 line | RBFOX1 | [54715 (RBFOX1)](http://www.ncbi.nlm.nih.gov/gene/54715) | RNA binding protein, fox-1 homolog (C. elegans) 1 | 7010479 .. 7010489 | 5,289,469..7,763,342 |  | element present inside the gene |
| 79 | ATTGGCCCACG found at 233466 line | PLCG2 | [5336 (PLCG2)](http://www.ncbi.nlm.nih.gov/gene/5336) | phospholipase C, gamma 2 (phosphatidylinositol-specific) | 81869925 .. 81869935 | 81,812,899..81,996,290 |  | element present inside the gene |
| 80 | ATTGGCCCACG found at 324192 line | SCNN1B | [6338 (SCNN1B)](http://www.ncbi.nlm.nih.gov/gene/6338) | sodium channel, non-voltage-gated 1, beta subunit | 23341685 .. 23341695 | 23,313,591..23,392,620 |  | element present inside the gene |
| 81 | ATTGGGCCACG found at 403527 line | (SEPT9) | [10801 (SEPT9)](http://www.ncbi.nlm.nih.gov/gene/10801) | septin 9 | 75388580 .. 75388590 | 75,277,492..75,496,678 |  | element present inside the gene |
| 82 | ATTGGGCCACG found at 533719 line | WIPF2 | [147179 (WIPF2)](http://www.ncbi.nlm.nih.gov/gene/147179) | WAS/WASL interacting protein family, member 2 | 38427610 .. 38427620 | 38,375,556..38,438,944 |  | element present inside the gene |
| 83 | ATTGGGCCACG found at 1099784 line | DNAH9 | [1770 (DNAH9)](http://www.ncbi.nlm.nih.gov/gene/1770) | dynein, axonemal, heavy chain 9 | 11700546 .. 11700556 | 11,501,748..11,873,065 |  | element present inside the gene |
| 84 | ATTGGTCCACG found at 800714 line | DHX40 | [79665 (DHX40)](http://www.ncbi.nlm.nih.gov/gene/79665) | DEAH (Asp-Glu-Ala-His) box polypeptide 40 | 57651287 .. 57651297 | 57,642,886..57,685,713 |  | element present inside the gene |
| 85 | ATTGGCCCACG found at 138181 line | ZNF473 | [25888 (ZNF473)](http://www.ncbi.nlm.nih.gov/gene/25888) | zinc finger protein 473 | 50542825 .. 50542835 | 50,528,786..50,552,033 |  | element present inside the gene |
| 86 | ATTGGCCCACG found at 613236 line | RUVBL2 | [10856 (RUVBL2)](http://www.ncbi.nlm.nih.gov/gene/10856) | RuvB-like 2 (E. coli) | 49496640 .. 49496650 | 49,496,738..49,519,183 | 98 |  |
| 87 | ATTGGCCCACG found at 641660 line | QPCTL | [54814 (QPCTL)](http://www.ncbi.nlm.nih.gov/gene/54814) | glutaminyl-peptide cyclotransferase-like | 46199395 .. 46199405 | 46,195,741..46,207,248 |  | element present inside the gene |
| 88 | ATTGGCCCACG found at 701985 line | PIN1 (Partial stop) | [5300 (PIN1)](http://www.ncbi.nlm.nih.gov/gene/5300) | peptidylprolyl cis/trans isomerase, NIMA-interacting 1 | 9948946 .. 9948956 | 9,945,883..9,960,365 |  | element present inside the gene |
| 89 | ATTGGCCCACG found at 794498 line | NFIC | [4782 (NFIC)](http://www.ncbi.nlm.nih.gov/gene/4782) | nuclear factor I/C (CCAAT-binding transcription factor) | 3367208 .. 3367218 | 3,359,561..3,469,215 |  | element present inside the gene |
| 90 | ATTGGACCACG found at 194989 line | RPS19 | [6223 (RPS19)](http://www.ncbi.nlm.nih.gov/gene/6223) | ribosomal protein S19 | 42363310 .. 42363320 | 42,363,988..42,375,484 | 678 |  |
| 91 | ATTGGACCACG found at 588381 line | CC2D1A | [54862 (CC2D1A)](http://www.ncbi.nlm.nih.gov/gene/54862) | coiled-coil and C2 domain containing 1A | 14039085 .. 14039095 | 14,016,956..14,041,693 |  | element present inside the gene |
| 92 | ATTGGTCCACG found at 589005 line | ZNF175 | [7728 (ZNF175)](http://www.ncbi.nlm.nih.gov/gene/7728) | zinc finger protein 175 | 52074921 .. 52074931 | 52,074,531..52,092,991 |  | element present inside the gene |
| 93 | ATTGGTCCACG found at 723264 line | ARHGEF1 | [9138 (ARHGEF1)](http://www.ncbi.nlm.nih.gov/gene/9138) | Rho guanine nucleotide exchange factor (GEF) 1 | 42408210 .. 42408220 | 42,387,240..42,411,604 |  | element present inside the gene |
| 94 | ATTGGGCCACG found at 521718 line | FAM83D | [81610 (FAM83D)](http://www.ncbi.nlm.nih.gov/gene/81610) | family with sequence similarity 83, member D | 37563563 .. 37563573 | 37,554,955..37,581,703 |  | element present inside the gene |
| 95 | ATTGGGCCACG found at 824169 line | CDS2 | [8760 (CDS2)](http://www.ncbi.nlm.nih.gov/gene/8760) | CDP-diacylglycerol synthase (phosphatidate cytidylyltransferase) 2 | 5153212 .. 5153222 | 5,107,407..5,178,533 |  | element present inside the gene |
| 96 | ATTGGTCCACG found at 169006 line | CDH4 | [1002 (CDH4)](http://www.ncbi.nlm.nih.gov/gene/1002) | cadherin 4, type 1, R-cadherin (retinal) | 60409102 .. 60409112 | 59,827,482..60,515,673 |  | element present inside the gene |
| 97 | ATTGGTCCACG found at 832818 line | CDH4 | [1002 (CDH4)](http://www.ncbi.nlm.nih.gov/gene/1002) | cadherin 4, type 1, R-cadherin (retinal) | 59962774 .. 59962784 | 59,827,482..60,515,673 |  | element present inside the gene |
| 98 | ATTGGCCCACG found at 505790 line | DNAJC5 | [80331 (DNAJC5)](http://www.ncbi.nlm.nih.gov/gene/80331) | DnaJ (Hsp40) homolog, subfamily C, member 5 | 62557912 .. 62557922 | 62,526,455..62,567,384 |  | element present inside the gene |
| 99 | ATTGGCCCACG found at 868862 line | CTNNBL1 | [56259 (CTNNBL1)](http://www.ncbi.nlm.nih.gov/gene/56259) | catenin, beta like 1 | 36416785 .. 36416795 | 36,322,357..36,500,531 |  | element present inside the gene |
| 100 | ATTGGTCCACG found at 559562 line | GRAP2 | [9402 (GRAP2)](http://www.ncbi.nlm.nih.gov/gene/9402) | GRB2-related adaptor protein 2 | 40288332 .. 40288342 | 40,297,086..40,369,347 | 8754 |  |
| 101 | ATTGGGCCACG found at 523765 line | CLCN4 | [1183 (CLCN4)](http://www.ncbi.nlm.nih.gov/gene/1183) | chloride channel, voltage-sensitive 4 | 10121437 .. 10121447 | 10,124,985..10,205,700 | 3548 |  |
| 102 | ATTGGCCCACG found at 479046 line | PCDH11X | [27328 (PCDH11X)](http://www.ncbi.nlm.nih.gov/gene/27328) | protocadherin 11 X-linked | 91764313 .. 91764323 | 91,034,260..91,878,229 |  | element present inside the gene |
| 103 | ATTGGCCCACG found at 1513569 line | SMS | [6611 (SMS)](http://www.ncbi.nlm.nih.gov/gene/6611) | spermine synthase | 22014044 .. 22014054 | 21,958,691..22,025,798 |  | element present inside the gene |
| 104 | ATTGGTCCACG found at 1145251 line | ZNF275 | [10838 (ZNF275)](http://www.ncbi.nlm.nih.gov/gene/10838) | zinc finger protein 275 | 152617631 .. 152617641 | 152,599,613..152,618,384 |  | element present inside the gene |
| 105 | ATTGGACCACG found at 2101084 line | MAGEB10 | [139422 (MAGEB10)](http://www.ncbi.nlm.nih.gov/gene/139422) | melanoma antigen family B, 10 | 27827028 .. 27827038 | 27,826,107..27,841,131 |  | element present inside the gene |
| 106 | ATTGGGCCACG found at 268143 line | SURF6P1 (Pseudo) | [643470 (SURF6P1)](http://www.ncbi.nlm.nih.gov/gene/643470) | surfeit 6 pseudogene 1 | 19306155 .. 19306165 | 19,296,350..19,306,649 |  | element present inside the gene |
|  |  |  |  |  |  |  |  |  |
|  | **ERSE-III** |  |  |  |  |  |  |  |
| **SNO** | **ERSE element** | **Gene** | **gene ID** | **Gene name** | **Hit position** | **Gene position** | **10 Kb** | **Remark** |
| 1 | CCAATGTCTGTTGAACAGCTATCACGTTGAACCACG found at 52800 line | DFFB | [1677 (DFFB)](http://www.ncbi.nlm.nih.gov/gene/1677) | DNA fragmentation factor, 40kDa, beta polypeptide (caspase-activated DNase) | 3801473 .. 3801508 | 3,773,845..3,801,993 |  | element present inside the gene |
| 2 | CCAATTCTGCCACAGGCCCCGTCACCTTCTCCCACG found at 197504 line | KAZN | [23254 (KAZN)](http://www.ncbi.nlm.nih.gov/gene/23254) | kazrin, periplakin interacting protein | 14220183 .. 14220218 | 14,219,646..15,444,544 |  | element present inside the gene |
| 3 | CCAATTGCCTGGTAACAACATGTTCACACTTCCACG found at 243376 line | PADI1 | [29943 (PADI1)](http://www.ncbi.nlm.nih.gov/gene/29943) | peptidyl arginine deiminase, type I | 17522967 .. 17523002 | 17,531,621..17,572,501 | 8654 |  |
| 4 | CCAATTAGGCACCCAGGACTATCCAATTTGGCCACG found at 609684 line | SZT2 | [23334 (SZT2)](http://www.ncbi.nlm.nih.gov/gene/23334) | seizure threshold 2 homolog (mouse) | 43897123 .. 43897158 | 43,855,556..43,919,918 |  | element present inside the gene |
| 5 | CCAATGGGGAAGACATAGAGAATGAGGTGTTCCACG found at 1640174 line | VPS25P1 (Pseudo) | [441899 (VPS25P1)](http://www.ncbi.nlm.nih.gov/gene/441899) | vacuolar protein sorting 25 homolog (S. cerevisiae) pseudogene 1 | 118092423 .. 118092458 | 118,092,037..118,092,537 |  | element present inside the gene |
| 6 | CCAATCTACAGGCCCTTACTTTTCCCTCCTACCACG found at 2104632 line | TUFT1 | [7286 (TUFT1)](http://www.ncbi.nlm.nih.gov/gene/7286) | tuftelin 1 | 151533359 .. 151533394 | 151,512,781..151,556,059 |  | element present inside the gene |
| 7 | CCAATGTCCCTTAGGGGAGTAAAATTCCTCTCCACG found at 2393887 line | DNM3 | [26052 (DNM3)](http://www.ncbi.nlm.nih.gov/gene/26052) | dynamin 3 | 172359719 .. 172359754 | 171,810,618..172,387,606 |  | element present inside the gene |
| 8 | CCAATGTAGAAGCTGCTCATCTGGCCCAGGTCCACG found at 2983082 line | CENPF | [1063 (CENPF)](http://www.ncbi.nlm.nih.gov/gene/1063) | centromere protein F, 350/400kDa | 214781786 .. 214781821 | 214,776,522..214,837,914 |  | element present inside the gene |
| 9 | CCAATGCCATCTCAGTCCATCTCACAATGCCCCACG found at 3046436 line | LYPLAL1 | [127018 (LYPLAL1)](http://www.ncbi.nlm.nih.gov/gene/127018) | lysophospholipase-like 1 | 219343306 .. 219343341 | 219,347,192..219,386,207 | 3886 |  |
| 10 | CCAATACCTCCACCAGAGCAGGTGCTGGTATCCACG found at 428410 line | LCLAT1 | [253558 (LCLAT1)](http://www.ncbi.nlm.nih.gov/gene/253558) | lysocardiolipin acyltransferase 1 | 30845428 .. 30845463 | 30,670,102..30,867,091 |  | element present inside the gene |
| 11 | CCAATCTGCAGTATTGATTTGAAAGATGATGCCACG found at 1388388 line | EIF5B | [9669 (EIF5B)](http://www.ncbi.nlm.nih.gov/gene/9669) | eukaryotic translation initiation factor 5B | 99963820 .. 99963855 | 99,953,821..100,016,728 |  | element present inside the gene |
| 12 | CCAATAGGAATGGATGCTCCGACAGACGAAGCCACG found at 1470186 line | GPR45 | [11250 (GPR45)](http://www.ncbi.nlm.nih.gov/gene/11250) | G protein-coupled receptor 45 | 105853291 .. 105853326 | 105,839,595..105,860,085 |  | element present inside the gene |
| 13 | CCAATAAAAGCCCCACATTCATCCTTCAAGTCCACG found at 3163457 line | RHBDD1 | [84236 (RHBDD1)](http://www.ncbi.nlm.nih.gov/gene/84236) | rhomboid domain containing 1 | 227768755 .. 227768790 | 227,700,652..227,863,926 |  | element present inside the gene |
| 14 | CCAATACGAACGCTACAGCTTCCGCAGCTTCCCACG found at 460497 line | CRTAP | [10491 (CRTAP)](http://www.ncbi.nlm.nih.gov/gene/10491) | cartilage associated protein | 33155647 .. 33155682 | 33,155,450..33,189,265 |  | element present inside the gene |
| 15 | CCAATTCTAATTATTTAGGGGGAAAAGAAATCCACG found at 2078068 line | RNF13 | [11342 (RNF13)](http://www.ncbi.nlm.nih.gov/gene/11342) | ring finger protein 13 | 149620797 .. 149620832 | 149,530,475..149,679,926 |  | element present inside the gene |
| 16 | CCAATAGGTGCCGCGAAGCAGCAGCCCACCCCCACG found at 2556605 line | POLR2H | [5437 (POLR2H)](http://www.ncbi.nlm.nih.gov/gene/5437) | polymerase (RNA) II (DNA directed) polypeptide H | 184075410 .. 184075445 | 184,079,502..184,086,383 | 4092 |  |
| 17 | CCAATGACCACTTTTCTGGAAGACAGTTTTTCCACG found at 2616411 line | LPP | [4026 (LPP)](http://www.ncbi.nlm.nih.gov/gene/4026) | LIM domain containing preferred translocation partner in lipoma | 188381502 .. 188381537 | 187,871,097..188,608,460 |  | element present inside the gene |
| 18 | CCAATGCAGCAGCCGCACTTCCCCGTCTGACCCACG found at 107519 line | SORCS2 | [57537 (SORCS2)](http://www.ncbi.nlm.nih.gov/gene/57537) | sortilin-related VPS10 domain containing receptor 2 | 7741230 .. 7741265 | 7,194,374..7,744,564 |  | element present inside the gene |
| 19 | CCAATTACATGGTCCATTTTAGAATTAAGTGCCACG found at 1712647 line | ADAD1 | [132612 (ADAD1)](http://www.ncbi.nlm.nih.gov/gene/132612) | adenosine deaminase domain containing 1 (testis-specific) | 123310429 .. 123310464 | 123,300,121..123,350,957 |  | element present inside the gene |
| 20 | CCAATGTTTCCATTAATAGACAAGAGCACCACCACG found at 1953638 line | MGST2 | [4258 (MGST2)](http://www.ncbi.nlm.nih.gov/gene/4258) | microsomal glutathione S-transferase 2 | 140661831 .. 140661866 | 140,586,922..140,661,899 |  | element present inside the gene |
| 21 | CCAATCTGGTACCCACCCACCCACCCACCTACCACG found at 564994 line | PTGER4 | [5734 (PTGER4)](http://www.ncbi.nlm.nih.gov/gene/5734) | prostaglandin E receptor 4 (subtype EP4) | 40679469 .. 40679504 | 40,680,032..40,696,962 | 528 |  |
| 22 | CCAATAAAGCAGAATGGTTTGTGGAAATAACCCACG found at 1687305 line | ZNF474 | [133923 (ZNF474)](http://www.ncbi.nlm.nih.gov/gene/133923) | zinc finger protein 474 | 121485805 .. 121485840 | 121,465,215..121,489,266 |  | element present inside the gene |
| 23 |  | LOC100505841 | 100505841 (LOC100505841) | zinc finger protein 474-like | 121485805 .. 121485840 | 121,495,871..121,518,358 | 10066 |  |
| 24 | CCAATAAGCTTCCTGGTTGTGAAGGTCTCTGCCACG found at 1952201 line | PCDHB@ | [56116 (PCDHB@)](http://www.ncbi.nlm.nih.gov/gene/56116) | protocadherin beta cluster | 140558384 .. 140558419 | 140,430,979..140,627,802 |  | element present inside the gene |
| 25 |  | PCDHB8 | [56128 (PCDHB8)](http://www.ncbi.nlm.nih.gov/gene/56128) | protocadherin beta 8 | 140558384 .. 140558419 | 140,557,371..140,560,081 |  | element present inside the gene |
| 26 |  | PCDHB16 | [57717 (PCDHB16)](http://www.ncbi.nlm.nih.gov/gene/57717) | protocadherin beta 16 | 140558384 .. 140558419 | 140,560,980..140,566,710 | 2596 |  |
| 27 |  | PCDHB9 | [56127 (PCDHB9)](http://www.ncbi.nlm.nih.gov/gene/56127) | protocadherin beta 9 | 140558384 .. 140558419 | 140,566,893..140,571,111 | 8384 |  |
| 28 | CCAATTACTTTGCCTCTCTGTGCCATAATTTCCACG found at 491280 line | PPARD | [5467 (PPARD)](http://www.ncbi.nlm.nih.gov/gene/5467) | peroxisome proliferator-activated receptor delta | 35372018 .. 35372053 | 35,310,335..35,395,968 |  | element present inside the gene |
| 29 | CCAATGTGTATTTTCCCAGCATAAATTGGATCCACG found at 1856257 line | EYA4 | [2070 (EYA4)](http://www.ncbi.nlm.nih.gov/gene/2070) | eyes absent homolog 4 (Drosophila) | 133650366 .. 133650401 | 133,561,512..133,853,258 |  | element present inside the gene |
| 30 | CCAATCATTTCAAACACTCTGGAGTCAAAGTCCACG found at 777775 line | ZNF713 | [349075 (ZNF713)](http://www.ncbi.nlm.nih.gov/gene/349075) | zinc finger protein 713 | 55999667 .. 55999702 | 55,955,148..56,008,519 |  | element present inside the gene |
| 31 | CCAATGCACCTCCTGGTCTGTACTTTCAAGACCACG found at 2148128 line | DPP6 (Partial start) | [1804 (DPP6)](http://www.ncbi.nlm.nih.gov/gene/1804) | dipeptidyl-peptidase 6 | 154665091 .. 154665126 | 154,400,205..154,685,995 |  | element present inside the gene |
| 32 | CCAATACTGCTGGGCAACCCAGGGAGACCAGCCACG found at 19933 line | DLGAP2 | [9228 (DLGAP2)](http://www.ncbi.nlm.nih.gov/gene/9228) | discs, large (Drosophila) homolog-associated protein 2 | 1435076 .. 1435111 | 877,021..1,656,642 |  | element present inside the gene |
| 33 | CCAATACTATTTTGGCATTTCGTAGCACCAACCACG found at 787291 line | TGS1 | [96764 (TGS1)](http://www.ncbi.nlm.nih.gov/gene/96764) | trimethylguanosine synthase 1 | 56684861 .. 56684896 | 56,685,791..56,738,007 | 930 |  |
| 34 | CCAATCAGTCTTCCAAACAAGCCATTGTGGGCCACG found at 1392307 line | VPS13B | [157680 (VPS13B)](http://www.ncbi.nlm.nih.gov/gene/157680) | vacuolar protein sorting 13 homolog B (yeast) | 100246002 .. 100246037 | 100,025,299..100,890,447 |  | element present inside the gene |
| 35 | CCAATAATCTTTTCTCCTGAAAGCACTGTCACCACG found at 311732 line | DMRTA1 | [63951 (DMRTA1)](http://www.ncbi.nlm.nih.gov/gene/63951) | DMRT-like family A1 | 22444568 .. 22444603 | 22,446,840..22,452,472 | 2272 |  |
| 36 | CCAATGCCGTCTACTGGGCTGCTCGGCATGGCCACG found at 1253644 line | DAPK1 | [1612 (DAPK1)](http://www.ncbi.nlm.nih.gov/gene/1612) | death-associated protein kinase 1 | 90262227 .. 90262262 | 90,112,601..90,323,566 |  | element present inside the gene |
| 37 | CCAATAATCTGAAGCTTCAGAAGTCACAGTTCCACG found at 1621746 line | ZNF618 | [114991 (ZNF618)](http://www.ncbi.nlm.nih.gov/gene/114991) | zinc finger protein 618 | 116765609 .. 116765644 | 116,638,562..116,818,875 |  | element present inside the gene |
| 38 | CCAATATAGTACAATTTTATTACCTATAGGCCCACG found at 1638306 line | (DEC1) | [50514 (DEC1)](http://www.ncbi.nlm.nih.gov/gene/50514) | deleted in esophageal cancer 1 | 117957925 .. 117957960 | 117,904,097..118,164,923 |  | element present inside the gene |
| 39 | CCAATCTGGCTTCACGGCGGCGGAACTGACGCCACG found at 1896114 line | DBH | [1621 (DBH)](http://www.ncbi.nlm.nih.gov/gene/1621) | dopamine beta-hydroxylase (dopamine beta-monooxygenase) | 136520120 .. 136520155 | 136,501,485..136,524,466 |  | element present inside the gene |
| 40 | CCAATCGATGCACACAGAAAACTCCTCTGGGCCACG found at 368128 line | GAD2 | [2572 (GAD2)](http://www.ncbi.nlm.nih.gov/gene/2572) | glutamate decarboxylase 2 (pancreatic islets and brain, 65kDa) | 26505122 .. 26505157 | 26,505,236..26,593,491 | 114 |  |
| 41 | CCAATGTGGCAGCTCCCCCGTCCTGGGACATCCACG found at 1868937 line | INPP5A | [3632 (INPP5A)](http://www.ncbi.nlm.nih.gov/gene/3632) | inositol polyphosphate-5-phosphatase, 40kDa | 134563363 .. 134563398 | 134,351,283..134,596,984 |  | element present inside the gene |
| 42 | CCAATGGGAGCCGTGAGGAATGCTACTGGGGCCACG found at 4044 line | ATHL1 | [80162 (ATHL1)](http://www.ncbi.nlm.nih.gov/gene/80162) | ATH1, acid trehalase-like 1 (yeast) | 291064 .. 291099 | 288,480..297,511 |  | element present inside the gene |
| 43 | CCAATTGGGTTCCCGCCCATGTTATTGGCCCCCACG found at 89045 line | SMPD1 | [6609 (SMPD1)](http://www.ncbi.nlm.nih.gov/gene/6609) | sphingomyelin phosphodiesterase 1, acid lysosomal | 6411101 .. 6411136 | 6,411,644..6,416,228 | 543 |  |
| 44 | CCAATTTTTCTATTTTTAATACAGGGTTTCACCACG found at 254980 line | GTF2H1 | [2965 (GTF2H1)](http://www.ncbi.nlm.nih.gov/gene/2965) | general transcription factor IIH, polypeptide 1, 62kDa | 18358465 .. 18358500 | 18,343,816..18,388,590 |  | element present inside the gene |
| 45 | CCAATGACTTACTAGTTACTCACTGACTTGACCACG found at 815221 line | GLYATL1 | [92292 (GLYATL1)](http://www.ncbi.nlm.nih.gov/gene/92292) | glycine-N-acyltransferase-like 1 | 58695778 .. 58695813 | 58,695,102..58,724,543 |  | element present inside the gene |
| 46 | CCAATCTCAAAGTATTGCACACTGCATGACTCCACG found at 840465 line | MS4A15 (Partial stop) | [219995 (MS4A15)](http://www.ncbi.nlm.nih.gov/gene/219995) | membrane-spanning 4-domains, subfamily A, member 15 | 60513371 .. 60513406 | 60,524,340..60,544,204 | 10969 |  |
| 47 | CCAATGCGGCTGCCAAGACCACGGCCAGCAACCACG found at 887292 line | FLRT1 | [23769 (FLRT1)](http://www.ncbi.nlm.nih.gov/gene/23769) | fibronectin leucine rich transmembrane protein 1 | 63884902 .. 63884937 | 63,803,442..63,886,655 |  | element present inside the gene |
| 48 | CCAATCATAAATGGATATCCAAGACTGAAGTCCACG found at 1543365 line | C11orf53 | [341032 (C11orf53)](http://www.ncbi.nlm.nih.gov/gene/341032) | chromosome 11 open reading frame 53 | 111122162 .. 111122197 | 111,126,707..111,156,973 | 4545 |  |
| 49 | CCAATGCATGGAGCAGGTACAGTGCCTACCGCCACG found at 1703347 line | UBASH3B | [84959 (UBASH3B)](http://www.ncbi.nlm.nih.gov/gene/84959) | ubiquitin associated and SH3 domain containing B | 122640835 .. 122640870 | 122,526,398..122,685,187 |  | element present inside the gene |
| 50 | CCAATCTCAGTTCCAAAACGAAAAGGGATGGCCACG found at 366082 line | SSPN | [8082 (SSPN)](http://www.ncbi.nlm.nih.gov/gene/8082) | sarcospan | 26357798 .. 26357833 | 26,348,032..26,387,710 |  | element present inside the gene |
| 51 | CCAATCCAGAAATACCTGACTCCACAGACAGCCACG found at 636053 line | ANO6 | [196527 (ANO6)](http://www.ncbi.nlm.nih.gov/gene/196527) | anoctamin 6 | 45795680 .. 45795715 | 45,609,770..45,834,187 |  | element present inside the gene |
| 52 | CCAATTGACTCCACCTGTGCCTCACTTTCTCCCACG found at 689736 line | TUBA1C | [84790 (TUBA1C)](http://www.ncbi.nlm.nih.gov/gene/84790) | tubulin, alpha 1c | 49660860 .. 49660895 | 49,621,715..49,667,117 |  | element present inside the gene |
| 53 | CCAATTTTCCAATATATTTATCGAAAAAAATCCACG found at 960062 line | NUP107 | [57122 (NUP107)](http://www.ncbi.nlm.nih.gov/gene/57122) | nucleoporin 107kDa | 69124365 .. 69124400 | 69,080,731..69,136,473 |  | element present inside the gene |
| 54 | CCAATGGGTCAGTTGCTATAGAACAACAACACCACG found at 1091754 line | NAV3 | [89795 (NAV3)](http://www.ncbi.nlm.nih.gov/gene/89795) | neuron navigator 3 | 78606149 .. 78606184 | 78,224,685..78,606,790 |  | element present inside the gene |
| 55 | CCAATGAAACAGAATATACCCAGAAATAAAGCCACG found at 1106586 line | SYT1 | [6857 (SYT1)](http://www.ncbi.nlm.nih.gov/gene/6857) | synaptotagmin I | 79674093 .. 79674128 | 79,257,773..79,845,788 |  | element present inside the gene |
| 56 | CCAATTCAGGGGTCCTTGATCAGACAACCCTCCACG found at 1482168 line | TCP11L2 | [255394 (TCP11L2)](http://www.ncbi.nlm.nih.gov/gene/255394) | t-complex 11, testis-specific-like 2 | 106715955 .. 106715990 | 106,696,570..106,741,365 |  | element present inside the gene |
| 57 | CCAATACCTTTGTTTGAGTAGCCCATCGTTTCCACG found at 1497107 line | BTBD11 | [121551 (BTBD11)](http://www.ncbi.nlm.nih.gov/gene/121551) | BTB (POZ) domain containing 11 | 107791617 .. 107791652 | 107,712,197..108,053,419 |  | element present inside the gene |
| 58 | CCAATTTCAGCCTTCAGTTTTCTCAGAAATCCCACG found at 1526883 line | UBE3B | [89910 (UBE3B)](http://www.ncbi.nlm.nih.gov/gene/89910) | ubiquitin protein ligase E3B | 109935487 .. 109935522 | 109,915,215..109,974,510 |  | element present inside the gene |
| 59 | CCAATAGCTGAACTTAATTCATCTGTAGTTCCCACG found at 1527871 line | MVK | [4598 (MVK)](http://www.ncbi.nlm.nih.gov/gene/4598) | mevalonate kinase | 110006575 .. 110006610 | 110,011,500..110,035,071 | 4925 |  |
| 60 | CCAATGGAGGGACCGTCTGTGCGAGAACCGGCCACG found at 1578988 line | TPCN1 | [53373 (TPCN1)](http://www.ncbi.nlm.nih.gov/gene/53373) | two pore segment channel 1 | 113687009 .. 113687044 | 113,659,260..113,736,390 |  | element present inside the gene |
| 61 | CCAATTTGCACACGTGGCATCTCTAAGGGTCCCACG found at 1659605 line | SRRM4 | [84530 (SRRM4)](http://www.ncbi.nlm.nih.gov/gene/84530) | serine/arginine repetitive matrix 4 | 119491472 .. 119491507 | 119,419,300..119,600,856 |  | element present inside the gene |
| 62 | CCAATTTTGGAAGAGTTAGCTGGCAGAGTCTCCACG found at 595463 line | AKAP11 | [11215 (AKAP11)](http://www.ncbi.nlm.nih.gov/gene/11215) | A kinase (PRKA) anchor protein 11 | 42873206 .. 42873241 | 42,846,261..42,897,403 |  | element present inside the gene |
| 63 | CCAATAACAATCAAAATGTATATGTTAAATTCCACG found at 647455 line | CPB2-AS1 | [100509894 (CPB2-AS1)](http://www.ncbi.nlm.nih.gov/gene/100509894) | CPB2 antisense RNA 1 | 46616625 .. 46616660 | 46,626,983..46,675,482 | 10358 |  |
| 64 | CCAATGTACAACTCTCAATGCGGAGTTGCCGCCACG found at 726958 line | WDFY2 | [115825 (WDFY2)](http://www.ncbi.nlm.nih.gov/gene/115825) | WD repeat and FYVE domain containing 2 | 52340866 .. 52340901 | 52,158,484..52,340,935 |  | element present inside the gene |
| 65 | CCAATCAGCTGTCTTGAACACCCACTTTGATCCACG found at 957981 line | RAD51B | [5890 (RAD51B)](http://www.ncbi.nlm.nih.gov/gene/5890) | RAD51 paralog B | 68974479 .. 68974514 | 68,286,496..69,149,889 |  | element present inside the gene |
| 66 | CCAATGTGCTGAATTCATTGTCATTTAAACCCCACG found at 1103648 line | NRXN3 | [9369 (NRXN3)](http://www.ncbi.nlm.nih.gov/gene/9369) | neurexin 3 | 79462570 .. 79462605 | 78,636,716..80,334,633 |  | element present inside the gene |
| 67 | CCAATGCTATCCCTCCCCCATCCCCCCCACCCCACG found at 621470 line | CTDSPL2 | [51496 (CTDSPL2)](http://www.ncbi.nlm.nih.gov/gene/51496) | CTD (carboxy-terminal domain, RNA polymerase II, polypeptide A) small phosphatase like 2 | 44745753 .. 44745788 | 44,719,579..44,819,455 |  | element present inside the gene |
| 68 | CCAATGGCCACCCCTCACACCACAAGCCAAGCCACG found at 38775 line | SRRM2 | [23524 (SRRM2)](http://www.ncbi.nlm.nih.gov/gene/23524) | serine/arginine repetitive matrix 2 | 2791656 .. 2791691 | 2,802,330..2,821,413 | 10674 |  |
| 69 | CCAATATTTCTCAAGTTCCCTTTTCAGTATGCCACG found at 106672 line | RBFOX1 | [54715 (RBFOX1)](http://www.ncbi.nlm.nih.gov/gene/54715) | RNA binding protein, fox-1 homolog (C. elegans) 1 | 7680280 .. 7680315 | 5,289,469..7,763,342 |  | element present inside the gene |
| 70 | CCAATCAGAGGTAACTTCAGTTTTTCATCTGCCACG found at 308703 line | EEF2K | [29904 (EEF2K)](http://www.ncbi.nlm.nih.gov/gene/29904) | eukaryotic elongation factor-2 kinase | 22226476 .. 22226511 | 22,217,592..22,300,066 |  | element present inside the gene |
| 71 | CCAATCCCTGCTTTAAAATGCAGAACTAGGACCACG found at 435908 line | ITGAX | [3687 (ITGAX)](http://www.ncbi.nlm.nih.gov/gene/3687) | integrin, alpha X (complement component 3 receptor 4 subunit) | 31385248 .. 31385283 | 31,366,488..31,394,320 |  | element present inside the gene |
| 72 | CCAATGCACAGCTGGCTGAGCAGGCCTGCCCCCACG found at 251971 line | LLGL1 | [3996 (LLGL1)](http://www.ncbi.nlm.nih.gov/gene/3996) | lethal giant larvae homolog 1 (Drosophila) | 18141780 .. 18141815 | 18,128,907..18,148,189 |  | element present inside the gene |
| 73 | CCAATTCAGCCACTAATACCTTTGTATACTACCACG found at 736687 line | STXBP4 | [252983 (STXBP4)](http://www.ncbi.nlm.nih.gov/gene/252983) | syntaxin binding protein 4 | 53041317 .. 53041352 | 53,046,119..53,241,646 | 4802 |  |
| 74 | CCAATGGAGCTGAACGTCAGCGCCTCTGTCCCCACG found at 909542 line | PITPNC1 | [26207 (PITPNC1)](http://www.ncbi.nlm.nih.gov/gene/26207) | phosphatidylinositol transfer protein, cytoplasmic 1 | 65486898 .. 65486933 | 65,373,397..65,693,379 |  | element present inside the gene |
| 75 | CCAATTAGGAATGGGAGCCTTGCTCACAGCCCCACG found at 1029819 line | RNF157-AS1 | [100507218 (RNF157-AS1)](http://www.ncbi.nlm.nih.gov/gene/100507218) | RNF157 antisense RNA 1 | 74146870 .. 74146905 | 74,136,637..74,150,729 |  | element present inside the gene |
| 76 | CCAATCAGAAGGACTTCCAATTTTCCATCTGCCACG found at 1117633 line | FOXK2 | [3607 (FOXK2)](http://www.ncbi.nlm.nih.gov/gene/3607) | forkhead box K2 | 80469429 .. 80469464 | 80,477,594..80,562,483 | 8165 |  |
| 77 | CCAATGTGATCCTCCTGTCTTCCTGGGCCCCCCACG found at 35662 line | NDC80 | [10403 (NDC80)](http://www.ncbi.nlm.nih.gov/gene/10403) | NDC80 kinetochore complex component | 2567531 .. 2567566 | 2,571,510..2,616,634 | 3979 |  |
| 78 | CCAATGCAAGGCTTCAGGAACAATTCCAGAGCCACG found at 122564 line | SOGA2 | [23255 (SOGA2)](http://www.ncbi.nlm.nih.gov/gene/23255) | SOGA family member 2 | 8824474 .. 8824509 | 8,705,659..8,832,776 |  | element present inside the gene |
| 79 | CCAATGTCCACTGCTGGCACGCTGGGCCAAGCCACG found at 782547 line | MALT1 | [10892 (MALT1)](http://www.ncbi.nlm.nih.gov/gene/10892) | mucosa associated lymphoid tissue lymphoma translocation gene 1 | 56343290 .. 56343325 | 56,338,618..56,417,371 |  | element present inside the gene |
| 80 | CCAATAGGAGGGGCGAATGACTCCACTGAGGCCACG found at 34391 line | GADD45B | [4616 (GADD45B)](http://www.ncbi.nlm.nih.gov/gene/4616) | growth arrest and DNA-damage-inducible, beta | 2476043 .. 2476078 | 2,476,123..2,478,257 | 80 |  |
| 81 | CCAATGTCCTACTGGTCATATAGTGAGCATCCCACG found at 180263 line | MAST1 | [22983 (MAST1)](http://www.ncbi.nlm.nih.gov/gene/22983) | microtubule associated serine/threonine kinase 1 | 12978828 .. 12978863 | 12,949,259..12,985,766 |  | element present inside the gene |
| 82 | CCAATTTGCCTAAGCAAGACCTTCCGGGCATCCACG found at 554370 line | PLEKHG2 | [64857 (PLEKHG2)](http://www.ncbi.nlm.nih.gov/gene/64857) | pleckstrin homology domain containing, family G (with RhoGef domain) member 2 | 39914532 .. 39914567 | 39,903,222..39,919,055 |  | element present inside the gene |
| 83 | CCAATCCCCTGAGCCCTGTGCAAATCAGACACCACG found at 598097 line | LIPE-AS1 (Partial stop) | [100996307 (LIPE-AS1)](http://www.ncbi.nlm.nih.gov/gene/100996307) | LIPE antisense RNA 1 | 43062866 .. 43062901 | 42,901,300..43,156,507 |  | element present inside the gene |
| 84 | CCAATATTGATACATTATTATTAACTAAAGTCCACG found at 617299 line | ZNF221 | [7638 (ZNF221)](http://www.ncbi.nlm.nih.gov/gene/7638) | zinc finger protein 221 | 44445376 .. 44445411 | 44,455,375..44,471,752 | 9999 |  |
| 85 | CCAATGTTATTACAAGACCTCACACCAGCAGCCACG found at 681863 line | SULT2B1 | [6820 (SULT2B1)](http://www.ncbi.nlm.nih.gov/gene/6820) | sulfotransferase family, cytosolic, 2B, member 1 | 49093996 .. 49094031 | 49,055,429..49,102,684 |  | element present inside the gene |
| 86 | CCAATTAAAGATGATTTTTACAGTCAATGAGCCACG found at 64818 line | PRNP | [5621 (PRNP)](http://www.ncbi.nlm.nih.gov/gene/5621) | prion protein | 4666794 .. 4666829 | 4,666,797..4,682,235 |  | element present inside the gene |
| 87 | CCAATCGGCGGTGCCCGCGCAGGGTGCTACGCCACG found at 324207 line | GZF1 | [64412 (GZF1)](http://www.ncbi.nlm.nih.gov/gene/64412) | GDNF-inducible zinc finger protein 1 | 23342767 .. 23342802 | 23,342,769..23,353,683 |  | element present inside the gene |
| 88 | CCAATCTCTTCTCGTGATAATGAGGGAGTTCCCACG found at 506238 line | CTNNBL1 | [56259 (CTNNBL1)](http://www.ncbi.nlm.nih.gov/gene/56259) | catenin, beta like 1 | 36448995 .. 36449030 | 36,322,357..36,500,531 |  | element present inside the gene |
| 89 | CCAATAGATCCATGTGCAAACAGATCAGGGGCCACG found at 807643 line | PHACTR3 | [116154 (PHACTR3)](http://www.ncbi.nlm.nih.gov/gene/116154) | phosphatase and actin regulator 3 | 58150208 .. 58150243 | 58,152,564..58,422,766 | 2356 |  |
| 90 | CCAATCTGAGCTCTTGTCTTTTCAAACCCCACCACG found at 839697 line | CDH4 | [1002 (CDH4)](http://www.ncbi.nlm.nih.gov/gene/1002) | cadherin 4, type 1, R-cadherin (retinal) | 60458065 .. 60458100 | 59,827,482..60,515,673 |  | element present inside the gene |
| 91 | CCAATTCCCATCCCCCAGCAGCGTGTTAGCGCCACG found at 656350 line | PCBP3 | [54039 (PCBP3)](http://www.ncbi.nlm.nih.gov/gene/54039) | poly(rC) binding protein 3 | 47257049 .. 47257084 | 47,268,023..47,362,368 | 10974 |  |
| 92 | CCAATGTAGAAGCTGCTCATCTGGCTCAGGTCCACG found at 312113 line | IGL | [3535 (IGL)](http://www.ncbi.nlm.nih.gov/gene/3535) | immunoglobulin lambda locus | 22472004 .. 22472039 | 22,380,474..23,265,085 |  | element present inside the gene |
| 93 | CCAATATCTTTAGCTGGGAATGGAAATATCCCCACG found at 437878 line | INPP5J | [27124 (INPP5J)](http://www.ncbi.nlm.nih.gov/gene/27124) | inositol polyphosphate-5-phosphatase J | 31527086 .. 31527121 | 31,518,893..31,530,683 |  | element present inside the gene |
| 94 | CCAATCAGAGGTACTTTCAGTTTTTCATCTGCCACG found at 656855 line | TBC1D22A | [25771 (TBC1D22A)](http://www.ncbi.nlm.nih.gov/gene/25771) | TBC1 domain family, member 22A | 47293410 .. 47293445 | 47,158,514..47,571,342 |  | element present inside the gene |
| 95 | CCAATTCTCCTGGCGTTTAGAAGACAAATCACCACG found at 657876 line | TBC1D22A | [25771 (TBC1D22A)](http://www.ncbi.nlm.nih.gov/gene/25771) |  | 47366965 .. 47367000 | 47,158,514..47,571,342 |  | element present inside the gene |
| 96 | CCAATCTGGTGGCTCTTTTAGCCGACTTTAGCCACG found at 176747 line | FRMPD4 | [9758 (FRMPD4)](http://www.ncbi.nlm.nih.gov/gene/9758) | FERM and PDZ domain containing 4 | 12725664 .. 12725699 | 12,156,585..12,742,642 |  | element present inside the gene |
| 97 | CCAATTATGCATTCTGGCACTGGGGAAATGACCACG found at 1491516 line | ATG4A | [115201 (ATG4A)](http://www.ncbi.nlm.nih.gov/gene/115201) | autophagy related 4A, cysteine peptidase | 107389047 .. 107389082 | 107,334,899..107,397,901 |  | element present inside the gene |
