# Supplementary material for: Identification of ASB7 as ER stress responsive gene through a genome wide in silico screening for genes with ERSE
Source: PLoS One. 2018 Apr 9;13(4):e0194310. doi: 10.1371/journal.pone.0194310 (PMC5890977; doi:10.1371/journal.pone.0194310)
Supplement: S4 Table — (DOCX) [file pone.0194310.s004.docx]

**S4 Table**

Gene/gene and Protein–Protein interactions of ASB gene family using cytoscape**:**

| **Gene** | **Interactions:** |
| --- | --- |
| ASB1 | TCEB1,TCEB2,RNF7,CUL5,SSBP1,SNRPD1,CASQ2,TRMT10C,CPS1,HMGB1,ASB12,ASB5,CPS1,EDTUD2,ATP2A2,SSR4 |
| ASB2 | ESR2,HSP90AB1,RAB40b,RNF7,CUL5,SMAD9,CUL2,JAK2,CAND1,ROA1,RL35A,VIME,TFE2,HNRH1,JAK3,MLL1,PROF1,SSBP,UBC,ELOB,ELOC,CK084,RAN,SKP2,HNRPK,PPM1A,HMGB1,SMD1 |
| ASB3 | AGL,ATP5C1,DNPEP,ERH,SFPQ,PRDX6,RIN3,HSP90AA1,TNS2,THRAP3,TCEB1,PGAM5,ZAP70,SLC4A1AP,RPS27,NONO,ASB6,CLASP2,DCUN1D1,TCEB2,SLC25A5,PRMT6,RCN2,CUL5,P05109,KDM1A,EPB41L3,TNFRSF1BSRP14,HAP90AB1,SH2D2A,ATP5F2D2A,ATP5F1 |
| ASB4 | HSPB1,ID2,RPS27L,HIF1AN,TUBB3,CUL2,GPS1,RIT1,CUL4A,TUBB8,UBED2D1,ASB7,IRS4,HSP90AA1,STUB1,TFRC,TUBB4A,DNAJB4,HSP90AB1,TCEB2,HSPB8,DNAJB5,FARSA,CUL5,RNF7,HSPD1,CHIP |
| ASB5 | ATP2A2,ATP5B,AKR1B1,PRKDC,PTS,CUL5,TCEB1,ASB1,BSG,SLC3A2,ENO2,ATP5C1,HIF1An,DNAJA1,PPP2CB,RAB1A,SLIRP,LRPPRC,TCEB2,RPN1,CRYAB,GCN1,ALDR,ELOB,ELOC |
| ASB6 | FGA,RNF41,XPNPEP1,HSP90AA1,CUL5,TCEB2,CUL4B,HSPB1,EZR,RNF7,HDGF,HIF1AN,COPS5,SH2B2,FH,TCEB1,FGG,ASB12,FGB,DCUN1D1,PROSC,ASB3,OGFOD1,FAM9B,CADPS2,ATIC,MCTS1,APP,HAP90AB1,ELOC,FIBA |
| **ASB7** | HIF1AN,RNF7,**ATF4**,**ASB4,**ASB12,CUL5,SRRM1,CEP170,CCDC136,CCT7,GCN,GANAB,SEPT1,**JUN**,TCEB2,VCL,HMBOX1,FGA,FGB,FGG,ELOC |
| ASB8 | RCN1,SERTAD1,TRIM11,TCEB2,MCM7,HIF1AN,SLC25A13,RIN3,SLC25A6,NEDD8,NPAT,TCEB1,TUBB3,CUL5,KIF26B,ZC3H6,AMBRA1,HOXD4,TMED10,PCNA,SERTAD1,ELOB,CMC2 |
| ASB9 | CKM,TCEB1,ANXA5,LDHA,CUL5,VIM,TLN1,CKB,ACTB,ENO2,TCEB2,HIST1H2BN,HSPD1,ANXA2,CRK,ASS1,AKR1B1,PGAM1,HSPA4,PGK1,UBB,PLCG2,UBED2D1,TCEB3,HELS29,ACO2,RAB1A,CKMT1B,HIF1A,PRPSAP1,HIST4H,ARIH2,SUMO1,KPRA,KCRB,ALDR |
| ASB10 | STUB1,RNF7,PGAM5,RPS10,TCEB1,TUBB6,HSPA1A,TCEB2,SLC25A1,PSMA4,MRPL23,SLC25A5,PSMA4,RPL38,EMD,TUFM,KDM1A,HSPB1,CUL5,SLC25A3,ARFGAP1,AGR3,CPVL,ARL1,AP1G1,MEOX2,MPCP,CHIP,TBB6,MLL1,ELOB,ELOC,EFTU,CPVL,AP1G1, |
| ASB11 | DDOST,ATP5B,GANAB,PSMD2,RPN1,CUL5,PSMC3,SLC3A2,UBC,PRPS1,PSMB2,PSMD11,ELIN1,PSMA2,OST48, |
| ASB12 | GLO1,ASB1,TCEB2,CADPS2,SFPQ,TCEB1,DHX38,DHX36,RCN2,CUL5,SRRT,UBA1,TUFM,PFKFB3,FGG,PFN2,RNF7,KRT8,LZTR1,RBM6,CKB,PRDX6,MTHFD1L,ASB7,NUDT21,ASB2,UBED2D1,ASB6,MYH10,RPL38,PFN1,AIFM1,ACTB,NFE2,HOXA2,MYOCD |
| ASB13 | RNF7,DNM1,POLR3A,HIF1AN,NUDT3,VAC14,SSBP1,DUSP23,CUL5,TCEB2,TRIP13,ZMIZ2,TCEB1,MARK3,CCT8,ELOC,DYN1,ELOB |
| ASB14 | ARF4,TUFM,CYC1,PGAM5,TCEB1,NOTCH2,PHB2,TCEB2,SLC25A1,RPL23,CUL5,ATP2A2,SSR4,GALK1,SLC25A6,VDAC1,VAPA,SLC25A5,EMD |
| ASB15 | FTH1,CUL5,VIM,DHRS2,TCEB2,DHRS2,SVIl,USP9X,GAPDH,RPS16,ACLY,WDR77,TCEB1,PRPF8,IPO5,WDR61,GPI,DLD,MIF |
| ASB16 | SRC,NCK1,ABL1,GRB2,FYN,HIF1AN,HIST1H2BN,PGAM1,VIM,SRC,ABL1,GRB2,SVIL,CSE1L,HIST1H1C,P0C0S5,TCEB1,CKB,RAN,HIST4H4,CUL5,TCEB2,FTH1,AHCY,GAPDH,HSPA1A,P84253,ELOCSAHH,XPO2,ELOB |
| ASB17 | HSP90AB1,TUBA1B,TCEB2,TUBB3,ATP2A2,SLC25A1,SSR4,HSPB1,TUBB,PHB2,HSP90AA1,RPL23,VAPA,VDAC1,TUFM,TUBB2B,TUBB4B,ARF4,CPD,RCN2,ELOB2,SSRD,TBB3,DAC1,EFTU,RL23,TBB5 |
| ASB18 | MLL1,CUL5,TCEB1,TCEB2,NONO,TUBB3,NME1,PRSP1,SFPQ,BCLAF1,SLC25A6,ASS1,ERH,THRAP3, TFG, AKR1B1, ABLIM1,ASS1,DHRS2,RPL15,AKR1B1,RBM6 |
