# Supplementary figures and images for: Identification of ASB7 as ER stress responsive gene through a genome wide in silico screening for genes with ERSE
Source: PLoS One. 2018 Apr 9;13(4):e0194310. doi: 10.1371/journal.pone.0194310 (PMC5890977; doi:10.1371/journal.pone.0194310)

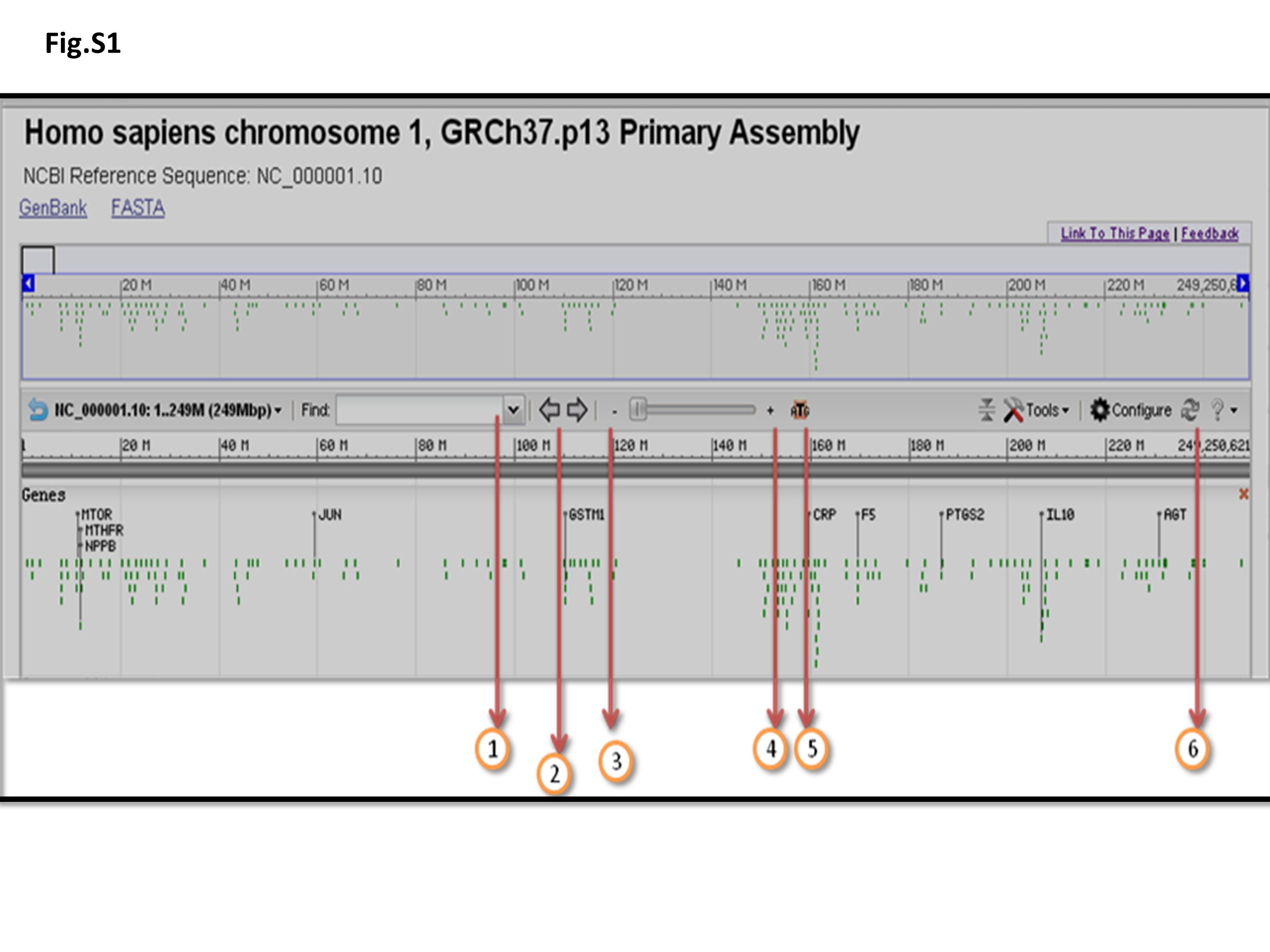

Supplement: S1 Fig — (TIF) [file pone.0194310.s005.TIF]

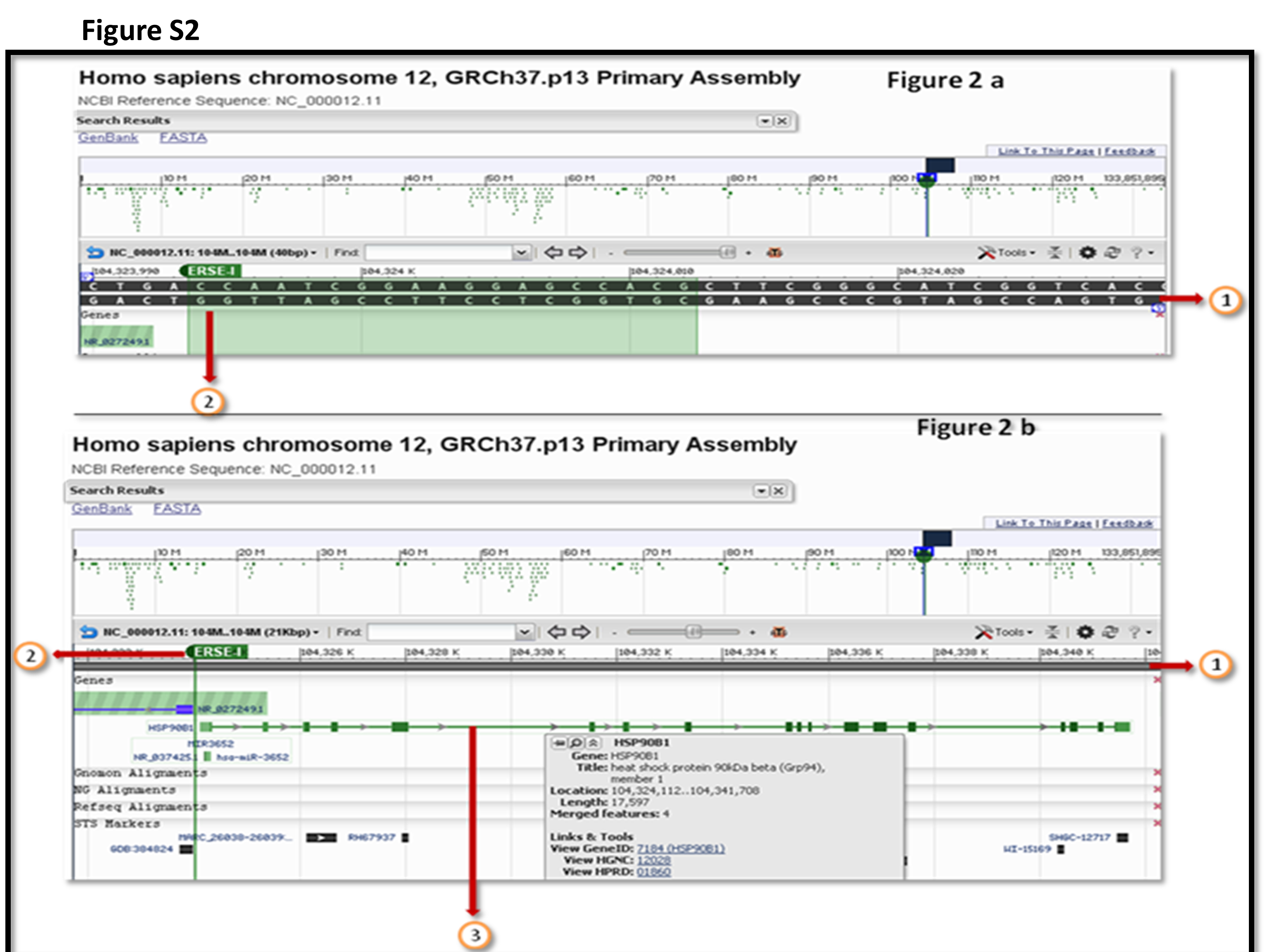

Supplement: S2 Fig — (TIF) [file pone.0194310.s006.TIF]

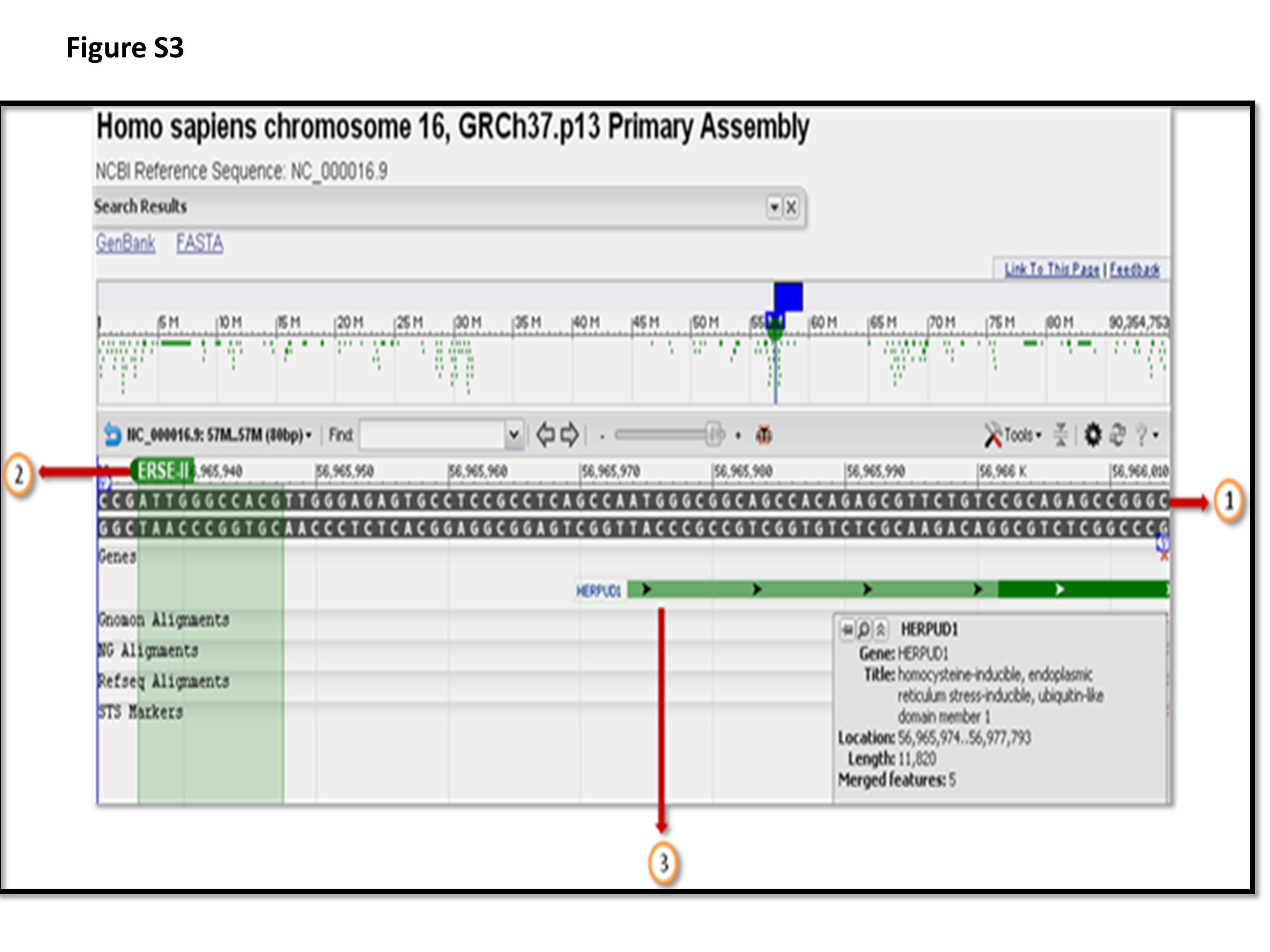

Supplement: S3 Fig — (TIF) [file pone.0194310.s007.TIF]

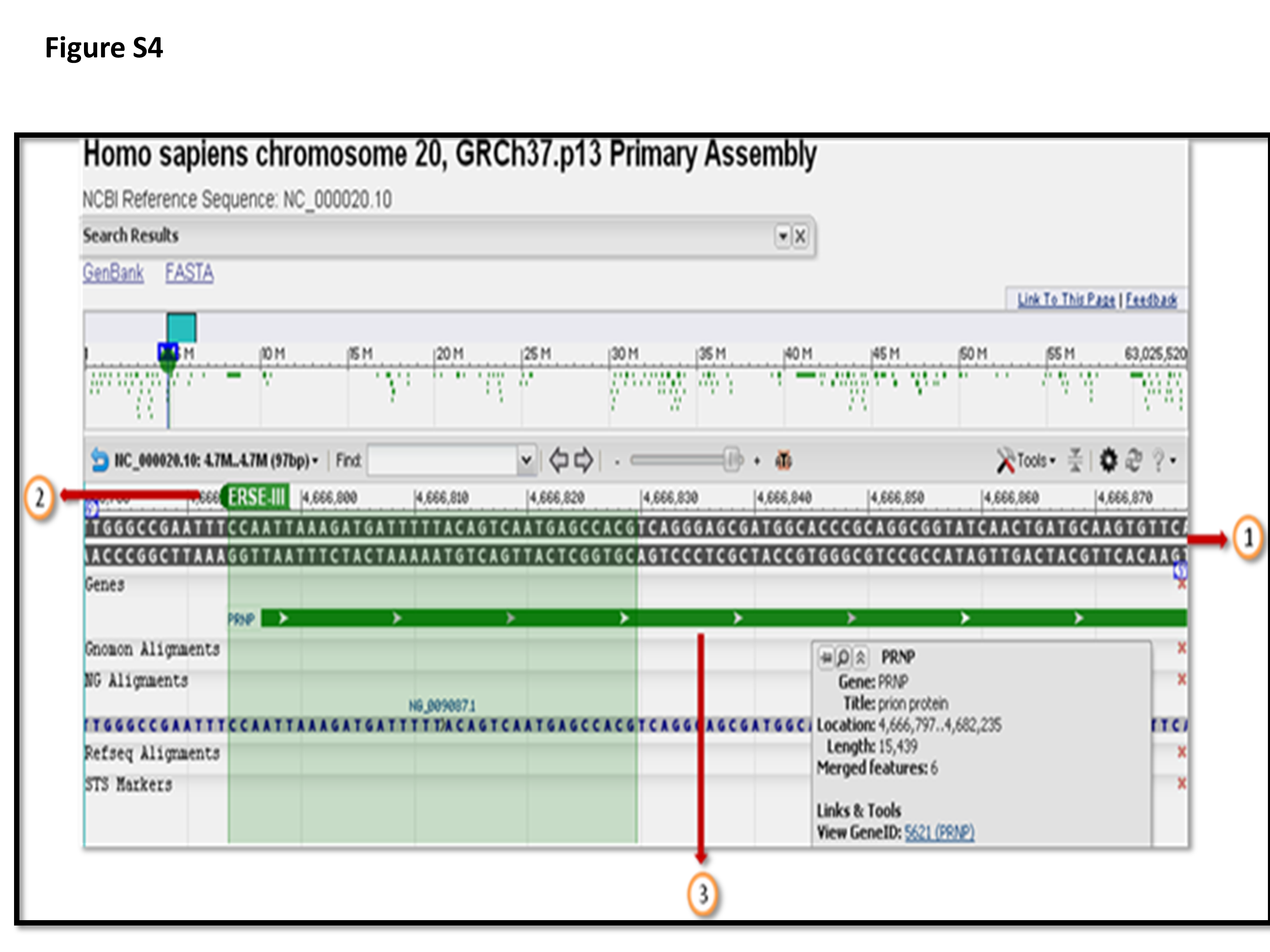

Supplement: S4 Fig — (TIF) [file pone.0194310.s008.TIF]

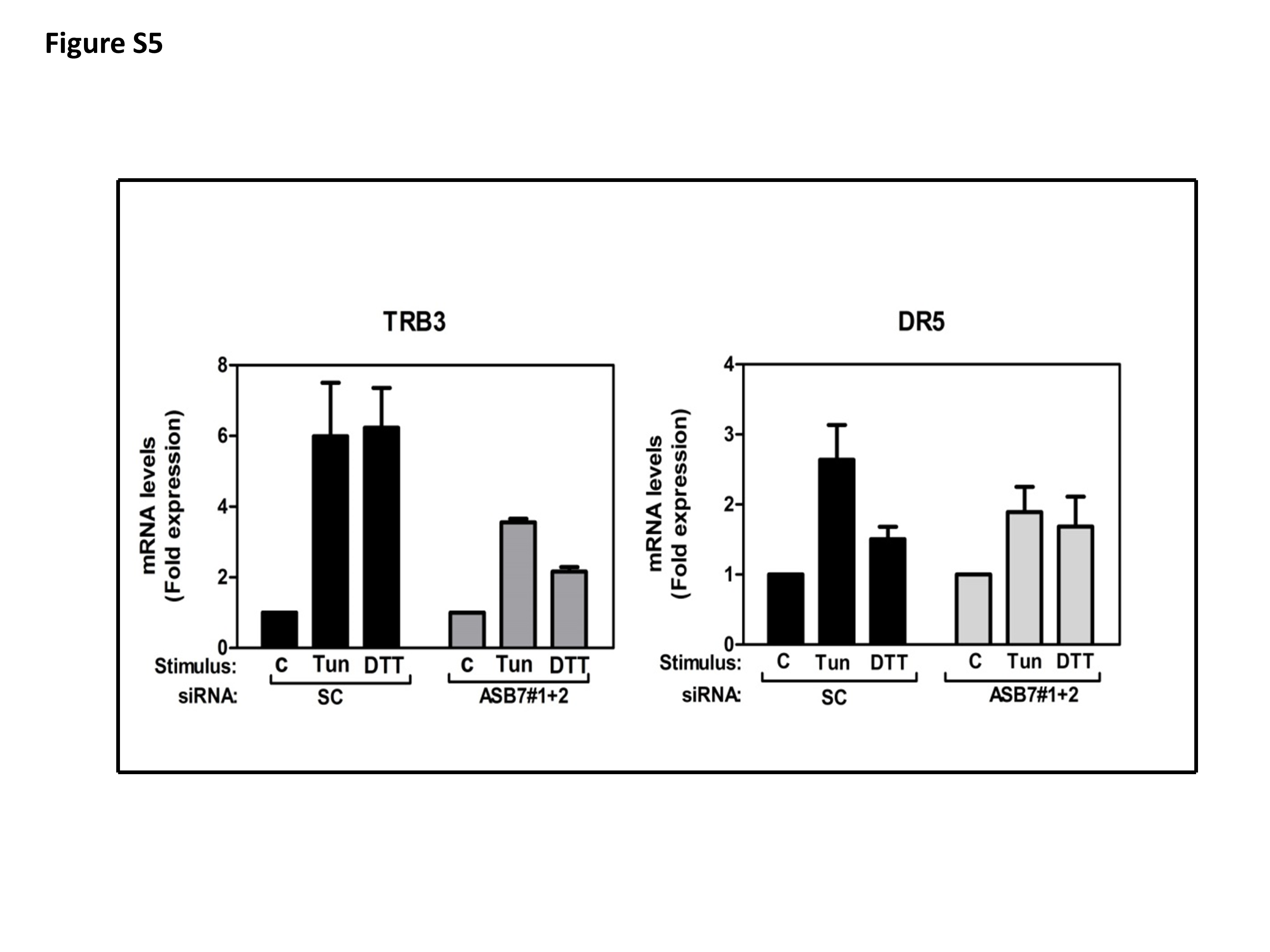

Supplement: S5 Fig — (TIF) [file pone.0194310.s009.TIF]
